# Supplementary material for: Wisconsin dairy farm worker perceptions and practices related to antibiotic use, resistance, and infection prevention using a systems engineering framework
Source: PLoS One. 2021 Dec 16;16(12):e0258290. doi: 10.1371/journal.pone.0258290 (PMC8675684; doi:10.1371/journal.pone.0258290)
Supplement: S1 Fig — The questionnaire guide developed to conduct the semi-structed focus group interviews. (PDF) [file pone.0258290.s001.pdf]

SEMI-STRUCTURED INTERVIEW/ FOCUS GROUP GUIDE

Questions Block 1: Getting to know the participant/ building a rapport.
Interviewer: First would you mind telling us a little information about yourself?

1. What is your age?
2. How would you describe your race?
    a. How about your ethnicity? (Hispanic, Latino, Neither)
3. How long have you been working on [insert facility name]?
4. Tell us, what kinds of jobs do you do on an average day
5. How long have you been doing this type of work on this farm?
    a. Have you worked on any other dairy farms? Where at? For how long?
6. Do you ever feel rushed doing all the work you need to get through in a day?
7. What is your favorite part of your job?

Question Block 2: Defining the Operations Organizational Structure
Interviewer: Now we would like to talk with you about how this farm is organized and run.

1. Do you get a lot of direction about how to do your job(s)? Who typically gives you direction?
    a. How many people are considered your manager/boss?
    b. *For leadership*: How do you typically communicate with this person/ these people?
2. Do you have someone you can ask questions?
    a. How comfortable are you asking questions at work?
3. IF a Spanish speaker:
    a. Are the people you ask questions and report to able to speak Spanish with you?
    b. Are you comfortable speaking English at work?
    c. Are there any barriers to your ability to communicate with you boss or manager or other coworkers?
4. Are there people you work with who do not speak the same language as you?
    a. How do you communicate with these coworkers?
    b. How effectively do you feel you are able to communicate with them?
5. Where/ from whom do you most commonly get information about what is happening on the farm?

Interviewer: Now we would like to ask you some questions about your health and the health of the cows on this farm.

Question Block 3: Knowledge of Antibiotics and Farm Policies
Interviewer: First, we would like to ask you some questions about antibiotics. Antibiotics are medicines doctors use to help a person who has an infection with bacteria get better. Veterinarians also use these medicines to help animals with infections get better. Are you familiar with antibiotics?

1. What do you think about antibiotics?
2. Has a doctor ever explained to you how to take them?
3. How easy is for you to get antibiotics if you think you need one?
4. Where do you usually get your antibiotics?
5. Have you ever gotten and infection and taken antibiotics that didn’t make you feel better?

Interviewer: Antibiotics sometimes stop working and are no longer able to kill or control the growth of bacteria in a person or animal. Because of this, that antibiotic may no longer work to treat the person or animal’s infection. This is called antibiotic resistance. Another way to say this is antibiotic resistance happens when the bacteria can resist the effect of the drug and continue to grow, even when the drug is being taken. Antibiotic resistance can affect both humans and animals. Are you familiar with antibiotic resistance?

6. In your daily life, how have you heard or discussed antibiotics and antibiotic resistance?
7. Have you or a loved one ever gotten an infection with an antibiotic resistant bacteria?
8. Do you think you or your family members are at risk of getting an antibiotic resistant infection?

9. How do you think dairy farms like this one are affected by antibiotic resistance, if at all?
    a. Do you think they play a role in antibiotic resistance? Could you tell me why you think yes/no?
    b. Do you think stringent rules and protocols about antibiotic use on the farm benefits the animals and/or the community?
10. What are the current policies on your farm about antibiotic use? Can you describe the current antibiotics policies to me as you understand them? How do you find out about the policies and if anything changes?
11. Who on the farm is allowed to use antibiotics on the animals?

Question Block 4: Personal Protective Equipment and Hygiene.
Interviewer: Now I'd like to ask you some questions about your daily work on the farm and the clothes you wear when you're at work.

1. Do you think there are things you can wear at work to help protect yourself?
    a. Follow-up: Can you give us some examples?
2. What are some of the things you do on the farm to help keep you and the cattle healthy?
3. What are some of the things you are required to wear on this farm to protect your health? Do you have a uniform you are supposed to wear? If so, does the farm provide it for you?
   [if the participant needs guidance on what we are looking for, things like coveralls, boots, gloves, eye protection, face masks/filter masks, shoe covers, etc.)
4. IF participant wears gloves:
    a. When do you wear gloves when you are on the farm?
    b. What kind of gloves do you wear? (Leather, latex, nitrile…)
    c. How often do you change *(if disposable)* or wipe off or wash *(If leather or cloth)* your gloves?
5. IF participant wears coveralls or other protective clothing:
    a. When do you wear [clothing item] on the farm? All the time or for specific tasks?
    b. How often do you wash the clothing you wear when on the farm?
6. Do you wash your hands during the work day? When are the times you are most likely to wash your hands?
    a. What do you use to wash your hands (soap/water, liquid or gel hand sanitizer, antibacterial wipes)
    b. Are there easily accessible places to wash your hands when you feel you need to?
    c. Does washing your hands ever interfere with your ability to complete your assigned duties?
7. Does your employer have any policies about hand washing or wearing any special protective equipment? If so, what are the policies?
    a. How does your employer tell you about these policies?
    b. How do you feel about wearing protective gear?
8. How do you get ready to leave work for the day? Is there a locker room for you to use?
9. Do you wear your work clothes and shoes home? What do you do with them?
10. Do you ever do any work with calves or sick cattle? If yes:
    a. Do you wear any special or different clothing or equipment when you work with these animals?
        i. How effective do you think wearing protective clothing is at keeping you healthy?
    b. When you work with calves or sick cattle, do you feel you are at risk of getting an illness from them?
11. How do you feel about wearing things like gloves, coveralls, or protective eyewear?

Question Block 5: Misc. Habits
Interviewer: For the remainder of our time, I'd like to ask you a few more questions about daily life and activities on the farm.

1. Where do you eat your meals while at work? Is there a breakroom or specific place you like to eat your meals?
2. How are raw milk and raw milk products handled on the farm?
3. Do you or other farm workers ever consume raw milk or raw milk products? If yes, what types?
4. Are there any rules on the farm about consuming raw milk products?
5. Do you feel there are any risk with drinking raw milk or eating products made with raw milk?
