# Supplementary material for: Wisconsin dairy farm worker perceptions and practices related to antibiotic use, resistance, and infection prevention using a systems engineering framework
Source: PLoS One. 2021 Dec 16;16(12):e0258290. doi: 10.1371/journal.pone.0258290 (PMC8675684; doi:10.1371/journal.pone.0258290)
Supplement: S2 Fig — Checklist used to conduct direct observations on farms. (PDF) [file pone.0258290.s002.pdf]

Direct Observations Checklist Instrument

Observer Name:________________________________ Date: ___ ___ / ___ ___ / ___ ___ ___ ___ Start Time: ___ ___ : ___ ___ am / pm

Farm ID: ____ End Time: ___ ___ : ___ ___ am / pm

**Zone: Entry/ Parking**	☐Not Applicable
Limiting non-essential traffic on farm[1]	☐Yes ☐No
Clearly designated visitor entry	☐Yes ☐No
Visitor sign-in	☐Yes ☐No
Disposable PPE including boot covers (or boot wash) provided	☐Yes ☐No
**Zone: Breakroom/ Kitchen**	☐Not Applicable
Boot cleaning station nearby	☐Yes ☐No
Refrigerators used for food storage only	☐Yes ☐No
Clean tables for eating /food prep	☐Yes ☐No
Sinks with soap and drying towels or blowers	☐Yes ☐No
Microbiology or Nutrition lab present	☐Yes ☐No
Signage reminding workers about proper hand hygiene	☐None ☐English ☐Spanish
Notes:	
**Zone: Bathroom Facilities**	☐Not Applicable
Boot wash nearby	☐Yes ☐No
Shower present	☐Yes ☐No
What type(s) of hand hygiene are provided:<br>☐Hand Sanitizer ☐Soap/ Water ☐Paper towels ☐ Air dryer	
Are hand hygiene instructions provided?<br>☐English ☐Spanish ☐None	
Notes:	

**Zone: Locker Facilities.**	☐Not Applicable
Boot wash station nearby	☐Yes ☐No
Access to shower facilities with soap	☐Yes ☐No
Notes:	
**Zone: Laundry**	☐Not Applicable
Instructions on proper use	☐None<br>☐English<br>☐Spanish
Instructions to use high heat and tumble dry	☐Yes ☐No
Cleanliness of the washing machine<br>☐Clean ☐Mold/mildew present ☐Noticeable malodor<br>☐Visibly soiled	
Does the farm use an off-site service for laundry?<br>☐Yes ☐ No ☐ Partial:_______________________	
What is laundry room used for?	☐Clothing<br>☐Milking Towels
Washing machine operational	☐Yes ☐No
Dryer operational	☐Yes ☐No
Are washer and/or dryer commercial/industrial or residential type	☐Commercial<br>☐Residential
Where are the laundry facilities located?	
Notes:	

**Zone: Milk Room/Production**	

**Zone: Adult Lactating Cows**	☐Not Applicable

Easily accessible gloves	☐Yes ☐No
Are milking technicians wearing gloves	☐Yes ☐No
Are milking technicians wearing plastic aprons	☐Yes ☐No
Are milking technicians wearing coveralls	☐Yes ☐No
Are milking technicians wearing safety glasses	☐Yes ☐No
Do milking technicians have arm protection	☐Yes ☐No
Cleanliness of bulk tank area<br>Signage designating clean area<br>☐ Yes ☐No	☐Clean<br>☐Some soiling<br>☐Visibly soiled
Eye wash station	☐Yes ☐No
Are soiled PPE garments changed prior to changing milkhouse activities	☐Yes ☐No
Cloth towel or disposable wipes?	☐Cloth towels<br>☐Disposable wipes
What type(s) of hand hygiene are provided:<br>☐Hand Sanitizer ☐Soap/ Water ☐Paper towels ☐ Air dryer<br>Are hand hygiene instructions provided?<br>☐English ☐Spanish ☐None	
Boot cleaning station nearby	☐Yes ☐No
Notes on milking production:	

**Zone: Medicine Storage**	☐Not Applicable
Locked entry	☐Yes ☐No
Controlled access	☐Yes ☐No
Gloves/hand hygiene present	☐Yes ☐No
Plastic/disposable obstetrical sleeves available	☐Yes ☐No
Notes:	

Storage for equipment used only around healthy lactating cows	☐Yes ☐No
Notes:	

**Zone: Sick Cow Pen/ Hospital/ Isolation**	☐Not Applicable
Boot wash station or disposable boot covers	☐Yes ☐No
Easily accessible gloves	☐Yes ☐No
Are workers either able to change their clothing or provided with protective clothing?<br>☐Yes, changing facility ☐Yes, coveralls provided or plastic aprons<br>☐No ☐Other:________________________	
Are workers wearing PPE?<br>☐Gloves ☐Boot Covers ☐Coveralls or Aprons ☐No ☐N/A<br>☐Other: ________________________	
What type(s) of hand hygiene are provided:<br>☐Hand Sanitizer ☐Soap/ Water ☐Paper towels ☐ Air dryer ☐No<br>Are hand hygiene instructions provided?<br>☐English ☐Spanish ☐None	
Notes:	

**Zone: Calving Pen/Maternity** (Cont. next page)	☐Not Applicable
Boot wash station nearby	☐Yes ☐No
Easily accessible gloves	☐Yes ☐No
Access to hand hygiene	☐Yes ☐No
Are workers wearing PPE	☐Yes ☐No
What type(s) of hand hygiene are provided:<br>☐Hand Sanitizer ☐Soap and Water ☐Paper towels<br>☐ Air dryer	

**Zone: Calving Pen/ Maternity** (cont.)	☐Not Applicable

**Zone: Cull Cows and Trucks**	☐Not Applicable

Are hand hygiene instructions provided?
☐English ☐Spanish ☐None

Are workers wearing PPE?
☐Gloves ☐Boot Covers ☐Coveralls or Aprons ☐No ☐N/A

☐Other: ______________________________

Notes:

**Zone: Baby Calf Hutches/ Housing**	☐Not Applicable
Boot wash station or disposable boot covers	☐Yes ☐No
Easily accessible gloves	☐Yes ☐No

Are workers wearing PPE?
☐Gloves ☐Boots or Covers ☐Coveralls or Aprons ☐No

☐Other: ______________________________

What type(s) of hand hygiene are provided:
☐Hand Sanitizer ☐Soap/ Water ☐Paper towels ☐ Air dryer

Are hand hygiene instructions provided?
☐English ☐Spanish ☐None

Restricted access to calf housing?	☐Yes ☐No
Equipment storage only for calves (e.g. shovels, buckets, halters, etc.)	☐Yes ☐No

Notes:

How are carcasses disposed of?
☐Disposal on site ☐Licensed Collection Company
If on site: ☐Burial ☐Incineration ☐Composting ☐Rendering

Protective clothing provided
☐Coveralls ☐Aprons ☐Gloves ☐Boot covers ☐None ☐NA

Are workers required to change clothing prior to handling other animals	☐Yes ☐No
Are there signs limiting who and what vehicles can be in the zone?	☐Yes ☐No
Is there a place to clean trucks and tires	☐Yes ☐No

What type(s) of hand hygiene are provided:
☐Hand Sanitizer ☐Soap/ Water ☐Paper towels ☐ Air dryer

Are hand hygiene instructions provided?
☐English ☐Spanish ☐None

Notes:

**Zone: Dry Cows**	☐Not Applicable
Are gloves easily accessible	☐Yes ☐No
Boot wash station or disposable boot covers	☐Yes ☐No
Are workers using gloves	☐Yes ☐No

What type(s) of hand hygiene are nearby:
☐Hand Sanitizer ☐Soap/ Water ☐Paper towels ☐ Air dryer

Are hand hygiene instructions provided?
☐English ☐Spanish ☐Both

Notes

**General Biosecurity Observations:**		**Notes:**
Do workers change or launder clothes or shoes prior to leaving the farm?	☐Yes ☐No	

Are coveralls provided for workers?	☐Yes ☐No	
Are boots or shoe coverings provided for workers?	☐Yes ☐No	
Where do workers typically eat?		
Are personal vehicles able or allowed to be driven where animals may walk or be transported?	☐Yes ☐No	
How often are employees encouraged to change PPE?		
Obstetrical chains?		
Hot water heater heats to temp and supplies sufficient hot water		

Which of the following disinfectants, if any, did you see on the farm?

☐Bleach/ chlorine based (Clorox) ☐Chlorhexidine (Nolvasan) ☐ Iodophors (Betadine/ Weladol) ☐Oxidizers (Virkon/ Oxy-Sept 333) ☐Ammonium (Roccal D Plus) ☐ Phenolic (Pine-Sol, One Stroke, Osyl) ☐No ☐Other:

Other Observations
