# Supplementary material for: Wisconsin dairy farm worker perceptions and practices related to antibiotic use, resistance, and infection prevention using a systems engineering framework
Source: PLoS One. 2021 Dec 16;16(12):e0258290. doi: 10.1371/journal.pone.0258290 (PMC8675684; doi:10.1371/journal.pone.0258290)
Supplement: S1 File — Full dataset used for analysis. (PDF) [file pone.0258290.s003.pdf]

Focus Group 1, Farm 1
Recording in English and Spanish

MODERATOR: Like we told you, this is going to be confidential. So while we're talking, please avoid using names since we're recording.

TRANSLATION: *So the idea is that you can't, that you don't use names. Please, avoid using names. What else?*

MODERATOR: Try only one person talk at a time.

TRANSLATION: *That you try to just speak one person at a time, not all at once.*

MODERATOR: And please silence your phones.

TRANSLATION: *And if you could, silence your phones.*

MODERATOR: But if you need to get up to take a phone call or go to the bathroom, feel free.

TRANSLATION: *If you need to take a call or go to the bathroom, feel free to take the call.*

MODERATOR: So to get started, I just want to learn a little bit more about the farm and what you do here.

TRANSLATION: *So to get started, she wants to know a little bit about what you guys do on the farm, your jobs.*

MODERATOR: So if we can go around, and, please, tell me about your role on the farm, how long you've worked here, and any other farming experience you've had.

TRANSLATION: *So we're going to go around the circle . . . if you don't mind, if you could describe what your role is on the farm, what job do you do, how long have you been working here, and if you have any other experience on another farm.*

MODERATOR: Okay. Do you want to start?

WOMAN: Sure. Okay. So you want to know what I do?

MODERATOR: Mm-hmm.

WOMAN: Okay. So I don't like, I guess I don't have like a specific like name for it, but I like feed all the calves in the morning. And then scrape barns and just do like if it, pumping manure, like I sit by the pump and do that or drive tractors, just a lot of different things. And like I said before, I've worked here for about two months. But it's just kind of all the little stuff around here is pretty much what I do, but . . .

MODERATOR: Thank you.

TRANSLATION: *Well, what is your job on the farm? What do you do? How long have you worked here?*

TRANSLATION: *I've only been here a week.*

INTERPRETER: *A week.*

MAN: *But before that, I had been working on this farm for a year. My job here is to take care of the cows, corral the cows and all that, clean the pens, and milk, and also take care of the calves. I haven't don't all that here, but I've done those jobs on other farms. Yeah, but I've just been here one week.*

INTERPRETER: *One week. Okay.*

MAN: *Yeah.*

INTERPRETER: Do you want me to translate it or . . .

MODERATOR: You can just general gist.

INTERPRETER: Yeah, so he has been here like for a week now. But he worked before, previously worked here. So he was in charge of like cleaning the barns and to secure the cows and the calves and, yeah, different.

MODERATOR: Okay.

MAN: *I've been here for approximately eight months. I have worked on two other farms. My job here is to corral, which consists of cleaning the cow bedding and send them to the parlor at 9:00 to be milked. And also, milking is my other job. Be watching to see if any cows are sick or have an illness like mastitis, which is the most common. So, well, check the cow and separate her so that she can go to be treated. On this farm, this is my job.*

INTERPRETER: Okay. He has been here nine months, but he has worked in two other farms before. His main job is corralear, like clean the beds of the cows. And like he, how do you say that in English? Like he brings the cows to the milking.

MODERATOR: Okay. Transport.

INTERPRETER: Yeah. And he sees a cow maybe has mastitis, so he separates the cow, and he gives notices that the cow is sick or something.

WOMAN: *I've been working here for two years. At the beginning, yeah, I was milking cows. Now, I just work with the calves. My job is to take care of the calves from the time they are born. And I treat them when they are sick. And I teach them to eat. That's it.*

INTERPRETER: She has been here two years. She started working milking cows, but now she is in charge of the calves. So she takes care of the calves, like from the start of when they are born. So she feeds them, and she takes, like she cures them. Like she treats the cows too if they're sick.

MODERATOR: Okay. Now, I'd like to learn a little bit more about what a typical shift on, or a day at the farm is like here.

INTERPRETER: *She wants to know what a typical shift here is like on the farm, like what are the activities that you do in a typical day.*

WOMAN: *A day of work here?*

INTERPRETER: *Yeah, like a normal day.*

WOMAN: *Oh, well, what they do is very different. My job, my day is just with the calves, 7:00 in the morning to 6:00 in the evening, just with the calves. What can I say? I'm there. When they are sick, I give them medicine.*

INTERPRETER: *You feed them?*

WOMAN: Alimentarlos en la mañana, en la tarde, limpiarles sus corrales. Y nosotros somos las que jalamos becerros de acá al otro rancho. Y como le digo, yo soy la que estoy siempre con ellos cuando se enferman, siempre, diario limpiando corrales, limpiando y lavando corrales para llevar becerros nuevos, y al mismo tiempo, viendo los enfermos. Los alimento en la mañana, y los alimento en la tarde. Ese es mi día de trabajo.
TRANSLATION: *Feed them in the morning and in the afternoon, clean their pens. And we are the ones that take the calves from here to the other farm. And like I said, I'm the one that is always with them if they are sick, always, every day, cleaning pens, cleaning and washing pens to bring in new calves, and at the same time, looking after the sick ones. I feed them in the morning, and I feed them in the afternoon. That is my workday.*

INTERPRETER: Yeah. She's focused on the calves. So she feeds them. If they are sick, then she treats them with medication and transports them like to the, I forgot the word.

MODERATOR: That's okay.

INTERPRETER: Yeah. So she's like focused, like 100% of her day is focused on the calves.

MODERATOR: Okay.

MAN:*Well, here, in my case, it depends on the day if I have to work in the pens or if I have to milk. So if I have to milk, well, I have to be here at 5:45 . . . machine, turn on the system. And then, well, I go to the pens, send the cows over here, and if I have to milk, well, connect the machines to the cows and clean off the teats, right, and stick the machines on. That is the whole day, eight hours of work if I have to work eight hours. If I have to work 12, well, then 12 hours working in a day.*

When I have to work in the pens, I work at 5:45. My job is to take the cows from the pens and send them to the milkers. From there, I stay back and clean the bedding and the walkways where the cows walk. Well . . . for that, depending on, well, also, I forgot to say that before I work in the pens, I'm also working where the cows are giving birth, if a cow is having a calf.

Also you are responsible for taking care of the cows, helping get the calf out, feeding the first feed, which we give, right, before . . . for . . . that's if a cow gives birth. And after that, we have to go put out the food for the cows . . . feed. And that is the daily routine for working in the pens.

INTERPRETER: Okay. So he has two functions. Some days, well, he start at 5:45 a.m. every day.

MODERATOR: *Very early.*

INTERPRETER: He can be like full-time milking the cows all day. So came in to clean the cows and like put the milking machines and everything all day. Sometimes he's like 8 hours, sometimes he's 12 hours. Or the other function is like he has to take care of the beds like clean their beds and like the rows. And he sees if a cow is giving birth, like a cow is in labor or something, so he take care of the cows, and, yeah.

MODERATOR: Cool.

MAN: *Right now, I don't have a lot to do. I just milk. Yeah, since I just got here, there are other workers now. But I know how to do all that stuff that he does. I know it. But they already have their jobs to do. So I just milk for now.*

INTERPRETER: Okay.

MAN: *Yeah.*

INTERPRETER: He's in charge of milking, milking the cows.

MODERATOR: Okay.

INTERPRETER: He knows what to, like how to do the other things because has . . . on another farm. But now his only function is milking cows.

MODERATOR: Okay.

WOMAN: Well, like I said earlier, I get here at, it kind of depends on the time, but no later than 8:00. Then I go and I feed the wean barn first, and I lay all the corn and hay out. And then I go and take buckets of corn over to graze on the other side. And then I come back and scrape barns, and I mean, after that, it's kind of whatever they need me to do, I do. Like, I mean, Wednesdays is we take, or we haul cattle back and forth. So that can take like eight hours, it can take, it kind of just depends how long. But it's, like I said, it's just after I'm done scraping barns, it's just kind of a whatever I need to do, I do. Yeah.

MODERATOR: Okay. Thank you. So what do you do if you have a question about how to do your job?

INTERPRETER: *What do you do if you have a question about your job, who do you ask or how, what do you do if you have a question or a concern?*

WOMAN:

In my case, I just work, there are just two of us. On the calf farm, there are just two. The one that knows more about medicine and illnesses is my partner. When they are really sick, she is the one who takes care of them. Now, if they have a fever, they don't want to eat, I know what to give

them. If they, I mean, I don't need to ask. I know how to get them up, what's the word? the first, I mean, to put it that way.

If they have been sick for a while, they are very, very sick, and they don't get better with what I give them, medicine for fever, their first injections to fight some bacteria, then I give them electrolyte to get them up, like fluids, if they are very . . . now. After, like I said, if it's really serious, then she takes care of them.

INTERPRETER: *And that's when you ask your partner?*

WOMAN: *I ask her. Well, really, she does it.*

INTERPRETER: Okay. So with the calves, only work two. So usually, if the cows are sick, she knows what to do. So she . . . like she does it. And if she has like a very sick cow, and she doesn't know what to do, she asks her partner, the other person that is in charge of the calves.

MAN: *In my case, when I have to work in the pens, if I have any difficulties or any questions, well, I always ask . . . they are always alone.*

INTERPRETER: Okay.

MAN: *But if I do have a question that I'm having trouble making a decision on, I have two options, call Linda, the owner, or talk to the most experienced person that is on the farm. If he can tell me over the phone, well . . . the question. If not, well, I have to call Linda or . . .*

INTERPRETER: Okay. So when he has a doubt, he asks the like the person that has been working there for a longer time. Or he usually is alone, so he has to figure it out by himself if he has a difficult question or a doubt he asks his partner or he calls the owner.

MODERATOR: Okay.

INTERPRETER: Yeah, when it's like pretty like . . .

MODERATOR: Okay.

MAN: *Yeah, in my case, well, it's the same as her. Yeah, because we basically do the same thing. And, well, Linda is almost always here. And, well, she . . . or the one who has been working here the longest. Yeah, if nobody is around like the owner or her kids, yeah, always . . . working here, he can tell us what to do.*

INTERPRETER: So he usually asks like, it's the same as him. He asks the one that has been working a long time here. Or he asks the owner.

MODERATOR: Okay.

WOMAN: Usually, I just have to figure it out myself. I don't really, I mean, I can call my co-workers, but they're usually doing things that are more important than what I'm doing. So I just kind of get, have to be brave about it and figure it out even if it means that I'm going to end up doing something wrong, I've got to figure it out sometimes.

[Man speaking in Spanish to someone else at same time as woman].

[inaudible conversation going on in background between interpreter and Spanish-speaking man]

MODERATOR: When do you talk with your manager or management?

INTERPRETER: *When do you talk with your supervisor or manager?*

WOMAN: *There is no manager here.*

INTERPRETER: Oh.

WOMAN: *There is no manager here. Whatever we need, we would talk to the owner about it.*

INTERPRETER: Okay. They don't have a manager. They go directly to the owner.

MODERATOR: Oh, okay. So I guess when, you said you talk to the owner when you have questions. Besides that, what other times do you talk to the manager, or the owner? Sorry.

INTERPRETER: *So you talk to the owner whenever you have questions, but are there any other circumstances where you talk with her, or just when you have questions?*

MAN: *It's hard to answer that question because when it's about work, well, we already said before that the person with the most experience, I mean, because like she says, there isn't a manager here. So, well, I have to . . . with the person with the most experience if it's about work. If it's something personal, then it's with the owner.*

INTERPRETER: Okay.

MAN: *Yeah.*

WOMAN: *Overall . . . us.*

INTERPRETER: Okay. So if they have like a personal thing, they go with the owner, but like, most of the time they asked their more-experienced coworker, or they try to figure out by themselves what to do.

MODERATOR: Do you feel the same?

WOMAN: Well, it's kind of, I guess, a little different for me because I'm around like the owner all the time. I wouldn't say we like work together, like I'm not like working with him, it's just like I'm around him a lot because I just do stuff. But I will mainly do it if there's like, I started making lists in my phone now that are every like sick animal that I see. Because I'm around the barn so much, I like make a list of the numbers of all the cows that I see that are limping have, just if they have problems in general. Or and then I'll share that list with them. If there's a dead cow, then I go, and I say something about that. But other than that, I just see them all the time. So I just, whatever, I mean, yeah.

MODERATOR: Okay. How do breaks work on the farm?

INTERPRETER:*Okay. How do breaks work, or how does time off work here on the farm, if you have time off?*

WOMAN: *Time off?*

INTERPRETER: **Y***eah, or, I mean, your break.*

WOMAN: When I'm hungry, I go take lunch.

[Simultaneous discussion]

WOMAN: *I have a break. One hour.*

INTERPRETER: *One hour?*

WOMAN: *Yeah.*

INTERPRETER: *Just one hour a day?*

WOMAN: Uh-huh.

INTERPRETER: *And if you want, for example, to go to the bathroom or something like that?*

WOMAN: *Oh, yeah.*

INTERPRETER: She usually has a one-hour break, like for lunch and everything. And if she wants to use the bathroom, she can go.

MODERATOR: Okay. Is the one-hour break scheduled?

INTERPRETER: *Is it scheduled at that time, like at a specific time?*

WOMAN: *Yes, at lunch.*

INTERPRETER: Okay. Tiene una hora de lunch entonces . . . They have a specific lunchtime.
TRANSLATION: *Okay. You have an hour for lunch then . . . They have a specific lunchtime.*

MODERATOR: Okay, and does, you stay on the farm for that, or leave?

INTERPRETER: *And do you eat lunch here on the farm, of do you go somewhere?*

WOMAN: *I live close to the farm. The house is over here. So right now, I have the ability to go home and eat. Before, we had to eat here.*

INTERPRETER: Okay. Yeah, she goes to her house. She lives very close. So she goes to her house. But before that, she came here to eat.

MODERATOR: Okay. How about everyone else?

INTERPRETER: *Yeah, same?*

MAN: *No, in our situation, what the milkers and the people working in the barns don't have a break when we work eight hours. For example, we get in at 6:00 in the morning, the shift is until 2:00 in the*

afternoon. So there isn't a lunchbreak. But if we have to go to the bathroom, go to, we have breakfast here. We have a microwave.

So if we bring something to eat, well, we can heat it up and . . . in the parlor, and try to curb the hunger a little. And when you work 12 hours, which is from 6:00 to 6:00, well, then you take a break because if you work quickly, that is the hour they wash everything, yeah, then you have an hour. So at that time, you can go get something to eat provided that you work fast. If you don't work fast, then you don't have time . . . get behind . . . you don't have time to eat.

INTERPRETER: *Is it the same for you?*

MAN: *Of course.*

INTERPRETER: So they have the eight-hour shift from 6:00 to 2:00, so they don't have a lunch break. So they usually will like take breaks for like going to the bathroom or eat something quick because they have a refrigerator and a microwave. So sometimes they eat like and take breaks. And if they have a 12-hour shift, they have like a, they eat like in the middle of the work. Like, for example, if they are cleaning the machines, they take that hour like to eat. But it's like very flexible.

MODERATOR: Oh, okay. And where do you eat?

INTERPRETER: *Where do you eat when you can eat? Where, do you have like a kitchen or?*

MAN: *When we work 12 hours?*

INTERPRETER: Mm-hmm.

MAN: *Yeah, there is a place to eat. Like I said, as long as you make the time to have lunch. If we have to eat a sandwich or something in the parlor or something there in the back well . . . a little bit . . . because we are with the cows and . . .*

INTERPRETER: Okay. If they don't, if they have time, they go to the, like they have a place to eat.

MODERATOR: Like a break room?

INTERPRETER: Yeah. But usually, they don't have a lot of time to eat, so they eat like in the same room, like working and eating because they don't have much time.

MODERATOR: Okay. Thank you. Is that similar?

WOMAN: Well, it's, I mean, like I said, like if I'm hungry, I'll, I started, this is a really good job to where I've just like started to bring snacks with me everywhere I go. Like it doesn't matter if I'm driving like the truck, the tractor, anywhere, I always have snacks in my pocket because I don't know when I'm going to get a lunch.

But, I mean, most of the time, if the days are really slow and laid back, I can be like, hey, I'm going to go on lunch real quick. And then I just take like a half an hour lunch. But there are days that I have worked from like 6:00 in the morning until 11:30 at night, where I've had like maybe a 20-minute period for like, lunch, bathroom, everything, just like, because I'm so busy doing so much stuff throughout the day that it just depends on the day, I guess.

MODERATOR: What rules does the farm have in terms of what you wear?

INTERPRETER: *What rules does the farm have in terms of what clothing you have to wear to work?*

WOMAN: *It depends on how cold it is. It depends on the cold if we put on the clothes we need. They don't require us . . .*

INTERPRETER: *But it's your clothing, your own clothing, or do they give you something?*

WOMAN: *There are uniforms here, so to speak, uniforms, you know, like clothing from the farm that we can, it's really just for the men. The other day they told us that if us women wanted to wear it, that was okay. But, no, we wear our own clothing.*

INTERPRETER: She usually wears her clothes. It depends on the cold. Like they have some clothes for the workers. But like she usually use her own clothes to work.

MAN: *Yeah, well, here is, like she said, there are uniforms for the farm. But it's for whoever wants to wear it.*

INTERPRETER: *It's voluntary.*

MAN: *It's voluntary. There aren't any rules that you have to use a uniform. That you have to use any kind of protection for milking or for working. No, it's whatever you want. So you could come in whatever you think is convenient and helps you do your job.*

INTERPRETER: Yeah, so they have uniforms here. But it's like, if they want to use them, it's okay. But if they don't want, they use their own clothes how they feel comfortable.

MODERATOR: Okay.

INTERPRETER: *Oh, sorry. Is it the same for you?*

MAN: *Well, It's the same, yeah. It's the same.*

INTERPRETER: Yeah, he's the same.

MODERATOR: Okay. Yeah. The same?

WOMAN: Pretty much the same. Yeah.

MODERATOR: Okay. Do you have to wear long pants?

INTERPRETER: *Do you have to wear long pants?*

WOMAN: *Yeah.*

INTERPRETER: *Yes?*

WOMAN: *Yes, because we're always walking around in the . . . and all that. I use, well, I don't hardly use a boot . . . yeah, rubber boots and long pants.*

INTERPRETER: She usually wears the long pants and the boots.

MODERATOR: Okay.

MAN: *Yeah, for the . . . for the job, if I have, well, I use the rubber boots, long pants, but it's not required. I mean, for example, if I feel like coming in shorts to work, well, nobody is going to kick me out of the parlor for wearing shorts or tennis shoes. It's not a rule on the farm.*

WOMAN: *If you come in shorts, yeah, because last time, there was an employee that decided to come in shorts, and they told him, no, no, don't come to work in shorts.*

MAN: *Oh, well, I didn't know that. From what I understand, there are no rules. I mean, I've been working here for eight, nine months. They've never given me a document saying, here are the rules.*

INTERPRETER: Okay.

MAN: *Yeah, there is a paper there that says, well . . . gone away. But . . . for clothing or anything like that. It's just about the way the farm works and the way . . .*

INTERPRETER: Mm-hmm. Yeah, they don't have a rule on how to dress. Like they are free like to wear. They usually wear pants because, and their boots because they are like in barns and everything. But they don't always, they don't have any rules or like any specific. It's not mandatory to wear pants or like . . .

MODERATOR: Okay. Any other thoughts?

WOMAN: I would definitely wear pants. Like, I mean, if they told me I can wear, I mean, they do kind of tell you, you can wear whatever you want in a sense. But I don't think I'd ever want to wear anything else because . . .

MODERATOR: Okay. Are there rules about protective gear, like in situations where you have to wear coveralls, shoe covers, gloves, eye protection, face mask?

INTERPRETER: *Is there a time where you have to have, like you guys are required to use eye protection, or an overall, or shoe covers or something very specific like that?*

MAN: *In our case, well, it's like you work with . . . for example, when I started working in the pens, my job was to prepare the water for the cows' hooves, so we used them there. They said because, I don't have . . . yeah. The powder that they use to mix, that mix is. Chemical that . . . so they say that there are masks and safety glasses, but they don't require me to wear them. They don't require it. They don't provide me with the means to say . . . the times I've prepared it, I'm careful . . .*

INTERPRETER: *Gloves.*

MAN: *Well, I'm careful. Gloves because we always use cloves at work here. But they just told me that that we have those kinds of things to use, but I've never seen them.*

INTERPRETER: Yeah, he said that he knows that for some specific tasks like, for example, prepare it's like water with chemicals for the cows' feet, they have to like maybe wear masks or some things. But he has never seen those. And like, yeah, like they don't have a mandatory like . . . ¿Usted igual?

WOMAN: *I don't have any experience with that.*

INTERPRETER: She said like she can wear whatever she wants. She doesn't have like any rules.

MAN: *I think that on the farm, the only place where there is a little bit of risk is in the preparation of that, that moment, you know, preparing the water for the cows' hooves because across the whole farm . . . the work, there isn't really anything that is putting your life in danger . . . to use some kind of . . .*

INTERPRETER: Yeah, he said like also, their work is very safe. So like so he thinks that he doesn't have any risk to wear like protection or something like that.

MODERATOR: What about like gloves when they're milking or anything like that?

INTERPRETER: *You use gloves, right, to milk?*

MAN: *We use gloves for everything.*

INTERPRETER: Yeah, gloves are like, they use gloves all the time.

MODERATOR: Okay.

INTERPRETER: *You too?*

WOMAN: *Yes, I always use gloves.*

INTERPRETER: She, the gloves are, yeah . . .

MAN: *Yeah, gloves are always used on the farm.*

INTERPRETER: Okay.

MAN: *Yeah, and . . .*

INTERPRETER: *And always . . .*

MAN: *Yeah, because he liquid that they put on the cow is called dip. It's very, it's not good for your skin. So you need to always have gloves on.*

INTERPRETER: Yeah, they wear gloves all the time because of the, like the liquid that they use for like cleaning . . .

MODERATOR: Okay, yeah. You're talking about . . .

INTERPRETER: When they are milking and everything, so they use gloves all the time.

MAN: *Well, the safety glasses are almost, on some farms, they require safety glasses because there are times where like a bottle of dip could get into your eyes. That burns a lot, and it's really bad. If you get a lot in your eye, you can lose your vision . . . so some farms require that you have to wear them. But . . . humid . . . so here, well, maybe before they wanted you to wear them, I think when I was here before. But people don't really like them, yeah. But, yeah, on some farms, they require them.*

INTERPRETER: He said that some other farms like they ask for them to use like eye protection like to milking cows. But here, they don't use them anymore because it can be like, people can use it. Like it's kind of, how do you say, awkward.

MODERATOR: Okay.

INTERPRETER: So they prefer not to use it. So here it's kind of . . .

MODERATOR: Anything you do different?

WOMAN: Just like about power washing equipment. They recommend we wear like rain gear, but you don't have to. It's either you get wet, or you don't, and it's your own choice.

MODERATOR: Do they have that for you?

WOMAN: Yeah, I mean, they have some stuff, but most choose not to use it. It's just, yeah.

MODERATOR: Anything different with sick cattle, in terms of gear that they wear?

INTERPRETER: *Any special clothing that you have to wear when you're working with sick cows?*

WOMAN: No.

MAN: No.

INTERPRETER: *You either, if you have a sick cow or something, is there some kind of special clothing you need to wear?*

MAN: *For the people who work in the barns when . . . a cow has a calf inside, and we don't know if it's coming, what position the calf is in, then, yes, we have to put on long gloves, up to here, use a liquid on your hands so you don't hurt the cow, right. And you can't put your hand in if you're not wearing that glove because of infection that you can give the cow. So that's the only thing.*

INTERPRETER: They only like wear the long gloves when they have problems with like when the cows is in labor, if they have to like go and try to fix it, but that's it.

MODERATOR: What are your handwashing practices at work?

INTERPRETER: *What are the handwashing practices here? How do you have to, what is the routine . . . obligated every hour?*

WOMAN: *To wash my hands?*

INTERPRETER: Mm-hmm.

WOMAN: *In my case, no . . .*

INTERPRETER: *Is there any special product?*

WOMAN: *We do have a special soap for us, yes.*

INTERPRETER: Yeah, she said that she washes her hands all the time. She doesn't have like a specific routine, but they have their own soap for like to clean their hands. ¿Usted también?

MAN: *Linda always puts some in the bathroom. Yeah, when we take off our gloves, and you want to wash your hands so that they are . . . gloves so that they are easy to put on. But, yeah, in the bathroom, there's always soap in the bathroom. Every farm always has their soap, but just that.*

INTERPRETER: *And disinfectant?*

MAN: *I think so.*

MAN: *. . . liquid.*

MAN: *Yeah, the soap isn't like, it's a different kind of soap. But, well, they bring it. I don't know what goes on with that.*

INTERPRETER: They have in the bathroom, like they said that they have to clean their hands every time they change their gloves. And they have a specific soap, they can do it whenever they want.

MODERATOR: Okay.

MAN: *There isn't a specific routine that says that you have to wash your hand after any certain task. But . . . logically, if my gloves are super dirty, then I take off my gloves, put new ones on, right. When you go to the bathroom, obviously, you have to wash your hands. But there's not like, anything established that says, when you do this, you have to wash your hands. But like I said, it's logical that if it is a cow, and it has . . . mastitis, and . . . take the tip off, then that glove is contaminated so I have to go wash my hands. And we don't have soap in the parlor, but the hoses that we have contain a special liquid for disinfecting.*

INTERPRETER: Yeah, they don't have a, like a specific routine of like how to wash their hands or anything, but like it's common sense. They have like, they have to go and change their gloves. And after they use the bathroom, it's the same thing. So it's not like a rule. It's most like common sense.

MODERATOR: Okay. That makes sense.

MAN: *With the calves too, there is a, there is disinfectant and soap. That disinfectant is used from the first to the fifth day of the calves. You have to be very careful when you feed them that the bacteria doesn't . . . because it's the . . . the calf, it's five days that the calf is, you have to know what to give it when it is bigger so that he doesn't grow up to be deformed and all that. Yeah, so you wash your hands each time you're going to feed them. You feed them. You wash your hands. You disinfect. And then you can feed the next one so that this one doesn't get sick, so it doesn't get what the other one has.*

INTERPRETER: *This is on the other farm?*

MAN: *It's here, well, no, here. I don't know how she does it. But, yeah, on other farms where I have fed calves, that's how it is.*

INTERPRETER: *Here too?*

WOMAN: *I don't know what farm he's talking about . . .*

INTERPRETER: Yeah, he said that in other farms, they have like a specific routine of washing hands when they are like managing, taking care of the cows, like from when they are born to the five first days. There are very specific rules like how to clean their hands. And when they feed the calves, and if they feed another one, they have to clean their hands between those. But like here, it's not.

MODERATOR: Are there certain tasks after which you typically wash your hands? Like specific tasks that they perform that they usually wash their hands for, after?

INTERPRETER: *Do you have any, is there specific moment or task that requires you to wash your hands afterwards?*

WOMAN: *In the morning, we always start the day, in the sink, on one side of the sink, we always have bleach water with soap. And that helps us wash our hands every once in a while. And we change gloves like two, three times per day or more. I like it always to be warm, and we go wash up every once in a while. It's, yeah, always . . .*

INTERPRETER: Yeah, she said that she works, like they wash their hands all the time. They have bleach in their bathroom and everything, so they go like and clean whenever they want.

MODERATOR: Whenever. Okay.

INTERPRETER: *And you?*

MAN: *It's like . . . common sense. But, no, there isn't a specific . . . your hands . . . infection. Right, but, yeah, when someone has . . . works on a farm . . . they tell you, but I didn't know what I had to do, so you just do it because it's common sense. There isn't . . .*

INTERPRETER: He said that here is not, they don't have a specific task where they say that you have to clean your hands. But they know, these people, it's like common sense. And they have experience on other farms, so they know that they have to clean their hands when like . . .

MODERATOR: Any other?

WOMAN: I mean, I usually do it after I scrape barns just because I get poop flying up at me all the time. So I try to do that as much as I can but nothing specific other than that.

MODERATOR: How do you get ready to leave work for the day?

INTERPRETER: *How do you get ready to go home for the day?*

WOMAN: *Me, at the end of the day?*

INTERPRETER: Uh-huh.

WOMAN: *I have to feed the calves and make sure they've all been fed. If they didn't want milk, I have to give them electrolytes. If they are sick, they I check their temperature, give them medicine, clean the pasteurizer, clean the . . . the milk with the special liquid for cleaning, clean the office, and I clean everything, then I'm done with work.*

INTERPRETER: *And you don't change your clothes or anything?*

WOMAN: *Oh, yes. Yeah, that . . . I have to take off all my clothing and all that. What's it called?*

INTERPRETER: *So do you leave your work clothes here?*

WOMAN:*Oh, no, I have a washing machine there. And, yeah, I leave it there to wash. All the clothing from work gets washed.*

INTERPRETER: She finish her work with the calves, and she has to get everything ready, clean everything. And then she has her own washing machine here. So she left her clothes there, and then she goes home.

MODERATOR: Okay.

MAN: *Well, we wash our own clothes here always. We change, well, those that come here from home, they are already ready. But, well, there are washing machines there. But, yeah, we always leave our work clothes here. We can't take home the clothes that is . . . and there they wash dirty clothes with work clothes. That's not okay. Perhaps some people do it, but it's not good. Yeah, everyone leaves their clothes here. Everything is taken off . . . they leave it in the washing machine here.*

INTERPRETER: *And you have a washer?*

MAN: *Yeah, here there is a washing machine, so, yeah ,we wash the dirty stuff.*

INTERPRETER: He said that they do the same thing. Like they leave their dirty clothes here in the washing machine. And they go like, the ones that leave here, that goes from here, so they come back they're ready to work. But they like leave their clothes here and wash here.

MODERATOR: Okay.

MAN: *In my situation, well, I come from the house. The same clothes for work . . . because in my case, I don't like to leave my stuff here because they use the same washer where they was the towels for . . . the cow. So I try to do different loads of clothing. And then I take it home and wash it there. I don't want to change my clothes here on the farm. It would just be the machines . . . the manure that I . . .*

INTERPRETER: He said that he prefers to, like to go home and wash his clothes there because here, the clothes get mixed with the towels they use for the cows. So he prefers to go home and change and wash his clothes there at home.

MODERATOR: Okay. How about you?

WOMAN: Well, usually when I leave, I have to feed the calves again a second time. So I do it once in the morning. And then I do it once, like right before I'm going to leave. And after that, I don't know, I don't have like a really long thing. It's just usually just go feed the cows and then come back, and I can go.

MODERATOR: Do you leave your laundry here?

WOMAN: No. No, I take mine home.

MODERATOR: Okay. So now we're going to switch focus a little bit and talk about antibiotics.

INTERPRETER: *We're going to switch now to talk about antibiotics and antimicrobials.*

MODERATOR: So you're probably familiar, but antibiotics are medicines doctors use to help a person who has an infection with bacteria, get better.

INTERPRETER: *Perhaps you're familiar with the antibiotics that are used as medicine to treat animals that are sick or people that have bacterial infection.*

MODERATOR: *Like in the nose.*

WOMAN: *Yeah.*

MODERATOR: And also in animals can be, which you've probably learned. What is your experience with taking antibiotics?

INTERPRETER: *The question is, what is your experience taking antibiotics?*

WOMAN: *Antibiotic . . .*

WOMAN: Like Tylenol and stuff like that, or just like, like what kind?

MODERATOR: Antibiotics are typically prescribed by the doctor. And so like amoxicillin is really common one.

INTERPRETER: *If like you've ever been prescribed antibiotics by a doctor?*

MODERATOR: When you have an infection or something.

WOMAN: I really don't know. I've never had antibiotics.

WOMAN: *Yeah . . . if you have a big infection or a, for example, I had a burn a little while ago, and it didn't want to heal. I had to go to the doctor, and he had to give me an antibiotic because the burn wasn't healing. Once I started taking the antibiotic, I felt that things got better.*

INTERPRETER: She has taken antibiotics especially like recently, she had a burn. She had a very bad burn. So she went to the doctor, and he proscribed her antibiotics. And she took them.

MODERATOR: Great.

MAN: *I think that the only antibiotics that I've taken is for kidney infection because . . . kidneys. After that, I can't take a lot of medication, I mean. . . . the majority of the medications. So . . . in my country, I have my doctor, and he knows what he can prescribe me. So it's possible that I might go to another place, and instead of curing me, they'll kill me. So . . . so but the only thing I self-medicate with is here for the kidneys because I know what I can take for that. If I have some kind of headache that I can't stand.*

INTERPRETER: He said he only use antibiotics when he has a kidney infection because he is allergic to most of them. So if someone tries to prescribe him antibiotics, they can kill him. So he's very like, he only like takes antibiotics when it's necessary.

MODERATOR: Okay.

MAN: *Well, I don't have, well, I don't know . . . I've never done any tests, but the one I've take is just for headache. Well, it's almost the only thing, but that's it.*

INTERPRETER: *But do you go to the doctor?*

MAN: *Well, here, in Nicaragua, it's just a few times. Not here. I've never been. So I don't know about taking many things. I really don't take anything. Maybe some vitamins, yeah, but medicines for diseases, no.*

INTERPRETER: Yeah, he hasn't taken any medications here, like when he has been sick. And back home he, sometimes for a specific disease, but like here, he haven't been.

MODERATOR: . . . how would you go about getting antibiotics if you needed them?

INTERPRETER: *How, if it were necessary to take an antibiotic, how would . . . if you needed an antibiotic, how would you obtain it, or how would you acquire it?*

WOMAN: My dad is a pharmacist, so I just have to ask usually. And he'll tell me if I can have it or if I can't.

INTERPRETER: *She says her dad is a pharmacist.*

MAN: *In my case, well, here in the United States, in general, if you don't have a prescription, they don't sell you anything. So here I've never bought anything that has to do with medicine. What I did was when I came here . . . Nicaragua, I brought a large quantity of medication which is what I normally take. It's possible that . . . via mail because here, I don't have anything to write a prescription for me to be able to buy it here.*

INTERPRETER: Yeah, he said that he doesn't have any to prescribe him antibiotics here. So he brought his own medication from Nicaragua. And he has like a stock of antibiotics. And when they exceed, when they finish, he said he has to ask them to, how do you say that? Like to send them.

MODERATOR: Oh, okay.

INTERPRETER: But here, he hasn't bought any antibiotics.

MODERATOR: Okay.

WOMAN: *In my case . . . go to the doctor, and he prescribed them for me.*

INTERPRETER: She asked, she went to the doctor, and they prescribed her for the infection . . . he hasn't taken any antibiotics here.

MODERATOR: Okay. So antibiotics sometimes stop working and are not able to kill or control the bacteria in a person or an animal.

INTERPRETER: *Sometimes antibiotics stop working against bacteria that they usually work against . . . they don't kill it.*

MODERATOR: So when this happens, the antibiotic isn't able to treat the person's or animal's infection, and it's called antibiotic resistance.

INTERPRETER: *Okay. So when that happens, it's possible that the antibiotic isn't working to treat the infection, and that is called antibiotic resistance. It's like when the bacteria isn't working for that antibiotic. I mean, it's not dying with the antibiotic.*

MAN: *Yeah, what happens is that a lot of bacteria they are, well, they are intelligent, right, medically intelligent. So when you take something which is very common, and you're using it all the time, then it becomes resistant to that medicine, and new forms develop that can survive in your body. So then you have to be checking your medication and switch medications.*

INTERPRETER: Yeah. He said that bacterias are very smart, and when you use, like frequently use an antibiotic there will be like resistance and like, yeah, he knows.

MODERATOR: . . .

INTERPRETER: *It is what she is . . .*

MODERATOR: Okay. Any other thoughts about antibiotic resistance, what have other people heard or what do you know about it?

INTERPRETER: *Do you know anything about antibiotic resistance or, what do you think about antibiotic resistance?*

MAN: *Well, it's not just with antibiotics, but with a lot of medications. The disease becomes resistant, and, yeah, well, then it becomes a case where the bacteria, I have to fight it with an antibiotic, well, yeah, they're more resistant to many medications, right. And I know that, for that reason . . . to research what we can inject. They can, they have the ability to avoid it and, well . . . survive and reproduce.*

INTERPRETER: Yeah, he said that like bacterias has the power to resist antibiotics if you use it frequently and change. And like the same thing happens with other medications. ¿Usted tiene algún sabida de resistencia de esta, o?

WOMAN: Nada.

INTERPRETER: ¿No? She didn't know like about this antibiotic resistance.

MODERATOR: Any other thoughts?

WOMAN: The only thing that I really know about this is just like a person thing is that, I mean, I found out like I didn't know that if you took like let's say Tylenol every day for like three months that you could become resistant to it. But I found that out.

[Simultaneous discussion]

INTERPRETER: . . .

MAN: *Well, sometimes the medications are good. But like he said, all the bacteria and diseases grow, and they keep getting bigger. For that reason, you need to have better, stronger medicine that can avoid those illnesses that you have. So you have, you have to look at a lot of . . . get more stronger things to avoid those things now because they are always going to resist. That's it.*

INTERPRETER: Yeah, he thinks like the same thing. Like that the bacterias get resistant, and we need to find a way like other medications, stronger medications to try to kill these bacteria that get stronger and stronger.

MODERATOR: What kind of risk do you think antibiotic-resistant infections pose to you or others in the community?

INTERPRETER: *What risks do you think that antibiotic resistance poses to you or the people in your community or family? I mean, what risk does the existence of antibiotic resistance pose to you?*

MAN: *Well, I think that one of the risks that can exist is that the disease . . . chronic. So when you don't have a positive response to the antibiotic that you're taking, then, well, you can . . . chronic illness. In, it's difficult, more difficult to treat. So because it gets complicated because you don't know . . . the part that was being damaged by the bacteria, or if it is damaging another organ in your body. It just gets complicated.*

INTERPRETER: He said that the diseases become chronic, and it's more difficult to treat them if they're more resistant to antibiotics, and it gets worse and worse.

MODERATOR: Okay. Any other thoughts? Okay.

MAN: *There are also bacteria that exists that can be passed, right, not just contained to one person. Sometimes, well, it's not just one person, it keeps reproducing to many people. And so it gets bigger and bigger.*

INTERPRETER: He said that . . . that diseases become like more contagious because of the antibiotic resistance . . . and you're sick with infection, and you like transmit it to another person . . .

MODERATOR: What role, if any, do you think dairy farms play in antibiotic resistance?

INTERPRETER: *What do you think the role that dairy farms play in antibiotic resistance is, if you think that they have anything to do with dairy farms?*

MAN: *If you could restate the question.*

INTERPRETER: *Yeah, like what part do you think dairy farms have in the antibiotic resistance, if you think that they play a part in it?*

MAN: *It has a lot to do with it because, well, they're not the same as ours, the same medicines. But bacteria grows fast in the, what are they called again, teats. Mastitis grows there. And if you can't cure it quickly, well, you can't cure it with medication because there are times when it doesn't make it, like you said. So it's in the teat, and it goes without that teat. Then there are just three left . . . so you can treat. So, yeah, you have to, there are times when you can cure it, and times you can't. Yeah, because it's a really strong disease, or it's too late to give her . . .*

INTERPRETER: *So it's more or less because here on the dairy farm, you are dealing with bacterias and . . .*

MAN: *Yeah, yes. Bacteria.*

INTERPRETER: *Bacterial infections and things like that.*

MAN: *Yeah, you have to be dealing with it. But for that you have to be cleaning almost all the time so that the bacteria doesn't grow and cause an infection in the cow's teat.*

INTERPRETER: Yeah. He thinks there is very related with milking farms because they usually, cows have a lot of bacteria and like, for example, the mastitis. And they're dealing a lot with bacterias and infections, bacterial infections in cows.

MODERATOR: Okay.

MAN: *If I understood the question, well, if the farm is using antibiotics on the animals, well, of course, that it is going to have an impact at the social level. If you don't fight some bacteria in the cows, then the milk can hurt us if we consume it. At the same time, the bacteria is also inside the meat of the animal, if it's going for meat. That is also another risk, right. And another thing that is interesting is I don't know how it works here, in Nicaragua, well, here in the United States, there is a company for, which is a product with colostrum from cows, right. And there are also a lot of products that are for fighting infections. So I also think that on the farm, they should be providing the colostrum of the cow so that they can . . . medicine . . .*

INTERPRETER: Okay. Yeah, he said that he thinks that also they play a role because well, for example, if a cow has a bacteria in the milk, and it goes to human consumption or like the meat of the cow, it can pass bacteria too. And it goes directly to the consumption of humans. And like the same with colostrum. Do you know what colostrum is, like the first milk of the cow?

MODERATOR: Yeah.

INTERPRETER: So, yeah, that's why he thinks that these are all . . .

MODERATOR: Okay. Anyone else?

WOMAN: I was definitely thinking the same thing as what he just said. But like I was talking to the owner yesterday, and we just kind of brought up like the fact that with the antibiotics and like the cow, and then you take it to like, goes on a meat truck. But they also have like two different places it goes. It can go to the one that is just like for, let's say dog food and stuff like that.

And then you've got to factor in, okay, oh, the antibiotics going in there. What is the like the product? Like you were saying, goes to many different things. And it's like, let's say it goes into your McDonald's hamburger, like whatever. But it's still, like it's getting spread to different things. But then like answering a question with a question it's, how do they know then to get all that bacteria out before it goes to like humans? And like I just want to, I would want to know how they can like test that to make sure that, yes, this cow meat is like 100% free of bacteria.

But then are they okay with doing it for humans, but then you put them in dog food or some kind of other source of whatever they use other cow meat for. But then, do they take like let's say only 70% of that bacteria out because that bacteria could also help and factor in like the other, dog

food. Let's say it actually like helps increase some nutrient from something else, I don't know. Just things I wonder, I guess.

MODERATOR: And then kind of the reverse, how do you think farms like this one are impacted by antibiotic resistance?

INTERPRETER: *And like the reverse, how do you think that farms like this are affected by antibiotic resistance.*

MAN: *In the case of farms, like the one we work for, it is affected with the part, for example, when a cow has mastitis, an infection, so if it's not treated on time or the mastitis is too advanced, then it's impossible to reduce the . . . with the medicine. So logically, that cow is going to die. And last month, we had ten cows die. So that's a loss for the farm that can't be recouped. But that's with the cows. And for the calves, well, her partner that treats the calves too . . . infection that also . . . dead calves. So, yeah, there is a direct effect that the antibiotics, not the antibiotics, but that the cow isn't treated in time.*

INTERPRETER: *For example, that they resist the antibiotics, that the bacteria isn't working, I mean, that it is resisting the antibiotics?*

MAN: *The problem is that, personally, I don't know what kind of antibiotic they use on the farm to fight . . . cows. I can't say if it's the right one or say that it's not . . . separate the animal. I mean, I can't give my opinion on it. But could it be that the bacteria is becoming resistant to the antibiotic that they're administering, or that they're not administering it at the right time. So, yeah, it affects it because the cow is going to die.*

INTERPRETER: He said that it impacts directly because the cow can die because of a disease. Like the disease are getting worse, and then the cow eventually can die. They had like ten cows that died last month. And the same thing he thinks about the calves because like if they are resistant, then the calf is getting sicker and sicker and can die. So he feels . . .

MODERATOR: Okay. Anyone else?

WOMAN: I think it's also affected too because we do get cows from two different places. And we like transport them from there to here to just different places. I think that like you have to factor in the bacteria that, it's not exactly the same in that, all three places. So then you're taking one, going to like another, but they're all in the same trailer.

So then you think, in that trailer, it's just one big, giant like bacteria whatever. And then it's like they go through them all day, all the time. So it just, I mean, I think it, especially big farms like this when you have like, let's say 950 cows, you're going to get that because there's like transports. And like how they were saying with the calves and all that, you get a lot of that from coming from like even the outside back in here.

MODERATOR: What are the current rules on your farm about antibiotic use?

INTERPRETER: *What are the rules that you have here on the farm regarding the use of antibiotics when, if you have can give them, how much, and all that, what are the rules? You?*

WOMAN: *In my case, yeah, I know when to apply the antibiotics to a calf after I've treated it for fever, whether or not it's dehydrated. I've treated it, and the calf is still sick. Obviously, I have to inject the antibiotics because I know that it's strong bacteria, and that it needs an antibiotic.*

INTERPRETER: She said that she knows when she used antibiotics that in case of fever or when the calves are getting worse, she uses antibiotics for treatment. With . . .

MAN: *On the farm, I think that the place where antibiotics are used the most is with the calves because here we are . . . big, the one who takes care of that is Linda, the owner. At least the milkers and the barn workers cannot administer any kind of antibiotic. We don't handle that . . . that . . . we don't do that. They take care of it . . . I think that it's mostly . . .*

INTERPRETER: *With the calves.*

MAN: *With the calves. And including . . . we have . . . when they're born, we can't give them antibiotics because that is the job of the person who is in charge of the calves.*

INTERPRETER: They don't have to apply any antibiotics for cows. If a cow is sick, the owner can get these, she's in charge of the use of antibiotics. So they don't use any antibiotics. And he thinks that it's mostly on the calves.

MODERATOR: Okay.

INTERPRETER: Yeah.

MAN: *Well, I don't know. Before, yes, we did administer them here once a day to the ones who had mastitis. So, yes, we did apply them once a day in the morning and after the shift ended . . . now that we all have shifts in the hospital, which is where the sick ones are, the ones who have illnesses, yeah, we used to put a shot in each teat. I forgot the name of it. But, yeah, not anymore. Now they do it after the shift is over.*

MAN: *And Linda does it.*

MAN: *Yes.*

INTERPRETER: Okay. He said that before, he had to apply antibiotics. Like the last time that he worked here, they had to apply antibiotics once a day, like only on the sick cows. They call it like the hospital for the cows. But now, like the owner is the only one that applies antibiotics.

MODERATOR: Who does it for the calves? Does she do it, or just?

INTERPRETER: *Do you give the calves antibiotics?*

WOMAN: *Yes.*

MODERATOR: Okay. Did you have anything you wanted to . . .

WOMAN: I mean, the only thing that I really do is stuff with pneumonia for when they go into like the wean barns. So right after they come from where she has them, they go there. And then we just do like the follow-ups on pneumonia and stuff like that but nothing else.

MODERATOR: Okay. Do you apply the antibiotics, or?

WOMAN: I haven't yet.

MODERATOR: Oh, okay. But you . . .

WOMAN: Yes, I will be. Yeah. But not yet.

MODERATOR: Maybe ask him if he knows why they changed the, it switched from him applying the antibiotics to the owner?

INTERPRETER: *She's asking if you guys know why they changed that rule that before you could give antibiotics, and now it's just the owner.*

MAN: *No, truthfully, the owner doesn't do if often. The guy who has been here the longest also does it. Yeah, because she can't sometimes. Or there are times when she has other things to do. So she puts him in charge of that job. Yeah. But she does it too. I don't know why. But, yeah, almost all of the bosses when they don't have anything to do, do it. They do it. Yeah, I don't know. Well . . . I don't know. It's different. Yeah, it's very different.*

INTERPRETER: He doesn't know really like why they changed it because he just got here. But like he said, that now, if it's not the owner that applies the antibiotics, it's like the most experienced worker that applies the antibiotics. He doesn't really know why he changed it.

MODERATOR: Does anyone know if that was a while ago that they changed it?

INTERPRETER: *If you know how long ago they changed that rule about the owner doing it.*

MAN:*What I understand is that before, there was a manager here on the farm. I mean, from what I understand, the manager always delegated the responsibilities for whoever was going to be giving medicine. I don't know if since they got rid of the manager, they lost their routine, I don't know. But . . . but when I got back here, it was like this.*

On other farms, like he said, on other farms, they allow the milkers to administer medicine including if . . . I see that a cow has a problem, and she doesn't want to let her milk down, give her an injection so that her milk lets down. But not here . . . or it could be that because there isn't a manager or because we're just used to the owner doing it. I don't know what the right answer is.

INTERPRETER: He thinks that maybe it's because they used to have a manager that like delegated these things. But now, like they don't have a manager, so the owner is in charge of that. Because on other farms, they usually do this. They apply the antibiotics.

MODERATOR: Okay. Thank you. Okay. And then the last thing I'd like to ask you about is raw milk. How are raw milk and raw milk products handled on the farm?

INTERPRETER: *Yes, the last question that she wants to ask is about the raw milk. How is the raw milk handled here on the farm? What do you do with the raw milk?*

MAN: *When you say raw, what do you mean?*

INTERPRETER: *Yeah, like unpasteurized. Like the milk that just came out of the cow.*

MAN: *It has to . . .*

MAN: *Well, here, the milk just goes from the machines into the tank.*

INTERPRETER: *The tank.*

MAN: *And I don't know what they do with that raw milk.*

INTERPRETER: *They sell it.*

MAN: *Yeah, I don't know what they do with it.*

INTERPRETER: Yeah, they said that like the milk goes directly to the tank, so they don't really know what happens after that like so.

MODERATOR: Do they ever use raw milk, even if it's not from this farm or anything?

INTERPRETER: *She's asking if you guys have ever used raw milk at home or drank it, even if it's not from this farm, but from another farm?*

MAN: *No, well, there isn't, well, it's not good. The raw milk here has a lot of, what's it called? I forgot.*

MAN: *Bacteria?*

WOMAN: *Here on this farm . . .*

MAN: *Cells.*

WOMAN: *Here on this farm, we don't handle the raw milk. No employee has access to it. The milk, at least, the milkers don't have any contact with the milk. For the calves, we do, we pasteurize it, but it's just for them. Not for human consumption on this farm though.*

INTERPRETER: She said like they don't have any contact with raw milk here. They use it for the cows, but they pasteurize the milk that they give. But they don't drink any, they don't have any contact with like, and he thinks that it's bad to drink because it has a lot of cells.

MODERATOR: Okay.

INTERPRETER: *So they don't, you don't, you guys don't drink raw milk?*

MAN: *No, the raw milk has too many cells in it. Yeah, so . . . cells and all that is not good for your body. It has to be cooked or something like that, pasteurized. So if you drink it, it can cause some damage to our stomach because, well, I don't know, it's just dirty.*

INTERPRETER: Yeah, he said that it's bad to drink. It's dirty, and it has a lot of cells, and it can be bad for you, so they don't drink it?

MODERATOR: Does everybody feel the same way?

INTERPRETER: *Do you all feel the same way?*

MAN: *No, well, like he said, it's because of the way that the livestock is treated here. So the milk has a lot of cells. I don't know actually how damaging consuming unpasteurized milk is. I have no*

idea. But also if you gave me a glass of milk from here, I wouldn't drink it, from any farm. I wouldn't drink a glass of milk from any farm. It's different in our countries where the cow is out in the fresh air, and it's not dealing with . . . mastitis because it's difficult. But here the way that livestock is treated is different so . . .

INTERPRETER: Yeah, he thinks that he wouldn't drink raw milk from here, like from the ranch because of how it's treated, and he's aware of all the diseases and everything. So he wouldn't drink. Maybe in his country because they are like pasture-raised kind of so, yeah, but here, no.

MODERATOR: How about you, do you have any thoughts on raw milk?

WOMAN: Well, I mean, I don't really handle it here at all. But the farm I used to work at, we drank it a couple times. And I'm not, it's not good. Like it's definitely not the greatest.

[Simultaneous discussion]

INTERPRETER: [Talking in background]

WOMAN: *. . . since we work here we don't have, what's the word, I don't know . . . because we see everything. We are at work. And aside from that, we know that the cows get a lot of medicine, the food, and all that, best not to drink it.*

INTERPRETER: Yeah. She thinks that it's because they are like with the cows, and they know how the cows are treated. And they use a lot of medication, so they feel like it's not safe.

WOMAN: *Perhaps it's not like dirty or that it's worthless, no, but, yeah, it needs to be pasteurized to be more hygienic.*

INTERPRETER: Yeah, she said that it's much more hygienic to drink pasteurized milk.

MODERATOR: Well, that's all the questions we have. Any other thoughts before we end that you think we should know about the farm or antibiotics?

INTERPRETER: *Those are all the questions that she has. But she wants to know if you have any other comments that you want to make or any questions about what we talked about.*

WOMAN: *What did you think of our answers?*

INTERPRETER: She wants to know what did you think about their answers?

MODERATOR: It was very helpful.

INTERPRETER: *She says that it was very helpful. You guys were very helpful. This is going to be a big help for the study.*

MODERATOR: It was really nice to hear what everyone thinks about this because we didn't really know what to expect coming in. So it was very helpful.

INTERPRETER: *She said that it was very helpful because she didn't know anything about the handling. So it's been very informative. And you guys are helping them a lot for their study.*

MAN: *I would like to know, what impact will this investigation have on the betterment of, or if the purpose is betterment, or if the purpose is to improve some kind of medicine or leave some kind of declaration for others(?)*

INTERPRETER: He wants to know like what is like the ultimate objective of the research like if, or the impact, if you're trying to develop a new medication or if you're trying to improve like practices.

MODERATOR: We are trying to improve practices. We might come back to the farms in a year or two and see if there is, not necessarily this farm, but other farms, and see if there are things they're doing that we learned from this groups should be changed. If you want to translate it, go ahead.

INTERPRETER: *What they are going to do is try to improve the practices that are happening, I mean, not just on this farm, but perhaps on others. Because this will be to improve, what's the word, or to combat antibiotic resistance. What they can do to improve the farm. So later, in like a year, year and a half, they're going to come back. When they have all the results, they are going to come to the farm and inform everyone.*

MODERATOR: Our main goal is to make sure that all the workers are safe and just the practices, we're trying to understand the practices and just end goals to make farm work safer for everyone.

INTERPRETER: *Their objective is to succeed in having safer farms for the workers and animals. And also to make sure that . . . infection and all of that. That is their objective. Like . . . to get better.*

WOMAN: *That is the question I was going to ask. Is the objective our health as employees or the health of the animals?*

INTERPRETER: Yeah, she wants to know if your goal is like the health of the workers or like the animals?

MODERATOR: Both, both.

INTERPRETER: *Both she said. That it will be beneficial to both humans and animals.*

MODERATOR: I think everybody should work, like everybody should work at a farm at least once in their life. I mean, not even for a long time. Like give it three days, just so they can understand what actually goes on. Because I know people still think that frickin' chocolate milk comes from brown cows. I know that there's people out there that, they just don't know what, like they think they just like take their milk off the shelf at a store, and that's like where it comes from. Like I just wish people would actually like be able to get up close and actually know like . . .

INTERPRETER: Experience it.

WOMAN: Yeah. And at least like go through and just like understand things about it because some people are just like idiots.

INTERPRETER: *She says that she thinks that people should have the opportunity to work on a farm, even if it's just for a few days, so that they can know what it's all about and everything.*

MODERATOR: Thank you.

MODERATOR: I have compensation.

[Simultaneous discussion]

INTERPRETER: *I don't know if you have any questions.*

MAN: No.

MODERATOR: ¿Está bien?
TRANSLATION: *Okay.*

Focus Group 2, Farm 2
Recording in English and Spanish

MODERATOR: . . . si quieren, la idea es que si puedan silenciar los celulares. Pero si tienen que tomar una llamada, por ejemplo, pueden hacerlo libremente o si tiene que hacer un break para ir al baño también, ¿okay?
TRANSLATION: *. . . if you want, the idea is to silence your phones. But if you have to take a phone call, for example, you can do that freely. Or if you have to go to the bathroom, that's fine too. Okay?*

MAN: Okay.

MODERATOR: Y bueno, y lo primero es saber un poco de ustedes y de qué funciones cumplen acá en esta granja. ¿Qué tipo de actividades hacen día a día? Entonces a ver si no le molesta mover ahí como por el circulo para que cada uno diga un poco de cual es su función acá, qué hace, a qué se dedica en a la granja, cuánto tiempo lleva trabajando aquí, y si tiene alguna experiencia en otra graja. Vamos a empezar por acá.
TRANSLATION: *And okay, and the first thing is to find out a little bit about all and what roles you fill here on this farm. What kinds of activities do you to day-to-day? So let's see if I can't bother you to move into a circle so that everyone can say a little bit about what their role is here, what they do, and what is your job on this farm, how long you have been working here, and if you have any experience on other farms. We're going to start here.*

MAN: Yo me dedico a ordeñar . . . becerros . . . llevo año y medio trabajando aquí. Y no tengo experiencia en otra granja.
TRANSLATION: *My job is milking . . . calves . . . I've been working here a year and a half. And I don't have any experience on another farm.*

MODERATOR: Okay bien.
TRANSLATION: *Okay. Good.*

MAN: ¿Yo?
TRANSLATION: *Me?*

MODERATOR: Mm-hmm.

MAN: Me dedico a ordeñar.
TRANSLATION: *My job is milking.*

MODERATOR: ¿Ordeñar?
TRANSLATION: *Milking?*

MAN: Sí. Y no más que aquí llevo cuatro meses . . .
TRANSLATION: *Yeah. I've only been here four months . . .*

MODERATOR: Cuatro meses.
TRANSLATION: *Four months?*

MAN: . . .

MAN: Yo atiendo los becerros. Llevo apenas un mes. Soy nuevo aprendiendo y no he tenido ninguna experiencia en . . .
TRANSLATION: *I take care of the calves. I've only been here a month. I'm new, and I'm learning, and I've not had any experience at . . .*

MODERATOR: Okay.

MAN: Yo tengo un año y medio trabajando. Me dedico a ordeñar algunos turnos y otros turnos hacer limpieza. Llevo, en esto un año y medio. Y yo ya había trabajado en otra granja también ordeñando nada más como dos años atrás. Pero en este . . . lo que es, manejo pues afuera el tractor que echar arena hago un día, y a la vez echarle comida.
TRANSLATION: *I've been working here a year and a half. My job is milking for some shifts, and on other shifts, I clean. I've been here a year and a half. And I have worked on other farms as well, milking, like two or three years ago. But on this farm . . . I'm outside, in charge of spreading sand with the tractor one day, and then feeding.*

MAN: La comida.
TRANSLATION: *The food.*

MODERATOR: La comida.
TRANSLATION: *The food.*

MAN: Sí, a dar de comer a las vacas. Pero eso es, no tan seguido. Es cada otro día.
TRANSLATION: *Yes, to feed the cows. But that it's not as frequent. It's every other day.*

MODERATOR: Okay. Muy Bien.
TRANSLATION: *Okay. Very good.*

WOMAN: Yo tengo cuatro años. Ordeño también, y también separo vacas, y un tiempo trabajaba también con los becerros.
TRANSLATION: *I've been here four years. I milk as well. And I also separate cows, and for a while, I was working with the calves too.*

MODERATOR: ¿Y usted había trabajado en otro lado?
TRANSLATION: *And have you worked somewhere else?*

WOMAN: Sí. En otra granja, sí.
TRANSLATION: *Yeah. On another farm, yeah.*

MODERATOR: Sí. ¿En lo mismo, con vacas?
TRANSLATION: *Yeah. Doing the same thing with the cows?*

WOMAN: Sí, ordeño nada más.
TRANSLATION: *Yeah, just milking.*

MODERATOR: Okay.

MAN: También inyecta vacas, ella.
TRANSLATION: *She also injects cows.*

WOMAN: También inyecto . . .
TRANSLATION: *I also inject . . .*

MODERATOR: También inyecta vacas. Okay.
TRANSLATION: *You also inject cows. Okay.*

MAN: Pues, yo soy ordeñador. Trabajo también en limpieza, insemino vacas, inyecto vacas, cuido los becerros. Y tengo cuatro años trabando por este rancho. Y no he tenido experiencia en otra granja.
TRANSLATION: *Well, I'm a milker. I also do cleaning, inseminate cows, inject cows, take care of calves. I've been working here at this farm for four years. I've never had any experience on another farm.*

MAN: Igual, como cuatro años en este rancho. Lo que hago es usar mucho antibiótico. Entre él y yo, usamos mucho antibiótico aquí desde la cuidar mastitis, cualquier enfermedad, con las frescas, igual a inseminar . . . ¿qué más? Y sí, he trabajado en otro rancho.
TRANSLATION: *Same here, like four years here on this farm. What I do is use a lot of antibiotics. Between me and him, we use a lot of antibiotics here for curing mastitis, whatever illness, with fresh cows, also insemination . . . what else? And yeah, I've worked on another farm.*

MODERATOR: También.
TRANSLATION: *As well.*

MAN: En 13 años, he trabajado en tres ranchos.
TRANSLATION: *In 13 years, I've worked on three farms.*

MODERATOR: ¿Y siempre con vaca lechera?
TRANSLATION: *And always with dairy cows?*

MAN: Sí.
TRANSLATION: *Yes.*

MODERATOR: Okay.

MAN: Okay. Yo me dedico nada más echar comida a las vacas el lunes a sábado una semana, y de lunes a viernes otra semana. Tengo dos años aquí. He trabajado en tres granjas en 18 años. Este es mi tercera granja que trabajo. Las otras granjas, trabajaba de herdsman, y ahora estoy echando comida. Bueno, no, primera granja, empecé ordeñando, y luego me pasaron a herdsman. Y la segunda, entré como herdsman. Y aquí como feeder.
TRANSLATION: *Okay. My job is just to feed the cows from Monday to Saturday one week, and then from Monday to Friday the next week. I've been here for two years. I have worked on 3 farms in 18 years. This is the third farm I've worked for. On the other farms, I was a herdsman, and now feed. Well, no, on the first farm, I started milking, and then they moved me to herdsman. And on the second one, I started as a herdsman. And here, I'm a feeder.*

MODERATOR: Muy bien. Entonces, bueno, a continuación, me gustaría también que nos contaran más o menos cuál es un turno típico, un típico acá en sus labores. ¿Qué es que normalmente hace en un día en su turno?
TRANSLATION: *Very good. So, okay, to continue, I would also like you all to tell us more or less what a typical shift is like here for your jobs. What do you normally do in a day during your shift?*

MAN: ¿Qué es lo que hacemos?
TRANSLATION: *What do we do?*

MODERATOR: Sí. Sí, como un turno, ¿qué hace? ¿Cómo empieza, así?
TRANSLATION: *Yeah. Yeah, like on a shift, what do you do? How does it start? Like that.*

MAN: Que actividades hace aquí, cuál es su función.
TRANSLATION: *What activities you do, and what is your role.*

MODERATOR: Claro, en un día típico acá trabajando.
TRANSLATION: *Yes, during a typical day working here.*

MAN: Bueno nosotros que estamos en la mañana siempre hay más cosas que hacer, siempre. Siempre llegando lo que es meter toda la información a la computadora de vacas frescas. Luego revisaran el cow mánager . . . programa . . . las que están listas para inseminar y las que están enfermas, e inmediatamente ir al corral a checar las temperaturas y todo eso. Después inseminar, si toca separar y inyectar todo . . . la mañana es cuando hay más trabajo como más movimiento, más personas trabajando. En la tarde, pues . . . en la noche. Pero en la tarde, digo en la mañana es más personas.
TRANSLATION: *Well, those of us who are here in the morning, there are always more things to do, always. When you get here, you put all the information into the computer about the fresh cows. Then you look at the cow manager . . . program . . . the ones that are ready for insemination and the ones that are sick, and then immediately go to the corral to check temperatures and all that. After that, inseminate, if you need to separate and inject all . . . the morning is when there is more work, like more movement, more people working. In the afternoon, well . . . in the night. But in the afternoon, I mean, in the morning, there are more people.*

MODERATOR: ¿Usted se encarga de todo esa parte?
TRANSLATION: *And you are in charge of all of that?*

MAN: Sí.
TRANSLATION: *Yes.*

MODERATOR: Okay. ¿Alguien más quiere contar como es su día?
TRANSLATION: *Okay. Does anyone else want to talk about how their day goes?*

WOMAN: Yo en la mañana pues ordeño. Y ya terminando de ordeñar, voy a lavar los tanques, y luego saco la basura, y ya.
TRANSLATION: *Well, in the morning, I milk. And after milking, I go wash the tanks, and then I take out the trash, and that's it.*

MAN: Mi turno es variado porque le digo hago diferentes cosas diario. Pero normalmente, cuando tengo que echar arena, pues, tengo que llegar y sacar los scrapers y pararlos para, dependiendo del lado que voy a echar. Ya tengo que parar, lo primero que hacer es pararlos y ya de allí mover las vacas hacia el lado contrario para poder entrar un tractor para echar arena de un lado, y después ir con otro. Así me la llevo todo el turno para terminar . . .
TRANSLATION: *My shift varies because, like I said, I do different things during the day. But normally, when I have to put down sand, I get there and turn off the scrapers depending on the side I'm going to put down. So I have to stop, the first thing I have to do is stop them, and then from there, move the cows to the other side so that I can get in with the tractor to put down sand on one side, then do the other side. That's how I spend the whole shift to finish . . .*

MAN: Para el calf management, hay todo un protocolo que estoy aprendiendo, bueno, ya lo tengo más dominado, pero son muchas actividades en el día. Hay que cuidar mucha la limpieza. Lo primero que se hace es alimentar los becerros que están en las pequeñas jaulas que tienen apenas de uno a diez días de nacido. Ellos se alimentan con . . . entonces hay que esterilizar a los . . . con químicos. Hay que darles el alimento directamente porque, o entrenarlos porque no saben beber.

Entonces después de eso, hay que lavar todo, todo el material que se utiliza. Hay que volver a, hay que lavar los tanques, hay que lavar, esterilizar el . . . al que . . . los becerros que estén bien de temperatura, cómo es su manure, si están haciendo bien o hay que suministrar el medicamento también. Hay que revisar las vacas que están enfermas y separadas, o los becerros. Y hay que volver a lavar. Se lava como unos tres o cuatros veces el área para que esté siempre limpio.
TRANSLATION: *For calf management, there is a whole protocol that I'm learning, well, I've got it more or less mastered, but there are a lot of activities during the day. You have to take care of a lot of cleaning. The first thing that you have to do is feed the calves that are in the small huts that are between one and ten days old. We feed them with . . . so you have to sterilize the . . . with chemicals. You have to feed them directly because, or train them because they don't know how to drink.*

So after that, you have to wash everything, all the material that you used. You have to return to, you have to wash the tanks, you have to wash, sterilize the . . . to the . . . the calves that have a good temperature, how is their manure looking, if they are going okay, or if we need to give them some medicine too. We have to look at the sick cows or the separate ones or the calves. And then you have to wash again. You wash the area like three or four times so that it is always clean.

MAN: ¿Qué hago? Pues queda uno a ordeñar. Terminando turnos que . . . vacas, hacer la limpieza también de lavar todas las . . .
TRANSLATION: *What do I do? Well, you stay and milk. After finishing a shift . . . cows, do some cleaning, and also clean all the . . .*

MAN: Pues ahorita llegando hay que, primero pues hay que cambiarse. Pero después hay que ir por las vacas . . . la maquina . . . líquido para, porque los primeros grupos se limpia con toalla . . . líquido. Uno se queda afuera a limpiar y ve a pushar las vacas. Ya después cada quien va por el grupo uno y uno . . .
TRANSLATION: *Well, right now, when you get there, the first thing you do is change your clothes. But after that, you have to go get the cows . . . the machine . . . liquid to, because the first few groups are cleaned with a towel . . . liquid. Then you're outside cleaning, and you go push the cows. And then after that, each person goes to get a group one by one.*

MAN: Lo primero que hago es llegar a . . . temporada, precalentar el tractor, monta carga. Y luego checar cuánta comida tiene cada grupo de vacas. Y luego empiezo a bajar comida de bunker, hacer filas para ya no más estar cargando el tractor. Y luego ya limpiar la comida que dejan las vacas de anterior. Entonces ya después el patrón me manda un mensaje de cuántas libras necesita por cada grupo. Y ya me dedico nada más a preparar las y repartir en los corrales. Tome aproximadamente seis horas. Y luego ya después, dejo todo preparado para el día siguiente, quitar llantas, plásticos, está todo limpia donde se prepara comida y todo eso.
TRANSLATION: *The first thing I do is get there to . . . season, warm up the tractor, forklift, and then check to see how much food each group of cows has. And then I start getting feed out of the bunker, make lines so that you're just driving the tractor. And then clean up all the feed the cows left from last time. So then after that, the boss sends me a message with how many pounds of feed each group needs. And then my job is just to prepare it and feed it out in the corrals. It takes approximately six hours. And then after that, I get everything ready for the next day, change tires, plastic, and everything is all clean where the food is prepared and all of that.*

MODERATOR: Nos falta uno más.
TRANSLATION: *We still have one to go.*

MAN: Yo pues soy igual. Mi trabajo es variado. Ahora ya me estoy dedicando más a ordeñar, pues la misma rutina que todos mis compañeros. Igual y hay un corral que está, que se tiene que vigilar cada hora donde están las vacas de preñadas porque hay unos corralitos pequeños donde se tiene que mover la vaca preñada para que tenga su cría en un lugar limpio. Entonces, se está vigilando cada hora. Y ahora si ya está una vaca allí con el becerrito, moverlo, calentar la leche, mover la vaca para la ordeña, y darle de comer al becerrito.

Tienen que pasar como 30 minutos para que coma su primer alimento, darle también un antibiótico . . . para que . . . la ordeña pues ir por las vacas, ordeñar, rastrillar allí en las camas de las vacas, los pasillos . . . lo que se hace en ocho horas. Siete horas de ordeña y una hora de limpieza, lavar las paredes con presión o jabón las pones. Es todo lo que estoy haciendo en un turno.
TRANSLATION: *Well, I do the same. My job varies. Right now, my job is milking, but I have the same routine as all my coworkers. There is a corral that is, that has to be monitored every hour where the pregnant cows are because there are some small corrals where the pregnant cows have to be moved to so they can have their calves in a clean place. So they are watched every hour. And then if there is a cow there with a calf, you have to move it, heat up the milk, move the cow to be milked, and feed the calf.*

You have to wait 30 minutes before the calf can have their first feeding, you give them an antibiotic too . . . so that . . . the milking, then go get the cows, milk, rake the cow bedding, the walkways . . . that's everything that is done in eight hours. Seven hours of milking and one hour of cleaning, washing the walls with a power washer or with soap. That's everything that I do in a shift.

MODERATOR: Y por ejemplo, si tienen alguna duda con lo que hacen en su trabajo, ¿a quién tiene, a quién le pregunta?
TRANSLATION: *And for example, if you have any questions about what to do in your job, who do you have, who do you ask?*

MAN: Dependiendo del caso. Pues en caso de una vaca se habla . . . el señor. Y así y es dependiendo del problema. Si es de maquinaria o de los sistemas, pues ya le hablamos al patrón. O los tractores igual, si tenemos problema con un tractor, se le habla directamente al patrón porque él sabe de sus maquinas y eso.
TRANSLATION: *It depends on the situation. Well, in the case of a cow, you talk to . . . the man. And it's like that, it's dependent on the problem. If it's about machinery or about systems, well, then we talk to the boss. Or if it's about tractors, same thing. If we have a problem with a tractor, we talk directly to the boss because he knows his machines and all of that.*

MODERATOR: Y ¿el patrón es el manager, o es el dueño?
TRANSLATION: *Is the boss the manager, or is he the owner?*

MAN: El dueño.
TRANSLATION: *The owner.*

MAN: Él es el dueño.
TRANSLATION: *He is the owner.*

MODERATOR: ¿El dueño?
TRANSLATION: *The owner?*

MAN: Sí.
TRANSLATION: *Yes.*

MODERATOR: Okay.

MAN: Pero es dependiendo de la situación.
TRANSLATION: *But it depends on the situation.*

MODERATOR: Todos hacen eso, si tienen una duda o primera . . . sí
TRANSLATION: *And you all do that if you have a doubt or first you . . . yeah.*

MAN: Sí, si es como algo que se pueda resolver más localmente, pues vamos con algunos de nuestros compañeros. Pero si es algún problema más grande o de alguna enfermedad en específico, se le pregunta al mánager.
TRANSLATION: *Yeah. If it is something that can be resolved locally, well, then we talk to our coworkers. But if it's a bigger problem or about a specific illness, then we talk to the manager.*

MODERATOR: Y normalmente, ¿cuándo tienen que hablar con el supervisor aparte de que si tienen un problema?
TRANSLATION: *And normally, when do you have to talk to the supervisor, aside from when you have a problem?*

MAN: En la entrada.
TRANSLATION: *When you come in.*

MODERATOR: Okay.

MAN: Entrando al turno está él, pues le pregunto si hay alguna novedad o una vaca que hay que ponerle más cuidado en su ordeño o tratar jalarla de corral de parlor y de regreso. Siempre es nuestro . . .
TRANSLATION: *When you come in for a shift, he is there, and, well, you ask him if there's anything new or if there are any cows that you have to watch more carefully for their milking or as you try to bring them from the corral to the parlor and back. It's always our . . .*

MODERATOR: O sea, en la mañana, ¿se comunican con él primero antes de partir . . .
TRANSLATION: *I mean, in the morning, do you talk to him first before you leave . . .*

MAN: No siempre.
TRANSLATION: *Not always.*

MAN: No siempre.
TRANSLATION: *Not always.*

MAN: No, solo dependiendo. A veces si hay algo que hacer, pues ya . . . trabaja así o, ahora . . . junta cada dos semanas entonces, ya, cualquier cosa lo platicamos en la junta también cuando . . . hay junta cada dos semanas entonces, ya nos ponemos de acuerdo en cosas que el patrón quiere decirnos . . .
TRANSLATION: *No, it just depends. Sometimes, if there is something to do, then . . . work like that or, right now . . . meeting every two weeks. So then if there's anything, we talk about it in the meeting too when . . . there is a meeting every two weeks. So then we agree on things that the boss wants to tell us . . .*

MODERATOR: ¿Y participan todos en la junta?
TRANSLATION: *And everyone participates in the meeting?*

MAN: Sí, todos los empleados que . . .
TRANSLATION: *Yes, all the employees that . . .*

MODERATOR: Cada dos semanas.
TRANSLATION: *Every two weeks.*

MAN: Últimamente, está haciendo de cada dos semanas.
TRANSLATION: *Lately, it's been every two weeks.*

MODERATOR: Y ¿cómo manejan cosas? ¿Cómo funcionan los descansos en la farma, o sea, la granja, por ejemplo, si tienen algún break de almuerzo o específico?
TRANSLATION: *And how are things handled? How do the breaks work on the farm, I mean, the dairy, for example, do you have a specific lunch break?*

MAN: Depende cada quien como quiera trabajar, descansa . . . días.
TRANSLATION: *It depends on each individual how they want to work, days off . . . days.*

MODERATOR: Pero, por ejemplo, en el día. O sea, si hacen el break de almuerzo o . . .
TRANSLATION: *But, for example, during the day, I mean, do you have a lunch break or . . .*

MAN: Ah, se toma su tiempo para ir a, para venir a tomar un café . . . particular . . . se viene y toma su café, y se regresa ya para dos horas pues igual se viene a comer algo. Pero la verdad más es, más pesada en la mañana porque es cuando se viene uno muy temprano sin comer nada y nada, entonces es muy necesario venir a tomar un café o ya después, almorzar algo. Ya cuando entra uno en la tarde, pues igual, ya viene de su casa entonces . . . come algo antes de venir.
TRANSLATION: *Oh, you can take your time to come in and have some coffee . . . particular . . . you come, drink your coffee, and you go back and then two hours later, same thing, you come back in to eat something. But to be honest, it's more, in the morning, it's harder because you come in early without eating anything, so then it's really necessary to come in and have some coffee, and then have something to eat later. And when you come in the afternoon, well, same, you come from home so . . . eat something before you come.*

MODERATOR: O sea, ¿no hay como algo estructurado?
TRANSLATION: *So there isn't like something structured?*

MAN: Ah, no.
TRANSLATION: *Oh, no.*

MODERATOR: ¿De cierta hora a cierta hora?
TRANSLATION: *From a certain time to a certain time?*

MAN: No.
TRANSLATION: *No.*

MAN: No, lo que hacen los que están ordeñando, por ejemplo, de que de repente, desconectan los dos lados al mismo tiempo, y ya uno se viene a desayunar. O por ejemplo, vienen y calientan algún desayuno, regresan otra vez y ya en unos diez, cinco minutos regresan otra vez. Es así, o sea, no

es que hay una hora específica, un tiempo específico, no.
TRANSLATION: *No, what the people who are milking do, for example, is that they will disconnect both sides at the same time, and then you can come in and get something to eat. Or, for example, they come in and heat something up for breakfast. Then they go back. And then they come back in like ten or five minutes. That's how it is. I mean, it's not like there's a specific hour or a specific time, no.*

MODERATOR: Pero se pueden tomar un descanso de vez en cuando para venir a comer.
TRANSLATION: *But you can come in for a break once in a while to come and eat.*

MAN: Sí.
TRANSLATION: *Yes.*

MAN: Pues, de hecho, en la mañana he visto que son dos momentos.
TRANSLATION: *Well, in fact, in the morning, I've seen that there are two moments.*

MODERATOR: Okay.

WOMAN: Nada más nos cambiamos so uno se queda en la parlor y uno viene a desayunar o a almorzar.
TRANSLATION: *We just switch so one person stays in the parlor, and the other comes to have breakfast or have lunch.*

MAN: Sí. Lo que yo he visto en la mañana es así. Casi son dos momentos. En las tardes la verdad, yo no veo como le hacen, ni en la noche que no estoy. Pero en la mañana, es así.
TRANSLATION: *Yeah. From what I've seen, that's how it is in the morning. There are two times. But in the afternoon, to be honest, I don't know how they do it or at night because I'm not here. But in the morning, that's how it is.*

MAN: . . . así porque pues no puede dejar la parlor solo tampoco. Tiene que estar . . . checando. Y en lo que uno checa, pues la otra se va. Se viene a . . . comer algo. Y luego ya cuando regresa la otra, pues ya puede ir la otra persona. O sea, dependiendo como se ponga de acuerdo con el compañero con el que está trabajando. Tiene mucho que ver.
TRANSLATION: *. . . like that because, well, you can't leave the parlor unattended either. You have to be there . . . checking. And while one person is checking, the other can go. Then they come back . . . eat something. And then when the other comes back, well, then the other person can go. I mean, it depends on how you arrange it with your coworker that you're working with. That has a lot to do with it.*

MODERATOR: Usted también, qué trabaja como un . . .
TRANSLATION: *You as well, since you work as a . . .*

MAN: Sí.
TRANSLATION: *Yes.*

MODERATOR: ¿Sí? ¿También puede venir acá?
TRANSLATION: *Yeah? And you also, you can come here?*

MAN: Sí, puedo venir . . . muchas veces cuando no tengo que calentar nada paro el tractor y arriba oyendo música, tomando café me como mi desayuno.

TRANSLATION: *Yes, I can come . . . a lot of times when I don't have to warm anything up, I can stop the tractor, and then I'm up there listening to music, drinking coffee, and I eat my breakfast.*

MODERATOR: Okay. ¿Y siempre vienen acá para comer, o se van, pueden comer donde están trabajando?
TRANSLATION: *Okay. And do you always come here to eat, or do you go, can you eat where you are working?*

MAN: No es necesario.
TRANSLATION: *It's not necessary.*

MAN: Hay dos personas trabajando.
TRANSLATION: *There are two people working.*

MAN: Porque pues, es mas higiénico que estar allá entre las vacas comiendo. No es apropiado eso. Por lo regular, bueno, pues, ya como le digo . . . afuera. Pues, afuera se puede porque no tiene contacto con los animales y eso, pero, pues, aquí en la parlor, es muy necesario venir a la cocina comer.
TRANSLATION: *Because, well, it's more hygienic to be here versus being there eating amongst all the cows. That's not appropriate. Normally, well, like I said . . . outside. Well, outside you can because you're not in contact with the animals and all that, but, well, here in the parlor, it's necessary to come to the kitchen to eat.*

MAN: En mi caso, sí, hay una estructura de horario. Generalmente las actividades están ya marcadas por hora. Entonces a las 10:00 de la mañana, estoy, de 10:00 a 10:15, tomo un break. Y ya está, y es, coincide con las actividades que se hacen. Entonces siempre de 10:00 a 10:15, estoy tomando break. Y sí, siempre es aquí.
TRANSLATION: *In my case, yeah, there is a structured schedule. Generally, my activities are scheduled by the hour. So at 10:00 in the morning, I'm, from 10:00 to 10:15, I take a break. And that's it, and it, it coincides with the activities that I do. So from 10:00 to 10:15, I'm always taking my break. And, yeah, that's how it is all the time.*

MODERATOR: ¿Y ustedes también?
TRANSLATION: *And you guys too?*

MAN: Sí, somos igual.
TRANSLATION: *Yeah, we're the same.*

MODERATOR: Okay. ¿Y qué reglas tiene la granja en términos de vestimenta, qué tiene que utilizar para trabajar?
TRANSLATION: *Okay. And what rules do you have on the farm regarding how you dress? What clothes do you have to wear to work?*

MAN: . . .

MAN: Está haciendo más exigente . . .
TRANSLATION: *They are being more strict . . .*

MAN: Los overoles.
TRANSLATION: *Overalls.*

MAN: Y de hecho . . . ¿no tienes? . . . porque había unos allí . . .
TRANSLATION: *And in fact . . . don't you have that . . . because there were some there . . .*

MAN: Sí, porque de hecho él siempre dice que debemos de usar uniformes, pero ya ves siempre hay personas que no lo usan. O sea, no es porque no lo tengan, simplemente por su mismo criterio de no . . . pero lo que él dice es que es muy necesario, por ejemplo, colores, así como pues . . . pues, sí, como ese color naranja. Dice que no lo hace tanto porque te gusta, simplemente es por precaución, seguridad, y protocolos de seguridad de . . .
TRANSLATION: *Yeah, because, in fact, he always says that we should wear uniforms, but, you know, there are always people who don't wear them. I mean, it's not because they don't have them, it's just because of their own agenda that they don't . . . but what he says is that it's very necessary, for example, colors, like . . . well, yeah, like that color orange. He says that it's not because you like it, it's just as a precaution, for safety, and safety protocols . . .*

MAN: Creo que el año pasado entró una nueva ley que exige que se andan con ropa fosforescente.
TRANSLATION: *I think last year, there was a new law that requires that you have this fluorescent clothing.*

MAN: De hecho, en la oscuridad o en así luego ya ves que ahorita con el clima también, entonces es mejor, yo creo que usar algo así pues, por seguridad de uno mismo.
TRANSLATION: *In fact, in the dark or like this, you see that right now with the weather too, so this is better. I think that using something like this, well, it's for your own safety.*

MAN: Pues, son botas, overol, y la camisa.
TRANSLATION: *Well, it's boots, overalls, and the shirt.*

MODERATOR: Y la camisa como color.
TRANSLATION: *The colored shirt.*

MAN: Sí, . . .
TRANSLATION: *Yeah . . .*

MAN: Botas naranjas.
TRANSLATION: *Orange boots.*

MAN: Botas naranjas también . . .
TRANSLATION: *Orange boots too . . .*

MODERATOR: O sea, como las botas, el overol, y . . . es obligatorio.
TRANSLATION: *I mean, like the boots, the overall, and . . . is required.*

MAN: Sí.
TRANSLATION: *Yeah.*

MAN: Y las mangas.
TRANSLATION: *And the sleeves.*

MAN: Las mangas de los ordeñadores.
TRANSLATION: *The sleeves for the milkers.*

MODERATOR: Los guantes también.
TRANSLATION: *The gloves too.*

MAN: Guantes.
TRANSLATION: *Gloves.*

MAN: Guantes, sí . . .
TRANSLATION: *Gloves, yeah . . .*

MODERATOR: ¿Y usan estos protectores de ojo?
TRANSLATION: *And do you use eye protection?*

MAN: Sí, también los lentes, pero como te digo . . .
TRANSLATION: *Yes, the safety glasses too, but like I said . . .*

MAN: Nadie los usa.
TRANSLATION: *Nobody uses them.*

MAN: Nadie. Es de otro . . . porque no se puede decir esto no me lo . . . simplemente que, en un momento, usaba todo, pero no por lo regular.
TRANSLATION: *Nobody. It's another . . . because you can say this doesn't . . . just that, sometimes you use everything, but not normally.*

MAN: Es que, con las gafas, es un poco incómodo.
TRANSLATION: *It's that the safety glasses are a little bit uncomfortable.*

MODERATOR: Incómodo.
TRANSLATION: *Uncomfortable.*

WOMAN: Se nublen, y luego ya no miro.
TRANSLATION: They fog up, and then I can't see.

MAN: . . . la respiración te . . . con los lentes y no se mira. Pero en otras cuestiones, pues las botas son con casquillo. No son botas sencillas, esas botas tienen casquillo por si . . .
TRANSLATION: *. . . from your breath . . . with the safety glasses, and you can't see. But on another topic, the boots are steel-toed. They're not just simple boots. These boots have a steel toe.*

MAN: Punta de fierro.
TRANSLATION: *Steel toe.*

MAN: La punta de fierro que, si le llega a pisar una vaca, no le lastima los dedos pues ya tiene protección también. Entonces no puedes, en este sentido, está muy bien.
TRANSLATION: *The steel toe is so that if a cow steps on you, they don't hurt your toes, so you have protection from that. So you can't, in that way, it's really good.*

MODERATOR: Okay. Ah, y con, ¿qué ropa de protección tienen que usar, si, por ejemplo, está trabajando con un animal enfermo o algo, tiene que usar algo especial, o no?
TRANSLATION: *Okay. Oh, and with, what protective clothing do you have to wear, if, for example, you're working with a sick animal or something, do you have to wear anything special or not?*

MAN: Pues un overol completo, hasta acá.
TRANSLATION: *Well, a full overall, up to here.*

MAN: Pues normal así como lo mira. Si va a atender a una vaca o un becerro, pues, así va, nada más con los guantes, siempre guantes.
TRANSLATION: *Well, just normal, just like you see. If you're going to take care of a cow or a calf, well, this is how you go, just with gloves, always with gloves.*

MAN: Sí, guantes. Sí.
TRANSLATION: *Yeah, gloves. Yeah.*

MAN: Sí. Y si hay que checar a una vaca, ya se checa con los guantes que se usan.
TRANSLATION: *Yeah. And if you have to check a cow, you check them with the gloves they use.*

MAN: Guantes para inseminar son diferentes, pero siempre con, porque se usa líquido también que se le echa en el ombligo . . . y eso mancha mucho la piel. Entonces todos eso no se usa así nada más. Tiene que . . .
TRANSLATION: *Gloves for insemination are different, but always with, because you use a liquid that you put on their navels . . . that really stains your skin. So you can't just use that stuff without anything else. You have to . . .*

MODERATOR: ¿A veces usan mascarilla, o no?
TRANSLATION: *Do you ever use masks, or no?*

MAN: Pues, sí, a veces pero . . .
TRANSLATION: *Well, yeah, sometimes but . . .*

MAN: Dependiendo.
TRANSLATION: *It depends.*

MAN: Depende del trabajo.
TRANSLATION: *It depends on the job.*

MAN: Cuando usa . . .
TRANSLATION: *When you use . . .*

MAN: Cuando usa . . . y el polvo y eso, entonces, sí, es muy necesario cubrirse la nariz porque todo se va, sí.
TRANSLATION: *When you use . . . and the dust and all that, so, yeah, it's necessary to cover your nose because it gets in there, yeah.*

MODERATOR: ¿Y qué piensan esta como medida de vestimenta, les parece que . . .
TRANSLATION: *And what do you think about the rules for clothing, do they seem . . .*

MAN: Está bien.
TRANSLATION: *They're fine.*

MAN: Está bien.
TRANSLATION: *They're fine.*

MAN: Como le digo, es . . . de cada persona.
TRANSLATION: *Like I said, it's . . . of every person.*

MAN: Yo creo que no se podría hacer el trabajo con otra ropa.
TRANSLATION: *I don't think you could do the job wearing other clothing.*

MODERATOR: ¿Se puede hacer, o no?
TRANSLATION: *You could or couldn't?*

MAN: No, no se puede hacer. O sea, tiene que ser esta porque pues, es repelente para el agua, para las manches, para es fácil de lavar. Entonces otro tipo de ropa no se . . .
TRANSLATION: *No, you couldn't do it. I mean, this is what is has to be because, it's water resistant, stain resistant. It's easy to wash. So another kind of clothing wouldn't . . .*

MODERATOR: ¿También piensan lo mismo ustedes?
TRANSLATION: *Do you guys think the same thing?*

MAN: Pues, sí. Lo usamos.
TRANSLATION: *Well, yeah. We use it.*

MAN: En mi caso no necesito usar overol nada más que la chamarra.
TRANSLATION: *In my case, I don't need to wear overalls or anything besides the jacket.*

MODERATOR: Claro, porque no está . . .
TRANSLATION: *Of course, because you're not . . .*

MAN: No tengo contacto con ningún químico con agua con nada. Prácticamente, soy operador de maquina. Pues lo único que exigen tu chamarra fosforescente. Y en verano, tenemos unos chalecos esos que usan los que trabajan en los caminos, en las carreteras, estos nos ponemos en verano el lugar de la chamarra o el sudadero.
TRANSLATION: *I don't have any contact with any chemicals or water or anything like that. I'm basically just a machine operator. Well, the only thing they require you to wear is your fluorescent jacket. And in the summertime, we have these vests that the people who work on the roads, on the highways wear. We put those on in the summer and then the jacket or the sweatshirt.*

MODERATOR: ¿También, piensan lo mismo, están de acuerdo ustedes, o?
TRANSLATION: *Do you guys also think the same, do you agree with that, or?*

MAN: Sí, está bien.
TRANSLATION: *Yeah, it's fine.*

MAN: No sé cómo son en otros ranchos.
TRANSLATION: *I don't know how it is on other farms.*

MODERATOR: . . .

MAN: De hecho, eso es el primer rancho que es así en los otros que he estado.
TRANSLATION: *In fact, this is the first farm that is like this from the others I've been on.*

MAN: Sí, para mi también.
TRANSLATION: *Yeah, for me too.*

MODERATOR: ¿Sí?
TRANSLATION: *Yeah?*

MAN: Mm-hmm.

MAN: Sí.
TRANSLATION: *Yeah.*

MAN: . . . trabajo que están todos uniformes como las, pues las medidas de seguridad que toma. He trabajado en otros tres ranchos, pero no . . .
TRANSLATION: *. . . job where you're all in the same uniform like, well, the safety measures that you take. I've worked on other farms, but not . . .*

MODERATOR: . . .

MAN: . . . como quieras y es problema tuyo ya. Tu patrón casi no toma, pues, más que nada, con que salga el trabajo es más que suficiente para ellos.
TRANSLATION: *. . . however you want, and it's your problem. Your boss doesn't really, well, more than anything, as long as the work is getting done, that's enough for them.*

MODERATOR: Claro.
TRANSLATION: *Of course.*

MAN: Y en este sentido, este rancho es, lo veo muy diferente que esta, las otras.
TRANSLATION: *And in that sense, this farm is, I see it very differently from the others.*

MODERATOR: Súper. Y bueno, ¿hay algún momento difícil ocupa toda la vestimenta, así como que diga no es necesaria tantas cosas?
TRANSLATION: *Super. And, well, are there any times where it's tough to wear the whole uniform, where you're like, all this stuff isn't necessary.*

MAN: No. Está hasta cómodo.
TRANSLATION: *No. It's even comfortable.*

MAN: Eso es mi pregunta. Digo porque está, pero hay personas que no lo quieren usar. O sea, ¿si saben que está bien, por qué no lo usan? Es lo primero.
TRANSLATION: *That's my question. I mean, because there is, but there are people that don't want to wear it. I mean, if they know that it's for their own good, why don't they wear it? That's the first thing.*

MODERATOR: Sí. Hay que preguntar a los que no lo usan.
TRANSLATION: *Yeah. You'd have to ask the ones who don't wear it.*

MAN: Sí.
TRANSLATION: *Yeah.*

WOMAN: Sí.
TRANSLATION: *Yeah.*

MAN: Sí, porque el patrón siempre se me acerca, ¿y porque esos dos no están usando el uniforme o sus camisas? Y yo voy y les pregunto. Ah, es que está grande, o es que está chico, o es que no me gusta. O sea, siempre me acerca . . . ¿y por qué? Pregúntales por qué no están usando el uniforme. Y es los que siempre responden.
TRANSLATION: *Yeah, because the boss always just comes over to me, why aren't those guys using the uniform or their shirts? And I go over and ask them. Oh, it's too big, or it's too small, or I don't like it. I mean, he always comes over . . . and why? Ask them why they're not using the uniform. And that is what they always say.*

MODERATOR: O sea, cuando ellos responden porque no les gustan o . . .
TRANSLATION: *So when they respond, it's because they don't like it or . . .*

MAN: O que no les queda a veces.
TRANSLATION: *Or sometimes that it doesn't fit them.*

MAN: Prácticamente . . . responsabilidad de esas personas. Es lo más lógico que puedo encontrar.
TRANSLATION: *Practically . . . responsibility of those people. That's the most logical thing I can think of.*

MODERATOR: Claro.
TRANSLATION: *Of Course.*

MAN: Si sabes que es para tu seguridad, debería de usarlo, ¿no?
TRANSLATION: *If you know it's for your own safety, you should use it, right?*

MODERATOR: Mm-hmm. ¿Y cuáles son, por ejemplo, las practicas de lavado de manos acá en su rutina de trabajo? ¿Cuándo tienen que lavarse las manos? ¿Cuándo no? ¿Cuando, si tienen alguna como regla o un protocolo?
TRANSLATION: *Mm-hmm. And which are the, for example, the hand washing rules here during your work routine? When do you have to wash your hands? When do you not? Do you have any rules or protocols?*

MAN: No.

MAN: Nada más con las botas para entrar aquí.
TRANSLATION: *Just with the boots to come in here.*

MODERATOR: ¿Qué tienen que hacer?
TRANSLATION: *What do you have to do?*

MAN: Lavarse nada más las botas para entrar aquí.
TRANSLATION: *You just have to wash your boots off to come in here.*

MODERATOR: Okay.

MAN: Para entrar al área de los becerros, hay que lavarse las botas.
TRANSLATION: *To go into the area where the calves are, you have to wash your boots.*

MODERATOR: Mm-hmm.

MAN: Sí, para tener más limpio todo alrededor.
TRANSLATION: *Yeah, to keep the whole surrounding area clean.*

WOMAN: Y para no andar agarrando las puertas así con los guantes sucios.
TRANSLATION: *And you can't go around grabbing the doors with dirty gloves.*

MODERATOR: Okay.

MAN: Pues, aquí se lava las manos para tomar algo, para ir al baño, para salir del baño.
TRANSLATION: *Well, you wash your hands to drink something, to go to the bathroom, when you come out of the bathroom.*

MAN: Se quitan los guantes, ¿no?
TRANSLATION: *You take off your gloves, right?*

MAN: No, sí se quita, nos quitamos los guantes que será cada dos, tres líneas de ordeñando. Cuando se mueve un becerro, se cambian los guantes. Tiene que agarrar el becerro con guantes limpios nuevos, más que todo. Entrando en su área donde están también, botas limpias, guantes nuevos, y . . .
TRANSLATION: *Oh, yeah, you take them off. We take off our gloves, what is it, every two or three lines for milking. You also change your gloves when you move a calf. More than anything, you have to have clean, new gloves on when you touch a calf. When you go into the area where they are too, clean boots, new gloves, and . . .*

MODERATOR: Y ¿Cuándo se cambian los guantes, se tiene que lavar las manos, o no es necesario?
TRANSLATION: *And when you change your gloves, do you have to wash your hands, or is that not necessary?*

MAN: Específicamente, no hay una regla que tenga que, que tienes que lavar las manos antes o después de hacer tal cosa. Es, yo pienso que responsabilidad de cada unos o del encargado del patrón que ya saben qué es lo que les tiene que decir a los, que nos tiene que decir a nosotros. Pero específicamente, no hay una regla. Nada más lo que está diciendo los dueños que cambien los guantes limpios cada vez que tiene a un becerro o algo así.
TRANSLATION: *There aren't any specific rules that you have to wash your hands before or after doing a particular thing. It's, I think it's everyone's responsibility or the boss is in charge of it, and they know what they have to tell them, or what they have to tell us. But there aren't any specific rules. Just whatever the owners are saying about changing to clean gloves every time you touch a calf, things like that.*

MAN: También depende de la actividad porque yo sí trabajo con mucha agua, entonces siempre me estoy mojando las manos. Entonces pues siempre me las tengo que estar lavando los, casi se lavan solos, no.
TRANSLATION: *It also depends on the activity you're doing because if I work with a lot of water, then I'm always getting my hands wet. So, well, I'm always washing my, they're basically washing themselves, right.*

MODERATOR: Claro. Sí, ¿usted también piensan lo mismo?
TRANSLATION: *Of course. Yeah, do you feel the same way?*

MAN: Sí.
TRANSLATION: *Yeah.*

MODERATOR: Okay. Y a ver, ¿en qué ocasiones, por ejemplo, que piensan que es más probable que se tengan que lavar las manos más frecuentemente? Por ejemplo, no sé en atender un animal enfermo o en la ordeña, no sé.
TRANSLATION: *Okay. And let's see, on what occasions, for example, what do you think is the most probably reason that you have to wash your hands frequently? For example, I don't know, taking care of a sick animal or milking, I don't know.*

MAN: Sí, eso, uno . . .
TRANSLATION: *Yeah, that you . . .*

MAN: . . .

MAN: . . . becerros, y después de ir al baño y ya antes y después de comer también.
TRANSLATION: *. . . calves, and then after going to the bathroom and before and after eating too.*

MAN: Cuidarlos.
TRANSLATION: *Taking care of them.*

MAN: Sí.
TRANSLATION: *Yeah.*

MAN: Si está cuidando una vaca enferma, empezar a curarla y después pienso que es el momento de lavarse las manos.
TRANSLATION: *If you're taking care of a sick cow, you take care of it, and then afterwards, I think that's the right time to wash your hands.*

MODERATOR: Y ¿qué producto usan para lavarse las manos, tienen qué tipo de jabón o jabón normal?
TRANSLATION: *And what product do you use to wash your hands, do you have any kind of soap or normal soap?*

MAN: Jabón.
TRANSLATION: *Soap.*

MAN: Hay jabón.
TRANSLATION: *There is soap.*

MAN: Hay en donde están los lavamanos en el baño y en el otro lado, hay jabón que trae una compañía. Es, no sé qué jabón sea, pero es para lavarse las manos.
TRANSLATION: *There is some where we wash our hands in the bathroom on the other side, there is soap that a company brings. I don't know what kind of soap that is, but it's for washing your hands.*

MAN: Y aquí ocupamos uno atrás.
TRANSLATION: *And here, we have one back there.*

MODERATOR: Okay. ¿Tiene eso, como desinfectante?
TRANSLATION: *Okay. You have like disinfectant?*

MAN: Sanitizante.
TRANSLATION: *Sanitizer.*

MODERATOR: Sanitizante.
TRANSLATION: *Sanitizer.*

MAN: Sí, en todas las áreas, bueno, no todas, pero el baño hay junto a la parlor, aquí está también uno. Es un jabón especial para manos con toallas y jabón.
TRANSLATION: *Yeah, in all the areas, well, not all, but in the bathroom that's attached to the parlor, here there is one too. It's a special kind of soap for hands with towels and soap.*

MODERATOR: ¿Usan toallas desechables?
TRANSLATION: *Do you use disposable towels?*

MAN: Sí.
TRANSLATION: *Yes.*

MAN: De papel.
TRANSLATION: *Paper towels*

MODERATOR: De papel. Okay.
TRANSLATION: *Paper. Okay.*

MAN: El patrón es muy exigente en ese sentido de que decir, quiero que tenga jabón siempre, y quiero que haya toallas siempre. Cuando se lavan las manos, se sequen allí mismo.
TRANSLATION: *The boss is very strict in the sense that he says, I want you all to always have soap, and I want there to always be towels. When you wash your hands, you can dry them there too.*

MODERATOR: Y bueno, ¿cómo se preparan para terminar su turno, para salir del día? ¿Cuál sería como su rutina para irse a la casa?
TRANSLATION: *Okay, and how do you prepare to end your shift, to leave for the day? What would be your routine to go home?*

MAN: Salir corriendo.
TRANSLATION: *Get out as fast as you can.*

MAN: . . . limpio todo, todo lo que se haya ocupado, salgo más limpio que se . . .
TRANSLATION: *. . . clean everything, everything you have used, I leave here cleaner than when . . .*

MAN: Estamos trabajando en eso. O sea, en la junta pasada se habló de so de que antes de salir, de checar que todo este limpio desde los trastes que ocupaste, la mesa, tu ropa y las botas, entonces limpio antes de irse cada quien de su turno.
TRANSLATION: *We are working on that. I mean, in the last meeting we talked about before you leave, checking that everything is clean down to the dishes you used, the table, your clothes, your boots, so it's all clean before each person leaves to go home after their shift.*

MODERATOR: Mm-hmm. Sí.
TRANSLATION: *Mm-hmm. Yeah.*

MAN: Estamos trabajando en eso.
TRANSLATION: *We are working on that.*

MODERATOR: ¿Y la ropa la dejan acá, o cómo le hacen? ¿O sea, lo llevan para lavar o?
TRANSLATION: *And you leave the clothes here, or how do you do that? I mean, are they taken away to be washed or?*

MAN: Hay lavadora que, lavadora grande y secadora . . .
TRANSLATION: *There is a washing machine that, a big washing machine and a dryer . . .*

[Simultaneous discussion]

MAN: . . . hay una compañía que viene y recoge la ropa sucia y trae su ropa limpia.
TRANSLATION: *. . . there is a company that comes to pick up the dirty clothes and brings clean clothes.*

MODERATOR: ¿Todos los días?
TRANSLATION: *Every day?*

MAN: No.

MAN: No.

MAN: Cada semana. De hecho, trae seis, siete por semana, y se lleva las siete, y le deja otra siete, y así. Cada miércoles . . . y se lleva, hay un cesto, una bolsa que echan allí las camisas sucias. Y ya viene, se lleva eso y deja más, y así.
TRANSLATION: *Every week. Actually, they bring six, or seven per week, and they take the seven, and they leave another seven, like that. Every Wednesday . . . and they bring, there is a hamper, a bag that all the dirty shirts go in. And then they come, and they take those and leave more, like that.*

MAN: En cuanto a . . .
TRANSLATION: *In terms of . . .*

MAN: En cuanto a las camisas. Y ya lo demás lo que son los overoles, pues, ya se lavan aquí en la lavadora cada . . .
TRANSLATION: *When it comes to the shirts. And then the rest, which are overalls, well, those get washed in the washing machine here every . . .*

MODERATOR: Cómo que cada uno cuando se va deja su . . .
TRANSLATION: *So when each person goes, they leave their . . .*

MAN: Sí, deja la ropa lavando. Y ya para el turno que viene la seca.
TRANSLATION: *Yeah, they put the clothes in the washer. And then the next shift dries them.*

MAN: . . . la casa, se puede decir usamos todo lo que es de trabajo en el área de donde están las otras cosas. Se deja lavarlo y al otro día un compañero la pone a secar y la encontramos allí en los lockers.
TRANSLATION: *. . . home, you could say that we use everything that is for work in the area where the other things are. Everything is washed and the next day, a coworker puts it in the dryer, and we find it there in the lockers.*

MAN: Lo que yo puedo decir es que yo con esta ropa es con la que yo vengo. Solamente me pongo el overol y las botas. Y lo que lavo en casa son las sudaderas estas. Y ya tengo otra sudadera para ir y venir de la casa.
TRANSLATION: *What I can say is that these clothes are what I come in. I just put on the overall and the boots. And the things I wash at home are these sweatshirts. I have another sweatshirt to come home in.*

MODERATOR: Okay. ¿Ustedes también hacen eso?
TRANSLATION: *Okay. And you guys do that too?*

MAN: Sí lo mismo . . . otra parte, pero después . . .
TRANSLATION: *Yeah, the same . . . another place, but later . . .*

MODERATOR: Okay. O sea, que acá tienen . . .
TRANSLATION: *Okay. So here you have . . .*

MAN: Un área.
TRANSLATION: *An area.*

MODERATOR: Un área para cambiarse y para ponerse la ropa y todo. Súper.
TRANSLATION: *An area to get changed and to put on your clothes and everything. Super.*

MAN: En mi caso, yo no . . .
TRANSLATION: *In my case, I don't . . .*

MODERATOR: Mm-hmm

MAN: Como me vengo así, me vuelvo en la misma ropa porque yo pues, no estoy en la sala de ordeña, no estoy con becerros, como prácticamente afuera nada más. Entonces pues como me puede . . . no estoy sucio.
TRANSLATION: *Since I come like this, I go home in the same cloths because, well, I'm not in the parlor. I'm not with the calves. I'm basically just outside, and that's it.*

MODERATOR: Mm-hmm.

MAN: No hay mucho contacto con las vacas y eso.
TRANSLATION: *There's isn't a lot of contact with the cows and all that.*

MODERATOR: Sí. ¿Qué más? Ah, bueno. ¿Cómo se, vamos a . . . un poco a saber cómo se maneja la leche cruda en la granja y los productos crudos que tiene, si es que producen aquí algo?
TRANSLATION: *Yeah. What else? Oh, okay. How, we're going to . . . a little to find out how raw milk is handled on the farm and the raw products that you have, if you produce any here.*

MAN: No.

MAN: No. Solo la leche.
TRANSLATION: *No. Just the milk.*

MODERATOR: Solo la leche. Y bueno, ¿cuáles son como las reglas que tienen aquí sobre la leche, sobre la leche cruda o si la pueden tomar o?

TRANSLATION: *Just the milk. Okay, what are the rules that you have about the milk, the raw milk or if you can drink it or?*

MAN: Más específico.
TRANSLATION: *More specific.*

MODERATOR: Leche, como la leche recién salida de la vaca.
TRANSLATION: *Milk, like the milk that has just come out of the cow.*

MAN: Sí, pero la pregunta más específica.
TRANSLATION: *Yeah, but can you be more specific with the question?*

MODERATOR: Es si, por ejemplo, ¿qué reglas tienen de que no se pueden, ustedes no la pueden tomar o?
TRANSLATION: *It is, for example, what rules do you have that you can't, you guys can't drink it or?*

MAN: Nadie la toma la leche.
TRANSLATION: *Nobody drinks the milk.*

MAN: Nadie la toma la leche cruda.
TRANSLATION: *Nobody drinks the raw milk.*

MAN: . . . cuando está ordeñando la vaca, nada más se exprime, pero de allí pues se conecta la manga y toda la leche se pasa directo al tanque por la tubería. Nunca ves la leche. Al menos que una vaca está enferma. Entonces, sí, la ordeñan aparte en un bote, y esa leche se tira al drenaje porque pues no sirve. En el caso de las vacas frescas, esas sí se tiene que guardar el primero y segundo ordeño porque ese es con la que se alimentan los becerros, primeros alimentos. Entonces nada más . . . pues, toda la leche se va para el tanque.
TRANSLATION: *. . . when we're milking the cow, you just squeeze out a little. But from there, you hook up the tubes, and all the milk goes through the tubes directly to the tank. You never see the milk. Unless a cow is sick. Then, yeah, you milk her separately into a bucket. And that milk gets thrown down the drain because it's useless. For the fresh cows, that we do save the first and second milking because that is what the calves are fed for their first feeds. So just . . . well, all the milk goes to the tank.*

MODERATOR: O sea, que no tienen ningún contacto con la leche.
TRANSLATION: *So you don't have any contact with the milk.*

MAN: Solo con la de las frescas. La ordeñan en bote, e inmediatamente en el . . .
TRANSLATION: *Just with the fresh cows. They get milked into a bucket, and immediately into the . . .*

MAN: . . .

MAN: Y ya este el muchacho que está con los becerros se encarga de pasteurizarla.
TRANSLATION: *And then the guy who is with the calves is in charge of pasteurizing it.*

MAN: Pero de la demás, toda la leche se va para el tanque, quién sabe para donde.
TRANSLATION: *But the rest of it, all of the milk goes to the tank, who knows where.*

MAN: El Walmart.
TRANSLATION: *The Walmart.*

MODERATOR: ¿Alguna vez ustedes o una de sus compañeras a tomado leche cruda en otro lado o leche sin pasteurizar?
TRANSLATION: *Have any of your or your coworkers ever drank raw milk anywhere or milk that hasn't been pasteurized?*

MAN: Sí.
TRANSLATION: *Yes.*

MODERATOR: ¿Sí?
TRANSLATION: *Yes?*

MAN: De los dos ranchos, yo sí he visto.
TRANSLATION: *I've seen it on the other two farms.*

MODERATOR: Okay. ¿Y qué le parece?
TRANSLATION: *Okay. And what did you think of that?*

MAN: No sé yo, desde que estaba, hace muchos, woo, cuando era niño, mi abuela no nos dejaba tomar leche de la vaca. Siempre la ponía en la estufa a hervir y luego ya nos daba la leche. Pero yo nunca he tomado leche así, cruda. No sé.
TRANSLATION: *I don't know, since I was, woo, a long time ago, when I was a kid, my grandmother wouldn't let us drink milk right from the cow. She always put it on the stove to boil and then she would give us some milk. But I've never drank raw milk. I don't know.*

MAN: Yo tampoco.
TRANSLATION: *Me neither.*

MAN: No a veces sí hay personas, le digo, que . . . una persona acá, personas diferente, no, pero es que sí quieren su botellita.
TRANSLATION: *No, sometimes there are people, I mean, that . . . a person here, different people, you know, that do want their little bottle.*

MAN: Tenía un compañero de trabajo tú Nacho, no voy a decir nombres, pues, él es americano. Lleva su termo su termal de café a la . . . de cualquier vaca, la ordeñaba y seguía tomando. Y luego teníamos gatitos entraban y salían. Agarraba el gatito y volteó el termo y ponía el gatito para que tomara del termo, y luego él seguía tomando leche. En serio.
TRANSLATION: *You had a coworker didn't you, Nacho, I'm not going to say names. Well, he is American. He brought in his thermos, his coffee thermos . . . any of the cows, he milked a cow a little and then kept drinking it. And then we had some little cats that came in and out. He grabbed a little cat and turned the thermos and put the cat there so it could drink from the thermos, and then he finished drinking the milk. Seriously.*

MODERATOR: ¿Tampoco han tomado la leche cruda?
TRANSLATION: *You've never drank the raw milk either?*

MAN: No.

MAN: Yo sí.
TRANSLATION: *I have.*

MODERATOR: ¿Sí?
TRANSLATION: *Yeah?*

MAN: Antes sí, pero no.
TRANSLATION: *I did before, but not anymore.*

MAN: La leche que manejo ya es pasteurizado. Solo a veces se le da la que resta en los recipientes se le da a los gatos igual, pero no lo tomas.
TRANSLATION: *The milk I deal with is already pasteurized. Sometimes we give what is left in the containers to the cats, but you don't drink it.*

MAN: ¿No la taza de café?
TRANSLATION: *Not into your coffee cup?*

MAN: . . .

MODERATOR: Bueno ya llegamos a pasar en la última ronda de preguntas que es más sobre el uso de antibióticos acá en la granja y quién los ocupa y todo eso de cualquieras que hay.
TRANSLATION: *Well, we've reached the last round of questions, which are more about the use of antibiotics here on the farm and who uses them and all that, which ones there are.*

MAN: Ellos son los . . .
TRANSLATION: *They are the ones who . . .*

MODERATOR: Sí. Bueno, ¿cuál es, primero, como cuál es su experiencia tomando medicamento, tomando antibiótico? ¿Alguno a tomado antibiótico?
TRANSLATION: *Yeah. Okay. What is, first, what is your experience taking medication, taking antibiotics? Have any of you taken antibiotics?*

MAN: ¿Nosotros?
TRANSLATION: *Any of us?*

MODERATOR: Sí, como . . .
TRANSLATION: *Yeah, like . . .*

MAN: Cuando traes infección.
TRANSLATION: *When you have an infection.*

MODERATOR: Uh-huh.

MAN: Solamente.
TRANSLATION: *Only.*

MAN: Pues, sí, somos mexicanos. Te duele la cabeza y tomas una aspirina. Te duele la garganta y toma penicilina.
TRANSLATION: *Well, yeah, we're Mexicans. If you have a headache, you take an aspirin. If your throat hurts, you take penicillin.*

MAN: Unos antibióticos.
TRANSLATION: *Some antibiotics.*

MODERATOR: El antibiótico es como para la bacteria.
TRANSLATION: *An antibiotic is for bacteria.*

MAN: Mm-hmm. Pues aquí casi no si no lo tiene recetada, los antibióticos siempre son con receta.
TRANSLATION: *Mm-hmm. Well, here, it it's not prescribed, antibiotics always have to be prescribed.*

MAN: Con receta.
TRANSLATION: *With a prescription.*

MODERATOR: Pero los han tomado alguna vez.
TRANSLATION: *But you have taken them at some point.*

MAN: Sí, de infección o por . . .
TRANSLATION: *Yeah, for infection or for . . .*

MAN: Antibiótico así . . . pues sí.
TRANSLATION: *Antibiotic . . . well, yes.*

MAN: Pasa todo por el médico nada más.
TRANSLATION: *Everything has to go through a doctor.*

MAN: Luego el médico te dice, te vas a tomar eso, pero no sabe ni qué es. No sí, yo creo que sí, pero . . .
TRANSLATION: *Then the doctor tells you, you're going to take this, but you don't even know what it is. Yeah, I think so, but . . .*

MODERATOR: Pero muy raro.
TRANSLATION: *But very rarely.*

MAN: Muy raro.
TRANSLATION: *Very rarely.*

MODERATOR: ¿Y en qué situaciones piensan que los antibióticos son útiles?
TRANSLATION: *And in what situations do you think antibiotics are useful?*

MAN: En el caso de infección o, pues, más que nada es para eso, para infecciones o . . .
TRANSLATION: *In the case of an infection or, well, more or less that's what it's for, for infections or . . .*

MAN: Enfermedades . . .
TRANSLATION: *Illnesses . . .*

MAN: . . . tratamiento de dental, ya tengo como dos, tres, cuatro meses o más. Y cada vez que me van a hacer algún trabajo grande, una semana antes me dan antibióticos.
TRANSLATION: *. . . dental treatment, it's been like two, three, four months or more. And every time that they're going to do a lot of work, they give me antibiotics for a week beforehand.*

MODERATOR: O sea, usted lo encuentra útil.
TRANSLATION: *So you do find it useful.*

MAN: Pues, sí.
TRANSLATION: *Well, yes.*

MODERATOR: ¿Ustedes también, o?
TRANSLATION: *And you guys too, or?*

MAN: Yo creo que no.
TRANSLATION: *I don't think so.*

MAN: Sí, porque si está uno así con un malo o muy enfermo con un antibiótico es que te ayuda más. Entre más fuerte sea la enfermedad, el antibiótico es . . . porque tú no . . . pues pasar con puro Tylenol . . . no te cura . . . algún antibiótico. O aquí a las vacas, las pone a las vacas también. Hay vacas que están con alta temperatura y con . . . tenemos que dar antibiótico.
TRANSLATION: *Yeah, because if you are not feeling well or very sick and antibiotics helps you more. The stronger the illness is, the antibiotic is . . . because you can't . . . well, get by with just Tylenol . . . doesn't cure you . . . some antibiotic. Or here with the cows, the cows get it too. There are cows with high temperatures and with . . . we have to give them antibiotics.*

MODERATOR: Y, por ejemplo, si tienen que tomar antibiótico, ¿dónde lo consiguen, o sea, cómo lo, si usted tiene que tomar, por ejemplo, usted tiene que tomar amoxicilina o algo así?
TRANSLATION: *And, for example, if you have to take an antibiotic, where do you get it, I mean, how, if you have to take, for example, you have to take amoxicillin or something like that?*

MAN: Ir al médico.
TRANSLATION: *Go to the doctor.*

MAN: Ir al médico solamente.
TRANSLATION: *Just go to the doctor.*

MAN: Solo él . . .
TRANSLATION: *Only he . . .*

MAN: Que te tiene que . . .
TRANSLATION: *He has to . . .*

MAN: Aquí no te vende sin receta.
TRANSLATION: *Here, you can't buy it without a prescription.*

MODERATOR: Okay.

MAN: Sí así . . . antibióticos yo, pero . . .
TRANSLATION: *Yeah, like that . . . antibiotics myself but . . .*

MAN: Las que uno puede . . . fácil son aspirina, Tylenol, y eso, pero es básico, no. Pero ya si necesitas más, pues como dice ustedes, para antibióticos, ya tiene que hacer a fuerza con el médico.
TRANSLATION: *The ones that you can . . . easily are aspirin, Tylenol, and all that. But that's basic, you know. But if you need more, like you said, for antibiotics, you have to make an effort to go see the doctor.*

MAN: Así nada más.
TRANSLATION: *That's the only way.*

MODERATOR: Bueno. A veces, los antibióticos dejan de funcionar contra cierta bacteria, eso está pasando ahora que se vuelven como resistente como que ya no funcionan. Y no se pueden controlar ni en una persona ni en los animales, como que les dejan de funcionar.
TRANSLATION: *Okay. Sometimes the antibiotics stop working against certain bacteria. That is happening now where they are becoming resistant like they don't work. And it can't be controlled in people or in animals, like it stops working for them.*

MAN: ¿A qué sea de eso?
TRANSLATION: *Why is that?*

MODERATOR: Eso es lo que están investigando.
TRANSLATION: *That is what they are investigating.*

MAN: Oh, okay. Yo digo porque en una ocasión, no hace mucho tiempo, el doctor me dijo que alternara Advil con Tylenol porque si tomo solamente Tylenol, como a los tres cuatro días, la función ya no sirve para nada Tylenol que tienen que ser alternados. ¿Ustedes que son investigadores, es cierto?
TRANSLATION: *Oh, okay. I'm asking because there was a time not very long ago where the doctor told me that I should alternate Advil and Tylenol because if I just take Tylenol, like after three or four days, Tylenol wouldn't work for my anymore, that they have to be alternated. Since you guys are investigators, is that true?*

MODERATOR: I think we are, maybe we can finish this.

WOMAN: Sure, yeah, that's fine.

MODERATOR: Oh, and he's asking if you know if he has to, how do you say, rotate Advil with Tylenol like because the doctor said that you can use it like only four days and then switch it to . . .

WOMAN: You can build like a . . .

MODERATOR: You can build resistance to?

WOMAN: Not necessarily like resistance, but people start to develop like, they get used to the feeling that the drug gives them, and so it doesn't work as well.

MAN: Oh, okay.

WOMAN: Also, Tylenol is hard on your liver.

WOMAN: It's hard, and Advil is hard on your kidneys.

WOMAN: Yeah, so that's probably why.

MAN: So rotate the organ that's . . .

MODERATOR: So dice que lo tiene que rotar, tiene que ir cambiando porque la gente se acostumbra al efecto, y después, ya no funciona. Y aparte el Tylenol as mal para el hígado si lo estás usando mucho tiempo.
TRANSLATION: *So they say that you have to rotate it, you have to switch it because people get used to the effect, and afterwards, it doesn't work. And aside from that, the Tylenol is bad for the liver if you are using it a lot.*

MAN: Es lo que estaba diciendo de la, de que la bacteria se aguanta . . .
TRANSLATION: *That is what he was saying that the, that the bacteria gets used to it . . .*

MAN: Resistente.
TRANSLATION: *Resistant.*

MAN: . . . es lo que dice que es ya le está pasando lo mismo. Por eso tiene que mezclarlos para que . . .
TRANSLATION: *. . . that's what they say, that the same thing is happening. That's why you have to mix them so that . . .*

MODERATOR: Algo está pasando con el Tylenol y . . .
TRANSLATION: *Something is happening with the Tylenol and . . .*

MAN: Y Advil.
TRANSLATION: *And Advil.*

MODERATOR: . . . también pasa con el antibiótico. Pero Tylenol y Advil no son antibióticos. Son analgésicos.
TRANSLATION: *. . . it also happens with antibiotics. But Tylenol and Advil are not antibiotics. They are analgesics.*

MAN: Analgésicos, ya.
TRANSLATION: *Yeah, analgesics.*

MODERATOR: Sí. entonces, bueno, ¿qué piensan sobre la resistencia antibiótico? ¿O sea, ustedes habían escuchado sobre esto que está pasando?
TRANSLATION: *Yeah. So, okay, what do you think about antibiotic resistance? I mean, have you heard about that happening?*

MAN: Yo no.
TRANSLATION: *Not me.*

MAN: No.

MAN: De hecho, sí, estaba leyendo un reportaje de algunas enfermedades que han generado resistencia sobre todo en climas fríos como en la parte norte de Europa en Estados Unidos. Unas de las que recientemente se empeora la gonorrea, ha habido una súper gonorrea, entonces creó resistencia, y ya con la misma, con el mismo tratamiento que existía, ya no se puede curar.
TRANSLATION: *In fact, yeah, I was reading a report about some diseases that have become resistant in cold climates like the northern part of Europe and the United States. One of them that recently got worse was gonorrhea, that there is a super gonorrhea. So it became resistant, and now with the same, it can't be cured with the treatment that existed before.*

MODERATOR: Exacto. Sí. ¿Y alguna de ustedes han tenido, por ejemplo, una infección que aún tomando un antibiótico no se ha podido curar, no ha podido tocar?
TRANSLATION: *Exactly. Yes. And have any of you ever had, for example, an infection that, even though you took and antibiotic, you couldn't cure?*

MAN: Yo no.
TRANSLATION: *Not me.*

MAN: No.

MAN: No.

MODERATOR: ¿No? ¿Nunca han experimentado eso? ¿No?
TRANSLATION: *No? You've never experienced that? No?*

MAN: No.

MODERATOR: Bueno. Y ¿qué papel piensan ustedes que juega la granja lechera en esta resistencia antibiótico, si es que piensan ustedes que la granja lechera tiene algo que ver con que exista resistencia de los antibióticos?
TRANSLATION: *Okay. And what role do you think dairy farms play in antibiotic resistance, if you think dairy farms have anything to do with the fact that antibiotic resistance exists?*

MAN: Puede ser.
TRANSLATION: *It could be.*

MAN: Pues, a lo mejor, sí.
TRANSLATION: *Well, perhaps, yeah.*

MAN: Si se han usado en exceso, no o se controla . . .
TRANSLATION: *If they are used in excess, or they're not controlled . . .*

MAN: Porque pues las vacas llevan mucha medicina.
TRANSLATION: *Because, well, cows get a lot of medicine.*

MODERATOR: Okay.

MAN: Inyectas a las vacas enfermas. Inyectas a las vacas que . . . inyectan a las vacas . . .
TRANSLATION: *You inject the sick cows. You inject the cows that . . . you inject the cows that . . .*

MAN: Todo es mucho químico y ya yo creo que . . .
TRANSLATION: *It's a lot of chemicals, I think.*

MAN: Mucha medicina en las vacas y eso se va en la leche. Con que la pasterizan, de todos modos, se va haciendo resistente.
TRANSLATION: *It's a lot of medicine for the cows, and all that goes into the milk. Even though they pasteurize it, it still becomes resistant.*

MODERATOR: ¿Ustedes también están de acuerdo? ¿Sí, no?
TRANSLATION: *Do you guys agree too? Yeah, no?*

MAN: De hecho . . .
TRANSLATION: *In fact . . .*

MAN: Es una idea no que se pueda dar. Pero yo pienso que igual por eso no porque pues, por lo regular, tanto como, no es tanto las granjas lecheras sino en las granjas que producen carne, también todo eso es mucho químico que se utiliza para que un pollo crezca rápido o para que un cerdo se engorda rápido. Todo es químico, entonces yo pienso que, con el paso de tiempo, aumentan mucho para tener más, más ganancias creo yo, no. Aumentan más el proceso de crecimiento y esto hace que hay más químico tanto carne como productos que se ocupan.
TRANSLATION: *That could be an idea. But I think that, all the same, for that reason, no, because, well, normally, as much, it's not just the dairy farms but the farms that produce meat. All of that also has a lot of chemicals in it that they use so that a chicken grows fast or so that a pig gets fat fast. It's all chemicals. So I think that, over time, they increase a lot so they can have bigger profits, I think. They increase the process of growth and that puts more chemicals into the meat, like the products that they use.*

MAN: Pues hay dos fenómenos. Creo que uno es directo y otro indirecto. El directo, sí, es por la leche, pero es de los medicamentos. Pero el indirecto es que, por ejemplo, las granjas lecheras son las que más producen gases de efecto invernadero, entonces todos estos gases que se van a la atmósfera, pues, afectan a las personas que viven cerca de las granjas y que están más en contacto con ellos. Entonces eso es como la parte indirecta. Pero a través de los años, pues, sí, todo esto hace que las bacterias son más difíciles de . . .
TRANSLATION: *Well, there are two phenomena. I think that one is direct, and the other is indirect. The direct one is, yes, it's from the milk, but it's from the medicines. But the indirect one is that, for example, dairy farms are the biggest producers of greenhouse gasses. So all those gasses go up into the atmosphere, well, they affect the people that live close to the dairies that have a lot of contact with them. So that is like the indirect part. But over the years, well, yeah, all of that makes the bacteria more difficult to . . .*

MAN: De controlar.
TRANSLATION: *To control.*

MODERATOR: Sí. ¿Ustedes piensan lo mismo, que tiene que ver, o piensan que no tiene nada que ver?
TRANSLATION: *Yeah. You guys feel the same, what does it have to do, or do you think it doesn't have anything to do with it?*

MAN: Todos estamos, como humanos, estamos haciendo con nuestro planeta, pues, tiene mucho que ver que la bacteria o virus que todas las enfermedades vayan en aumento y vayan haciéndose fuertes porque no estamos haciendo nada para ayudar a la naturaleza. En cuanto a las granjas, se usa mucho químico para producir más, entonces deberíamos de dar un poco más el, pues, los medicamentos o ser un poco ya más consiente de lo que estamos haciendo.
TRANSLATION: *We're all, as humans, we're all using this planet, well, that has a lot to do with the fact that bacteria or viruses, all the illnesses are getting worse and getting stronger because we aren't doing anything to help the environment. When it comes to the farms, they use a lot of chemicals to produce more. So we should do more, well, the medications, or be a little bit more conscious of what we are doing.*

MODERATOR: Y ¿qué piensan que granjas lecheras como estas se vayan a afectar por resistencia antibiótico, o piensan que no se van a afectar?

TRANSLATION: *And how do you think dairy farms like this one are affected by antibiotic resistance, or do you think that they aren't affected?*

MAN: Sí. Siempre hay referidas, tanto de animales o pérdidas(?) en una a producción, y eso afecta a todos desde el dueño, los empleados, todo.
TRANSLATION: *Yeah, there are always referendums(?), about animals or losses(?) of production, and that affects everyone from the owner to the employees, everyone.*

MAN: Si el dueño no gana, los empleados tampoco.
TRANSLATION: *If the owner isn't making money, the employees aren't either.*

MAN: Y de otra manera, siempre afecta a todo. Lo afecta los problemas . . . si por ejemplo, la gente deja de consumir leche, ¿qué va a hacer la leche, qué van a hacer con la leche? Si por ejemplo dice, no, pues la leche es la que causa el problema. Pues yo creo que, obviamente, la gente va a dejar de consumirla. ¿Y qué va a pasar con las granjas? Pues, se van para abajo.
TRANSLATION: *And in another way, it always affects everything. It affects the problems . . . if, for example, people stop drinking milk, what are they going to do with the milk? If, for example, they say, no, well, milk is the cause of the problem, well, then I think obviously that people are going to stop drinking it. And what is going to happen with these farms? Well, they'll go under.*

MAN: Pues, sí. Al rato no hay leche, no hay vacas. Está como el cáncer de mama. A la señora que les da cáncer de mama, les prohíben que tomen leche, productos lactios I don't know why.
TRANSLATION: *Well, yeah. Then there's no milk, there's no cows. It's like with breast cancer. The women who get breast cancer are told that they should drink milk, products with lactose. I don't know why.*

MAN: Por las hormonas.
TRANSLATION: *Because of the hormones.*

MAN: ¿Por las hormonas?
TRANSLATION: *Because of the hormones?*

MAN: La leche tiene muchas hormonas, y . . . hay como varias cosas en la leche que no sé pueden . . .
TRANSLATION: *The milk has a lot of hormones in it, and . . . there are like a few things in milk that they can't . . .*

MODERATOR: ¿Y qué reglas tiene la granja con respeto al uso antibiótico? ¿Cuándo tiene que aplicar el antibiótico a la vaca, y quién lo hace, todo eso?
TRANSLATION: *And what rules does the farm have regarding the use of antibiotics? When do you have to use them on a cow, who does it, all that?*

MAN: Cada vez que . . .
TRANSLATION: *Every time . . .*

MAN: . . .

MODERATOR: ¿Quiénes?
TRANSLATION: *Who?*

MAN: Ellos porque . . .
TRANSLATION: *Them because . . .*

MAN: Casi siempre son casos extremos. Sí, anteriormente usábamos mucho lo que viene siendo la penicilina, y ya usamos menos. Se está usando menos. Vacas con neumonía, estamos usando lo que es la oxitetraciclina. Igual, muy raro, es como dos veces por año. Eso no es muy común usar. Yo lo que he visto, pues, cuando yo llegué a este rancho se usaba más antibiótico para infecciones, por ejemplo, de útero y eso. Pero ha disminuido.
TRANSLATION: *It's almost always extreme cases. Yeah, before, we used a lot of penicillin. And now we use less. We are using less. For cows that get pneumonia, we are using oxytetracycline. But still, very rarely. It's like twice a year. It is not very common to do use that. And from what I have seen, well, when I came here to this farm, they were using a lot of antibiotics for infections, for example, of the uterus and all that. But it's gone down.*

MODERATOR: Ha disminuido.
TRANSLATION: *It has gone down.*

MAN: Ha disminuido.
TRANSLATION: *It has gone down.*

MODERATOR: Y ¿solo ustedes dos aplican antibiótico a la vaca?
TRANSLATION: *And you two are the only ones who give antibiotics to the cows?*

MAN: Sí.
TRANSLATION: *Yes.*

MAN: Yo también con los becerros.
TRANSLATION: *I do too with the calves.*

MODERATOR: ¿Tú también con los becerros?
TRANSLATION: *You do with the calves.*

MAN: Y él con los becerros. Ella también.
TRANSLATION: *And him, with the calves. Her too.*

MAN: Ellos son los autorizados.
TRANSLATION: *They are the authorized ones.*

MAN: Verdad . . .
TRANSLATION: *Right . . .*

WOMAN: Sí, yo también.
TRANSLATION: *Yes, me too.*

MAN: Sí, la señora que les ayude inyectar también los antibióticos.
TRANSLATION: *Yeah, the lady that also helps them inject antibiotics.*

WOMAN: Ahorita, el que estamos ocupando mucho es la penicilina.
TRANSLATION: *Right now, what we use a lot of is penicillin.*

MAN: Penicilina.
TRANSLATION: *Penicillin.*

WOMAN: Sí.
TRANSLATION: *Yeah.*

MAN: O en los becerros, se lo ocupa más, ¿verdad?
TRANSLATION: *Oh, with the calves you use it more, right?*

WOMAN: Sí, bastante.
TRANSLATION: *Yes, a lot more.*

MAN: Sí, aquí en las vacas, ha disminuido.
TRANSLATION: *Yeah. Here with the cows, it has gone down.*

MODERATOR: Okay.

MAN: Es que lo que pasa con los becerros es más como que se enferman de neumonía, especialmente en este clima húmedo. En esta temporada que un día está húmedo y otro día está caliente, y así . . .
TRANSLATION: *Well, what happens with the calves is that they get sick with pneumonia, especially in this humid climate. In the season where it's one day humid and the next day is hot, and like that . . .*

MODERATOR: Claro.
TRANSLATION: *Of course.*

MAN: Después hay que usar más la penicilina allí.
TRANSLATION: *So then you have to use more penicillin there.*

MODERATOR: Mm-hmm. ¿Pero solo serían entonces las vacas enfermas?
TRANSLATION: *Mm-hmm. But it's just with the sick cows then?*

WOMAN: Sí.
TRANSLATION: *Yes.*

MAN: Oh, sí.
TRANSLATION: *Oh, yes.*

MODERATOR: Y a los terneros, o los becerros.
TRANSLATION: *And with the calves, or the calves.*

MAN: Sí.
TRANSLATION: *Yes.*

MAN: Pues, hay un protocolo igual para identificar hasta cinco puntos, no, digo, grado por grado enfermedad de los becerros. Si no está en ese grado, todavía no se les da medicamento si no está. Hasta que al final ya es inevitable.
TRANSLATION: *Well, there is a protocol to identify, up to five points, no, I mean, level by level of illness with the calves. If it's not at this level, then don't give them medicine if they're not there until, in the end, it's unavoidable.*

MODERATOR: Y creo que me falta la última pregunta. ¿ . . . qué piensan sobre este protocolo y estas reglas sobre el uso de antibiótico? Está bien. Está mal.
TRANSLATION: *And I think that there is just one question left . . . what do you all think about this protocol and these rules about the use of antibiotics? It's good. It's bad.*

MAN: Creo que está bien. Todo con medida.
TRANSLATION: *I think it's good. Everything in moderation.*

MAN: Yo creo que nos tenemos que informar más porque yo tengo muchas veces, con el respeto del uso de medicamente adecuadamente, o sea, a veces no podemos identificar, como en los humanos, los síntomas claramente, y no sabemos, en mi caso, no sé que dar.
TRANSLATION: *I think that we need to be more informed. Because I've had it many times, with respect to the use of the right medication, I mean, sometimes we cannot identify, as humans, the clear symptoms, and we don't know, in my case, I don't know what to give.*

MODERATOR: Claro.
TRANSLATION: *Of course.*

MAN: O sea, no se si realmente . . . o mejor espero y dejo que la bacteria se cure o no o qué hacer, no. Entonces, no soy médico, entonces no sé exactamente que . . .
TRANSLATION: *I mean, I don't know if really . . . or if it's better to wait until the bacteria goes away on its own or not or what to do, you know. So I'm not a doctor. So I don't know exactly what . . .*

MODERATOR: ¿El veterinario tampoco le dice a uno qué sirve para tal cosa?
TRANSLATION: *Does the veterinarian not tell you what works for what kinds of things?*

MAN: Pues, sí.
TRANSLATION: *Well, yes.*

MAN: Sí.
TRANSLATION: *Yes.*

MODERATOR: ¿Sí?
TRANSLATION: *Yeah?*

MAN: Cada lunes viene.
TRANSLATION: *He comes every Monday.*

MAN: Yo me estoy en contacto todo el día con él, con el veterinario.
TRANSLATION: *I'm in contact with him every day, with the veterinarian.*

MODERATOR: Okay. A ustedes si le explican más o menos si esto tiene un protocolo porque . . .
TRANSLATION: *Okay. And he explains to you guys more or less if there is a protocol because . . .*

MAN: Sí. Pero viene cada lunes.
TRANSLATION: *Yes. But he comes every Monday.*

MODERATOR: Cada lunes.
TRANSLATION: *Every Monday.*

MAN: Y se le llama a menos una emergencia. Pero cada lunes que viene después de hacer el pre chequeo de las vacas. Pues, se queda un rato y si tiene uno preguntas o le decimos que cheque las vacas enfermas también. Le enseñamos las vacas y él te dice que darles que no darles.
TRANSLATION: *And we only call him if there is an emergency. But every Monday, he comes after doing a check of the cows. Well, he stays for a little bit, and if we have some questions, or we ask him to check on the sick cows too. We show him the cows, and he tells us what to give them or what not to give them.*

MAN: Y de hecho ellos saben, o el veterinario o el patrón le dice, no darle más de tres o dos días o cinco días, depende de los días del medicamento, que no sobrepase.
TRANSLATION: *And in fact, they know or the veterinarian or the boss tells them, don't give this for more than three or two days or five days, depending on the days of the medication, not to give too much.*

MODERATOR: Okay. Creo que estos son todas las preguntas. No sé si ustedes tienen alguna duda, quieren comentar algo, no sé . . .
TRANSLATION: *Okay. I think those are all the questions. I don't know if you guys have any concerns, if you want to make any comments or something, I don't know . . .*

MAN: Pues que . . .
TRANSLATION: *Well, that . . .*

MODERATOR: Hay alguna . . . algo que a lo mejor se me olvidó preguntar y que . . .
TRANSLATION: *There is a . . . something that perhaps I forgot to ask and that . . .*

MAN: De todo tipo de granjas . . . usar los medicamentos para la producción, para el crecimiento de animales y ser más naturales.
TRANSLATION: *On every kind of farm . . . use the medications for production, for the growth of animals and be more natural.*

MAN: Orgánico como dicen.
TRANSLATION: *Organic, as they say.*

MAN: Uh-huh. Orgánico. Esto sería mi respuesta, mi idea para poder sobrevivir otros años más como humanos. Si no algo va a suceder si seguimos así.
TRANSLATION: *Uh-huh. Organic. That would be my answer, my idea to survive another few years as humans. If not, something is going to happen if we continue like this.*

MAN: Extinción.
TRANSLATION: *Extinction.*

MODERATOR: Bueno. Muchas gracias por su participación. Va a ser mucha ayuda. Aquí le va a . . .
TRANSLATION: *Okay. Thank you very much for your participation. It's going to be a big help. Here is . . .*

MAN: No más un comentario que ellas que son investigadores y que saben, todas las granjas, todas las granjas, de la manure, la caca de las vacas, ellos riegan en los campos. En estos campos siembran maíz, siembran soya, siembran todo. Mi pregunta es, ¿la bacteria se va en el maíz, se va a la vaca, y luego a la leche?
TRANSLATION: *Just a comment for them since they are investigators, and they would know. On all*

the farms, all the farms, they irrigate the fields with the manure, the cow poop. In those same fields, they plant corn, they plant soy, they plant everything. My question is, does the bacteria get into the corn, go to the cow, and then to the milk?

MAN: Sí.
TRANSLATION: *Yes.*

MODERATOR: He wants to know . . .

MAN: ¿Qué tanto afecta al eco?
TRANSLATION: *How does that affect the eco?*

MODERATOR: The manure on the fields, you know the manure from the cows.

WOMAN: Mm-hmm.

MODERATOR: And he wants to know if the bacteria that are from the manure like, they spread on the corn, then the cow eats the corn, you're going to like transfer that?

WOMAN: They have to, that's why, like when there's outbreaks with like lettuce and stuff, that's what it's from. It's from the manure being spread on it and it not being cleaned. So, yes.

MODERATOR: Okay. Sí. O sea, de hecho, algunos como, por ejemplo, ahora hay problema con las lechugas, no sé si, están contaminadas las lechugas. Pero cuenta allí como estos . . . de contaminación . . .
TRANSLATION: *Okay. Yes. I mean, actually, some of the, for example, how there have been some problems with lettuce, I don't know if you, the lettuce has been contaminated. But it's like those . . . from contamination . . .*

MAN: Un aviso, sí.
TRANSLATION: *A warning, yeah.*

MODERATOR: Y por ejemplo, esta lechuga está contaminada es producto de que se regó con el guano.
TRANSLATION: *And, for example, that lettuce is contaminated because they irrigated with the manure.*

MAN: El gobierno está haciendo algo o pueda hacer algo para eliminar eso, o hay otra forma de . . .
TRANSLATION: *Is the government doing anything, or can it do anything to eliminate that, or is there another form of . . .*

MODERATOR: Sí.
TRANSLATION: *Yes.*

MAN: De no usar eso para los campos sino pueden usar otra forma de energía en . . .
TRANSLATION: *From not using it in the fields, but instead using it for another form of energy in . . .*

MODERATOR: Hay productos de energía también usar lo y transformar todo el guano en energía. Pero, sí, da poco . . .
TRANSLATION: *There are products of energy that they also use and transform all the manure into energy. But, yeah, it's a little . . .*

MAN: Debe hacer algunas partes que lo usan para este, por el . . . ¿cómo de dice? La . . .
TRANSLATION: *They should do it in some parts that use it for that, for the . . . what's it called? The . . .*

MODERATOR: A claro. Sí, ese . . . idea también. Pero, sí, la verdad. Deberían cuidar con eso. De limpiar bien las cosas que también que uno consume . . .
TRANSLATION: *Oh, of course. Yeah, that . . . idea too. But, yeah, for sure. You should be careful with that. Clean things off well so that you don't consume . . .*

MAN: Pues, yo veo que todo está dando vuelta. Es un círculo.
TRANSLATION: *Well, what I see is that it goes around. It's a circle.*

MAN: Es un círculo.
TRANSLATION: *It's a circle.*

MODERATOR: Exacto.
TRANSLATION: *Exactly.*

MAN: Sale de la vaca, va al campo, del campo sale la pastura, la pastura para la vaca, y allí está.
TRANSLATION: *It comes out of the cow, goes to the field, pasture grows in the field, the pasture goes to the cows, and there you go.*

MAN: Sí. Pero en toda, no nada más en Wisconsin, en todos lados en Estados Unidos. No sé en otros países, peros eso es el sistema que tienen para reusar el . . .
TRANSLATION: *Yes. But all over, not just in Wisconsin, in every part of the United States. I don't know about other countries, but that is the system that they have to reuse the . . .*

MODERATOR: Exacto . . . muchas gracias.
TRANSLATION: *Exactly . . . thank you very much.*

MAN: ¿Eso sería todo?
TRANSLATION: *Is that it?*

MODERATOR: Sí. You . . .
TRANSLATION: *Yes. You . . .*

WOMAN: Yeah. I'm just going to stop the recordings.

Focus Group 3, Farm 2
Recording in Spanish (Translated to English)

MODERATOR: Okay. Yeah. So . . .

MAN: *Yeah, so I believe that, in all the jobs, a uniform is used, right? And, yeah, it's good. It's good to use a uniform.*

MODERATOR: *More comfortable.*

MAN: *Yeah.*

WOMAN: *Yeah.*

MODERATOR: *What else? And what are the practices, like handwashing, that you have here at work? Do they tell you, you have to wash your hands for this, for example?*

MAN: *Yes, every time a calf is born, you have to change all the, if you are milking, you're using gloves, you have to change. You change the tank, you have to wash your boots, change gloves, so they're clean, and change yourself so that, to not contaminate the milk at all. That's what we do.*

MODERATOR: *The same for milking?*

WOMAN: *Yes, as well.*

MODERATOR: *And how do you prepare to leave work? For example, when the day is done, and you have to go home, what's your routine like? How do you prepare?*

MAN: *Ending a shift?*

MODERATOR: Mm-hmm.

WOMAN: *Just, well, everything is left ready, and you go and change. You punch your card.*

MODERATOR: *Do you leave the clothes here?*

WOMAN: *Yes.*

MODERATOR: *The work clothes?*

WOMAN: *Yeah, because there is a washer and a dryer to wash, and the next shift, they put the clothes in to dry, and like that . . .*

MODERATOR: *You guys, when you arrive here, you have all the clothes here and you put . . .*

MAN: *Yes.*

WOMAN: *Yes, we change here.*

MODERATOR: *That?*

WOMAN: *Yes.*

MAN: *Yes.*

MODERATOR: *Okay. And, well, do you guys know how the raw milk is handled here, the milk recently taken from the cow, if they do something with it or if, I don't know, they drink it or something like that?*

MAN: No.

WOMAN: *Just the stuff from the seventh week to give to the calves. We don't use that milk for, if they don't send it over there, that milk would be, and if there is milk, good, if there isn't milk, then they send to the calves. It's the only one that is thrown or sent over there. Then, the rest, no, everything goes to the tanks.*

MODERATOR: Mm-hmm.

WOMAN*Yeah.*

MODERATOR: *And, for example, do you know anyone, or have you guys ever drunk like raw milk?*

WOMAN: Mm, no.

MAN: No.

WOMAN: No.

MODERATOR: *And, well, the last questions that I wanted to ask you guys is about use of antibiotics here on the farm. Well, antibiotics are the medications that they give to people, and animals also, to improve or to cure some bacterial infection, right?*

WOMAN: Mm-hmm.

MAN: Mm-hmm.

MODERATOR: *What is your experience taking an antibiotic? Have you guys ever taken an antibiotic?*

MAN: *Yes.*

WOMAN: *Mm, yes.*

MODERATOR: *Yes, and when, like was it for something specific or?*

WOMAN: *For a fever, yeah.*

MODERATOR: *Do you find them useful?*

MAN: *What?*

MODERATOR: *Do you find them useful, like that they work or don't work?*

MAN: *Yeah, sometimes.*

MODERATOR: *And, for example, if you guys have to take an antibiotic, where do you get it or where do you go when you need it?*

WOMAN: *The pharmacy.*

MODERATOR: *The pharmacy?*

WOMAN: *Yeah . . .*

MAN: *Yeah.*

MODERATOR: *Well, what happens is that sometimes antibiotics stop working, like there are bacteria that now it doesn't do anything to them, what is called they become resistant, in people and in animals. And that is what they are researching, why that happens and what things affect the bacteria to become resistant to the antibiotic and so then it doesn't work. So did you guys know this about all the antibiotic resistance? And what do you think could happen . . .*

WOMAN: Mm . . .

MAN: *Well . . .*

MODERATOR: *Did you know that?*

MAN: *. . . . . like, no.*

MODERATOR: *And what do you think about antibiotic resistance?*

MAN: *I think that, well, I believe that the bacteria, how would I say, they get used to it, they become stronger, that's why it doesn't work anymore.*

MODERATOR: *Exactly.*

WOMAN: *Yeah, what my colleagues were just commenting a while ago, that about how everything is injected in the cows, nothing you eat is natural anymore, instead it's already, for example, the meats you buy have been frozen a long time. And, well, I believe that that is harming us, and for that reason, we can no longer count on antibiotics. And, yeah, yeah.*

MODERATOR: *Mm-hmm. And, for example, have you guys ever had an infection that, resistant to an antibiotic, that the antibiotic didn't work?*

WOMAN: No, no.

MODERATOR: *No? Has something happened to you?*

MAN: *. . . . . no.*

MODERATOR: *No? That's good.*

MAN: *Yeah.*

MODERATOR: *And what risk do you think that this can make for you in the future or for other people?*

MAN: *Regarding . . . of antibiotics?*

MODERATOR: *Regarding antibiotic resistance.*

MAN: *Well, who knows? What can be done about it? It's something that's already very advanced, right?*

MODERATOR: *Mm-hmm, but do you think that it can be dangerous or that there is a risk?*

MAN: *Yes.*

WOMAN: *Yes, because I have seen that right now people die really sooner than before. It was very different. People lasted for many years, and now, no. There are people that are already very weak, now a lot of diseases for the same reason of the things we eat. It's not healthy anymore.*

MODERATOR: Mm-hmm.

WOMAN: *Yes, I think that it does have a risk later on in the future.*

MODERATOR: *And what do you think that, what role, or if the dairy farm plays any role in this antibiotic resistance, if you guys think it has to do with like some relationship that the dairy farm has with . . . antibiotic?*

WOMAN: *Mm, could be. Yeah, because a lot of, well, because of all that they put in the cows, because they put a lot of, what's it called, very strong medications that, yeah, I believe that that does also affect us, yeah.*

MODERATOR: *And what rules does the farm have about the use of antibiotics, for example, who can apply the antibiotic on the cow and who, do you know?*

WOMAN: *There are just two people.*

MAN: *Three including the owner.*

MODERATOR: *Okay. Just those two people can apply the antibiotic?*

MAN: *Yeah.*

WOMAN: *Yeah, and the other one so that the milk comes down, we do look after the . . . too like that. That we do take care of, well, very often, almost every day we look after that.*

MODERATOR: *And in the calves, you don't apply antibiotic?*

MAN: No.

MODERATOR: ¿No?

MAN: No.

MODERATOR: *And, well, and what do you think about this rule, like that there are only two people to do it, to apply the antibiotic?*

WOMAN: *Mm, I think it's okay because they are better prepared than us, and for me, it's fine, yeah.*

MAN: *Yeah, they are the ones that already have experience applying the antibiotics.*

WOMAN: *Yes, he's done, yes, they gave them training for that. Yeah.*

MODERATOR: *Well, those are like all the questions that we had. I don't know if you guys have something you want to ask . . . that you wanted to make. No?*

MAN: No.

MODERATOR: *All right, thank you very much for participating.*

Focus Group 4, Farm 3
Recording in English

WOMAN: All right. You're good.

MODERATOR 1: Okay. Thank you. All right. So like I said, the purpose is we really just want to learn more about how farms use antibiotics in, to treat animals and just different practices that might be related to antibiotic resistance. So I'll be asking questions related to that. My name is Nicole Bryce(?). I'm sorry I didn't introduce myself. But I am a researcher at UW, and I'm just helping out on this study. I think we, and then, yeah, just if, feel free to get up if you need to use the restroom or take an urgent call or whatever. That is completely fine.

So to get started, I'd like to learn a little bit about you guys and your roles on the farm. So if you guys wouldn't mind just sharing with me what your role on the farm is, how long you've been working here, and any other farming experience that you've had.

WOMAN 1: Well, I'm assistant herdsman. I take care of most of the shots here. We're basically hospital, treating cows, doing assistant nutrition for the relief feeder.

MAN: . . .

WOMAN 1: . . .

MODERATOR 1: For the what?

MAN: . . .

WOMAN 1: Relief feeder. So like you do cows every other weekend and stuff. I guess I really don't know, kind of everything, help with all the outside stuff when needed.

MAN: And you've been here how long?

WOMAN 2: Three and a half years, I think.

MODERATOR 1: Okay. Any previous farming experience?

WOMAN 2: Yes.

MODERATOR 1: Is this, okay.

WOMAN 2: The fourth or, I think the fourth farm, third or fourth farm.

MODERATOR 1: . . . okay. So how long total have you been working on farms?

WOMAN 2: I grew up farming.

MODERATOR 1: Okay. So . . .

WOMAN 2: But they haven't, my parents stopped milking in 2000, so I went to school for dairy herd management and worked on two other farms between going to school and coming here, basically milking before I came here.

MODERATOR 2: Okay. Great. Thank you.

WOMAN 2: I've been here, what, a year and something?

MAN: About a year and a half.

WOMAN 2: Yeah, about a year and a half. And I grew up on a farm too. My parents used to milk cows, goats. We raised pigs. We had our hands in about everything. I scrape pens, milk cows, help with fresh cows, help WOMAN 1 and MAN with herds work sometimes.

MODERATOR 1: And then so the next set of questions, I just want to learn a little bit more about the farm and how it works. So if you could just kind of tell me what a typical shift on this farm is like.

WOMAN 1: On the milking side or from both?

MODERATOR 1: I think both would be, what do you . . .

MODERATOR 2: Yeah, whatever your experience typically is.

MODERATOR 1: Yeah, whatever your experience is, like what, just kind of, and maybe your shifts are different . . .

WOMAN 1: They are different almost every day . . .

MODERATOR 1: . . . like . . . almost every day? Okay.

MAN: Yeah, they do two different things. So both of you can answer.

WOMAN 1: She, when she works the morning shift, she'll come in and milk in the morning. I . . .

WOMAN 2: Or scrape, wherever they put me.

WOMAN 1: Or scrape. I will come in typically and put in nights records from cabins and stuff like that, check on any sick cows that we may have from overnight, temp all the fresh cows, make sure they're doing okay, check baby calves, any newborns that might be fed or calves after that that need to be fed before I head up the hill, temp cows, help with the hospital pen. And that's usually comes to about 10:30, 11:00 in the morning. And then the mornings are different every day, whether we're doing herd health, foot trim, drag treating, routing cows, different every day. So I would, little bit different at the noon hour every day of the week.

MODERATOR 1: Do you have set breaks throughout the day, or do you . . .

WOMAN 1: Just kind of . . .

MODERATOR 1: . . .

WOMAN 1: . . . depends how busy the morning is and then afternoon and . . .

WOMAN 2: If you're on the milking team, you don't have no breaks. Sometimes you have to find the time to take a pee, to be honest.

MODERATOR 1: How long is a typical shift?

WOMAN 2: About eight and a half hours, eight hours.

MAN: Yeah, we milk three times a day, 4:00, noon, and 8:00 p.m. And they'll come in 15, 20 minutes before the start of that unit's on designation time, and, you know, they may end up being here 15, 20 minutes after. So a lot of times, they get eight and a half hours in a shift as a milker or the drover.

MODERATOR 1: And then what do you do if you have a question about how to do your job?

WOMAN 1: Talk to the upper management, whether it's her talking to me, and if I don't know, we both talk to him. And if he's not around, then we attempt to find the boss.

MAN: Phone, I get called all hours of the day and night, as herdsman and operations manager. So I get paid the big bucks for, is to answer the phone.

WOMAN 2: He tries to teach us simple things that people get stumped on so he doesn't have to come in, but sometimes it's . . .

WOMAN 1: Doesn't always work that way.

WOMAN 2: Yeah, sometimes we don't have the answers either.

WOMAN 1: A lot of times, we get called in for smaller things, whether it's they're behind or easier things to fix. Beyond that, then it's all him.

MODERATOR 1: Okay. What are the types of things that you tend to have to ask him for?

WOMAN 1: If we can't figure out why a cow is down, if we can't figure out why, if we need IV or if she's down for a certain reason.

WOMAN 2: Why the . . . grate don't work.

WOMAN 1: Sometimes we have mechanical things that . . . us just don't know exactly everything about, more or less that kind of stuff. If we have to plate a cow or something, it definitely takes more than two or three people, especially if it's us. It's two guys and, three guys, it's different.

WOMAN 2: But in the mornings, there's more people here, but in the afternoon shifts, there's only two people per shift. So if we have trouble . . .

WOMAN 1: More of the outside guys are gone for the day and stuff like that, so that's why . . .

MAN: Or doing field operations in the warm months, so.

WOMAN 1: I'm usually gone then. I'm usually outside . . .

WOMAN 2: So if we have trouble on those shifts in the afternoon or at night, like a cow is down, one person can't plate a cow, so you have to call somebody in to come help you.

MODERATOR 1: Gotcha. Okay.

WOMAN 2: Or trouble calving that you need help with or something.

MODERATOR 1: Okay. And then are there, what are the situations that he would come talk to you, or what, I'm just trying to get a sense of how you, do you have ongoing meetings, or are there certain . . .

WOMAN 2: Yeah, we have staff meetings regularly.

WOMAN 1: Probably once a month, try to be like about once a month.

MAN: Yeah, about month . . .

WOMAN 1: Unless things are just flowing just right, we just don't need one.

WOMAN 2: Or if, because I'm on the milking staff, and she does herd health, if . . .

WOMAN 1: I catch something . . .

WOMAN 2: . . . he's been gone or . . .

WOMAN 1: . . . when he's not around, and then I can tell her to communicate since she milks with them. So they kind of communicate between her to go back and forth to us to them sometimes, so.

MODERATOR 1: Makes sense. In between meetings, are there other types of topics that you'll, or updates that you'll give?

WOMAN 1: Yep. We have two different, well, we have a whiteboard out in the hallway, so a lot of times when there's stuff going on that needs to be passed on to somebody else, we always put notes up there for the Hispanics or for us or whatever.

MODERATOR 1: Sounds good. Do you eat meals on, during your shift?

WOMAN 1: Yeah. Well, definitely for our meetings and stuff. It's . . .

MAN: Yeah, generally speaking, they're not prohibited from eating food, but some people will bring it and, you know, may end up eating it eight hours later. It just, you know . . .

WOMAN 1: Some people eat right before they come to their shift and then go home and eat afterwards.

WOMAN 2: I would say the morning shift probably eats more when they're here than others . . .

MODERATOR 1: Others, okay.

WOMAN 2: . . . because they get here at 3:30 in the morning.

WOMAN 1: Nobody typically eats at that time of day.

MODERATOR 1: Yeah, I mean, you're probably pretty hungry at that point.

WOMAN 2: So usually when we bring down the second pen, there's, it's not quite as stressful outside. Outside person comes back in early, and they'll go eat while the other person is milking, and then we switch and . . .

MODERATOR 1: Okay. And where do people tend to eat?

WOMAN 1: Right here in the . . . room.

WOMAN 2: In here or in the barn. We eat in the parlor all the time too.

WOMAN 1: I mean, as far as . . . the barns and stuff like that that you can actually . . .

MODERATOR 1: It's easy to . . .

WOMAN 1: . . . keep . . .

MAN: It's more of a snacking environment. You know, generally speaking, the afternoon shift, because we start at noon, has eaten when they get here. So, you know, most people will go most of the shift with, and then eat when they get home at night. But doesn't mean that they don't grab a banana or an apple or a granola bar and eat in the parlor.

WOMAN 2: So then we wrap it in a towel, because our hands are dirty, and we eat at the cow towel. It's supposed to be clean.

MODERATOR 1: Okay. Sounds good. Okay. Now we're going to talk fashion. We're interested in learning about what rules your farm has in terms of what you wear.

WOMAN 1: Kind of depending on, really, what you're doing, but there is really no dress code. I mean, there, we have aprons for the milkers by choice if they want to wear them . . .

WOMAN 2: . . . gloves. We usually . . .

WOMAN 1: Rubber, we, I guess that is . . .

WOMAN 2: We all usually wear those . . .

MAN: Yeah, it's essentially expected. It's not a written rule that they wear milking gloves, but if some . . .

WOMAN 1: Unless you want your hands stained with iodine.

MAN: . . . if somebody was, yeah, if somebody was not wearing gloves, we would correct that and make them wear gloves. But it's not a written rule.

MODERATOR 1: Are there other unwritten expectations in terms of clothing?

MAN: As far as clothing, no.

WOMAN 1: Typically, everybody just wears their rubber gloves and their rubber boots.

MAN: Yeah, you know, it comes down to common sense. As long as they've got appropriate footwear, you know, we don't require safety toed boots. We just, I just monitor what, you know, if we have a new employee, that they come with somewhat appropriate footwear. And, you know, beyond that, it's kind of common sense applies.

MODERATOR 1: Okay. Any eye protection or facemasks in situations that you'd use those in?

WOMAN 1: Everybody pretty much has their own supply of that stuff. Like this time of year, everybody has got their facemasks on, stuff for going outside, but . . .

MAN: As far as eye protection, in the past, we've had milkers, by personal preference, you know, maybe wear safety glasses. But generally speaking, we have safety glasses around. We have hearing protection around. You know, most people tend not to utilize it.

WOMAN 1: It's definitely here for the option if they want it.

MODERATOR 1: Okay. It's provided.

WOMAN 1: Yeah.

WOMAN 2: It is, but I don't think anybody. . .

WOMAN 1: Nobody ever has problems with getting it. Like, I mean, the guys up in the shop, which doesn't have anything to do with this, they almost always wear that kind of stuff because they're . . .

MAN: . . .

WOMAN 1: . . . not with the cows or grinding stuff.

MODERATOR 1: Okay. And then do you wear anything special if you're working with calves or with a sick cattle, any differences . . .

WOMAN 1: Just mainly our rubber gloves.

MODERATOR 1: . . . just gloves, rubber gloves, okay.

WOMAN 1: Depending if we're pulling a cow, we usually put our breathing sleeves on, sanitation . . . stuff.

WOMAN 2: And I don't know if we really do that for us. It's more for the cow.

MODERATOR 1: Okay. Yeah, to keep them . . .

WOMAN 2: Yeah.

MODERATOR 1: . . . okay. What are your hand-washing practices at work?

WOMAN 2: Well, we wear rubber gloves, so I guess I don't . . .

WOMAN 1: They get changed plenty enough.

WOMAN 2: Yeah.

WOMAN 1: We go through a lot of gloves, so.

MODERATOR 1: Okay. So it's more rubber gloves to protect your hands and . . .

WOMAN 2: Yeah, like I'll wear two pairs, and then I'll, if I have to go to the bathroom or something, I'll take the top pair off, and my other pair is still clean, so, because it's hard to put rubber gloves back on sweaty hands. And if I double up my gloves, I found that they last longer because one pair will rip through really quickly, it seems, so.

WOMAN 1: I guess enough of us are in the milk house enough, washing stuff, washing pails, washing dump pails, washing something. We're in there enough to . . .

WOMAN 2: Heating up milk.

WOMAN 1: Yep, washing . . .

WOMAN 2: You're in the water lots, so.

MODERATOR 1: Okay. Have you been given any instructions about washing your hands from management or . . .

[Simultaneous discussion]

WOMAN 1: No, it's kind of a common sense rule, I guess.

MODERATOR 1: Common sense, yeah.

MAN: As a rule, you know, as part of training, we will comment about keeping their milker sleeves hosed off. And, you know, they've heard this speech enough about, we're making food, that they have to be conscientious of not contaminating and wiping off milkers and keeping their gloves changed or rinsed down, not that it's, there's a written policy. There's very few written things here because I find that . . .

WOMAN 1: Nobody wants to be dirty anyway.

MAN: I find that most the time, the ripped stuff gets water hosed and, or you print it off, and it ends up in the garbage. So I've just kind of, after 15 years of working with Hispanic employees, I've just gone away from written stuff, and we just kind of try to communicate orally the important points at staff meetings.

WOMAN 2: And I think too, like for me and Ashely, because we were basically born on the farm, it's just kind of a no-brainer. We just grew up that way, so we . . .

WOMAN 1: We usually correct anybody that's not doing something like that, but you don't find that around here really ever.

MODERATOR 1: Great. How do you get ready to leave work for the day?

WOMAN 2: Usually wash my boots off, take off my dirty sweatshirt, put on a clean one, take, if I have bibs on that are dirty, I take them off . . .

MODERATOR 1: Okay. And where do you . . .

WOMAN 2: . . . wash my hands, sometimes my face if I feel like I've gotten hit in the face with a dirty tail or whatever. But that's about it for me. I get really dirty, so, because I'm the milking staff.

WOMAN 1: Other than washing the boots here, mainly everything with us goes home.

MODERATOR 1: Okay, goes home. And is there a particular spot that you usually finish up for the day and clean the boots . . .

WOMAN 2: In the milk room usually.

WOMAN 1: Or right here . . .

WOMAN 2: In here.

WOMAN 1: . . . right when you come down the ramp before you come in, there's a drain, and there's a hose there. So when you're coming in here or you're going outside, you're coming in to the parlor, there's a hose there to come, to enter, to leave.

MAN: I just want to touch on it. Every milking staff member has a locker. They have the right to wash their clothes, their barn clothes here. So generally, everybody changes in and out at work time. They don't come to work . . .

WOMAN 1: Oh, right, yeah.

MAN: . . . they don't come to work in work clothes and then leave dirty in work clothes. They generally change clothes here . . .

WOMAN 2: The Hispanics mostly. Like for me, I, because I work two farms, so I take a lot of my clothes home because I need them, you know.

MAN: But most over the year, most of our Hispanic help is using the laundry facilities here and leaves clean.

MODERATOR 1: Okay . . . so now I'd like to learn more about raw milk and your experiences with that. How are raw milk and raw milk products handled on this farm?

WOMAN 2: Handled, like . . .

WOMAN 1: I guess . . .

WOMAN 2: . . . we don't.

MODERATOR 1: You don't? Okay.

WOMAN 2: It goes in the milk tank, and we don't do anything with it . . .

WOMAN 1: . . .

WOMAN 2: . . . except for the classroom when we milk fresh cow. But everything is clean. Milk the cow, we test the milk, put it in the refrigerator. We have a pasteurizer that we pasteurize it, and then we bottle it and feed it back to our babies. But other than raw milk and any other way, we don't really handle it.

WOMAN 1: We, after the cows are clean, the milker is gone, and the next person who looks at it basically, or tests it would be our milk caller(?), and it's gone. It's all enclosed.

MAN: Yeah, we have a very closed system here by the nature, as large as we are, and I don't think that's different than most larger diaries today, you know. Any raw milk that doesn't end up in the bulk tank for consumption goes down the drain from breaking connections, you know, pipe connections that they just aren't handling milk. It's either in the tank and is never touched by a, anybody, or it's down the drain.

WOMAN 2: Yeah, the only milk we have . . . fresh cow milk basically. It would be raw.

MODERATOR 1: Yeah. Are there any formal rules about it, or is it a more, about how you handle like, say, the colostrum . . .

WOMAN 2: Get it in the refrigerator as soon as possible because we don't want it growing bacteria.

WOMAN 1: Yep, typically, we just milk the cow into a pail, and it comes in and gets tested, goes in the fridge.

WOMAN 2: Like immediately, as soon as it comes from the cow, it goes in the fridge.

WOMAN 1: Then we turn around, and one of us usually pasteurizes it, gets it back in the fridge until babies are ready to be fed.

MODERATOR 1: Okay. Do you or any other farm workers ever consume raw milk or raw milk products?

WOMAN 1: We used to all the time when we were little.

WOMAN 2: Yes we did. But when our parents sold their cows, now we don't anymore.

WOMAN 1: Nobody here.

WOMAN 2: But we grew up on raw milk, both of us.

MAN: My mom was a pasteurizer person. We drank milk out of the tank, but we had a two-gallon pasteurizer. So I've never been one to drink raw milk. When I did dairy myself, as young adult, I drank raw milk. But since I've sold my cows 15, 20 years ago, I've not drank raw milk from this farm, so, yeah, we just, not that it's prohibited. You know, from time to time, we'll have an employee ask if they can take milk out of the tank to make cheese or whatever. But it's very rare.

MODERATOR 1: Okay. Now I want to focus more on antibiotics, and you're probably familiar with them. They're the penicillin, amoxicillin that you get when you have an ear infection, and they're basically used to treat illnesses that are caused by a bacteria. And as I'm sure you know,

veterinarians use them as well on animals. So what is your personal experience with taking antibiotics?

WOMAN 1: If you're sick, you take it, and it fixes you. Yeah, we're just trying to make the cows just as, treat the cows as if they were us.

MODERATOR 1: Okay. And in what situations have you found antibiotics helpful?

WOMAN 1: Here on the dairy or personally?

MODERATOR 1: I think personally. We'll, my next question will focus more on kind of how you use it on the animals.

WOMAN 1: So how does it work for us, is that what you're . . .

MODERATOR 1: Yeah, like and I mean you don't have to share but, I guess, personal health information, but are there, you know, when would you seek out an antibiotic, or what types of illnesses do you think are, not even personal to you, but would be, if you thought you had it, you might go to the doctor . . .

WOMAN 1: We typically . . .

WOMAN 2: Infection . . .

WOMAN 1: . . . yeah.

WOMAN 2: . . . of some sort.

WOMAN 1: Definitely an infection. We've always, we have never been ones to go to the doctor, so you have to be sick over a week before you get to go. So making sure that you . . .

WOMAN 2: And then we still don't want to go.

WOMAN 1: Yeah, more or less making sure it's something that's going to be, not going to just pass on its own.

MODERATOR 1: Okay. And how do you usually go about getting antibiotics for yourself?

WOMAN 2: The doctor. It will prescribe if we have to go to the doctor.

WOMAN 1: Yep, prescription medicine.

MODERATOR 1: Okay. And what do you think about antibiotics just in general?

WOMAN 2: They typically work when you take them . . . supposed to, how you're supposed to, as long as you're supposed to.

MODERATOR 1: Okay. And then I talked earlier about antibiotic resistance of how they stop sometimes, with more use, stop being effective, or the bacteria becomes resistant to the antibiotic, and it no longer works to treat the infection. And it can affect both humans and animals. What are

your thoughts on antibiotics or antibiotic resistance in terms of, you know, what have you heard about it, if anything?

WOMAN 2: That doctors overprescribe antibiotics a lot to people that, like if they would've waited a week, not been to the doctor, they would've been fine. But, I don't know, I think some people reuse it in animals too sometimes, depending on the situation or where you're at in a farm.

WOMAN 1: Depending on the type of antibiotic, what you're trying to get to, the cause, the problem.

MODERATOR 1: Yeah. And you've both worked on multiple farms. Have you noticed differences between the farms on how they use antibiotics?

WOMAN 2: I have.

MODERATOR 1: Okay. How so?

WOMAN 2: Like here, it don't seem like we use a lot of antibiotics at all.

WOMAN 1: No.

WOMAN 2: We don't even . . . treat our cows with antibiotics . . .

WOMAN 1: We . . .

WOMAN 2: . . . most of them.

WOMAN 1: . . . try to self-care everything, you know, your aspirin . . .

WOMAN 2: Something . . .

WOMAN 1: . . . if they don't feel good, just try to help them . . .

WOMAN 2: Udder mint for . . .

MAN: Supportive care.

WOMAN 2: Supportive care.

WOMAN 1: Yes.

WOMAN 2: Try to let them heal themselves. But a lot of farmers . . .

WOMAN 1: . . .

WOMAN 2: . . . they see a cow with mastitis, tubes right now. They don't try to let them heal themselves.

MODERATOR 1: Okay. So it sounds like here it's more of a, like the antibiotics are more of a later resort. You try other things first.

WOMAN 1: Yes.

WOMAN 2: Yes.

MODERATOR 1: Okay, great. What role, if any, do you think dairy farms play in antibiotic resistance?

WOMAN 1: I guess I don't know what you mean.

WOMAN 2: She means by working at a dairy farm or by a farm using antibiotics . . .

MODERATOR 1: Yeah, does it affect whether bacteria become resistant . . .

WOMAN 2: Resistant and not . . .

MODERATOR 1: . . . or what is the effect on . . .

WOMAN 1: I guess the more you use them, the more, less prone they're going to help themselves. I guess kind of like if you get used to using ibuprofen all the time, I guess, you know, your body just gets immune to it if you're going to treat something all the time, and it's get immune to getting the TLC.

MAN: Okay. So that's your perception, in theory, of what could happen. Do you have any personal experiences from working on your other farms where you have reference that, oh, they use drugs a lot, and then they weren't able to fix a cow using treatments?

WOMAN 2: Oh, yeah. I've used, I've been told to use penicillin on a mastitis cow a lot, and she'll never get better, and we end up selling her in the end anyways, or she dies or whatever. So there was no point in using it, you know. But that's always seems to be the answer for some people, especially when it comes to mastitis, I find.

MODERATOR 1: That's kind of like right, some farms go right to the antibiotics and . . .

WOMAN 2: Yeah.

MODERATOR 1: How do you treat, or what, I'm not super familiar with dairy farms, but what are the alternative treatments that . . .

MAN: Did you girls come in . . .

MODERATOR 1: . . . you use . . .

MAN: . . . a black pickup truck?

MODERATOR 1: No.

WOMAN 2: Alternative, we do fluids.

MAN: Milk hand is here, and people are in the way out here.

WOMAN 2: We help with . . . fluids, which has what in it? Has . . .

WOMAN 1: Or definitely has our calcium. It has dry hay, dry alfalfa . . . meal in it.

WOMAN 2: It has a, for salmonella.

WOMAN 1: Yep, it has protection for that in it. There's propylene glycol, which just helps the whole body.

WOMAN 2: It's like a sugar boost when they don't eat.

WOMAN 1: Yeah, if they don't feel good or if they just freshen or whatever, they need a little more energy, all that mixed in with a five-gallon pail of water. And if they look like they've just freshened and they need a little bit of TLC or they're kind of slowly going downhill, we use it as a, we give them almost . . .

[Simultaneous discussion]

WOMAN 1: . . . every, that's our first go-to, pretty much.

WOMAN 2: So we pump it.

[Simultaneous discussion]

MODERATOR 1: Okay. Yep. Okay.

WOMAN 2: But if they don't feel like eating, then that's their nutrition.

WOMAN 1: That's their go-to.

WOMAN 2: That's how we keep them going.

WOMAN 1: Just like here at the hospital when people aren't eating, they just IVed. So that's our . . .

WOMAN 2: And we use a lot of liniment, like . . .

WOMAN 1: Liniment . . .

WOMAN 2: . . . on mastitis.

WOMAN 1: . . . for blood flow, circulation.

WOMAN 2: Helps reduce inflammation, which is kind of like Icy Hot for cows.

WOMAN 1: It definitely is.

MODERATOR 1: Yeah. And so I asked earlier what role you think farms play in antibiotic resistance . . . you see. How do you think dairy farms like this one are affected by antibiotic resistance, if at all? Has it . . .

WOMAN 2: I don't really know we've been affected by it. MAN, do you have any thoughts?

MAN: Give your, answer the question, and I'll chime in. Give your perspective because they want to collect, you know, opinions.

WOMAN 1: I don't know, personally.

MODERATOR 1: Don't, okay. That's fine.

WOMAN 2: At this farm, I don't really, I don't think we've had too much trouble with . . . because we don't use as much antibiotics, I don't feel.

WOMAN 1: We don't have problems with antibiotic. If we, we don't use it, so when we do use it because we need to use it, because it's something that's needs it, it usually cures it, and it's, we don't see the problem.

MODERATOR 1: Yeah. Do you feel like there's any relationship if there's farms nearby that use a lot of antibiotics? Have you heard, I'm, or does it, do other farms that do use a lot of antibiotics, you feel like they impact your work and, at all?

WOMAN 1: I don't think so.

MAN: My perspective on it is the animal industry as a whole has gotten a bad rep, and unfortunately, perception is reality. And veterinary feed directive got put into place two years ago because of that perception and the need to control our drug use, and it ultimately has taken drugs away from us. You know, there's been drugs that they're trying to set aside for human-only type use, and they're definitely, our veterinarians are definitely encouraging us to not use stuff off label anymore, if this drug was not intended for that.

And I was like, well, we used to use it for that. And it's like, well, we, I know, they admit, the vets admit that, but we're not supposed to anymore because that was an extra-label use, and now it's off label because somebody changed the rules. And we have less drugs that are accessible to us. So from a certain standpoint, it definitely has affected us as animal livestock producers, but is it good, is it bad? Our perspective here in managing cattle, it isn't really affecting us, but for somebody else, it maybe does.

WOMAN 2: Like I could, at my own farm, we used to raise cattle. We used to raise big cats, steers, Holstein steers. There were certain drugs that I used for other things other than they were labeled for that I wasn't supposed to. But it worked, so you used it because you can't afford to lose the animal. You know what I, I'll admit to that. I used to do that. But I didn't give it to them unless I knew that that was what they needed. Does that make sense?

MODERATOR 1: Right. You weren't just like, oh, they look a little sick. Let's see if this works.

WOMAN 2: Right, yeah. They're, when they get treated, they're sick.

MODERATOR 1: Yeah, okay. Makes sense. Are there formal rules on this farm about antibiotic use?

WOMAN 2: Formal rules as in . . .

WOMAN 1: Nothing really gets . . .

WOMAN 2: There's . . .

WOMAN 1: . . . used unless it goes through higher management. Nothing, they don't, actually, they don't, the milking staff doesn't use anything unless it's written down for them to use, unless they are told that it's, that's even with supportive care.

WOMAN 2: But we hardly, I think I could count on one hand the number of actual antibiotic-treated cows that we've had here since I've started working here. Maybe, I bet it was less than five that we had to dump.

MAN: Yeah milk withhold. We use . . .

WOMAN 2: . . . withhold, right.

MAN: . . . septiaflor(?), Dixie(?), you know . . . product line with no milk holds. But generally speaking, we don't use antibiotics that require a milk withhold. So, you know, WOMAN 2 is not exaggerating. We don't treat many cows . . .

WOMAN 2: For the safety of that whole milk tank in there, in case somebody messes up and actually puts a hot cow in the tank, because we work with a lot of people here, and they don't understand that a lot. They're new to this. They're not new. How should I say that? They have no experience, so they don't understand.

WOMAN 1: We're all humans, and accidents can happen, so we . . . prevent it.

MAN: Yeah, we just try to avoid . . .

WOMAN 2: We try to avoid it.

WOMAN 1: If it's not there to use, and if the cow is not treated, then they can't have the chance of . . .

WOMAN 2: Getting it in the tank.

WOMAN 1: Yep.

MODERATOR 1: Getting it in the tank. And it sounds like all those decisions are made higher up. It's not people making . . .

WOMAN 2: No, I don't even make that decision. Mostly it's him.

MAN: The, right. The milking staff has access to oxitose(?) and has been instructed through training and staff meetings to how to use oxitose. But generally speaking, that is the only drug of any form that our milking staff just freely uses.

WOMAN 2: Unless you're on a herdsman position, then they . . .

MAN: Yeah. No, we have a hospital clipboard that, you know, prescribes medicines and supportive cares for the day, and then that data ends up into the computer, which is kind of what I'm working at here, is making sure that all critical stuff is in.

WOMAN 1: But that's an everyday thing, and you can look back on any cow that has been treated in the last month typically.

MODERATOR 1: Okay. And what positions on the farm administer antibiotics after they've been given permission?

WOMAN 1: Antibiotics?

MODERATOR 1: Yeah, to the animals.

WOMAN 2: Us three, mostly. The morning milking staff ,if somebody is not here, and if it's on the clipboard, and if they know what it is, they will, somebody that's been here long enough knows what it is, so they will do it because it's on their list. But if, a lot of times, they come to one of us.

WOMAN 1: Typically, if somebody that doesn't know how to give the medicine is in milking, one of us is there too. So nobody is ever around it that doesn't know how to use it.

MAN: Yeah. If the Hispanic laborers can't understand what's written on the clipboard, and they don't know what it is, they don't give anything. They, you know, if they aren't trained and instructed that this bottle and this syringe, we don't cross syringes and drugs that, you know . . .

WOMAN 1: Everything is labeled.

MAN: . . . if they're, everything has on written procedures and expectations. But for the most part . . .

WOMAN 2: If they don't know, they'll come to one of us, WOMAN 2ny, and they'll point at it, and I already know that they don't understand, so then I'll just do it.

WOMAN 1: We have a group of milkers that don't ever touch medicine. They are more, they're cleaning up. They're doing that kind of stuff. And we have the group of milkers that usually take care of, they understand the procedures of medicine.

MAN: By virtue of more experience.

MODERATOR 1: . . . and then what do you think about how antibiotics are used on this farm . . . policies for the, informal policies that are in place and how . . . works?

WOMAN 1: I guess in terms of . . .

MODERATOR 1: In terms of, and maybe you have no opinion either, but like does it make sense to you as how the process works? Is there something that you think could be changed or, to make it work better or . . .

WOMAN 1: I think what we do here works. If it's not broke, you don't fix it. We don't try to change things. If it's not working, we try to make things so they do work, but . . .

But then if we change something, it's brought up at our staff meetings, so everybody knows on the same page. Everybody knows what's happening.

WOMAN 1: Yep. And if our changes don't seem to work as good as the old way, then we go back to the old way.

MODERATOR 1: . . .

WOMAN 2: We try to keep, because there's Hispanics and there's non-Hispanics, we try to keep . . .

WOMAN 1: Things easy. We don't try to change things much.

WOMAN 2: We don't try to break the, what am I trying to say?

WOMAN 1: We got a rhythm here, and we try to . . .

WOMAN 2: We have a rhythm. We try to keep it the same way all the time, or else that's when things get messed up.

MODERATOR 1: Yeah . . .

WOMAN 1: Everybody stays on the same page that way.

MODERATOR 1: Sounds good. And how about you, do you have any thoughts about antibiotic usage here that you wanted to add, or?

MAN: Not specifically. I think they've kind of, the girls have expressed our attitude, you know. I don't, I'm not anti-antibiotics. I'm not organic and a natural freak. I just think that the perception of people out there is such that I want to rise, or raise our level of management to a point where actually herdsman skills and animal husbandry and supportive care helps these animals get through their challenges without having to resort to drug use. You know, if you give an animal supportive care, a lot of things that are, our bodies are amazing designs. You know, we can cure a lot of things given a little time and supportive care.

And that's our approach, you know. If we treat cows and have to discard milk, there, that's a economic stressor. You know, we try to, we're in the business to make saleable milk and feed people. You know, I don't want to create non-saleable milk by overusing drugs.

WOMAN 2: But I would add too that I worked on a organic farm before, and there's a lot of things they would not, they can still sell a cow in a conventional MANet if they had to treat an organic cow. But because they're an organic farm, they wouldn't allow the drugs on the farm period because they were too scared it would get in the tank, which is normal for any dairy, but because they were organic, it's a really big no-no. So I've watched cows' feet fall off from foot rot because they're not being able to be treated with antibiotics. So I'm not very . . .

WOMAN 1: Cows just dying because they do not get the help that they . . .

WOMAN 2: They didn't get the care that they needed that an antibiotic could have took care of, and it was just . . .

WOMAN 1: The supportive care was just not enough, and they needed the antibiotic.

WOMAN 2: Yeah, it was . . .

WOMAN 1: Just like a, one of us, if you need it, and it's going to fix you better than all the other way.

WOMAN 2: So I guess it would come down to people knowing what they're doing too. You have to know what you're doing, what the drug is for, what you're treating it, using to treat it for. Does that make sense? So I've been on the flip side of things too. So antibiotics are a good thing too. But they can be abused too, I think.

MODERATOR 1: Mm-hmm. That makes sense. All right. That's about all the questions I had. Is there anything else that I didn't ask about that you feel like it's important for me to know?

WOMAN 1: I don't know. I don't think so. Just basically that people don't understand that antibiotics are an okay thing to use. It's no different than us using them on cows or people using them themselves. There's people that will abuse it for themselves, and there's people that will abuse it for the animals. And without giving it to the animals, they're no different than us. You got to give it when it's needed and not give it when it's not needed.

MODERATOR 1: Well, thank you so much for your time today. We really appreciate this. This helps us a lot to kind of just learn about farms and risks. So thank you for taking the time.

WOMAN 1: Thank you.

WOMAN: You're welcome.

WOMAN: . . .

MODERATOR 1: I'll turn this off.

Focus Group 5, Farm 3
Recording in Spanish (Translated to English)

WOMAN: Treinta y siete . . .
TRANSLATION: *Thirty-seven . . .*

MAN: Treinta y ocho.
TRANSLATION: *Thirty-eight.*

WOMAN: Treinta y nueve.
TRANSLATION: *Thirty-nine.*

MODERATOR: Okay. ¿Y cómo describe su raza?
TRANSLATION: *Okay. And how do you describe your race?*

MAN: Hispano.
TRANSLATION: *Hispanic.*

MAN: Latino.

MAN: Latino.

MODERATOR: Igual. ¿Y tu étnica, también latino?
TRANSLATION: *Same. And your ethnic, also Latino?*

MAN: Sí.
TRANSLATION: *Yes.*

MODERATOR: Yo nunca sé si, con mi raza y étnica, yo siempre digo latino.
TRANSLATION: *I never know if, with my race and ethnic, I always say Latino.*

MAN: Sí, latinos incluye a centroamericanos . . .
TRANSLATION: *Yeah, Latinos include Central Americans . . .*

MODERATOR: Sí, es que nada más nunca sé qué decir cuando me preguntan por mi raza y mi étnica, para mi, lo mismo, soy una . . .
TRANSLATION: *Yeah, is just that I never know what to say when they ask me for my race and my ethnic, for me, the same, I'm a . . .*

MAN: Sí.
TRANSLATION: *Yeah.*

MAN: Sí.
TRANSLATION: *Yeah.*

MAN: Porque . . .
TRANSLATION: *Because . . .*

MODERATOR: . . . siempre me digo latino, no sé.
TRANSLATION: *. . . I always say Latino, I don't know.*

MAN: . . . étnico es cuando hay algunos dialectos, ¿no?
TRANSLATION: *. . . ethnic is when there are different dialects, right?*

MODERATOR: Sí, sí, pero yo siempre digo latino para los dos. Okay, ¿cuánto tiempo ha estado trabajando aquí?
TRANSLATION: *Yeah, yeah, but I always say Latino for both. Okay, how long have you been working here?*

MAN: En esta granja?
TRANSLATION: *On this farm?*

MODERATOR: Sí.
TRANSLATION: *Yes.*

MAN: Ella y yo, 11 meses.
TRANSLATION: *Her and I, 11 months.*

MAN: Sí, yo soy de cuatro años.
TRANSLATION: *Yeah, I've been here four years.*

WOMAN: Año y medio.
TRANSLATION: *A year and a half.*

MODERATOR: Okay. ¿Qué tipo de trabajo hace en tu día típico?
TRANSLATION: *Okay. What type of work do you do in a typical day?*

MAN: Tu trabajo cotidiano.
TRANSLATION: *Your daily work.*

WOMAN: Ordeñar.
TRANSLATION: *Milking.*

MODERATOR: ¿Mande?
TRANSLATION: *Excuse me?*

WOMAN: Ordeñar.
TRANSLATION: *Milking.*

MODERATOR: Ordeñar, ¿qué es eso?
TRANSLATION: *Milking, what is that?*

WOMAN: O sea . . .
TRANSLATION: *I mean . . .*

MAN: Ordeñar vacas.
TRANSLATION: *Milking cows.*

MODERATOR: Oh, oh, oh. Okay.

MAN: Sí, ordeñar es el trabajo.

TRANSLATION: *Yeah, milking is the job.*

MODERATOR: ¿Y todos hacen lo mismo?
TRANSLATION: *And do you all do the same thing?*

MAN: Sí, nosotros, diferentes . . .
TRANSLATION: *Yeah, we, different . . .*

MAN: Nosotros hacemos diferentes trabajos porque somos los que vamos a traer las vacas a los corrales, limpiamos los corrales, limpiamos las camas, atendemos las vacas de parto, atendemos los becerros cuando nacen, les damos medicina a las vacas cuando están en una lista allí que hay que darle x medicina a tal vaca. Bueno, a nosotros los que corraleamos, los que nos toca hacer todo el trabajo que se hace allí afuera como . . .
TRANSLATION: *We do different jobs because we are the ones that bring the cows to the corrals, we clean the corrals, clean the beds, take care of the cows giving birth, take care of the calves when they're born, we give the cows medicine when they are on a list there that you have to give x medicine to such and such cow. Well, us who work in the corrals, we have to do all the work that's done outside like . . .*

MAN: Limpiar los establos, establo donde están las vacas, donde ellas se acuestan . . . después de eso, ordeñar, si hay que dar medicina de acuerdo lo que ordena el mánager, pone en una libreta lo que hay que dar la vaca, y nosotros encargamos de eso. Y lo que nosotros no podemos hacer, pues, él lo termina de hacer.
TRANSLATION: *Clean the stables, stable where the cows are, where they lay down . . . after that, milking, if we have to give medicine according to what the manager orders, he puts it in a little book what you have to give the cow, and we're in charge of that. And whatever we can't do, well, he finishes doing it.*

MODERATOR: ¿Y ustedes dos hacen eso, nada más?
TRANSLATION: *And do just you two do that?*

MAN: Sí, porque somos . . .
TRANSLATION: *Yes, because we are . . .*

MAN: Sí, nosotros los dos, y por el momento, nosotros los dos, allí la muchacha norteamericana que se llama Jenny, hace lo que nosotros hacemos y otro señor que se llama Henry también hace eso, pero, bueno, no sé por que no están ellos acá, pero por ahorita, somos cuatro los que hacemos ese trabajo.
TRANSLATION: *Yeah, us two, and for now, us two, there is an American lady named Jenny, she does what we do, and another man named Henry also does that, but, well, I don’t know why they aren't here, but for now, us four do that job.*

MODERATOR: Okay. ¿Cuánto tiempo ha estado haciendo este tipo de trabajo en esta granja?
TRANSLATION: *Okay. How long have you been doing this type of work on this farm?*

MAN: Desde que entré . . .
TRANSLATION: *Since I entered . . .*

MODERATOR: Son los 11 meses. ¿Y los cuatro años, lo mismo?
TRANSLATION: *Those 11 months. And four years, the same?*

MAN: Sí. Sí, sí, sí.
TRANSLATION: *Yeah. Yeah, yeah, yeah.*

MODERATOR: Un año haciendo lo mismo también?
TRANSLATION: *A year doing the same thing also?*

WOMAN: Uh-huh, año y medio.
TRANSLATION: *Uh-huh, a year and a half.*

MAN: Sí, ordeñan. Ellas dos se encargan lo que es la parla de ordeñar.
TRANSLATION: *Yes, they milk. Those two are in charge of the milking parlor.*

MODERATOR: Okay. ¿Han trabajado en otro lecheras?
TRANSLATION: *Okay. Have you worked on other dairy farms?*

MAN: Sí.
TRANSLATION: *Yes.*

WOMAN: Sí.
TRANSLATION: *Yes.*

MODERATOR: ¿Sí? ¿Dónde, aquí en Wisconsin?
TRANSLATION: *Yeah? Where, here in Wisconsin?*

MAN: En Wisconsin, en Iowa y en Minnesota hemos trabajado nosotros.
TRANSLATION: *We've worked in Wisconsin, in Iowa, and in Minnesota.*

WOMAN: Ah, todo el Midwest. ¿Y han trabajado en otra lechera?
TRANSLATION: *Ah, the whole Midwest. And have you worked on another dairy farm?*

WOMAN: Yo no.
TRANSLATION: *I haven't.*

MAN: Yo, anteriormente, sí, pero ahora de que regresé a este país, solo aquí he venido a trabajar. Aquí me he quedado trabajando para ellos.
TRANSLATION: *I, before, yes, but now that I returned to this country, I've just come here to work. I've stayed here working for them.*

MODERATOR: Okay. ¿Y cuánto tiempo estuvieron en las otras lecheras?
TRANSLATION: *Okay. And how long were you on those other dairy farms?*

MAN: Yo estoy trabajando en granja lechera desde el 2011. Tengo ocho años trabajando, y ella tiene siete años . . .
TRANSLATION: *I've been working on dairy farms since 2011. I've been working for eight years, and she's been working for seven years . . .*

MODERATOR: Oh, wow. ¿Y cuatro años?
TRANSLATION: *Oh, wow. And four years?*

MAN: Sí.

TRANSLATION: *Yes.*

MODERATOR: ¿Antes de los cuatro años, ha trabajado?
TRANSLATION: *Have you worked before those four years?*

MAN: Sí, estuve anteriormente acá y trabajé tres años en otro, sí. Pero ahora que regresé, solo acá . . .
TRANSLATION: *Yes, I was here before, and I worked three years on another one, yeah. But now that I came back, just here . . .*

MODERATOR: Okay. ¿Y primera vez en una lechera?
TRANSLATION: *Okay. And first time on a dairy farm?*

WOMAN: Mm-hmm.

MODERATOR: Okay. ¿Alguna vez te sientes apresurado cuando estás haciendo todo el trabajo que necesitas terminar en un día?
TRANSLATION: *Okay. Have you ever felt pressured when you're doing all the work that you need to finish in a day?*

MAN: Depende, a veces uno se siente como apresurado porque de acuerdo a la cantidad de trabajo que haiga. No todos los tiempos igual. Hay días de que está bien, se trabaja cómodo, hay días que uno anda apresurado. Depende . . .
TRANSLATION: *Depends, sometimes you feel like pressured because of the amount of work there is. It's not always the same. There are days that it's fine, you work comfortably, there are days that you are pressured. It depends . . .*

MODERATOR: Mm-hmm, nada más depende del día.
TRANSLATION: *Mm-hmm, it just depends on the day.*

MAN: Sí, depende. Por lo menos, ahorita, en el tiempo de hielo, uno siente muy presionado porque hay demasiado trabajo y hay que anivelar los turnos, ¿me entiende? Porque uno se pone de acuerdo de que el compañero de trabajo, uno mismo como trabajador, uno hace consciencia. Uno dice, pues, uno no quiere que acompañar, siente también presionado como uno. Entonces uno trata de que todos, me entiende, tienen que echarle ganas para . . .
TRANSLATION: *Yeah, it depends. At least, right now, in wintertime, you feel very pressured because there is a lot of work and you have to even out the shifts, you know. Because you are in agreement that a coworker, you as a worker, you have to be conscientious. You say, well, you don't want to accompany, you also feel pressured. So you try to make sure everyone, you know, they have to put in the effort to . . .*

MODERATOR: Sí.
TRANSLATION: *Yeah.*

MAN: Más el frío. Pues, sobre todo, el frío, lo hace a uno, lo presiona, ¿no?
TRANSLATION: *Also the cold. Well, above all else, the cold, it makes you, it pushes you, you know.*

MODERATOR: Sí. No me gusta el frío. Soy de Los Ángeles, y no estoy acostumbrada. Me estoy muriendo. ¿Y cuál es tu parte favorita del trabajo?

TRANSLATION: *Yeah. I don't like the cold. I'm from Los Angeles, and I'm not accustomed. I'm dying. And what is your favorite part of the job?*

MAN: Para mi, mi parte favorita, a mi me gusta todo. Me gusta trabajar afuera, me gusta, o sea, en mi caso de que soy, trabajo fuera y adentro, los dos, me siento bien porque hay como un cambio de rutina. Estoy adentro, es diferente que andar afuera, entonces . . .
TRANSLATION: *For me, my favorite part, I like everything. I like to work outside, I like, I mean, in my case that I am, I work outside and inside, both, I feel good because there's like a change in routine. I'm inside, it's different than being outside, so . . .*

MODERATOR: So no se aburre.
TRANSLATION: *So you don't get bored.*

MAN: Sí, para mi, es igual. O sea, la parte que me gusta, trabajar en esto de la granja, me gusta mucho.
TRANSLATION: *Yeah, for me, it's the same. I mean, the part that I like, working on the farm, I like it a lot.*

MODERATOR: Good. ¿Alguien más, otra parte favorita?
TRANSLATION: *Good. Anyone else, another favorite part?*

MAN: Yo no tengo preferencia. Donde me toque trabajar, para mi, es igual. Me siento preparado para trabajar en el área que me toca.
TRANSLATION: *I don't have a preference. Wherever I have to work, for me, it's the same. I feel prepared to work in the area that I have to.*

MODERATOR: Ah, awesome. Okay. So ahora le voy a hacer preguntas, a ver, okay, so le voy a hacer preguntas de cómo trabaja la granja aquí. ¿So recibes muchas direcciones sobre cómo hacer tu trabajo, y quién les da las direcciones?
TRANSLATION: *Ah, awesome. Okay. So now I'm going to ask you questions, let's see, okay, so I'm going to ask you questions about how the farm works here. So do you receive many directions about how to do your job, and who gives you the directions?*

MAN: El mánager.
TRANSLATION: *The manager.*

MAN: El mánager es el que . . . o sea, el mánager es como, cómo dijera, no es quien nos paga, pero es quien está siempre frente a nosotros, dando los ordenes . . .
TRANSLATION: *The manager is the one . . . I mean, the manager is like, how would you say, he's not the one that pays us, but he's the one that's always in front of us, giving orders . . .*

MODERATOR: Mm-hmm, ¿el mánager era el que estaba aquí?
TRANSLATION: *Mm-hmm, the manager was the one that was here?*

MAN: Sí, él es el encargado para nosotros como . . .
TRANSLATION: *Yes, he's the one in charge of us like . . .*

MODERATOR: ¿No es el dueño?
TRANSLATION: *He's not the owner?*

MAN: No.

MAN: No, el dueño es el encargado de él. Él encarga el personal.
TRANSLATION: *No, the owner is the one in charge of him. He's in charge of the staff.*

MAN: El señor Mark es el mánager, y el dueño de la granja, el señor John.
TRANSLATION: *Mr. Mark is the manager, and the owner of the farm, Mr. John.*

MAN: John es el dueño de . . .
TRANSLATION: *John is the owner of . . .*

MODERATOR: Y les da muchas direcciones o les dejan hacer lo que . . .
TRANSLATION: *And do they give you a lot of directions or do they let you do what . . .*

MAN: Cuando, eso depende. Cuando uno ya ha agarrado el ritmo de trabajo, le da confianza a uno. O sea, eso depende siempre, como en todos lados, me entiendes, que uno tiene que ganarse la confianza al patrón. Él mira de que hace un buen trabajo, entonces no lo anda apresurando. Ya sabe lo que va a hacer. Ahora, si una persona nueva, sí, él tiene que . . .
TRANSLATION: *When, that depends. When you've got the hang of the pace of work, he trusts you. I mean, that always depends, like everywhere, you know, that you have to earn the trust of the boss. He sees that you're doing a good job, so he's not on top of you. You already know what you're going to do. Now, if a person is new, yeah, he has to . . .*

MODERATOR: Un poquito más sabría de ellos.
TRANSLATION: *He would know a little bit more about them.*

MAN: Sí, claro, un poquito más estricto porque a todo encargado de un mánager, o sea, siendo un mánager, va encargado, el deber de él es mantener, tratar de hacer esa persona, entiende, que la persona haga bien en el trabajo porque es el trabajo de él también, me entiende, pues . . .
TRANSLATION: *Yes, of course, a little stricter because everything a manager is in charge of, I mean, being a manager, you're in charge of, his duty is to maintain, to try to make that person, you know, that the person does a good job because that's his work as well, you know, so . . .*

MODERATOR: Yeah. Okay. ¿Cuántas personas se considera su jefe? ¿Nada más él, el único patrón que está aquí, el mánager?
TRANSLATION: *Yeah. Okay. How many people do you consider to be your boss? Just him, the only boss that's here, the manager?*

MAN: Sí, es el único . . .
TRANSLATION: *Yes, he's the only one . . .*

MODERATOR: El único. Okay.
TRANSLATION: *The only one. Okay.*

MAN: Aparte de que nosotros respetamos a los dos a uno porque . . . porque se mantiene más con nosotros él.
TRANSLATION: *Apart from us respecting each other because . . . because he's around us more.*

MODERATOR: Okay. ¿Y cómo se comunican con él? Porque veo que no habla mucho español, ¿so cómo hacen eso?

TRANSLATION: *Okay. And how do you communicate with him? Because I see that he doesn't speak much Spanish, so how do you do that?*

MAN: No habla, pero entiende bastante. Él entiende mucho español.
TRANSLATION: *He doesn't speak it, but he understands a lot. He understands a lot of Spanish.*

MAN: Cuando yo necesito hacerle alguna pregunta, cualquier cosa, yo le mando mensajes de texto con mi teléfono con un traductor de Google, y así nos entendemos . . .
TRANSLATION: *When I need to ask him a question, anything, I send him text messages with my phone with a Google translator, and that's how we understand each other . . .*

MODERATOR: Okay. ¿Y allí tienen a alguien que tú puedes preguntarle preguntas si tienen preguntas?
TRANSLATION: *Okay. And do you have someone there that you can ask questions of if you have questions?*

MAN: ¿Como qué?
TRANSLATION: *Like what?*

MODERATOR: ¿Hay alguien que, si ustedes tienen una pregunta, alguien que pueden ir a preguntarle?
TRANSLATION: *Is there someone that, if you have a question, someone that you can go ask?*

MAN: ¿A alguien? No.
TRANSLATION: *Someone? No.*

MAN: Pues, todos estamos bien claros del trabajo que hacemos.
TRANSLATION: *Well, we are all clear on the job that we do.*

WOMAN: Sí.
TRANSLATION: *Yeah.*

MAN: Todos entendemos lo que hacemos. No necesitamos andarle preguntando a nadie porque . . .
TRANSLATION: *We all understand what we do. We don't need to go around asking anybody because . . .*

MODERATOR: Okay.

WOMAN: Ya sabemos lo que tenemos que hacer y cada quien lo que tiene que hacer y ya . . . andar preguntando.
TRANSLATION: *We already know what we have to do, and everyone what they have to do, and that's it . . . go around asking.*

MODERATOR: ¿Están cómodos hablando inglés en el trabajo?
TRANSLATION: *Are you comfortable speaking English on the job?*

MAN: ¿Dígame?
TRANSLATION: *Excuse me?*

MODERATOR: ¿Ustedes están cómodos hablando inglés en el trabajo?
TRANSLATION: *Are you comfortable speaking English on the job?*

MAN: No le comprendo.
TRANSLATION: *I don't understand you.*

MODERATOR: ¿Si están cómodos hablando en inglés ustedes?
TRANSLATION: *If you guys are comfortable speaking in English?*

MAN: Mm, nosotros no hablamos inglés.
TRANSLATION: *Mm, we don't speak English.*

MODERATOR: ¿Puro español?
TRANSLATION: *Only Spanish?*

MAN: Sí.
TRANSLATION: *Yes.*

WOMAN: Mm-hmm.

MAN: Sí.
TRANSLATION: *Yes.*

MODERATOR: Okay. A ver, ¿y hay obstículos que porque no pueden hablar inglés?
TRANSLATION: *Okay. Let's see, and are there [mispronunciation] because you can't speak English?*

WOMAN: Hay obstáculos.
TRANSLATION: *[Correcting moderator] Are there obstacles.*

MODERATOR: Oops, gracias.
TRANSLATION: *Oops, thanks.*

WOMAN: No.

MAN: Porque como cada quien sabe hacer su trabajo, no.
TRANSLATION: *Because everyone knows how to do their job, no.*

MAN: Claro, pues, siempre cuando . . . sería bueno. Estamos en un país de que es para que nosotros habláramos el inglés, entiende, porque aquí, gracias a Dios se acostumbra uno, pero es para que nosotros habláramos en inglés y tuviéramos más comunicación con ellos, me entienden, con los patrones. Claro, para mi, es algo importante, el inglés . . .
TRANSLATION: *Of course, well, always when . . . would be good. We're in a country that's for us to speak English, you know, because here, thank God you get accustomed, but it's for us to speak English and have more communication with them, you know, with the bosses. Of course, for me, it's something important, English . . .*

MODERATOR: Sí.
TRANSLATION: *Yeah.*

MAN: . . . pues, no sé, como que no entra o es que no ponemos . . .
TRANSLATION: *. . . so, I don't know, it just doesn't stick or it's that we don't put . . .*

WOMAN: No, a veces no hay como mucho tiempo también porque son diferentes horarios. Entonces, claro, a veces uno sale tan . . .
TRANSLATION: *No, sometimes there's not like a lot of time also because they are different schedules. So, of course, sometimes you leave so . . .*

[Interruption, one participant steps away]

WOMAN: . . . cansado de que lo que desea es descansar. Digamos como ayer, salimos a la, un ejemplo, salimos a las 8:00 p.m. y teníamos que entrar a las 3:30 a.m. Y había, que digamos, sacar el turno de 700 vacas en, para estar aquí en la reunión a las 10:45 a.m., ¿verdad? Entonces, sí . . .
TRANSLATION: *. . . tired that what you want is to rest. Let's say like yesterday, we left at, an example, we left at 8:00 p.m. and we have to go in at 3:30 a.m. And there was, let's say, taking the shift of 700 cows in, in order to be here at the meeting at 10:45 a.m., right? So, yeah . . .*

MODERATOR: Siempre entran a las 3:00 a.m. or nada más por la reunión tuvieron que entrar a las 3:00 a.m.?
TRANSLATION: *Do you always go in at 3:00 a.m. or just for the meeting you had to go in at 3:00 a.m.?*

WOMAN: No . . .

MAN: No, es que siempre da de acuerdo a los horarios porque se hacen tres turnos, me entiende, uno que entra, el que entra a las 3:00 a.m., 3:30 a.m., el otro entra a las 11:30 a.m., y comienza a las 11:30 a.m., y el otro entra a las 7:30 p.m. hasta las 4:00 a.m. Entonces . . .
TRANSLATION: *No, it always goes according to the schedules because there are three shifts, you know, one that comes in, the one that comes in at 3:00 a.m., 3:30 a.m., the other comes in at 11:30 a.m. and starts at 11:30 a.m., and the other comes in at 7:30 p.m. until 4:00 a.m. So . . .*

WOMAN: Ah. Okay. Ya entendí.
TRANSLATION: *Ah. Okay. Got it.*

MAN: . . . ¿me entiende? Y como nosotros los rotan, entonces a veces, tenemos el . . . tenemos que tratar de descansar y dejar de hacer unas cosas para descansar . . .
TRANSLATION: *. . . you know. And since we rotate them, so sometimes, we have the . . . we have to try to rest and not do some things in order to rest.*

WOMAN: Exacto.
TRANSLATION: *Exactly.*

MODERATOR: Ya entendí. Okay. ¿Y trabaja con alguien que no habla el mismo idioma que ustedes?
TRANSLATION: *Got it. Okay. And do you work with someone that doesn't speak the same language as you guys?*

MAN: Sí, a veces, pero ellos se acostumbran a uno, por lo menos, la americana, Jenny, ella, muchas veces tuve que trabajar con ella, pero ella habla palabritas así . . .
TRANSLATION: *Yes, sometimes, but they get used to you, a least, the American, Jenny, she, I've had to work with her many times, but she speaks some works like . . .*

MODERATOR: Okay.

MAN: . . . y a veces uno un poquito te entiende . . . porque yo no lo hablo, pero sí entiendo mucho. Yo sí entiendo . . .
TRANSLATION: *. . . and sometimes they understand you a little . . . because I don't speak it, but I understand a lot. I do understand . . .*

MODERATOR: ¿Hay alguien que tiene otro idioma fuera de inglés y español?
TRANSLATION: *Is there anyone who has another language other than English or Spanish?*

MAN: No.

WOMAN: No.

MODERATOR: ¿Nada más inglés y español? Okay.
TRANSLATION: *Just English and Spanish? Okay.*

[Interruption, participant who stepped away returns, then moderator speaks to lead researcher]

MODERATOR: ¿Dónde reciben más información sobre qué está sucediendo en la granja?
TRANSLATION: *Where do you receive more information about what's happening on the farm?*

MAN: Con el mánager . . .
TRANSLATION: *With the manager . . .*

MODERATOR: El mánager. Okay.
TRANSLATION: *The manager. Okay.*

WOMAN: Mm-hmm.

MODERATOR: ¿Cuándo tienen las reuniones, reunión?
TRANSLATION: *When do you have the meetings, meeting?*

MAN: No hay una fecha estipulada, pero de vez en cuando . . .
TRANSLATION: *There's isn't a stipulated date, but once in a while . . .*

MODERATOR: ¿Como una vez al mes?
TRANSLATION: *Like once a month?*

MAN: A veces una vez al mes, a veces a los dos meses, así.
TRANSLATION: *Sometimes once a month, sometimes every two months, like that.*

MODERATOR: ¿Dependiendo lo que está pasando?
TRANSLATION: *Depending on what's happening?*

MAN: Dependiendo de la necesidad que ellos tengan de hablar con los trabajadores, de comunicación con los trabajadores. Eso depende. Si todo está bien . . . pero, o sea, a veces tienen que hacerla, reuniones a veces al mes porque necesitan hablar algo. Ellos nos comunican de lo que está pasando, si todo está bien. Sí . . .
TRANSLATION: *Depending on the necessity, they have to speak with the workers, communication with the workers. That depends. If everything is fine . . . but, I mean, sometimes they have to do it, meetings sometimes every month because they need to talk about something. They communicate to us what's going on, if everything is okay. Yeah . . .*

MODERATOR: ¿Y todos los que trabajan aquí vienen a la reunión?
TRANSLATION: *And everyone that works here comes to the meeting?*

MAN: Todos.
TRANSLATION: *Everyone.*

WOMAN: Sí, o a veces tienen que informar algo de algún medicamento nuevo o cosas que hay que hacerles a las vacas, entonces también comunican.
TRANSLATION: *Yes, or sometimes they have to inform something about some new medicine or things that have to be done to the cows, so they also communicate.*

MODERATOR: Okay. ¿Y comunican nada más así o también hacen por text, email?
TRANSLATION: *Okay. And do they communicate just like that or do they also do by text, email?*

MAN: No. No, no, no.

WOMAN: No.

MAN: No, tienen una persona que viene a traducir.
TRANSLATION: *No, they have a person that comes to translate.*

MODERATOR: Okay. So les voy a hacer unas preguntas sobre su salud y la salud de las vacas, ¿okay? So primero, les voy a hacer preguntas sobre los antibióticos. Y los antibióticos son medicinas que usan los médicos para ayudar a una persona que tiene, persona o animal que tiene infección con bacteria para mejorarse. Los veterinarios usan estos medicinas también para ayudar los animales con infecciones para mejorarse. ¿Estás familiarizado con los antibióticos?
TRANSLATION: *Okay. So I'm going to ask you some questions about your health and the health of the cows, okay? So first, I'm going to ask you questions about antibiotics. And antibiotics are medicines that doctors use to help a person that has, person or animal that has an infection with bacteria to get better. Veterinarians use these medicines also to help animals with infections to get better. Are you familiar with antibiotics?*

MAN: Sí.
TRANSLATION: *Yes.*

MODERATOR: ¿Un poquito? Okay. ¿Y qué piensan sobre los antibióticos?
TRANSLATION: *A little? Okay. And what do you think about antibiotics?*

MAN: Pues, creo que es la manera más efectiva para combatir bacterias, infecciones, tanto en los seres humanos como en los animales.
TRANSLATION: *Well, I think that it's the most effective way to combat bacteria, infections, as much in humans as in animals.*

MODERATOR: Mm-hmm. Okay. ¿Y alguna vez un médico te ha explicado cómo tomarlos?
TRANSLATION: *Mm-hmm. Okay. And has a doctor ever explained to you how to take them?*

MAN: No. En esta granja, no. Yo he recibido información en otra granja que he trabajado, pero en esta granja, nunca ha venido alguien a explicarnos desde que yo he estado.
TRANSLATION: *No. On this farm, no. I have received information on another farm that I've worked on, but on this farm, no one has ever come to explain to us since I've been here.*

MODERATOR: Okay. ¿Y un doctor de humano, lo ha explicado cómo tomar antibióticos? ¿Sí?
TRANSLATION: *Okay. And a human doctor, have they explained how to take antibiotics? Yes?*

WOMAN: Sí, a mi, sí.
TRANSLATION: *Yes, to me, yes.*

MODERATOR: Okay. ¿Y qué tan fácil puedes obtener los antibióticos si piensas que los necesitas para usted?
TRANSLATION: *Okay. And how easily can you obtain antibiotics if you think you need them for yourself?*

MAN: No, para, solamente con una prescripción médica.
TRANSLATION: *No, for, just with a medical prescription.*

MODERATOR: ¿Y tienen acceso a doctores y antibióticos si se enferman?
TRANSLATION: *And do you have access to doctors and antibiotics if you get sick?*

MAN: Por lo menos yo, de acá, tengo mi doctor porque viajo hasta Rochester, Minnesota cuando necesito consulta médica. Ya fue tres horas de acá para ir donde mi médico, a una clínica comunitaria que hay en Rochester, Minnesota. Desde que estaba en Minnesota, pues, acostumbré a ir allí, y siempre voy a ir por cualquier cosa de mi y de ella.
TRANSLATION: *At least I, from here, I have my doctor because I travel to Rochester, Minnesota when I need a medical appointment. It was three hours from here to go to where my doctor is, to a community clinic that there is in Rochester, Minnesota. Since I was in Minnesota, well, I got used to going there, and I'm always going to go for anything regarding myself or her.*

MODERATOR: Okay. ¿Y dónde obtienen sus antibióticos cuando los necesita? ¿Minnesota?
TRANSLATION: *Okay. And where do you obtain your antibiotics when you need them? Minnesota?*

MAN: Bueno, si el doctor me los receta, yo decido en que farmacia los quiero comprar.
TRANSLATION: *Well, if a doctor prescribes them to me, I decide which pharmacy I want to buy them at.*

MODERATOR: Okay. ¿Y ustedes?
TRANSLATION: *Okay. And you guys?*

MAN: Como no me he sentido mal . . .
TRANSLATION: *Since I haven't felt sick . . .*

MODERATOR: ¿Nunca ha necesitado? Okay.
TRANSLATION: *You've never needed to? Okay.*

WOMAN: Yo tampoco.
TRANSLATION: *Me either.*

MODERATOR: Okay. ¿Alguna vez has tenido una infección y tomado antibióticos que no les hicieron sentir mejor?
TRANSLATION: *Okay. Have you ever had an infection and taken antibiotics that didn't make you feel better?*

MAN: No, o sea, yo solamente una vez cuando estaba en Minnesota que tenía un dolor de muela y por eso fue que me recetaron antibióticos para que se me rebajara la infección y después hacerme la extracción dental.
TRANSLATION: *No, I mean, I just once when I was in Minnesota that I had a toothache and because of that they prescribed me antibiotics so that the infection would go down and then to do the tooth extraction.*

MODERATOR: Yo también estoy tomando ahorita por mi diente. Me duele.
TRANSLATION: *I am also taking right now for my tooth. It hurts.*

MAN: Me recetaron muchísimo.
TRANSLATION: *They prescribed me a lot.*

WOMAN: ¿También fue el nervio?
TRANSLATION: *Was it also the nerve?*

MODERATOR: Sí, me quitaron el diente, so estoy tomando antibióticos ahorita también. So sé cómo se siente. Okay. Algunas veces los antibióticos paran de trabajar y no pueden matar o controlar la infección. Debido a esto, el antibiótico puede que ya no funcione para tratar la infección de una persona o un animal. Esto es el nombre resistencia de antibiótico. Otra manera para decir esto es la resistencia antibiótico. Ocurre cuando la bacteria puede resistir el efecto de la medicina y cuando, y continúa a crecer, insulo cuando se toma la medicina, incluso cuando se toma la medicina. La resistencia antibiótica puede afectar los humanos y los animales. ¿Está familiarizado con la resistencia antibiótica? ¿Han oído de eso?
TRANSLATION: *Yeah, they took out my tooth, so I'm taking antibiotics right now as well. So I know how you feel. Okay. Sometimes antibiotics stop working, and they can't kill or control the infection. Due to this, the antibiotic may not function anymore to treat the infection of a person or an animal. This is the name antibiotic resistance. Another way to say this is antibiotic resistance. It happens when the bacteria can resist the effect of the medicine and when, and continues to grow, [mispronunciation] when the medicine is taken, even when the medicine is taken. Antibiotic resistance can affect humans and animals. Are you familiar with antibiotic resistance? Have you heard of that?*

MAN: Sí, yo he oído.
TRANSLATION: *Yeah, I've heard.*

MODERATOR: ¿Sí? Okay. En tu vida contidiana . . .
TRANSLATION: *Yeah? Okay. In your [mispronunciation] life . . .*

[Correcting moderator]

MAN: Sí, cotidiana.

TRANSLATION: *Yeah, daily.*

WOMAN: Cotidiana.
TRANSLATION: *Daily.*

MAN: Cotidiana.
TRANSLATION: *Daily.*

MODERATOR: Cotidiana, gracias. ¿En tu vida cotidiana, alguna vez has escuchado o discutido antibióticos en la resistencia de antibióticas?
TRANSLATION: *Daily, thanks. In your daily life, have you ever heard of or discussed antibiotics in the antibiotic resistance?*

MAN: Yo no.
TRANSLATION: *I haven't.*

MODERATOR: ¿No? Okay. ¿Alguna vez tú o un ser querido ha tenido una infección con una bacteria resistencia a los antibióticos, resistente?
TRANSLATION: *No? Okay. Have you or a loved one ever had an infection with a bacterium resistance to antibiotics, resistant?*

MAN: No.

MODERATOR: ¿No? Okay. ¿Piensas que tu familia están en resgo . . .
TRANSLATION: *No? Okay. Do you think your family is at [mispronunciation] . . .*

[Correcting moderator]

MAN: Riesgo.
TRANSLATION: *Risk.*

MODERATOR: . . . riesgo, gracias, riesgo de contraer una infección resistente a antibióticos?
TRANSLATION: *. . . risk, thank you, risk of contracting an infection resistant to antibiotics?*

MAN: Pues, todos los seres humanos estamos propensos a cualquier tipo de infección, cualquier tipo de bacteria que se puede poner. Porque como las bacterias son microscópicas. Nadie las puede mirar. Nadie sabe en qué momento puede obtener una bacteria. En cualquier parte que vaya uno, tanta en un baño público, incluso en una tienda, en un supermercado, en cualquier parte, puede obtener una bacteria uno sin saberlo.
TRANSLATION: *Well, all human beings are susceptible to any type of infection, any time of bacteria that can be placed. Because like the bacteria are microscopic. No one can see them. No one knows at what moment you can get a bacterium. Anywhere you go, just as well in a public bathroom, even in a store, in a supermarket, anywhere, you can get a bacterium without knowing it.*

MODERATOR: Uh-huh, yeah. Okay. ¿Y cómo piensas que su lechera, lecherías, cómo estas son, a ver, la pregunta está . . . okay. ¿Ustedes piensan que la lechería aquí está afectados a los antibióticos que están, que tiene resistencia?
TRANSLATION: *Uh-huh, yeah. Okay. And how do you think that your dairy farm, dairy farms, how these are, let's see, the question is . . . okay. Do you think that the dairy farm here is affected to the antibiotics that are, that have resistance?*

MAN: Creo que no porque no hay mucha mastitis, no hay . . .
TRANSLATION: *I don't think so because there isn't much mastitis, there isn't . . .*

MODERATOR: ¿No han visto una infección en una vaca que no se ha ido con los antibióticos?
TRANSLATION: *You haven't seen an infection in a cow that hasn't gone away with antibiotics?*

MAN: No.

MODERATOR: ¿Siempre lo tratan bien?
TRANSLATION: *They always treat it well?*

MAN: Sí.
TRANSLATION: *Yes.*

WOMAN: Mm-hmm, mm-hmm.

MODERATOR: ¿Piensan que ellos tienen un papel en la resistencia antibiótica?
TRANSLATION: *Do you think they have a role in antibiotic resistance?*

MAN: ¿Quién es ellos?
TRANSLATION: *Who is they?*

MODERATOR: No entiendo la pregunta. Voy a preguntar.
TRANSLATION: *I don't understand the question. I'm going to ask.*

[Moderator speaks to lead researcher, clarifying with participants, skips question]

MODERATOR: Okay. ¿Listos?
TRANSLATION: *Okay. Ready?*

MAN: Sí.
TRANSLATION: *Yeah.*

MAN: Sí. Está bien.
TRANSLATION: *Yeah. Okay.*

WOMAN: Sí.
TRANSLATION: *Yeah.*

MODERATOR: ¿Qué son las reglas actuales en la granja sobre el uso de antibióticos?
TRANSLATION: *What are the current rules on the farm about the use of antibiotics?*

MAN: Pues, los antibióticos se usan solamente con, cuando el mánager dice, o sea, el mánager deja una lista que qué es lo que se va a aplicarle a cada vaca, si está una vaca con una enfermedad.
TRANSLATION: *Well, antibiotics are only used with, when the manager says so, I mean, the manager leaves a list that what is going to be administered to each cow, if a cow has an illness.*

MODERATOR: Okay. ¿Y cómo se entera los detalles sobre las reglas y si algo cambia?

TRANSLATION: *Okay. And how do you find out the details about the rules and if something changes?*

WOMAN: Ellos lo comunican, el mánager . . .
TRANSLATION: *They communicate it, the manager . . .*

MODERATOR: Okay. ¿Y quién en la granja tiene permiso para usar antibióticos en los animales?
TRANSLATION: *Okay. And who on the farm has permission to use antibiotics on the animals?*

MAN: Solo el mánager.
TRANSLATION: *Just the manager.*

MODERATOR: El mánager. Okay. ¿So ustedes nunca le dan los antibióticos?
TRANSLATION: *The manager. Okay. So you never give antibiotics?*

MAN: No.

MAN: No.

WOMAN: No.

MAN: Al menos que él ordene.
TRANSLATION: *Unless he orders it.*

MAN: Al menos que lo ordene.
TRANSLATION: *Unless he orders it.*

MAN: Si lo ordene, pero ya le dice a uno lo que va a hacer y, entiende, pero no podemos nosotros tomar esas medidas sin ordenes de él. Él es el que mira la vaca si lo necesita o no lo necesita. Pero nosotros no tenemos el permiso de . . .
TRANSLATION: *If he orders it, but he tells you what you're going to do and, you know, but we can't take those measures without orders from him. He is the one that looks at the cow to see if it needs it or no. But we don't have permission . . .*

WOMAN: Solo por . . .
TRANSLATION: *Just by . . .*

MODERATOR: Pero sí les, ¿mande?
TRANSLATION: *But you do, pardon?*

WOMAN: Solo por indicación médica.
TRANSLATION: *Just by medical direction.*

MODERATOR: Okay. Pero si tiene la indicación, ¿ustedes le pueden dar la medicina?
TRANSLATION: *Okay. But if you have the direction, you can give the medicine?*

MAN: Sí, eso cuando él ordene.
TRANSLATION: *Yes, that when he orders it.*

MODERATOR: Okay. So si él les dice, dale medicina . . .
TRANSLATION: *Okay. So if he tells you, give medicine . . .*

MAN: Sí.
TRANSLATION: *Yes.*

MODERATOR: ¿So sí están trabajando con los antibióticos?
TRANSLATION: *So you are working with the antibiotics?*

MAN: Pues, antibiótico . . .
TRANSLATION: *Well, antibiotic . . .*

MAN: Depende del tipo de antibiótico, me entiende, porque por lo menos hay un antibiótico que es para mastitis que es para cuando tiene infección en la ubre, por dentro, aquí ya casi no están usando eso. Antes se usaba bastante. Ahora, no, están prohibidos que es para, es como una que se llama espectramac(?), es para, que eso va por dentro de la vaca para combatir infecciones internas.
TRANSLATION: *It depends on the type of antibiotic, you know, because at least there is an antibiotic that is for mastitis that is for when it has an infection in the udder, inside, here now they're really not using that. Before it was used a lot. Now, no, they are prohibited that are for, it's like one that's called espectramac(?), it's for, that goes inside the cow to combat internal infections.*

MAN: Hay otra que se llama ToMORROW, otro que se llama pirsu(?), pero aquí no usan esa. En otra granja, yo he usado eso, pero en esta granja, veo que no usan eso.
TRANSLATION: *There's another one that's called ToMORROW, another that's called pirsu(?), but they don't use that here. On another farm, I have used that, but on this farm, I see that they don't use that.*

MAN: Antes usaba, pero ahora no. Ahora solo se trata diferente . . .
TRANSLATION: *They used it before, but now no. Now it's just treated different . . .*

MODERATOR: Okay. ¿Y porque no tienen mucho mastitis aquí o . . .
TRANSLATION: *Okay. And because you don't have much mastitis here or . . .*

MAN: Casi no hay mastitis. Son mastitis, lo que se hace es que ahora, desde que una vaca comienza con mastitis, se aparta y para el mejor tratamiento más continuo y . . . o sea, han visto que da más resultado, entonces.
TRANSLATION: *There really isn't mastitis. They are mastitis, what's done is that now, from the time a cow begins to have mastitis, it's separated and for the better, more continuous treatment and . . . I mean, they have seen that it has better results, so.*

MODERATOR: Okay. Pero entonces, ¿si una vaca tiene mastitis y necesita antibióticos, si el mánager les dice que le vaya y le de la medicina, ustedes sí la pueden dar?
TRANSLATION: *Okay. But then, if a cow has mastitis and needs antibiotics, if the manager tells you to go and give medicine, you can give it?*

MAN: Sí, allí, sí.
TRANSLATION: *Yes, there, yes.*

MAN: Sí.
TRANSLATION: *Yes.*

MAN: Que él ordene, si no . . .
TRANSLATION: *If he orders it, if not . . .*

MODERATOR: Okay. So entonces, sí les están dando ustedes, sí están trabajando con los antibióticos y dándolo.
TRANSLATION: *Okay. So then you are giving them, you are working with the antibiotics and giving it.*

MAN: Sí, depende, como le digo, porque hay un antibiótico, por lo menos aquí se usa antibiótico no son demasiados, cómo le dijera yo, muy perjudicante para la . . .
TRANSLATION: *Yeah, it depends, like I said, because there is an antibiotic, at least here they use antibiotics that aren't so much, how would I say it, very harmful for the . . .*

WOMAN: Agresivos, muy agresivos.
TRANSLATION: *Aggressive, very aggressive.*

MAN: . . . muy agresivos. Por lo menos, igual que uno, por lo menos, muchas veces uno, en nuestros países se usa una, cuando uno está con infección, toma una pastilla que se llama penicilina . . . es muy, demasiada. Y cambiaron a amoxicilina. Es más baja. Entonces lo mismo han hecho ahora con los animales. Han disminuido eso para no maltratar el animal.
TRANSLATION: *. . . very aggressive. At least, same as yourself, at least, many times you, in our countries they use a, when you have an infection, you take a pill that's called penicillin . . . it's very, too much. And they changed to amoxicillin. It's lower. So they've done the same now with the animals. They've decreased that in order to not abuse the animals.*

MODERATOR: Okay. Gracias. Okay. Ahora les voy a hacer preguntas sobre tu trabajo diario en la granja y la ropa que se lleva cuando está trabajando, que se pone cuando está trabajando. Okay. ¿Piensas que hay algunas cosas que puedes llevar al trabajo para protegerte?
TRANSLATION: *Okay. Thank you. Okay. Now I'm going to ask you questions about your daily work on the farm and the clothes that you wear when you're working, that you put on when you're working. Okay. Do you think that there are some things that you can wear to work to protect yourself?*

MAN: Sí.
TRANSLATION: *Yes.*

MODERATOR: ¿Sí? Okay. ¿Y me puede dar un ejemplo?
TRANSLATION: *Yeah? Okay. And can you give me an example?*

MAN: Para protegerse a uno, pues, por lo general, nosotros usamos ropa que nos protejan de que nos caiga mucha, ensuciarnos mucho. Aparte de eso, también mantiene los guantes que nos cambiamos para mantener la higiene, muchas cosas así.
TRANSLATION: *To protect yourself, well, in general, we use clothes that protect us from getting a lot of, getting really dirty. Apart from that, also keeping gloves that we change to maintain hygiene, many things like that.*

MODERATOR: Okay. ¿Botas especiales?
TRANSLATION: *Okay. Special boots?*

MAN: Sí. Sí, sí, sí. Botas especiales, calcetines especiales.
TRANSLATION: *Yes. Yes, yes, yes. Special boots, special socks.*

[Interruption, one participant leaves]

MODERATOR: Okay. ¿Cuáles son algunas cosas que haces para mantener la salud de ti y de las vacas?
TRANSLATION: *Okay. What are some of the things that you do to maintain your and the cow's health?*

MAN: Sobre todo, mantener limpio . . .
TRANSLATION: *Above all, keeping clean . . .*

WOMAN: Sobre todo, la . . . la higiene.
TRANSLATION: *Above all, the . . . hygiene.*

MAN: Sí, la estimulación de la vaca también. Hay que estimularla bien para que ya no se enferme. La buena estimulación y darle tiempo para que ya baje la leche . . . entiende, porque las vacas se enferman. Sí, igual que uno, uno, me entiende, tiene que . . .
TRANSLATION: *Yeah, the stimulation of the cows as well. You have to stimulate them well so that they don't get sick anymore. Good stimulation and giving them time so that their milk comes down . . . you know, because the cows get sick. Yeah, same as yourself, you, you know, have to . .*

WOMAN: Darle buen trato.
TRANSLATION: *Treating them well.*

MAN: Darle un buen trato . . .
TRANSLATION: *Treating them well . . .*

MODERATOR: ¿Separan las vacas cuando se enferman?
TRANSLATION: *Do you separate the cows when they get sick?*

MAN: Sí. Sí, aquí hay un establo aparte donde van, donde, que se llama el hospital donde mantienen las vacas que están enfermas. Casi mantienen vacas crónicas, crónicas, y solo las que están comenzando a enfermar, las apartan allí para, es de, allí es, cómo le dijera yo, ya se sabe cuando, en ese corral, allí en el establo, allí hay vacas que me dicen, tratarlas, necesitan más trato porque están enfermas. O sea, cuando entras a ese establo . . .
TRANSLATION: *Yes. Yes, here there is a stable set aside where they go, where, it's called the hospital where they keep the cows that are sick. They mostly keep chronic cows, chronic, and just the ones that are beginning to get sick, they separate them there to, it's, there is, how would I say it, it's already known when, in that corral, there in the stable, there are cows there that they tell me, treat them, they need more treatment because they are sick. I mean, when you enter that stable . . .*

MODERATOR: Y cuando están trabajando con las vacas que están enfermas, ¿se tienen que cambiar de ropa cuando van para atrás de las que están saludable o si tienen que lavar las manos o algo o no tienen que hacer . . .
TRANSLATION: *And when you are working with the cows that are sick, do you have to change clothes when you go back to the ones that are healthy or if you have to wash your hands or you don't have to . . .*

WOMAN: No, como usamos los guantes, entonces cambiamos guantes.
TRANSLATION: *No, since we use gloves, so we change gloves.*

MODERATOR: Okay. ¿Se cambian botas o hacen otra cosa?
TRANSLATION: *Okay. Do you change boots or do something else?*

WOMAN: No, solo nuevos guantes, nada más, porque es lo, uno más necesita las manos para . . . la vaca. Para no pasarle la infección a las que están buenas, se hace por eso.
TRANSLATION: *No, just new gloves, nothing else, because it's the, you need your hands most for . . . the cow. In order to not pass along the infection to the ones that are well, it's done for that reason.*

MODERATOR: ¿Cuáles son algunas cosas que estás obligada a llevar en la granja para proteger tu salud? Casi es la misma pregunta.
TRANSLATION: *What are some things that you are obligated to wear on the farm to protect your health? It's pretty much the same question.*

MAN: Guantes.
TRANSLATION: *Gloves.*

WOMAN: Guantes.
TRANSLATION: *Gloves.*

MODERATOR: Okay. ¿Tienes un uniforme?
TRANSLATION: *Okay. Do you have a uniform?*

MAN: No.

WOMAN: No.

MAN: Nuestra ropa, decir, la granja no nos proporciona ningún tipo de uniforme. Nosotros usamos, compramos nuestra propia ropa para protegernos.
TRANSLATION: *Our clothes, to say, the farm doesn't provide any type of uniform. We use, we buy our own clothes to protect ourselves.*

MODERATOR: Okay. So ahora les voy a hacer pregunta de los guantes. ¿So cuándo lleva guantes cuando estás en la granja?
TRANSLATION: *Okay. So now I'm going to ask you a question about the gloves. So when do you wear the gloves when you are on the farm?*

MAN: Cuando, para ordeñar las vacas, para limpiarles las ubres, las tetas de las vacas, para ir, siempre andamos guantes.
TRANSLATION: *When, to milk the cows, to clean the udders, the cows' teats, to go, we are always using gloves.*

MODERATOR: Siempre. Okay. ¿Y qué tipo de guantes se ponen?
TRANSLATION: *Always. Okay. And what type of gloves do you put on?*

MAN: De goma, de . . . ¿cómo le llaman?
TRANSLATION: *Gummy ones . . . how do you call them?*

WOMAN: De látex.
TRANSLATION: *Latex.*

MODERATOR: ¿Látex?
TRANSLATION: *Latex?*

WOMAN: Uh-huh.

MAN: Mm-hmm.

MODERATOR: ¿Y con qué, cuándo se las cambian, cuánto se ha ido?
TRANSLATION: *And how, when do you change them, how much has gone by?*

MAN: Cuando se me rompió un guante, me lo cambio inmediatamente.
TRANSLATION: *When a glove breaks, I change it immediately.*

MODERATOR: Okay. ¿Y estaba diciendo si trabajan con las vacas que están enfermas, se las cambian después, dijeron? Entendí o, si están con una que está enferma, después van a ir donde están saludables, ¿se las cambian?
TRANSLATION: *Okay. And you were saying, if you work with the cows that are sick, you change them after, you said? Did I understand or, if you are with one that is sick, then you go to where they are healthy, do you change them?*

MAN: Sí.
TRANSLATION: *Yes.*

WOMAN: Mm-hmm.

MODERATOR: Okay. ¿Algunas veces nada más se limpian los guantes o siempre se las cambian si están sucias?
TRANSLATION: *Okay. Do you sometimes just clean the gloves, or do you always change them if they are dirty?*

MAN: No, se cambian.
TRANSLATION: *No, they're changed.*

MODERATOR: Okay. ¿Se lavan las manos cuando están trabajando?
TRANSLATION: *Okay. Do you wash your hands when you're working?*

MAN: ¿Cómo así?
TRANSLATION: *What do you mean?*

MODERATOR: ¿Si se lavan las manos en el trabajo?
TRANSLATION: *If you wash your hands at work?*

MAN: No, pues, allí siempre tiene las manos sucias uno, por eso se usa guantes.
TRANSLATION: *No, well, there you always have dirty hands, that's why gloves are used.*

MODERATOR: Mm-hmm, ¿so no muy seguido?
TRANSLATION: *Mm-hmm, so not very often?*

WOMAN: No muy seguido.
TRANSLATION: *Not very often.*

MAN: No, porque . . .
TRANSLATION: *No, because . . .*

WOMAN: Siempre está protegido con los guantes. Ya retirar los guantes, sí se lavan . . .
TRANSLATION: *You're always protected with the gloves. Once you take off the gloves, you do wash . . .*

MODERATOR: ¿Cuando se quitan los guantes?
TRANSLATION: *When you take off the gloves?*

WOMAN: Exacto.
TRANSLATION: *Exactly.*

MODERATOR: ¿Y cuándo se van a quitar los guantes, ya cuando se van a casa o . . .
TRANSLATION: *And when are you going to take off the gloves, when you're going home or . . .*

MAN: No, si se le rompe un guante, pues, tiene que cambiárselo uno.
TRANSLATION: *No, if a glove breaks, well, you have to change it.*

WOMAN: Uh-huh.

MODERATOR: Pero dijo que se lava las manos después de quitarse los guantes, ¿cuándo es eso?
TRANSLATION: *But you said that you wash your hands after you take off the gloves, when is that?*

WOMAN: Digamos, en el momento que uno se quita los guantes, de hacer que se dañan o así, pues, uno se lava las manos para ponerse otros guantes.
TRANSLATION: *Let's say, the moment that you take off the gloves, maybe they are damaged or like that, well, you wash your hands in order to put on other gloves.*

MODERATOR: Oh. Okay. Ya entendí. ¿Cuáles son las veces más probables que se lave las manos?
TRANSLATION: *Oh. Okay. Got it. What are the most likely times that you wash your hands?*

MAN: Pues, cuando ya uno mira que las tiene sucias.
TRANSLATION: *Well, when you see that they are dirty.*

MODERATOR: ¿Y qué usa para lavarse las manos?
TRANSLATION: *And what do you use to wash your hands?*
+
WOMAN: Jabón en líquido.
TRANSLATION: *Liquid soap.*

MODERATOR: Okay. ¿Y agua?
TRANSLATION: *Okay. And water?*

MAN: Sí.
TRANSLATION: *Yes.*

MODERATOR: ¿Hay lugares accesibles fácilmente para lavarse las manos cuando se necesita?
TRANSLATION: *Are there easily accessible places to wash your hands when you need to?*

MAN: Sí.
TRANSLATION: *Yes.*

MODERATOR: ¿Sí?
TRANSLATION: *Yeah?*

WOMAN: Sí.
TRANSLATION: *Yes.*

MODERATOR: Okay. ¿El lavarse las manos algunas veces interferie con sus, con su capacidad para complitar tus deberes asignados? Perdón.
TRANSLATION: *Okay. Has washing your hands ever [mispronunciation] with your, with your ability to [mispronunciation] your assigned tasks? Sorry.*

WOMAN: No entendí.
TRANSLATION: *I didn't understand.*

MODERATOR: ¿Me la puede leer por favor la que está allí arriba?
TRANSLATION: *Can you read it for me, please, the one that's up top?*

WOMAN: ¿La primera . . .
TRANSLATION: *The first . . .*

MODERATOR: Sí, la primera, mm-hmm.
TRANSLATION: *Yes, the first one, mm-hmm.*

WOMAN: ¿El lavarse las manos alguna vez interfiere con su capacidad para completar sus deberes asignados? No.
TRANSLATION: *Has washing your hands ever interfered with your ability to complete your assigned tasks? No.*

MODERATOR: Gracias. Okay. ¿Tienes algunas reglas de, tiene unas reglas tu empleado sobre lavar, lavando las manos?
TRANSLATION: *Thank you. Okay. Do you have some rules, does your employee have some rules about washing your hands?*

MAN: Hmm-mm.

MODERATOR: ¿No? ¿Cómo te preparas para salir del trabajo para el día? Cuando se van de aquí, ¿se cambian la ropa . . .
TRANSLATION: *No? How do you prepare to leave work for the day? When you leave here, do you change clothes . . .*

MAN: Sí.
TRANSLATION: *Yes.*

MODERATOR: . . . o se quedan en lo que vinieron?
TRANSLATION: *. . . or do you stay in what you came in?*

WOMAN: No, se cambia.
TRANSLATION: *No, you change.*

[Interruption]

MODERATOR: Okay. ¿Dónde estamos? Okay. ¿Cómo se prepara para irse a casa?
TRANSLATION: *Okay. Where are we? Okay. How do you prepare to go home?*

WOMAN: Nos cambiamos la ropa.
TRANSLATION: *We change clothes.*

MODERATOR: ¿So todo la ropa, los zapatos, los jeans?
TRANSLATION: *So all clothes, shoes, jeans?*

MAN: Todo, todo. Todo.
TRANSLATION: *Everything, everything. Everything.*

WOMAN: Sí, todo. Uh-huh.
TRANSLATION: *Yes, everything. Uh-huh.*

MODERATOR: Okay. ¿Y se lavan las manos de nuevo?
TRANSLATION: *Okay. And do you wash your hands again?*

MAN: Sí . . .
TRANSLATION: *Yes . . .*

WOMAN: Sí.
TRANSLATION: *Yes.*

MAN: . . . nos lavamos las manos . . .
TRANSLATION: *. . . we wash our hands . . .*

MODERATOR: ¿Se tienen que bañar antes de irse a la casa?
TRANSLATION: *Do you have to shower before going home?*

MAN: No.

WOMAN: No.

MAN: No . . .

MODERATOR: Okay. Nada más se quitan, se cambian de ropa.
TRANSLATION: *Okay. You just take off, change clothes.*

MAN: Sí.
TRANSLATION: *Yeah.*

MODERATOR: Okay. ¿Y se llevan la ropa a la casa o la dejan aquí?
TRANSLATION: *Okay. Do you take the clothes home or leave them here?*

MAN: Nosotros llevamos la ropa para lavarla.
TRANSLATION: *We take the clothes to wash them.*

MODERATOR: Okay. ¿So la ropa sí se meta a su casa, la ropa sucia?
TRANSLATION: *Okay. So the clothes do go into your house, the dirty clothes?*

WOMAN: Mm-hmm, mm-hmm.

MODERATOR: Okay. ¿Y qué hace con ellos, con ropa sucia?
TRANSLATION: *Okay. And what do you do with them, with the dirty clothes?*

MAN: La lavamos.
TRANSLATION: *We wash them.*

WOMAN: Sí . . .
TRANSLATION: *Yeah . . .*

MODERATOR: Okay. ¿Lo lavan con toda su ropa de la casa?
TRANSLATION: *Okay. Do you wash it with your clothes from home?*

MAN: No, aparte, aparte.
TRANSLATION: *No, separate, separate.*

MAN: No, solamente la ropa que trabajamos en el rancho, la lavamos solamente la ropa en que trabajamos.
TRANSLATION: *No, just the clothes that we work in on the farm, we wash just the clothes we work in.*

MODERATOR: Okay. Y ya les pregunté esto, pero ¿trabajan alguna vez con los becerros o las vacas enfermas? ¿Trabajan con los becerros?
TRANSLATION: *Okay. And I already asked you this, but do you ever work with the calves or the sick cows? Do you work with the calves?*

WOMAN: Con los becerros y las vacas enfermas.
TRANSLATION: *With the calves and the sick cows.*

MAN: . . . o sea, la primera leche, calostro, segunda leche, nada más. Solamente le damos dos bebidas a los becerros pequeños, recién nacidos, nada más.
TRANSLATION: *. . . I mean, the first milk, colostrum, second milk, nothing else. We just give two drinks to the small calves, newborns, nothing else.*

MODERATOR: . . . ¿y también trabajan con las vacas enfermas?
TRANSLATION: *. . . and do you also work with the sick cows?*

MAN: Sí, sí.
TRANSLATION: *Yes, yes.*

WOMAN: Mm-hmm.

MODERATOR: Okay. ¿Llevan ropa especial o diferente cuando están trabajando con los becerros o las vacas enfermas?
TRANSLATION: *Okay. Do you wear special or different clothing when you are working with the calves or the sick cows?*

MAN: No.

MODERATOR: ¿No? Okay. ¿Qué tan efectivo piensan que llevando ropa, o, pues, no, no lo usan. ¿Cuándo trabajan con los becerros? Cuando les dan la leche, nada más, ¿verdad? . . .
TRANSLATION: *No? Okay. How effective do you think wearing clothing, or, well, no, you don't use it. When do you work with the calves? When you give them milk, just that, right?*

MAN: Sí.
TRANSLATION: *Yes.*

MAN: Sí.
TRANSLATION: *Yes.*

MAN: Sí.
TRANSLATION: *Yes.*

MODERATOR: Okay. ¿Y cuándo trabajan con las vacas enfermas?
TRANSLATION: *Okay. And when do you work with the sick cows?*

MAN: Pues, cuando, con las vacas enfermas, siempre trabajamos cuando los ordenan de que hay que tratarlas. En caso de las vacas, las que están recién paridas, siempre cuando tenemos que tratar . . . porque es una orden de que hay que darle fluid(?), la banda roja, todo eso ya es de nosotros. Ya sabemos cada quien de que tiene que hacerlo.
TRANSLATION: *Well, when, with the sick cows, we always work when they order that you need to treat them. In the case of the cows, the ones that have recently given birth, we always have to treat . . . because it's an order that you have to give fluid(?), the red band, all of that is on us. Everyone knows they have to do it.*

MODERATOR: Okay. ¿Sientes que corres riesgo en contratar una enfermedad de ellos?
TRANSLATION: *Okay. Do you feel that you run a risk of contracting an illness from them?*

MAN: No.

MODERATOR: ¿No?

MAN: No. Son más saludables, los animales son más saludables que uno.
TRANSLATION: *No. They're healthier, the animals are healthier than you.*

MODERATOR: ¿Y cómo te sientes sobre llevando cosas como guantes, overoles, o ropa de protección?
TRANSLATION: *And how do you feel about wearing things like gloves, overalls, or protective clothing?*

MAN: Pues, como nosotros nunca los hemos usado, pues, me da igual.
TRANSLATION: *Well, since we've never used them, well, it doesn't matter to me.*

MODERATOR: ¿Cómo se sienten de los guantes?
TRANSLATION: *How do you feel about the gloves?*

MAN: Así es costumbre.
TRANSLATION: *That's customary.*

MODERATOR: ¿Le gusta . . . los guantes o no les importa?

TRANSLATION: *Do you like . . . the gloves or it doesn't matter to you?*

MAN: Siempre se acostumbra . . .
TRANSLATION: *You always get used to it . . .*

MAN: Sí, acostumbra porque . . .
TRANSLATION: *Yeah, you get used to it because . . .*

WOMAN: Mm-hmm.

MAN: . . . hay que cubrirse las manos por las . . . de la vaca.
TRANSLATION: *. . . you have to cover your hands because of the . . . from the cow.*

MODERATOR: Okay. So les, ahora les voy a hacer preguntas más sobre las vidas de ustedes y las actividades en la granja. ¿Okay? Okay. ¿Dónde comes tus comidas cuando estás en el trabajo?
TRANSLATION: *Okay. So now I'm going to ask you questions more about your lives and the activities on the farm. Okay? Okay. Where do you eat your meals when you're at work?*

MAN: No comemos.
TRANSLATION: *We don't eat.*

MODERATOR: ¿No comen?
TRANSLATION: *You don't eat?*

MAN: Solo venimos listo.
TRANSLATION: *We just come ready.*

MODERATOR: Oh. ¿No agarran lonche, nada?
TRANSLATION: *Oh. You don't take lunch, nothing?*

MAN: No.

MODERATOR: ¿Nada más vienen con ya las panza llenitas?
TRANSLATION: *You just come with your bellies already full?*

MAN: Aquí no hay break . . .
TRANSLATION: *There is no break here . . .*

MAN: Aquí no hay break.
TRANSLATION: *There's no break here.*

MAN: . . . desde que empieza a trabajar hasta que termina. No hay break.
TRANSLATION: *. . . from when you start to work to when you finish. There isn't a break.*

MODERATOR: Okay. Si deciden que quieren comer algo, ¿hay un lugar que pueden . . .
TRANSLATION: *Okay. If you decide that you want to eat something, is there a place that you can . . .*

MAN: No.

MODERATOR: . . . o no los dejan?
TRANSLATION: *. . . or they don't let you?*

MAN: No, no es que no nos dejan, sino que ya venimos preparados para . . .
TRANSLATION: *No, it's not that they don't let us, rather that we already come prepared to . . .*

MODERATOR: Okay. ¿Cómo se encarga la leche cruda y productos de leche cruda en la granja?
TRANSLATION: *Okay. How do you handle the raw milk and raw milk products on the farm?*

MAN: ¿Cómo?
TRANSLATION: *What?*

MODERATOR: La leche cruda, ¿cómo la, qué hacen con ella, cómo se encargan?
TRANSLATION: *Raw milk, how, what do you do with it, how do you handle?*

MAN: No, la leche va al tanque, y ahorita está la sistema llevando la leche. No sé yo para qué compañía la lleva.
TRANSLATION: *No, the milk goes to the tank, and right now the system is bringing the milk. I don't know what company it brings it to.*

MODERATOR: ¿So ustedes nunca trabajan . . .
TRANSLATION: *So you never work . . .*

MAN: No.

MAN: No.

WOMAN: Hmm-mm.

MODERATOR: ¿Consumes o los . . . okay, ¿ustedes consumen la leche cruda aquí?
TRANSLATION: *Do you consume or the . . . okay, do you consume raw milk here?*

MAN: No, no.

MAN: No.

WOMAN: No.

MODERATOR: ¿So nadie aquí consume la leche cruda o productos de leche cruda?
TRANSLATION: *So no one here consume raw milk or raw milk products?*

MAN: No.

MODERATOR: ¿Nada?
TRANSLATION: *Nothing?*

MAN: No.

MODERATOR: Okay. ¿Hay reglas en la granja sobre consumir productos de la leche cruda?
TRANSLATION: *Okay. Are there rules on the farm about consuming raw milk products?*

MAN: No, pues, nunca nos han dicho nada, pero es que nadie acostumbra a consumir leche de las vacas.
TRANSLATION: *No, well, they've never told us anything, but no one really consumes milk from the cows.*

MODERATOR: ¿Piensas que hay riesgos en beber leche cruda?
TRANSLATION: *Do you think there are risks drinking raw milk?*

MAN: Claro que sí, porque es una leche que no se sabe cómo . . . le puede traer mucha bacteria de la vaca, entonces mejor no hacerlo. Cuando quiero leche, voy al supermercado y la compro.
TRANSLATION: *Of course, because it's a milk that it's unknown how . . . it can bring you a lot of bacteria from the cow, so better to not do it. When I want milk, I go to the supermarket, and I buy it.*

WOMAN: Ya está procesada, pasteurizada.
TRANSLATION: *It's already processed, pasteurized.*

MODERATOR: Sí, yeah. Okay. Se me hace que eso fue todo.
TRANSLATION: *Yes, yeah. Okay. I think that was everything.*

WOMAN: Ah, de rápido.
TRANSLATION: *Ah, quick.*

MODERATOR: Sí. Nada más le voy a preguntar a asegurar que ya es todo, pero se me hace que ya terminamos.
TRANSLATION: *Yeah. I'm just going to ask to make sure that that's everything, but I think that we're done.*

Focus Group 6, Farm 4
Recording in English

INTERVIEWER: . . . cover our bases. So like I said, my name is Nicole. This is Amanda and Paula. And just for the recordings, there's only two of you, so it shouldn't be that big of an issue, but one person talking at a time just makes it easier to transcribe. If you need to use the restroom or take a call, please, feel free at any time. And like I said, everything you say here is confidential. We won't share anything you say with managers, so we take privacy very seriously. So to get started, can each of you maybe just, I'd like just to learn a little bit more about your roles on the farm. So can you describe your role on the farm, how long you've been working here, and any other farming experience that you have?

RESPONDENT 1: Well, I'm like a calf feeder. And I've been working here for about two and a half years. And, I mean, that's about it.

INTERVIEWER: Okay. So what does calf feeding involve?

RESPONDENT 1: Basically, just coming in, feeding the calves their milk, mixing, washing bottles, watering, and giving grain to both the hutches and the group pens that need to be fed like starter or grower.

INTERVIEWER: Okay. Great, any previous farming experience?

RESPONDENT 1: I worked at a farm before here for like two months, but then I had to move. So it was not working out.

INTERVIEWER: Oh, okay. Sounds good.

RESPONDENT 2: I've been here for about 19 years. I can, my main job is primarily maintenance and field work. But I've been around long enough that I jump in, I can do, I know all the protocols for all positions on the dairy. I've even sat in the office a couple times. So it's previous farming experience, I was, I worked at a dairy before that. And actually before that, went to LTC, which is a college just south of here, and worked with this family as part of my internship. That's how I ended up back here.

INTERVIEWER: Okay. And what did, what were you studying at LTC?

RESPONDENT 2: Dairy herd management.

INTERVIEWER: Dairy herd management. Okay. Great. So now, I'd like to learn a little bit more specifically about this farm. Can you kind of walk me through what a typical shift at this farm is like?

RESPONDENT 2: Which position?

INTERVIEWER: If there is one. Both, so since there's just two of you, we can kind of go over.

RESPONDENT 1: Well, I come in roughly like around 11:15. And then I go to the milk room, count all the like calves that are on 3X, like fed three times a day, and count how many there are, if they're on high or low. The ones that are on high get the high feeding. So they're getting six pints each feeding, three times a day. And then low feeding, four pints, three times a day. Write them down, look at the sheet, kind of tells you how much milk, I mean, milk replacer and water is supposed to go

in the mixer and add all those numbers up, start filling up the mixer, get all the bottles out, then feed them.

And then, right, depending on what you do next, I normally start watering next, so they get their new, more water than they had right away. After that, I normally start washing bottles. And start taking off the nipples, then you kind of see which calves didn't drink right down, you know, how much they didn't drink, or, and, you know, dump it. Start rinsing, and we have a detergent that we use, then acid and bleach and then kind of repeat that cycle with the mixer so it gets, make sure it's all clean.

Then go and start graining hutches with the starter. And depending on if they're only fed milk once a day, then they get a half a pail of grain. And if they're on no milk, they get fed full. And move calves, if there are any moving, depending on the time of year. If they're not dry, then you normally leave them to, under the heater, you know, to make sure that, I don't think I'm missing anything.

INTERVIEWER: And what do you do if you have questions?

RESPONDENT 1: I normally go to either him or one of the other, I guess, managers, I guess I you could call her.

INTERVIEWER: Okay.

RESPONDENT 1: But, yeah, both kind of who I go to if I have questions or more of a . . .

INTERVIEWER: Do you have breaks, or work continuously?

RESPONDENT 1: Yeah, I mean, if I feel that I need a break and just like if I'm having a really bad headache or something, I can sit down and work.

INTERVIEWER: So it's kind of, but it's not scheduled. It's kind of when you need it?

RESPONDENT 1: Yeah.

INTERVIEWER: Okay.

RESPONDENT 2: And that particular shift is only like four or five hours long, so.

INTERVIEWER: Oh, okay.

RESPONDENT 1: Yeah, so it's not like it's a long, where it's eight hours. It's only a few. So it's not like I really need breaks.

INTERVIEWER: Okay.

RESPONDENT 2: Depending on the day, I'm here from anywhere from 4:00 in the morning at, I start anywhere from 4:00 in the morning until 7:30 in the morning. Some days I feed cows. Some days I'm in the tractor all day. Some days I'm fixing stuff all day because it's just been a bad day, and it really varies. I mean, you know, I might show up in the morning with a whole list of stuff to do and not get any of it done because I got pulled somewhere else.

INTERVIEWER: Oh, okay.

RESPONDENT 2: So, yeah, there's really not a set schedule that I have. And like, you know, I can take a break whenever I want to, but I don't go home until I'm done. So it might be 5:00 at night. It might be 10:00 at night. So I do, there are a lot of hours. I usually average about 75 a week.

INTERVIEWER: It sounds like maybe you're the one that people come to with questions, do you have someone you go to if you . . .

RESPONDENT 2: Some, yes. Yeah, for the most part, I know what's got to be done or who to go to get the right answer.

INTERVIEWER: Okay. Okay. So in, when, what kind of situations would you talk with your manager?

RESPONDENT 2: Expensive ones.

INTERVIEWER: Expensive ones. Okay.

RESPONDENT 2: If something is going to cost him a lot of money, I want to know how he wants it taken care of. But other than that, I mean, I pretty much have the rein to do what I feel. They've, in the 19 years I've been here, they've never questioned a decision I've made, so.

INTERVIEWER: How about you?

RESPONDENT 1: Kind of pretty much, you know, if I notice a calf isn't looking right, seem, you know, be off, doesn't seeming to be eating a lot like it should be, I kind of, you know, go and kind of let them know and ask if there's something I can do. Or, you know, if they want to take care of it. I mean, or if I notice something is wrong with something I'll, like the mixer, I don't know, mixer, for example, I guess, go and tell them like, oh, hey, having this issue with the mixer or kind of thing, four wheelers not acting right, or kind of thing.

INTERVIEWER: Okay, great. Can you describe any situations in which your manager would come talk to you?

RESPONDENT 1: More I guess, if, I guess if I'm doing something, wouldn't necessarily say correct. But, I mean, I guess if I'm kind of, they want something done a little bit differently than I've been doing it, like or maybe I'm not washing the bottles as correctly or something as I should be, then, you know, they'll be like, oh, hey, can you fix this and kind of make sure you're spending more time during this cycle, like on the bleach or whatever. Or if they want help with something that I can help them with kind of like, oh, can you have a few extra minutes to give me hand with this?

INTERVIEWER: Okay. How, what's kind of the frequency of when you interact with your, or how often you interact with your managers? Is it a kind of a daily basis, or?

RESPONDENT 1: I wouldn't say daily. But, I mean, I kind of, if I have a question about something, it's more of I'll go quick ask and give me an answer right away. But, I mean, I'm kind of at the point that, for the most part, I kind of have an idea of really what to do. So, I mean, that I don't often have to ask a bunch of questions about how something is done or . . .

INTERVIEWER: Sounds good. And how about you, are there situations that you have to . . .

RESPONDENT 2: I usually talk to them every morning around 7:30 or so for 15 or 20 minutes, just to, I mean, the only people that are above me are the two owners. So it, I mean, if I, just so that everybody is on the same page. Or if they know of something that is coming up that needs to be addressed, they'll let me know or the other way around. But other than that, if something comes up during the day, you've got a phone number, you call them or, whatever, text.

INTERVIEWER: Okay. And we touched a little bit on this, but how do breaks work on this farm? It sounds like it's kind of as needed.

RESPONDENT 1: Yeah.

RESPONDENT 2: Pretty much all the way through it's as needed in any position. The guys know what they have to do and when it's, you know, when they can get away for a few minutes, even in the parlor. There's specific groups that you really need two people in the parlor. And they know that they need to be there for that.

But other than that, like, no, here's, run 12-hour shifts, but they get an hour break in the middle. Whenever they want to do it in their shift, it's up to them. If it's four hours in or six hours in, that's totally at their discretion. But they do get that hour break. And everybody else is pretty much the same way. Whenever, you know, they know what their day is like, when they can, where they have to be, when they have to be, and they fit their breaks in wherever they see it appropriate.

INTERVIEWER: Okay. Where do you eat your meals while you're at work?

RESPONDENT 2: I usually don't sit down to eat. I usually eat in between jobs. I'm walking between wherever. But like anybody else uses this room.

INTERVIEWER: Okay. Yeah.

RESPONDENT 1: I'm normally am here late enough that I will try and eat beforehand. So, I mean, I'm not really, I don't really eat here unless I'm working a little bit longer than, but.

INTERVIEWER: Okay. Do people typically stay here for breaks, or will people, say it's an hour break . . .

RESPONDENT 2: Usually, they stay here because most people are traveling 10, 15 miles.

INTERVIEWER: Okay, yeah.

RESPONDENT 2: So most of them will stay here. They just bring stuff to eat with them. Every once in a while, somebody will run into like Subway in town or whatever, at the gas station and then bring it back here and eat it. But other than that, it's, they pretty much bring in what they need with them, stay here.

INTERVIEWER: Okay. So now we're going to kind of switch gears and ask, and talk a little bit more about like the clothes and gear that you wear while working. What rules does your farm have in terms of what you wear, if any?

RESPONDENT 2: There really aren't any other than, you know, milkers are required to wear plastic gloves. We do the same thing for calf feeding. But other than that, it's whatever you're comfortable in.

INTERVIEWER: Okay. And then, are there certain situations where you're expected to wear like coveralls, boots, shoe covers, gloves, oh, you said gloves, eye protection, face masks, filtered masks? Okay.

RESPONDENT 2: Not really. I mean, use common sense, I guess. If you're doing something where there’s a lot of stuff blowing around, and you need to wear eye protection, that’s not required, but . . .

INTERVIEWER: It would be a good idea.

RESPONDENT 2: It would be a good idea to wear. And for the most part, there's, other than myself, most of the employees aren't in situations like that unless it's like a really windy day outside or something.

INTERVIEWER: Okay.

RESPONDENT 2: But then again, I mean, it’s your own perspective, I guess.

INTERVIEWER: Is, are there, is there different gear that you use if you're working with calves or with, you said gloves . . .

RESPONDENT 1: Yeah.

INTERVIEWER: . . . or with sick animals?

RESPONDENT 2: Just make sure you change your gloves.

RESPONDENT 1: Yeah. Especially like with working like with sick and healthy calves. I mean, sick calves are always the last to be worried about because, you know, you don’t want to be working with them, and then you go and do something with a healthy calf, and you don't change out your gloves or kind of thing, it's . . .

RESPONDENT 1: So the one thing here, all of the sick cows that need to be treated with any antibiotics are separated from the main milking herd. So there’s just two people, primarily, that work with those animals. I mean, they do other vaccinations and stuff within the milking herd too, but the majority of their time is spent working with those animals during the day. And calves, we try to specify that, you know, if you have a sick animal, work with that one last so that you don't, you know, if you have a calf that's three hours old, you don't go and take whatever the one that's two weeks old has and give it to the one that's only three hours old.

INTERVIEWER: Okay. Yeah. And then you said something about switching gloves.

RESPONDENT 1: Yeah.

INTERVIEWER: Is that between each calf, or . . .

RESPONDENT 2: If you're working . . .

INTERVIEWER: If they’re sick?

RESPONDENT 2: If they’re sick.

RESPONDENT 1: Yeah.

INTERVIEWER: Okay. Got it. What are your hand washing practices at work?

RESPONDENT 2: Next to none.

INTERVIEWER: Are there times that you're more likely to wash your hands?

RESPONDENT 2: Well, yeah, if you're out working in like manure and stuff, then you try to.

RESPONDENT 1: Yeah.

INTERVIEWER: What is available to wash your hands with here?

RESPONDENT 2: There's several sinks around and hand soap.

INTERVIEWER: I see, okay. Let's see, how do you get ready to leave work for the day?

RESPONDENT 2: I just jump in my truck and go home.

RESPONDENT 1: I mean, I . . .

INTERVIEWER: So you leave with what you're wearing.

RESPONDENT 2: Come with what I wear, or come to work ready to work, and leave in the same clothes I came in, usually.

RESPONDENT 1: That's basically, I mean, I rinse off my boots, but if I, you know, have to run someone quick after, I might change. But, I mean, other than that I just, I'm five minutes away, so it's just . . .

INTERVIEWER: Is there laundry here that staff use, or does everyone pretty much come ready to work and . . .

RESPONDENT 2: No, there is, the milkers have, there's a washing machine here for the milkers to use. And the herds people use it too. But we don't have a drier here so they just hand their clothes up wherever. So and because there are so many people coming around, that sometimes stuff ends up missing. So that's, I just, it's easier for me to take it home. And it's . . .

INTERVIEWER: Don't have to worry about it.

RESPONDENT 1: Yeah.

RESPONDENT 2: You know, I go home and jump in the shower anyway, so.

INTERVIEWER: Okay. So you said you will hose off your boots, but do you leave boots here at all? Everything?

RESPONDENT 2: Yeah. We usually have, most people have boots that slip over their regular shoes so that your feet don't get wet.

INTERVIEWER: So now we're just asking about raw milk and a lot of the farms have said they don't usually deal with it. But how are raw milk and raw milk products handled on this farm?

RESPONDENT 2: As far as here, it just goes in the tank, and the milkman comes and gets it. There's no . . .

INTERVIEWER: Okay, so . . .

RESPONDENT 2: I mean, the only thing that, the only contact the milkers would have with it is if they are, well, in their prepping procedure, they have to strip, but that's why they wear gloves. And for strip, I don't know if you know what that means or not. They actually, when they're prepping the cow to milk, they strip the milk to make sure that it's normal. So they strip some milk out onto the floor to make sure it's normal and good to go into the tank.

And then there's a filter that they have to change because the regulations are the milk has to be filtered before it goes into the tank. So every three or four hours, they have to go into the milk room and change a filter. But other than that, it just goes into the tank, and the milkman comes and picks it up. We don't have any contact with it at all.

INTERVIEWER: So how would they, when they're stripping it, how would they know if it's not okay to go into the tank?

RESPONDENT 2: Different color or consistency.

INTERVIEWER: Okay. And are there any rules related to raw milk on the farm?

RESPONDENT 2: As far as?

INTERVIEWER: Use of it or . . .

RESPONDENT 2: We don't allow anybody to buy it. We don't allow anybody to drink it. I can't say that it doesn't happen. But, I mean, it's not like there's somebody here because we milk around the clock. There are, obviously, there are cameras and stuff around, but not everywhere.

INTERVIEWER: Okay. And then are you aware of any farm workers that have ever consumed raw milk or raw milk products?

RESPONDENT 2: I have, but not here.

INTERVIEWER: Not here, okay.

RESPONDENT 2: It used to be, growing up, that was, the milk came from the barn.

INTERVIEWER: Yep. And then so now, I'd like to ask some questions about antibiotics and just, I think you're probably aware that they're medicines, antibiotics are medicines that doctors use to help someone who has an infection that's caused by a bacteria, to get better. And as you're likely aware, veterinarians also use them for animals to help them. What is your personal experience with taking antibiotics?

RESPONDENT 2: Very little.

INTERVIEWER: Okay.

RESPONDENT 2: I’ve been to the doctor three times in the last nine years.

INTERVIEWER: Nice. I wish I were that healthy.

RESPONDENT 1: I don't really get sick often. So it’s more if I just, I don’t really go in that much for like sickness because I don't really get sick. So I don't really, I’ve really, I think I’ve taken maybe antibiotics maybe two times in my life. So I, yeah.

INTERVIEWER: You guys are exceptionally healthy.

RESPONDENT 1: Uh, we’re . . .

INTERVIEWER: In what situations are antibiotics helpful?

RESPONDENT 2: For us or for . . .

INTERVIEWER: Right now, for you. My next question will focus on animal usage.

RESPONDENT 2: I guess anytime you’re running, like you have some sort of infection.

RESPONDENT 1: Like an ear infection, or I don’t, I think you take antibiotics for strep throat.

RESPONDENT 2: Yes.

RESPONDENT 1: Okay, okay, yeah.

INTERVIEWER: And what situations are they not helpful?

RESPONDENT 2: For viruses.

INTERVIEWER: And then, so like I was mentioning, we’re focusing on antibiotic resistance, which is when antibiotics stop working and are no longer able to kill or control the growth of bacteria. And because of this, it doesn’t work to treat the infection. And so what are your thoughts on antibiotic resistance or kind of level of awareness or experiences related to it?

RESPONDENT 2: I think there’s enough change, just in antibiotics, you know, there’s always new ones coming out, probably for that reason, to try to, you know, head off the resistance. But they always come out with newer, better stuff, or usually anyway.

INTERVIEWER: It’s kind of, I, we’re just, it’s kind of a term that we’re trying to get a sense of what people’s awareness is. So is this like a word you’ve, or a phrase you’ve heard before, or is it, and something you're aware of like on the farm as an issue?

RESPONDENT 2: I’ve heard of it. But we’ve never had, I mean, the way the drugs are labeled, they’re labeled for, or antibiotics, they’re labeled for specific things. I mean, so you're not going to use, for instance, a mastitis treatment to treat something for pneumonia. So, I mean, it's, and, you know, there’s several different things to pick from. You just have, as far as antibiotics, you just have to look at the level of severity, and, you know, even the value of the animal.

INTERVIEWER: Have you or a loved one ever had an antibiotic-resistant infection? And what risks do you think antibiotic resistance poses to you and others in the community?

RESPONDENT 1: I mean, I guess, not being able to treat the, you know, like the infection or whatever that's going on. And, you know, you're either going to get more sicker, and other people are going to get sick too if you're not cautious. And, I mean, yeah.

RESPONDENT 2: Some if it has to do with, I think, how much it's used. I mean, if everybody, every person runs to the doctor every time they cough and get the same antibiotic, then sooner or later, it's going to catch up, but.

INTERVIEWER: What role, if any, do you think dairy farms play in antibiotic resistance?

RESPONDENT 2: As far as on the farm or in the general public?

INTERVIEWER: Both, I think.

RESPONDENT 2: On the farm, I think there's enough change. And as far as, like I mentioned before, new drugs, and the vet we work with is always on top of things as far as new stuff. So I would say, on the farm, it's probably pretty little.

INTERVIEWER: Do you have sense of it or?

RESPONDENT 1: No. I don't know.

INTERVIEWER: I see. Okay. And then what are the current rules on your farm about antibiotic use for animals?

RESPONDENT 2: Follow the label.

INTERVIEWER: Okay. Who on the farm is allowed to use antibiotics on animals?

RESPONDENT 2: The herdsmen. We have two herdsmen and myself. She does some, and my wife feeds calves. She does most of the treating of the calves. And the owners will sometimes. But usually, it just gets written down on a piece of paper that says, you need to do this. But in emergency cases, they will too.

INTERVIEWER: And what do you think about the antibiotic usage on this farm?

RESPONDENT 2: From an expense point of view, we try to keep it as a minimal, at a minimum. I mean, it's pointless to use it if it's not going to work. From a lot of perspectives, I mean, you're paying somebody to do it. You're paying for the drug. And if it's not going to do anything, I mean, it's a lot, I mean, it comes down to whether it's needed or not.

INTERVIEWER: All right. Well, that's pretty much all I have then. So but this was really helpful. So I appreciate you sitting down and talking with us. It helps us better understand how the inner workings of farms being not familiar ourselves. So thanks.

Focus Group 7, Farm 5
Recording in Spanish

MODERATOR: Okay. Bueno, primero, ya les había mencionado que vamos a grabar esto, que es confidencial. Hay evitar usar nombres de las personas si quieren hablar de alguien más o de ustedes. Y . . . que cada uno hable, una persona a la vez, y tratar de, si tienen los celulares, tratar de silenciar el celular. Pero si quieren tomar una llamada, pueden hacerlo libremente. O si quieren ir al baño, no hay problema, ¿okay?

Bueno, para empezar, lo primero que quería saber si me pueden contar un poco sobre ustedes, como su role en la granja, ¿qué es lo que hacen? Ya, así como, si no les molesta, vayamos como por cada uno de ustedes que me diga ¿cuál es el role que, el trabajo que hacen? ¿Hace cuánto tiempo llevan trabajando acá? Y si tienen alguna otra experiencia en otra granja, eso, básicamente. ¿Quiere comenzar?

TRANSLATION: *Okay. Well, first, I've already mentioned that we are going to record this, which is confidential. If you want to talk about yourselves or other people, we need to avoid using names of people. And . . . each person talks one at a time. And try to, if you have cellphones, try to silence your cellphone. But if you have to take a call, you can do that freely. Or if you want to go to the bathroom, that's not a problem. Okay?*

Okay. To start, the first thing I would like to know is if you can tell me a little bit about yourselves, like your role on the farm. What do you do? And then, if it's okay with you, we'll go one by one, and tell me, what is your role, what job do you do, how long have you been working here, and if you have any other farm experience, just that, basically. Would you like to start?

RESPONDENT 1: Empieza para allá.
TRANSLATION: *Start over there.*

RESPONDENT 2: ¿No, nombres?
TRANSLATION: *No names?*

MODERATOR: No, no. Solo que me cuentan que hacen.
TRANSLATION: *No, no. Just tell me what you do.*

RESPONDENT 3: Pues, yo, en particular, trabajamos seis días, descansamos dos. Y mi rutina diaria es traer las vacas de los corrales, limpiarles, ¿qué más? Prácticamente es lo que hago en todo, en mis ocho horas que trabajamos, entonces.
TRANSLATION: *Well, me particularly, we work six days, we're off for two. And my daily routine is to bring the cows from the pens, clean them, what else? That's pretty much what I do all, in the eight hours that we work, so.*

MODERATOR: Okay. Enfocar en los corrales entonces.
TRANSLATION: *Okay. You focus on the pens then.*

RESPONDENT 3: Sí.
TRANSLATION: *Yes.*

MODERATOR: Mm-hmm. Y ¿hace cuánto tiempo llevan trabajando?
TRANSLATION: *Mm-hmm. And how long have you worked here?*

RESPONDENT 3: Yo estoy ya ocho años.
TRANSLATION: *I've been here for eight years already.*

MODERATOR: Ocho años.
TRANSLATION: *Eight years.*

RESPONDENT 3: Ocho años.
TRANSLATION: *Eight years.*

MODERATOR: Y antes de eso, ¿trabajó en otra granja?
TRANSLATION: *And before this, did you work on another farm?*

RESPONDENT 3: No, no. Aquí empecé a trabajar en la granja.
TRANSLATION: *No, no. This is where I started working on a farm.*

MODERATOR: Okay. Sí, ¿usted?
TRANSLATION: *Okay. Yeah, you?*

RESPONDENT 2: Igual, seis días trabajo y dos días de descanso, ocho horas diarias. Y yo he trabajado en otros ranchos en este estado de Wisconsin.
TRANSLATION: *The same, I work six days, and I'm off for two days, eight hours a day. And I have worked on other farms in the state of Wisconsin.*

MODERATOR: Y ¿qué trabajo, específicamente, hace acá en la granja?
TRANSLATION: *And specifically, what job do you do here on the farm?*

RESPONDENT 2: No le entiendo.
TRANSLATION: *I don't understand.*

MODERATOR: Así, como, por ejemplo, alimentar a las vacas o limpiar o ordeñar, ese tipo.
TRANSLATION: *Like, for example, feed the cows or clean or milk, that kind of thing.*

RESPONDENT 2: Oh, sí, también ando afuera limpiando trayendo vacas . . .
TRANSLATION: *Oh, yeah, I'm also outside cleaning and bringing in cows . . .*

MODERATOR: Y ¿cuánto tiempo ya trabaja acá?
TRANSLATION: *And how long have you worked here?*

RESPONDENT 2: Más o menos como cuatro años.
TRANSLATION: *Four years, more or less.*

MODERATOR: Mm-hmm. ¿Usted?
TRANSLATION: *Mm-hmm. And you?*

RESPONDENT 1: Pues, yo trabajo cuatro días afuera, y trabajo dos ordeñando, mis dos otros días, ordeño.
TRANSLATION: *Well, I work outside for four days, and I milk for two days. On my two other days, I milk.*

MODERATOR: Cuando dice trabaja afuera, ¿es como en los corrales afuera?
TRANSLATION: *When you say you work outside, is that like in the pens outside?*

RESPONDENT 1: Sí, pues en los corrales . . . por las vacas. Porque hacemos trabajos diferentes. Trabajamos todos afuera, pero unos encarga de andar limpiándoles, andar limpiando corrales y

otros, como yo, nada más voy y ayuda a sacar el grupo, regreso. Les doy break acá adentro a los ordeñadores. Me encargo de poner a secar las toallas, echar a lavar las sucias, checar los tanques cuando se llenan, acomodarlos, es todo.

Y como cuando trabaja uno en el turno de la noche, pues, es diferente. Tengo que venir acá también los, las vacas que están pariendo. Pues, es diferente, como son tres turnos. Y vámonos cambiando cada mes, se van moviendo los turnos. O sea, no trabajamos los tres turnos fijos. Se va moviendo cada vez.
TRANSLATION: *Yeah, well, in the pens . . . with the cows. Because we do different jobs. We all work outside, but some people are in charge of cleaning, cleaning out pens, and others, like myself, I just help get the group out, and then I come back. I give breaks inside here to the milkers. I'm in charge of drying towels, washing dirty ones, checking the tanks when they're full, setting those up, that's all.*

And like when you work the night shift, well, that's different. I have to come over here as well for the cows that are giving birth. Well, it's different since there are three shifts. And every month they change the shifts, so we get moved around. I mean, we don't work a fixed shift. Each time, they move us.

MODERATOR: ¿Cuánto tiempo lleva trabajando acá?
TRANSLATION: *How long have you been working here?*

RESPONDENT 1: Llevo casi diez años. No más que me salí. Me salí como ocho meses, ya casi . . .
TRANSLATION: *Almost ten years. But I did leave. I left for like eight months, almost . . .*

MODERATOR: Y ¿tiene experiencia en otra granja también?
TRANSLATION: *And do you have experience on another farm as well?*

RESPONDENT 1: Pues, fueron como ocho meses, pero fueron una granja pequeña. Pasé como ocho meses allí. Pero ya casi llevo diez aquí.
TRANSLATION: *Well, it was like eight months, but it was a small farm. I was there for like eight months. But I've been here for almost ten years.*

MODERATOR: Mucho. Súper. Okay.
TRANSLATION: *That's a lot. Super. Okay.*

RESPONDENT 4: Pues . . . igual que ellos afuera. También a veces limpiamos o andamos. . . adentro checando los becerritos, pero hacemos lo mismo.
TRANSLATION: *Well . . . same as them outside. We also sometimes clean or we go . . . inside to check on the calves, but we do the same thing.*

MODERATOR: ¿Cuánto tiempo lleva trabajando aquí?
TRANSLATION: *How long have you been here?*

RESPONDENT 4: Siete años.
TRANSLATION: *Seven years.*

MODERATOR: ¿Y experiencia en otro lado, en otra granja?
TRANSLATION: *And have you had experience somewhere else, on another farm?*

RESPONDENT 4: No.
TRANSLATION: *No.*

MODERATOR: No. Siempre acá.
TRANSLATION: *No, only here.*

RESPONDENT 4: Siempre aquí.
TRANSLATION: *Always here.*

RESPONDENT 5: Yo soy el encargado aquí de la gente, de mánager. Yo me encargo de poner arena en los corrales cuando, se pone dos días a la semana, los martes y viernes. Y los jueves, secamos vacas. Les hago las pezuñas, y él me ayuda, y él también ayuda hacerlas.
TRANSLATION: *I'm in the manager in charge of the people here. I'm in charge of putting sand in the pens two days a week, Tuesdays and Fridays. And on Thursdays, we dry cows. I trim their hooves, and he helps me, and he also helps me trim.*

RESPONDENT 2: Se me pasó eso.
TRANSLATION: *Oh, I forgot to mention that.*

RESPONDENT 5: Eso hacemos los jueves. Entrando empezamos y acabamos a las 11:00, 12:00, más o menos. Pues, más que todo, yo me encargo adentro también de hacer los horarios, los turnos de si alguien pide día, cubrirlo, todo eso. Y también llevarle arena, ponerles arena acá a los demás corrales. Porque acá hay otros que trabajan en la mañana, y para ponerles arena también, los traigo. Y lo qué se necesita más que todo. Y cuando hace falta algo, ya, también llamarle al patrón para que lo ordene y todo eso. Y ya tengo 20 años aquí.
TRANSLATION: *We do that on Thursdays. We start when we get to work, and we finish by 11:00, 12:00, more or less. Well, I'm mostly in charge of making the schedules, the shifts if someone asks for a day off, covering them, all that. And I also bring in sand. I bring in sand over here to the rest of the pens because there are others who work in the morning, and I bring them sand as well. And, really, whatever needs to be done. When something is running low, then I call the boss so that he can order it and everything. I've been here for 20 years already.*

MODERATOR: Y ¿tiene experiencia en otras granjas?
TRANSLATION: *And do you have any experience on other farms?*

RESPONDENT 5: Estuve trabajando en una chiquita unos días. Un tiempo ayudando a un señor que necesitaba ayuda. Pero no más como dos meses iba en las tardes, tres horas no más. Pero es todo. Casi todo el tiempo he trabajado aquí.
TRANSLATION: *I was working on a small farm a few days. Just for a short time, I was helping a guy that needed help. But I only went for like two months in the afternoons, no more than three hours. But that's it. I've worked here almost the entire time.*

MODERATOR: Sí, 20 años . . . ¿y usted?
TRANSLATION: *Yeah, 20 years . . . and you?*

RESPONDENT 6: Yo también me dedico a lo mismo. Somos corraleros todos. Limpiamos los corrales, pushamos las vacas . . . y todo eso, y pues los becerros. Casi estamos haciendo lo mismo.
TRANSLATION: *I also do the same thing. We all work in the pens. We clean the pens, we push the cows . . . and all that, and the calves too. We all pretty much do the same thing.*

MODERATOR: Y ¿también los tres, los van turnándose?
TRANSLATION: *And also the three, your shifts change too?*

RESPONDENT 6: Sí, cambiamos diferente. O sea, maneja un día sí, un día no, nada más limpiando, y . . . a limpiar . . .
TRANSLATION: *Yes, we change to different ones. I mean, one day, yes, one day, no, just cleaning, and . . . to clean . . .*

MODERATOR: Y ¿cuánto tiempo lleva acá?
TRANSLATION: *And how long have you been here?*

RESPONDENT 6: Diez años.
TRANSLATION: *Ten years.*

MODERATOR: Diez años. Y ¿tiene experiencia en otros lados?
TRANSLATION: *Ten years. And do you have experience other places?*

RESPONDENT 6: Pues, trabajé en un rancho de caballos. Un rancho de caballos, pero a veces ayudo por ratos, pero ya tengo mayor tiempo aquí trabajando.
TRANSLATION: *Well, I worked on a horse ranch. A horse ranch, but sometimes I help for a while. But the majority of the time I've been working here.*

MODERATOR: ¿Usted?
TRANSLATION: *And you?*

RESPONDENT 7: Yo también hago lo mismo de traer las vacas. Y a veces, cuando, por ejemplo, cuando trabajo con los demás, como a veces vienen diferentes personas a ayudarnos entonces yo, ellos limpian o si yo limpio los corrales. Y a la otra persona se encarga de dar break y todo eso. Es casi lo mismo lavar donde toman agua las vacas. Y más que nada, checar los becerritos y también las vacas cuando están pariendo. Y trabajé dos años y medio en una granja pequeña también, como de 500 vacas. De allí, trabajé dos años en un rancho también de caballos. Igual, limpiando, dándolos de comer y todo. Aquí, llevo seis años.
TRANSLATION: *I also do the same, bring the cows. And sometimes when, for example, when I work with the others, like sometimes different people come to help us, so I, they clean, or I'll clean the pens. And the other person is in charging of giving breaks and all that. It's the same, wash where the cows drink water. And more than anything, checking on the calves and the cows when they are giving birth. And I work for two and a half years on a small farm too, like with 500 cows. From there, I worked for ten years on a horse ranch as well. Same thing, cleaning, feeding, all that. I've been here for six years.*

MODERATOR: Okay. Súper. Y si me pueden contar un poquito como cual sería la rutina diaria. Si usted, cuando llegan, como la rutina durante todo el día, lo que hacen.
TRANSLATION: *Okay. Super. And if you could tell me a little bit about your daily routine. If you, when you get here, like what is the daily routine throughout the day, what do you do?*

MAN: ¿Quién se anima?
TRANSLATION: *Who wants to answer?*

MODERATOR: ¿Quién se anima? Cualquiera.
TRANSLATION: *Who wants to go for it? Whoever.*

MAN: Pues, llegamos y a la hora de empezar vamos y lavamos las tomaderas donde toman agua. Y Pues ya esperamos a que salga el otro turno para empezar nosotros a sacar las vacas. Platicamos también.
TRANSLATION: *Well, we get here, and when we start, we go and we wash the tanks that the cows drink from. And, well, then we wait for the other shift to leave so we can get the cows out. We chitchat too.*

MAN: . . .

MAN: Pero pues, eso es lo que, como nos toca a veces. Como dice él, nos toca un día . . . otro día . . . a veces tenemos que ir allá al . . . arriba ayudarles a pasar el primer grupo como son las más nuevecitas que no quieren entrar. Pero esas casi son las rutinas de diaria.
TRANSLATION: *But, well, that is what, what we do sometimes. Like he said, sometimes it's one day . . . the other day it's . . . sometimes we have to go over to . . . up there to help them get the first group through since they're the new, and they don't want to go in. But those are like the daily routines.*

MODERATOR: ¿No les toca ordeñar?
TRANSLATION: *You guys don't milk?*

MAN: Cuando damos break, no más. Cuando le damos 10 minutos, bueno, ya son 15, ¿13? ¿15?
TRANSLATION: *Only when we're giving someone a break. When we give them 10 minutes, well, it's 15 now. 13? 15?*

MAN: Trece minutos.
TRANSLATION: *Thirteen.*

MAN: Yeah.

MAN: Sí, solamente cuando les damos break.
TRANSLATION: *Yeah, just when we give them a break.*

MAN: Para que vayan a comer algo, los que están adentro porque son, como quien dice ocho horas sin parar. Entonces tienen que ir al baño o algo. Ya si en seguida alguien quiere ir al baño, lo cubren también o algo, especialmente las mujeres. Pero regularmente, son tres turnos. Y cuando empiezan es un poco diferente en cada uno de lo que se hace también.

Por ejemplo, ahorita el turno de la tarde llega y lava tomaderas y tiene que poner medicina a las patas de las vacas, pasar los grupos. Eso es lo que hacen. Y en la mañana, no. No más es las tomaderas y limpia todos los pasillos de los corrales de los grupos. Y en la noche, también lavan las tomaderas y limpia otros pasillos de otros grupos.

Y son los que se dedican, más que todo, acá venir en la noche, el vaquero que trae, él que limpia a chequear a los becerros. Están chequeando toda la noche hasta las seis de la mañana, ya que llegan los demás. Es, en cada turno, es un poco diferente lo que hacen afuera. Sí, pero todos puchan las vacas, pues. Las arriman(?) para la parlor.
TRANSLATION: *So that the ones who are on the inside can go get something to eat because they are like eight hours without stopping. So they have to go to the bathroom or something. Then if someone has to go to the bathroom, then someone covers them, especially the women. But normally, there are three shifts. And when they start, each one is a little bit different also.*

For example, right now, the afternoon shift comes and washes the water tanks and has to put medicine on the cow's hooves as the groups pass through. That's what they do. And in the morning,

they don't. It's just the water tanks and cleaning the walkways of the pens of the different groups. And at night, they also wash the water tanks and clean the walkways for other groups.

And they are the ones that, more than anything, dedicate their time at night, the herdsman(?) that, the one that cleans, checks the calves. He checks on them all night until 6:00 in the morning until the rest come. It's, each shift is a little bit different in what they do outside. Yeah, but everyone pushes the cows. They get them together to go to the parlor.

MODERATOR: ¿Acá todos tienen más o menos la misma rutina entonces?
TRANSLATION: *So you guys have the same routine here more or less?*

[Simultaneous discussion]

MAN: No, porque en la mañana . . .
TRANSLATION: *No because in the morning . . .*

MAN: Afuera es diferente.
TRANSLATION: *Outside, it's different.*

MAN: . . . en la mañana que estamos . . .
TRANSLATION: *. . . in the morning we are . . .*

MAN: Ordeñando es otro . . .
TRANSLATION: *Milking is another . . .*

MAN: Como ya en la mañana, estamos un mes. Para el otro mes que viene, vamos a andar en la tarde. Entonces todos van cambiando. Pero es casi la misma rutina que hacen en la mañana, en la tarde, y en la noche. Pero hay diferentes cosas que se hacen. Sí.
TRANSLATION: *Like how we'll work in the morning for a month. Next month, we'll be working in the afternoon. So we all change. But it's basically the same routine for the morning, afternoon, and at night. But there are some different things that are done. Yeah.*

MODERATOR: ¿Alguien me quiere comentar más de su rutina?
TRANSLATION: *Does anyone want to say anything more about the routine?*

MAN: De la ordeña, llegue uno a ponchar y faltan diez minutos de la entrada de ordeñar. Hay que ponchar, se necesita alistarse, preparar las máquinas, descolgarlas, y esperar que terminen . . . todo listo ya, ya empiezan.
TRANSLATION: *For milking, I punch in and I have ten minutes from getting here to milking. You have to punch in. You have to get ready, prepare the machines, unhook them, and wait . . . everything is ready, then you start.*

MAN: . . . checar los tanques también que estén abiertos porque luego se quedan cerrados. Tengo que chequear todo esto, pues.
TRANSLATION: *. . . check that the tanks are open too because then they stay closed. I have to check all that.*

MAN: Todo listo, ya empezamos a trabajar, a ordeñar. Cuatro personas adentro ordeñando. Uno testeando, uno limpiando, otro pegando, y otro echando dip.
TRANSLATION: *When everything is ready, then we start milking. There are four people inside milking, one testing, one cleaning, one hooking up, and another applying dip.*

MAN: Es que casi toda la mañana nos dedicamos a trabajar afuera. Ahorita los únicos que ordeñan más, son ustedes, ¿no?
TRANSLATION: *Almost the whole morning we are working outside. Right now, the only ones who are milking more are you guys, right?*

MAN: Nosotros dos. Somos los únicos que ordeñamos, creo, dos días ¿no? . . . nosotros somos los dos que, los demás son puros que trabajan afuera. Nada más traen las vacas.
TRANSLATION: *Us two. We are the only ones milking, I think, two days, right . . . we are the ones that, the rest just work outside. They just bring the cows in.*

MAN: Seis días.
TRANSLATION: *Six days.*

MAN: Si encuentran vacas que vienen enfermas o a veces, se les pone un bote para que no se ordeñe para el tanque. Ya estés, allí atrás el que estaba persona,(?) lo aparta uno, y ya lo traen . . . hospital, ya que se encargan de darle medicina o hacerles lo que tienen que hacer.
TRANSLATION: *If you find cows that look sick, sometimes they put on a bucket so that the milk doesn't go into the tank. And then there's a person behind that separates them and brings them to . . . hospital, and there they're in charge of giving them medicine or doing whatever they have to do.*

MAN: Eso es lo que hacemos todos los días.
TRANSLATION: *That's what we do every day.*

MODERATOR: Y por ejemplo, ¿qué hacen si tienen alguna duda durante el día si, a donde, a quién le preguntan?
TRANSLATION: *And, for example, what do you do if you have questions during the day if, where do you, who do you ask?*

MAN: A él.
TRANSLATION: *Him.*

MAN: A él, o al patrón.
TRANSLATION: *Him or the boss.*

MAN: O al patrón.
TRANSLATION: *Or the boss.*

MAN: Como más en la noche. Como en la noche, pues, andamos solos, si se descompone una maquina o está fallando una maquina o no jala bien algo, no sirve, entonces le hablan el patrón.
TRANSLATION: *More at night. Like at night, well, we're alone. If a machine breaks down or fails or isn't working right or something, then I call the boss.*

MODERATOR: Y entonces, ¿cuándo hablarían como con su administrador con el mánager? ¿Hablan siempre con usted, o solamente cuando ya está muy, como muy complicado? ¿O es como una comunicación frecuente así?
TRANSLATION: *So when would you call, talk with your administrator or your manager? Do they always talk to you, or just when it's really, like really complicated? Or is it like pretty frequent communication?*

MAN: Pues, a veces tratamos de arreglar las cosas nosotros. Si no podemos, le hablamos a él y ya les hablen al patrón que está más cerca y ya está bien, o al hijo del patrón que está allí al lado. Pero

la mayoría de las veces, hablamos a él.
TRANSLATION: *Well, sometimes we try to fix things ourselves. If we can't, we call him, and him, and then they call the boss that is closest, or the owner's son, who is over there. But most of the time, we call him.*

MODERATOR: Perfecto. Y, por ejemplo, ¿en qué situaciones o el mánager, usted en ese caso, tendría, viene hablar con ellos en el trabajo?
TRANSLATION: *Perfect. And, for example, in what situations would the manager, you in this case, have to come and talk with them on the job?*

MAN: Cuando se va a hacer un cambio que se necesita o cuando haya algún problema, tratar de solucionarlo . . . entre uno para no ir con el patrón. Y ya si no se soluciona, entonces hacemos una junta los juntamos a nuestros problemas y vamos con el patrón. Pero casi siempre cuando, pues, se va hacer algún cambio aquí en el turno.
TRANSLATION: *When there needs to be a change or when there is a problem, to try to solve it . . . amongst ourselves so we don't have to go to the boss. And then if it can't be fixed, then we have a meeting, we talk about our problems and we go to the boss. But almost always when, well, when there is going to be a change during the shift.*

MODERATOR: Cuando cambian de turno, ¿allí como que se reúne?
TRANSLATION: *When they change shift, so how would you meet?*

MAN: Es cuando si voy a hacer algún cambio, pues, les digo a los vaqueros y a los del turno adentro que, por ejemplo, ayer que no se lavó, les dije a ellos para que se arrimaran las vaca y siguieran ordeñando, que no lavaran porque andábamos un poco atrasados para . . . pero casi siempre cuando los del turno . . . cambio o algo . . .
TRANSLATION: *It's when I'm going to make a change, well, I tell the herdsmen and those on the shift inside that, for example, yesterday when we didn't wash, I told them to get the cows together and keep milking and not to wash because we were running a little bit behind to . . . but almost always when the people on the shift . . . change something . . .*

MODERATOR: Y ¿cómo funciona los descansos acá en . . . tienen algunos descansos así cada cierto tiempo, o cuando pueden, o son fijo?
TRANSLATION: *And how do the breaks work here . . . do you have breaks like at a certain time, or when can you, or are they at a fixed time?*

MAN: Son . . .
TRANSLATION: *They are.*

MAN: Son fijos.
TRANSLATION: *They're fixed.*

MAN: Son fijos.
TRANSLATION: *They're fixed.*

MAN: Se mueven. Pero son seis días de trabajo si descansan dos.
TRANSLATION: *They move. But it's six days of work and two days off.*

MAN: Por ejemplo . . .
TRANSLATION: *For example . . .*

MAN: Entramos seis días. Estamos descansando dos. Y al siguiente descanso, se mueve uno para atrás, un día. Por ejemplo, si esta descansa lunes y martes, para el otro martes y miércoles, y de allí, miércoles y jueves porque son seis días, y la semana trae siete. Entonces se va recorriendo un día. Pero son seis días. Ya si alguien quiere un día extra de descanso, me dicen y tengo junta para cubrir, para trabajar cuando alguien quiere entonces . . .
TRANSLATION: *We're here for six days. We are off for two days. And for the next off days, it moves one day backwards. For example, if you're off on Monday and Tuesday, then the next week is Tuesday and Wednesday, and then Wednesday and Thursday, because it's six days, and the week has seven days. So it keeps going back by a day. But it's six days. And if someone wants an extra day off, they tell me, and I have a meeting to find coverage for work when someone wants of, so . . .*

MAN: No más los que si no tienen fijos, son los cubridores. Los que están de cubridores, ellos sí no saben qué día.
TRANSLATION: *But the ones who don't have a fixed schedule are the fill-ins. The fill-ins don't know what days.*

MAN: Los cubridores, sí, cualquier hora.
TRANSLATION: *The fill-ins, yeah, whatever time.*

MODERATOR: Pero, y durante el día, así como, ¿sí tiene un break para almorzar o cosas así?
TRANSLATION: *But, and during the day, like do you have a break for lunch or things like that?*

MAN: No más los 13 minutos que le dan.
TRANSLATION: *Just the 13 minutes they give you.*

MODERATOR: Y ¿eso es fijo . . . o se lo van tomando dependiendo como va el trabajo?
TRANSLATION: *And that's fixed . . . or do you take it depending on how work is going?*

MAN: Los de adentro saben cuando les va a tocar, pegar, es, allí es cuando les toca su break.
TRANSLATION: *The ones inside know when it's going to be their turn, it's, in there, it's when their break is.*

MODERATOR: Y en ese break de, ¿ese es para lunch, no?
TRANSLATION: *And that break, is it for lunch?*

MAN: Sí.
TRANSLATION: *Yeah.*

MODERATOR: ¿Vienen a comer acá, o dónde comen?
TRANSLATION: *Do you come eat here, or where do you eat?*

MAN: Allá tienen . . .
TRANSLATION: *They have a . . .*

MAN: Allí está la cocina.
TRANSLATION: *The kitchen is over there.*

MODERATOR: Ah, tienen una cocina. Okay. Y por ejemplo ¿si tienen descanso para ir al baño, o cada uno puede, sabe que puede ir cuando quiera o tiene que preguntar?
TRANSLATION: *Oh, you have a kitchen. Okay. And for example, do you have a break to go to the*

bathroom, or everyone can just, everyone knows that they can go when they want, or do they have to ask?

MAN: Le preguntan al que está dando los breaks al vaquero que, si le da chanza de cubrirle, pues, un ratito para que vaya al baño.
TRANSLATION: *They ask the person who is giving breaks, the herdsman, if they can cover them for a little bit so that they can go to the bathroom.*

MODERATOR: Ah, ya, se va preguntando allí a la persona. Okay. Y por ejemplo, ah bueno, y esos descansos se quedan acá en la granja, entonces no salen para comer afuera, bueno 13 minutos.
TRANSLATION: *Oh, okay, you go ask the person there. Okay. And for example, okay, and you guys stay here on the farm for those breaks, so you don't go out to eat, well, 13 minutes.*

MAN: No pero, después de que acaban de, se acaban . . . y a veces, se acaban una hora antes, pues, ya salen a break, a tomarse un break.
TRANSLATION: *No, but after we're done, after . . . and sometimes, you get done an hour early, well, then you go for a break, to take a break.*

MAN: Dependiendo del tiempo en que terminamos cada turno porque a veces, por cualquier cosa, se retraso uno en el turno porque a veces se cae una vaca porque está enferma o ya no se levanta del corral, entonces uno pierde esos 10 o 15 minutos que tarda en pararla . . .
TRANSLATION: *It depends on the time that each shift gets done because sometimes, for whatever reason, someone gets behind on the shift because sometimes a cow falls down because it's sick and won't get up out of the pen. So you lose those 10 or 15 minutes that it takes to get her up.*

MAN: Quince minutos, y para recuperarlo no está fácil.
TRANSLATION: *Fifteen minutes, and then to get those back is not easy.*

MAN: . . . entonces diario, no tenemos una hora fija que acabar. Entonces depende a qué horas terminemos al último, pues también podemos comer porque, pues, sí, hay veces que hay tiempo, hay veces que no. Pero por lo regular es diferente diaria entonces.
TRANSLATION: *. . . so daily, we don't have a fixed end time. So it depends on the time that we finish the last one, well, then we can eat because, well, sometimes there is time and sometimes there's not. But regularly, it's different daily, so . . .*

MAN: Pues, sí, la pregunta es que, si agarramos media hora, a veces la agarramos, a veces, no porque está muy difícil a . . .
TRANSLATION: *Well, if the question is if we get half an hour, sometimes we do and sometimes we don't because it's really difficult to . . .*

MODERATOR: ¿Pero siempre se quedan en la granja, por ejemplo a comer a algún otro lado o a la casa?
TRANSLATION: *But do you always stay on the farm, for example, to eat somewhere else or at home?*

MAN: No, quedamos cerca a la cocina.
TRANSLATION: *We stay close to the kitchen.*

MAN: Andamos al 100% todo el día.
TRANSLATION: *We're at 100% all day.*

MODERATOR: Ahora vamos a repasar como otras preguntas sobre, qué tipo de ropa ocupan y todas esas cosas para trabajar. Lo primero es que ¿qué reglas tiene la granja en términos de vestimenta, qué ropa tienen que usar para el trabajo?
TRANSLATION: *Now we're going to go over some other questions about the type of clothing you use and all those things to do your job. The first is, what rules does the farm have in terms of what you wear? What clothing do you have to wear to do your job?*

MAN: Pues la regla, reglas de seguridad aquí sí tenemos que usar como adentro que trabajo uno ordeñando, de usar los lentes y pues, hay mandiles, hay mandas para cubrirse entonces. Prácticamente son las reglas más, usar los lentes para los dos. Porque usa uno líquido entonces puede . . . ya la ropa sí una usa ropa uno la que uno quiera.
TRANSLATION: *Well, the rule, the safety rules here are that we do have to use, inside if you're milking, you have to use the safety glasses, and, well, there are aprons, there are aprons to cover yourself. Those are practically the rules, use the safety glasses for both because you use a liquid so you could . . . but the clothing, yeah, you wear whatever clothing you want.*

MAN: Cómodo.
TRANSLATION: *Comfortable.*

MAN: La que uno se sienta más cómodo. Entonces con la ropa no hay . . .
TRANSLATION: *What you feel most comfortable in. So with the clothes there isn't . . .*

MAN: La bota es así con casquillo, botas que tengan casquillo porque . . .
TRANSLATION: *Boots that have steel toes, steel-toed boots because . . .*

MAN: Sí, pues, puede pisar una vaca o con una máquina entonces sí, es más preferible usar una bota con un casquillo.
TRANSLATION: *Yeah, well, because a cow could step on you or with a machine so, yeah, it's preferable to use steel-toed boots.*

MODERATOR: O sea, como de las botas serían como los más obligatorio, ¿no?
TRANSLATION: *So like the boots would be the most mandatory thing, right?*

MAN: Botas y lentes.
TRANSLATION: *Boots and safety glasses.*

MAN: Lentes.
TRANSLATION: *Safety glasses.*

MAN: Botas y lentes y los guantes también.
TRANSLATION: *Boots and safety glasses and the gloves too.*

MAN: Sí, guantes también.
TRANSLATION: *Yeah, gloves too.*

MODERATOR: ¿Esos para la ordeña?
TRANSLATION: *Those are for milking?*

MAN: Sí.
TRANSLATION: *Yes.*

MAN: También . . .
TRANSLATION: *Also . . .*

MODERATOR: Y si no, cualquier, la ropa cómoda, ¿no?
TRANSLATION: *And if not, whatever, comfortable clothing, right?*

MAN: Sí.
TRANSLATION: *Yeah.*

MAN: Antes sí, tenían de este ¿no? Antes, sí, tenían uniformes, hace tiempo.
TRANSLATION: *Before you guys had these, right? Before, you had uniforms, a while ago.*

MAN: Antes sí.
TRANSLATION: *Before, yes.*

MODERATOR: Existe alguna situación donde tengan que usar, por ejemplo, cubre zapatos, o bueno, la protección para los ojos me dijeron para solamente la ordeña, o máscaras faciales, ¿ese tipo de cosa no?
TRANSLATION: *Is there any situation that exists where you have to use, for example, shoe covers, or I mean, you told me eye protection just for milking, or face masks, that kind of thing?*

MAN: Allí cuando uno, cuando ponen la medicina para las patas posiblemente como es polvo y . . . máscaras para que . . . porque como se pueda y . . . la nariz y hay máscaras. Pero para las botas, todas usan botas regulares y van para adentro y las lavan se lavan, y ya van para afuera regresan las lavan, pero botas de plástico es todo.
TRANSLATION: *When you, when you put on the hoof medicine, it's possible, since it's dust and . . . masks to . . . because you can . . . your nose and there are masks. But for the boots, we all use regular boots and we go inside, and we wash them, they're washed, and when they go back out, they wash them, but just the plastic books, that's all.*

MODERATOR: Y por ejemplo, cuando están trabajando con los . . . enfermo, ¿alguien si trabajan con algún animal (?) enfermo?
TRANSLATION: *And, for example, when you are working with sick . . . does someone work with any sick animals?*

MAN: No . . .
TRANSLATION: *No . . .*

MAN: Casi nosotros, no.
TRANSLATION: *We don't really, no.*

MAN: Los, ellos, aquí no más si la miren en el corral una vaca enferma, la apartan y la traen aquí al hospital. Y entonces el señor, ellos dos encargan. Los de este lado y otro. Son los que se encargan de ponerle calcio o lo que necesite. Pero con los enfermos, no. Lo único que hacen en la noche es darle de comer a los becerros aquí ellos también, cuando trabajan de noche que nacen, es lo único. Pero lo de más enfermo, no.
TRANSLATION: *They, them, here they just, if they see a sick cow in a pen, they separate it, and they take it to the hospital. And then the man, those two guys are in charge. The guys over there and one other guy. They're the ones that are in charge of applying the calcium or whatever they need. But with the sick ones, no, the only thing is the night shift feeds the calves here when they work at night and they're born, but the rest of the sick ones, no.*

MODERATOR: Okay. ¿Ninguna de ustedes ha tenido la experiencia manejando con animales enfermo?
TRANSLATION: *Okay. None of you have had the experience dealing with sick animals?*

MAN: No.
TRANSLATION: *No.*

MAN: Yo, sí, he puesto . . . también, pero ellos son los que se encargan.
TRANSLATION: *I have given . . . also, but they are the ones who are in charge.*

MAN: A veces cuando vamos andamos de . . . de vez en cuando.
TRANSLATION: *Sometimes when we're . . . once in a while.*

MODERATOR: Claro. Bueno, y ¿qué piensa sobre las reglas de vestimenta que tienen que usar, por ejemplo, cuando ordeña qué les parece?
TRANSLATION: *Of course. Okay. And what do you think about the rules about the clothing that you have to use, for example, when you're milking, what do you think?*

MAN: Pues, son, parece bien porque, pues, te cae, si te cae en el ojo el . . . perjudica de su uno(?).
TRANSLATION: *Well, they are, they seem okay because, well, if it gets, if it gets in your eye . . . you could get hurt.*

MAN: Más que todo seguridad es lo mismo. Porque a veces hay alguna, hace alguna basura o algo entonces cubre algo . . .
TRANSLATION: *More than anything, safety is the same because sometimes there's some kind of junk or something so it covers something . . .*

MAN: O hay unas vacas que se van moviendo y . . .
TRANSLATION: *Or there are some cows that are moving and . . .*

MAN: O una que patean a veces y . . . toda la cara y ya te protejan los lentes.
TRANSLATION: *Or one that kicks sometimes and . . . in the face and the safety glasses protect you.*

MODERATOR: Sí, pero no es como que sea molesto o incómodo para ustedes?
TRANSLATION: *Yeah, but it's not like bothersome or uncomfortable for you?*

MAN: No.
TRANSLATION: *No.*

MAN: Se acostumbra.
TRANSLATION: *You get used to it.*

MODERATOR: Se acostumbran, o sea, ¿los guantes y esas cosas?
TRANSLATION: *You get used to is, you mean, the gloves and those things?*

MAN: Sí.
TRANSLATION: *Yeah.*

MAN: Sí, ya nos acostumbraba desde mucho tiempo. Y al primero, sí, como que no los lentes, pero ya nos acostumbramos.

TRANSLATION: *Yeah, we got used to it a long time ago. And at first, yeah, like you didn't want to wear the safety glasses, but we're used to it now.*

MODERATOR: ¿Y hay ocasiones, por ejemplo, las que es difícil usar ese equipo de protección? . . . bueno, no los quieren usar.
TRANSLATION: *And are there occasions, for example, that it's difficult to use the safety gear . . . or, don't want to use it?*

MAN: Hay unos que no los quieren usar. Hay unos que . . .
TRANSLATION: *There are some people that don't want to use it. There are some that . . .*

MAN: . . . calor.
TRANSLATION: *. . . heat.*

MAN: Sí el calor y anda uno sudando. Y ya no alcanzo ni mirar bien.
TRANSLATION: *Yeah, the heat, and you're sweating, and you can't even see well.*

MAN: En este tipo de trabajo, así como dice el mánager, no es que quiera uno, sino que como dices, por bien para uno porque pues si se lastima uno, hay tener cuidado . . . en esos trabajos. Hay que, como dicen unos que . . .
TRANSLATION: *In this type of job, like the manager said, it's not about wanting to do it, it's like you said, for your own good because, well, if you get hurt, you have to be careful . . . in these jobs. You have to, like some say . . .*

MAN: Es una regla más que todo.
TRANSLATION: *It's a rule more than anything else.*

MODERATOR: Okay.

MAN: No, pero sí sirve comoquiera traer los lentes. A mi una vez andaba poniendo liquido de salida, y me metí el vaso para echarle líquido a la vaca y me lo pateo, me lo aventó así y me cubrieron los lentes. Si no hubiera trajeron . . . los ojos. No más, que, sí hay veces que también uno, ahí está uno de, que no los quiere usar y eso, pero . . . si te protejan . . .
TRANSLATION: *No, but either way, it does help to have the safety glasses on. One time I was putting some liquid on on my way out, and I had the glass to apply the liquid and the cow kicked me and it flew out like this, and my safety glasses covered me. If I hadn't had them . . . my eyes. It's just that, there are times when you just don't want to use them and all that, but . . . they do protect you.*

MODERATOR: ¿Y cuáles son las prácticas de lavado de manos acá cuando tienen que ir a lavarse las manos?
TRANSLATION: *And what are the handwashing practices here, when do you have to go wash your hands?*

MAN: Pues todo el tiempo. Como cuando se mete uno por, pues, igual, como cuando va uno a comerse algo o que va comer para adentro para la cocina, allí se lava uno los manos entonces, pero no hay . . .
TRANSLATION: *Well, all the time. Like when you want to go, well, like when you want to go eat something or you're going to go inside the kitchen to eat. You wash your hands in there, but there isn't . . .*

MODERATOR: Y pero en el trabajo ¿tienen algún lugar para lavarse las manos, qué tipo de jabón ocupan y eso?
TRANSLATION: *But at work do you have a place to wash your hands, what type of soap do you use, those kinds of things?*

[Group responds, Yes, simultaneously]

MODERATOR: Y ¿Tienen como jabón especial o cómo?
TRANSLATION: *And do you have like a special soap or what?*

MAN: Pues jabón especial, especial, no. O sea, casi siempre usamos con el que usamos para lavar. Es el mismo. Pero, pues, es, no pasa nada. Pues, es jabón. Pero todo el tiempo pues hay en el baño, hay en un sink que está allí cortito, la cocina, para hacer eso. Así es que cada vez que van a comer o algo se las lavan. O a veces que hacen el cambio también porque los de adentro están rotando cada hora en cada grupo, se están moviendo de posición también. Entonces hay unos que se quitan los guantes y cambiárselos también, todo eso.
TRANSLATION: *Well, like a special, special soap, no. I mean, we almost always use the one we use to wash. It's the same. But, well, it's, nothing bad happens. It's just soap. But there's always soap in the bathroom, there's soap at the sink that is right there, the kitchen, to be able to wash. So every time you're going to go eat or something you wash them. Or sometimes, when the rotate, because inside, they're rotating every hour in each group. They are switching positions. So there are some people that take their gloves off and change them too, all of that.*

MODERATOR: Y en la ordeña ¿hay un momento donde digas es más frecuente la lava de mano . . .
TRANSLATION: *In during milking, is there a moment where you would say you wash your hands more frequently?*

MAN: No, cuando cambian los filtros . . .
TRANSLATION: *No, when you change the filters . . .*

MAN: Usamos muchos los guantes.
TRANSLATION: *We use the gloves a lot.*

[Simultaneous discussion]

MAN: Usan todo, en la ordeña, usan todo el tiempo los guantes, entonces.
TRANSLATION: *They use, during milking, they use gloves all the time.*

MODERATOR: Okay.

MAN: Se cambia uno, y se pone guantes nuevos, se tire los sucios y agarre guantes nuevos todo el tiempo . . . igual cuando cambian los filtros uno, se pone guantes nuevos para no, para que no toque la mano al filtro. Ya ponemos guantes nuevos.
TRANSLATION: *You change, and you put new gloves on. You throw out the dirty ones and grab new ones all the time . . . also when you change out the filters, you put new gloves on, so you don't, so your hand doesn't touch the filter. We put new gloves on.*

MODERATOR: O sea, ¿no hay como alguna instrucción fija de, cuándo tienen que lavarse, cómo tiene que lavarse las manos y cómo que no hay una regla?
TRANSLATION: *I mean, so there's not like a strict instruction on when you have to wash, how you have to wash your hands?*

MAN: Pues, no porque, pues, todos saben que se les tienen que lavar. Especialmente cuando van a ir a comer algo se tienen que lavar las manos porque si las traen sucios, pues, bueno, hay unos que no se las lavan . . .
TRANSLATION: *Well, no, because, well, we all know that we have to wash. Especially if you're going to go eat you have to wash your hands because they're dirty, well, I mean, there are some that don't wash . . .*

[Simultaneous Discussion]

MAN: Cuando va uno al baño.
TRANSLATION: *When you go to the bathroom.*

MAN: Es normal, tiene que lavarse las manos.
TRANSLATION: *It's normal, you have to wash your hands.*

MAN: Sí, ya saben que hay bacterias . . .
TRANSLATION: *Yeah, we know that there's bacteria . . .*

MAN: . . . de líquido, de arena, de cualquier cosa. Siempre vienen sucio de las manos, se tiene que lavar.
TRANSLATION: *. . . from liquid, from sand, from whatever. Your hands are always dirty, you have to wash them.*

MODERATOR: Okay. Y ¿cómo se preparan para salir del trabajo en el día? Por ejemplo, ¿dónde se preparan para salir? ¿Tiene algún lugar?
TRANSLATION: *Okay. And how do you prepare to leave for work for the day? For example, where do you prepare to go home, is there a place?*

MAN: Tenemos un vestidor.
TRANSLATION: *We have a changing room.*

MODERATOR: ¿Un vestidor?
TRANSLATION: *A changing room?*

MAN: Sí.
TRANSLATION: *Yeah.*

MAN: Venimos con ropa de la casa y aquí tenemos nuestra ropa para el trabajo, botas, ropas, lo que usemos.
TRANSLATION: *We come with clothes from home, and we have our clothes here for work, boots, clothing, what we use.*

MAN: Tiene sus, cada quien sus lockers.
TRANSLATION: *You have your, everyone has their locker.*

MODERATOR: O sea, ¿que cuando llegan, llegan con su ropa, y acá, se ponen la ropa del trabajo?
TRANSLATION: *I mean, so when you come, you come with your clothes. And when you get here, you put your work clothes on?*

MAN: Mm-hmm. Sí.
TRANSLATION: *Mm-hmm. Yeah.*

MODERATOR: Okay. Y . . .
TRANSLATION: *Okay. And . . .*

MAN: Todas la dejan allí y la lavan juntos en la lavadora. Y alguien la recoge y al otro día, limpia.
TRANSLATION: *Everyone leaves them there, and they get washed together in the washing machine. And someone picks them up the next day, clean.*

MODERATOR: Entonces cuando van a salir de vuelta . . . claro, se cambian . . .
Okay. Bueno, ahora vamos a empezar preguntas sobre la leche cruda, que se, como se maneja la leche cruda acá en la granja. Por ejemplo, y si se consume algo, producto crudo acá en la granja, qué hacen con la leche cuando está cruda y todo eso.
TRANSLATION: *So when you come back again . . . of course, you change . . . Okay. Well, now we're going to begin some questions about raw milk, how it's handled here on the farm. For example, and if you consume it, any kind of raw milk product here on the farm, what you do with the raw milk here and all of that?*

MAN: La leche todo el tiempo está cruda aquí. De aquí se la llevan a una quesería. Pero, pues, yo, hay algunos que vienen por leche para hacer queso. Pero quede cruda la leche todo el tiempo. Y no más que ya cuando sale del tanque, ya va helada porque pasa por el congelador, y helada allí.
TRANSLATION: *The milk is raw all the time here. From here, they take it to a cheese factory. But, well, I, some people do come for the milk to make cheese. But the milk is raw all the time. And when it leaves the tank, it's already chilled because it goes through the cooler, and it chills there.*

MAN: Ya filtrada.
TRANSLATION: *And it's been filtered.*

MAN: Filtrada y toda limpia. Sí, pero no veo nadie que toma leche. Yo sí, tomo a veces tomo leche.
TRANSLATION: *Filtered and all clean. Yeah, but I don't see anybody drink the milk. I do drink the milk sometimes.*

[Simultaneous Discussion]

MAN: Eres el único.
TRANSLATION: *You're the only one.*

MAN: Yo nunca lo he probado.
TRANSLATION: *I've never tried it.*

MODERATOR: ¿Pero leche de acá?
TRANSLATION: *The milk from here?*

MAN: Yo sí. Sí. A veces allí del chiche, lo ordeño yo a veces. Sí, pero . . .
TRANSLATION: *I have, yeah. Sometimes from the teat, I milk it sometimes. Yeah, but . . .*

MODERATOR: ¿Y ustedes?
TRANSLATION: *And you guys?*

[Simultaneous Discussion]

MAN: No.
TRANSLATION: *No.*

MAN: No, yo nunca he probado.
TRANSLATION: *No, I've never tried it.*

MAN: Les pega . . .
TRANSLATION: *You get . . .*

MAN: El olor está mal de la leche.
TRANSLATION: *The smell of the milk is bad.*

MODERATOR: ¿Sí?
TRANSLATION: *Oh, yeah?*

MAN: Es muy fuerte.
TRANSLATION: *It's very strong.*

MODERATOR: ¿Es muy fuerte?
TRANSLATION: *It's strong?*

MAN: Sí.
TRANSLATION: *Yes.*

MODERATOR: Y en las otras granjas donde han trabajado ¿no saben, tampoco lo han probado?
TRANSLATION: *And on the other farms where you've worked, do you know, have your tried it there?*

MAN: Yo de la otra granja, sí. Sí, llevaba de la otra. Porque pues allí en aquella leche la leche es más natural porque allí no las inyectan ni nada. Era, como eran poquitas vacas.
TRANSLATION: *From the other farm, yes. I did take some from the other farm. Because, well, that milk is more natural because there they don't inject them or anything. It was, since it was just a few cows.*

MAN: Aquí tampoco.
TRANSLATION: *Here either.*

MAN: Antes no . . . antes sí.
TRANSLATION: *Before . . . before, we did.*

MAN: Ya tenemos dos años que no se inyectan.
TRANSLATION: *It's been two years now that they don't get injected.*

MAN: No, si, verdad que sí. No, pero del otro rancho, yo si llevaba de allí.
TRANSLATION: *Yeah, that's true. No, but the other farm, I did take milk from there.*

MAN: ¿Qué pasó?
TRANSLATION: *What happened?*

MAN: Por eso, allí es más natural.
TRANSLATION: *Well, there it was more natural.*

MODERATOR: Pero tienen alguna como . . .
TRANSLATION: *But they have like a . . .*

MAN: O sea, no digo que está mala, pero, pues, o sea, no de este.
TRANSLATION: *I mean, I'm not saying it's bad or anything, but well, I mean, not from this . . .*

MODERATOR: Y ¿tienen alguna regla acá sobre la leche cruda que como se debería de manejar?
TRANSLATION: *And do you have any rules here about the raw milk and how it should be handled?*

MAN: No porque de las pompas pues, va al tanque.
TRANSLATION: *No because from the pumps, it goes to the tank.*

MODERATOR: Ah, claro, directo al tanque.
TRANSLATION: *Oh, of course, directly to the tank.*

MAN: Entonces de ese tanque, ya pasa por la pipa y pasa por el cooler y se va al tanque que se las llevan, es todo, pero.
TRANSLATION: *So from that tank, it goes through the pipe and it goes through the cooler and it goes to the tank that they haul away, that's it.*

MODERATOR: O sea ninguno tiene contacto en el fondo con la leche.
TRANSLATION: *So nobody has contact with the milk in the background?*

MAN: No.

MAN: No. No más cuando cambian los tanques. Pero no exactamente con la leche, sino con los tubos . . . todo eso.
TRANSLATION: *No. Just when they switch out the tanks. But not exactly with the milk, but with the tubes . . . all that.*

MAN: Porque va directo todo desde la, donde están ordeñando la vaca. Entonces todo va directo. No hay un . . .
TRANSLATION: *Because it goes directly from the, where they're milking the cow. So it all goes directly, there isn't a . . .*

MODERATOR: Y la última parte ya que queda poquito son sobre, qué conocen sobre los antimicrobianos, los antibióticos y las políticas del uso de todos los antibióticos acá en la granja. Bueno ustedes saben, no sé se si saben que los antibióticos son medicamentos que los médicos usan para ayudar a las, a algunas personas con alguna infección bacteriana a mejorarse y que los mismos productos que muchas veces se ocupan en las personas, lo ocupamos en los animales. Y cuál es su experiencia, si tienen alguna experiencia tomando antibióticos.
TRANSLATION: *And then the last part, we only have a little bit left, is about what you know about antimicrobials and antibiotics and the policies about the use of all of the antibiotics here on the farm. Okay, you guys know, I don't know if you know that antibiotics are medications that doctors use to help some people with some bacterial infections to get better and that, many times, the same products that are used on people are used on animals. And what is your experience, if you have any, taking antibiotics?*

MAN: Nosotros no trabajamos eso.
TRANSLATION: *We don't work with any of that.*

MODERATOR: Pero, por ejemplo, si está enfermo, si alguno de ustedes está, ¿cuál es su experiencia tomando ustedes antibióticos?
TRANSLATION: *But, for example, if you are sick, if one of you is sick, what is your experience taking antibiotics?*

MAN: Yo creo que nunca nos enfermamos.
TRANSLATION: *I don't think we ever get sick.*

MODERATOR: ¿Nunca le ha tocado tomar antibiótico?
TRANSLATION: *You've never had to take antibiotics?*

MAN: No.
TRANSLATION: *No.*

MODERATOR: ¿Penicilina o algo, no?
TRANSLATION: *Not like penicillin or something like that?*

MAN: Pues, cuando trae uno infección, es para la infección. Pero no del que hay aquí.
TRANSLATION: *Well, when you have an infection, it's for infections. But not for what is here.*

MODERATOR: No, no, pero la pregunta es así como cuando tiene que ir al doctor o alguna . . .
TRANSLATION: *No, no, but the question is like when you have to go to the doctor or something . . .*

MAN: No.

MODERATOR: ¿No? Son saludables. O de su familia, ¿nadie?
TRANSLATION: *No? You're all healthy. Or anyone in your family or anybody?*

MAN: Cuando tiene gripa.
TRANSLATION: *When you have the flu.*

MAN: Cuando tienen gripa. Cuando anda lo de la garganta que tomas las cápsulas de penicilina, algo así.
TRANSLATION: *When you have the flu. When you have a sore throat and you take the penicillin capsules, something like that.*

MAN: Pero es muy normal, la gripa, ¿no?
TRANSLATION: *But that's normal, the flu, right?*

MAN: La garganta es normal. Cuando se pone pues frio, se cambia el clima, pues, sí, la garganta como . . . pero pues . . .
TRANSLATION: *The sore throat is normal. When it gets cold, the weather changes, well, yeah, like your throat . . . but, well . . .*

MODERATOR: Pero poco, lo usan poco entonces, nunca, así nunca, okay. ¿Y Qué situaciones piensan que los antibióticos puedan ser útiles para ustedes? O si piensan que no sirven.
TRANSLATION: *But little, you use it very little, then, you've never, like never, okay. And in what situation do you think that antibiotics could be helpful for you, or do you think they don't work?*

MAN: Pues, sí sirvan.
TRANSLATION: *Well, yeah, they work.*

MAN: Yo pienso que no sirvan.
TRANSLATION: *I don't think they work.*

MODERATOR: ¿Sí?
TRANSLATION: *Yeah?*

MAN: Yo pienso que no. O sea, esos, te lo calman, pero una gripa casi siempre se quita solo a los, solo a los días. Pero, pues, sí te calma el dolor de cabeza si traes dolor de cabeza y todo. Pero, pues, las están tomando también todo el rato, todo el día, pastilla y pastilla. Yo casi no me enfermo, pero cuando me enfermo si que no tomo nada yo.
TRANSLATION: *I don't think so. I mean, those, they calm the illness, but a flu almost always goes away on its own, just in a few days. But, well, it does help your headache if you have a headache and everything. But, well, then you're taking them all the time, all day long, pill, after pill. I almost never get sick, but when I do, I don't take anything.*

MAN: . . .

MODERATOR: Pero ¿nunca ha estado así como muy enfermo como una neumonía o algo así que necesite?
TRANSLATION: *But you've never been like really sick or anything like with a pneumonia or something that required it?*

MAN: No.

MAN: Hasta ahorita, no.
TRANSLATION: *Up until now, no.*

MAN: Ya para eso, sí, necesita ir uno al hospital cuando tiene uno esa, algo más grave.
TRANSLATION: *And for that, yeah, you need to go to the hospital when you have that, something serious.*

MODERATOR: Y por ejemplo, si necesitan, si piensan que necesitan antibiótico, ¿cómo harían para obtenerlo?
TRANSLATION: *And, for example, if you need, if you think you need an antibiotic, how would you obtain it?*

MAN: Pues, yendo al . . .
TRANSLATION: *Well go to the . . .*

MAN: Al hospital.
TRANSLATION: *To the hospital.*

MAN: Al médico.
TRANSLATION: *To the doctor.*

MAN: . . . .tequilita, ¿no? No ocupamos medicamento.
TRANSLATION: *. . . a little tequila, right? We don't need medicine.*

MAN: Sí, o sea, es más efectivo eso.
TRANSLATION: *Yeah, I mean, that's more effective.*

MODERATOR: Claro.
TRANSLATION: *Of course.*

MAN: Sal y limón.
TRANSLATION: *Salt and lime.*

MODERATOR: Sí. Bueno, lo que pasa es que yo les pregunto estas cosas porque a veces, normalmente, los antibióticos uno los ocupa para mejorar a una persona o un animal, pero a veces no funciona. Dejan de funcionar. Y entonces, y por lo mismo, como no funciona, ya no se pueden recuperar ni las personas ni los animales, y eso se le llama resistencia al antibiótico que la bacteria crea resistencia a pesar de que esté el antibiótico. Sobrevive y sigue la infección y empeora. Y ¿qué piensan sobre la resistencia antibiótico? Si sabían acerca de eso de que ese está pasando y eso.
TRANSLATION: *Yeah. Well, I'm asking you these things because sometimes, normally, a person or an animal uses antibiotics to get well, but sometimes, they don't work. They stop working. And so, for that reason, that they don't work, the person or the animal can't get better. And that is called antibiotic resistance where the bacteria creates a resistance despite the fact that the antibiotic is present. The infection survives and gets worse. And what do you all think about the antibiotic resistance? Did you know about this that this was happening?*

MAN: Eso dice que la resistencia cuando una persona ya se acostumbró al medicamento, ¿no?
TRANSLATION: *That means that the resistance is when a person is accustomed to a medication, right?*

MODERATOR: Mm-hmm.

MAN: Que por más que tomen medicamento, la misma persona sigue enferma porque el mismo . . .
TRANSLATION: *That despite taking more medication, the same person keeps getting sick with the same thing . . .*

MAN: Sí, hasta la bacteria se acostumbró a eso.
TRANSLATION: *Yes, the bacteria got used to it.*

MODERATOR: Claro. Mm-hmm. O sea . . . familiar esto de la resistencia antibiótico, ¿lo conocían? ¿Sabían que pasa?
TRANSLATION: *Of course. Mm-hmm. I mean . . . familiar with antibiotic resistance, did you know about it? Did you know what happens?*

MAN: Pues sí, pero . . .
TRANSLATION: *Well, yeah, but . . .*

MAN: Pues, sí, sabemos desde la escuela.
TRANSLATION: *Well, yeah, we knew about it from school.*

MODERATOR: ¿Nunca les ha tocado o si saben de alguien que estaba tomando un antibiótico y que no le han funcionado y que han empeorado?

TRANSLATION: *Has it ever happened to you or have you known anybody that was taking an antibiotic what didn't work and they got worse?*

MAN: Yo no.
TRANSLATION: *Not me.*

MAN: . . . pasar por eso.
TRANSLATION: *. . . go through that.*

MAN: No lo he visto eso.
TRANSLATION: *I've never seen it.*

MAN: todos estamos saludables ahorita.
TRANSLATION: *We are all healthy right now.*

MODERATOR: ¿Ninguna de ustedes ha tenido como una infección resistente al antibiótico?
TRANSLATION: *None of you guys have had an antibiotic resistant infection?*

MAN: . . . no más la tos o la gripa que dura una semana a veces.
TRANSLATION: *. . . just a cough or the flu, which lasts a week sometimes.*

MODERATOR: Claro.
TRANSLATION: *Of course.*

MAN: Pero no dura mucho. O sea . . . vamos por una tequilita y ya . . .
TRANSLATION: *But it doesn't last long. I mean . . . let's go get a little tequila and that's it . . .*

MAN: Se fue.
TRANSLATION: *It's gone.*

MODERATOR: Y ¿qué piensan que, qué riesgo pueda representar para ustedes o para otras personas que haya infección resistente al antibiótico?
TRANSLATION: *And what do you think, what risk does antibiotic resistant infections pose to you and other people?*

MAN: Que se hacen adictos, ¿no? Se hacen adictos al medicamento. Y ya muchas personas según ya no pueden dejar de tomar el medicamento, aunque no les ayude para nada. Ya como que la adición del mismo medicamento ya no los deja.
TRANSLATION: *They become addicts, right? They get addicted to the medication. And then many people apparently can't stop taking the medication, even though it's not helping them. Like they can't get rid of the addiction to the medication.*

MODERATOR: Y ¿qué otros riesgos puedan ser también, así como para ustedes o para otras personas de que se esté tratando a una persona con medicamento que no sirve?
TRANSLATION: *And what other risks could there be as well, like for you or for other people, by taking a medication that is not working?*

MAN: Pues, hay bacterias que se pueden, se les puede pasar a los demás. Es lo que también, por eso a veces que anda alguien enfermo es muy importante que traiga tapaboca también porque a veces tosen y si hay alguien cortito, entonces va a respirar eso, la persona. Aquí casi siempre cuando anden así, piden el día para descansar. Y, pues, está mejor por una parte que descansen.

TRANSLATION: *Well, there are bacteria that can be passed to others. That's also why, when someone is sick, it's very important that they have a face mask on because sometimes they cough, and if there's someone close by, they could breathe that in. Here, when people are sick like that, they almost always ask for the day off to rest. And, well, in some ways, rest is the best thing.*

MODERATOR: En el fondo es como, porque pueden contagiar también a otras personas si no . . .
TRANSLATION: *So behind that it's because others could catch it if they don't . . .*

MAN: Pues, se siente mal. Se siente mal también, pero es, deberían de andar con tapabocas cuando anden así enfermos.
TRANSLATION: *Well, if they feel bad. They feel bad too, but it's, they should have a face mask on when they are sick.*

MODERATOR: ¿Alguien de ustedes quiere, piensa así que existe algún otro riesgo para ustedes o para otras personas? ¿No?
TRANSLATION: *Do any one of you want, think that there are any other risks for you or other people? No?*

MAN: No.
TRANSLATION: *No.*

MODERATOR: Y, bueno, y ¿qué papel piensan ustedes que juega las granjas lecheras en el tema de la resistencia antibiótico, si piensan que hay alguna relación?
TRANSLATION: *And, well, and what role do you think you guys have on the dairy farm regarding the antibiotic resistance, if you think it's related at all?*

MAN: ¿En el qué?
TRANSLATION: *In the what?*

MODERATOR: La granja lechera en la resistencia antibiótico, si piensan que tiene alguna relación con eso.
TRANSLATION: *The dairy farm and antibiotic resistance, if you think there is a relationship between them.*

MAN: No creo. Quién sabe?
TRANSLATION: *I don't think so. Who knows?*

MAN: No creo porque todo es diferente a veces en las personas y está pasando algo y no trabaja en granja, ¿qué tiene que ver eso? O lo mismo, a un animal, pues, puede ser que, entre los animales, entre las vacas, sí hay algunas, pero, pues, sí, hay animales que se han muerto. Les han puesto antibiótico y no resistan, siguen enfermas y, pero, pues, entre la misma granja, puede ser que sí haiga entre los mismos animales. Los mismo entre las mismas personas y es lo mismo, pero . . .
TRANSLATION: *I don't think so because everything is different sometimes with people, and something is happening and they don't work on the farm, what does that have to do with it? Or same with an animal, well, it could be between animals, between the cows, if there are any, but, well, there are animals who have died. They have given them antibiotics, and they don't resist. They stay sick. And, well, on the farm, it could be that there is between the animals. Between the people, and it's the same, but . . .*

MODERATOR: Sí, así como pensando en que acá igual se ocupan antibióticos y todas esas cosas. ¿No? Y ¿cuáles son las reglas que tiene la granja sobre el uso de antibióticos acá? ¿Quiénes lo

ocupan, cuándo lo ocupan y eso?
TRANSLATION: *Yeah, like think about here, you do use antibiotics and all of those things, right? And what are the rules that you have here on the farm about the use of antibiotics here? Who uses them, when do they use them, stuff like that?*

MAN: No sabemos.
TRANSLATION: *We don't know.*

MAN: Las reglas como . . .
TRANSLATION: *The rules like . . .*

MAN: ¿Para los animales?
TRANSLATION: *For the animals?*

MODERATOR: Mm-hmm. Sí, para los animales.
TRANSLATION: *Mm-hmm. Yeah, for the animals.*

MAN: Ellos son los que se encargan de eso. Lo usan cuando haya una vaca que tiene fiebre de leche o si de allá a veces encontramos alguna vaca que tiene en los pies enfermedad, ¿cómo se dice? infección. Esa la traemos para acá y entonces ellos le ponen medicina para que se le va eso. A veces, ya vienen muy enfermas, y no resuelve el problema. Entonces la tienen que vender.
TRANSLATION: *They are the ones that are in charge of that. They use them when there is a cow with milk fever. Or sometimes, we find a cow that has, what's it called, infection in her feet. We bring that one over and then they give her medicine so that goes away. Sometimes they are really sick, and the problem doesn't resolve. So they have to sell her.*

MAN: ¿Te acuerdas que antes había una que le ponían, y le ponían y también no se le quería quitar que tardó bien harto hasta ya después? Y ya, luego, ya, se acomodó, pero tardó bastante.
TRANSLATION: *Do you remember there was one that they would put on and put on and it too would go away, and it took a really long time. And then after that, it would get better. But it took forever.*

MAN: Sí, no más que esa, esa medicina ocupan de ponerla y tenerlo en cuenta que tiene que durar la vaca 30 y tantos días sin que se venda porque la carne está mala. Deben de llevar el control de eso. Sí, porque si la venden para la carne, entonces va a contaminar.
TRANSLATION: *Yeah, just that that medicine that the give her, you have to keep in mind that the cow has to make it 30 some days without being sold because the meat is bad. They have to get that under control. Yeah, because if they sell the meat, then it will contaminate.*

MAN: . . .

MODERATOR: Y por ejemplo . . .
TRANSLATION: *And for example . . .*

MAN: Aquí no más se les da de comer, y otro día vuelven por ellos. Se los llevan los que nacen. Se les da de comer una vez y se, lo único que se les pone en el ombligo es un líquido para que no agarre infección o algo y es todo. Pero esos se los levan . . . por ellos.
TRANSLATION: *Here we just feed them, and then the next day they come for them. They take the ones that were just born. They feed them once and they, the only thing they put on their navels is a liquid so that they don't get an infection or something, that's it. But they take those . . . for them.*

MODERATOR: O sea que ¿ninguno de ustedes tienen, maneja medicamento?
TRANSLATION: *So none of you guys have, deal with medicines?*

MAN: No usamos nada de eso.
TRANSLATION: *We don't use any of that.*

MAN: No. Todo se encarga el patrón de todo eso, dar medicina y eso.
TRANSLATION: *No. The boss is in charge of all of that, giving medicines and all that.*

MODERATOR: Okay. ¿Qué me falta? Entonces ¿quién en la granja está autorizado para usar antibiótico, serían como que hay un grupo?
TRANSLATION: *Okay. What's left? So, who on the farm is authorized to use antibiotics, is it like a group?*

MAN: Sí.
TRANSLATION: *Yes.*

MAN: Sí hay personas específicas que pueden usar eso. Nosotros, no me puedo meter con los antibióticos.
TRANSLATION: *Yeah, there are specific people that can use that. We, I can't touch the antibiotics.*

MAN: A veces que necesita alguna vaca operación o algo, que trae el estómago torcido, viene un . . .
TRANSLATION: *Sometimes a cow needs some kind of operation or something, if she has a twisted stomach a . . .*

MODERATOR: Un veterinario.
TRANSLATION: *A veterinarian*

MAN: Veterinario hacer eso.
TRANSLATION: *Veterinarian comes to do that.*

MODERATOR: Okay.

MAN: Pero aquí así cositas que no más es calcio o una pastilla, ellos son los que se encargan de eso.
TRANSLATION: *But here it's just things like calcium or a pill, they are in charge of that.*

MODERATOR: Y ¿qué piensan sobre esta regla? ¿Parece que está bien, está mal?
TRANSLATION: *And what do you think about this rule? Does it seem good or bad to you?*

MAN: A mi, me parece bien.
TRANSLATION: *It seems okay to me.*

MODERATOR: ¿Sí?
TRANSLATION: *Yeah?*

MAN: A lo mejor si estaba más pequeño el rancho, a lo mejor nos enseña más, pero no tan grande. Hay demasiada gente.
TRANSLATION: *Perhaps if the farm was smaller, they would teach us more but not this big. There are too many people.*

MODERATOR: Claro. Esas son las preguntas. Eso era todo lo que les tenía que preguntar. No sé si tienen alguna consulta o algo antes de que termináramos.
TRANSLATION: *Of course. Those are the questions. That was all that I had to ask you. I don't know if you have any suggestions or anything before we finish.*

MAN: Pues, no.
TRANSLATION: *Well, no.*

MODERATOR: No fue tan difícil.
TRANSLATION: *That wasn't that hard.*

MAN: Somos gente de pocas palabras.
TRANSLATION: *We are people of few words.*

MODERATOR: No, pero es muy útil. Todo lo que han compartido con nosotros es súper útil para el estudio, así que, muchas gracias.

TRANSLATION: *No, but it's very useful. Everything you have shared with us is super useful for the study, so, thanks a lot.*

Focus Group 8, Farm 5
Recording in English

INTERVIEWER: All right so just to get started, I'd like to know a little bit about what you kind of do on the farm, and what you would say that your role is. So if you could just go back and forth to each of you and just kind of describe your role, how long you've been working here for, and just what other farming experience that you have.

RESPONDENT 1: Let's see, my role is to kind of work with the sick cows in the hospital area there. And I've been here ten years. Treat cows with antibiotics and stuff like that, the mastitis.

INTERVIEWER: Have you been primarily at this farm then?

RESPONDENT 1: Mm-hmm.

INTERVIEWER: Have you worked at another one in the past?

RESPONDENT 1: No, I had my own dairy before I come here.

INTERVIEWER: Okay. That's cool. Nice. And what about you?

RESPONDENT 2: I assist with his role. And I've been here just a little over a year now. And I also had a dairy farm of my own prior to that, so.

INTERVIEWER: So what made you guys switch from your own farm to working here?

RESPONDENT 1: Well, for me, I don't know. Well, I knew Andres, the owner. And then I kind of got lonesome. Cows weren't keeping me company. So I thought, well, I wonder what it would be like to work out? But, yeah, it's good. It's all right. It's different. So I sold the cows and come here. Wanted more time off too.

INTERVIEWER: Yeah. That's nice.

RESPONDENT 1: So, you know, I was working like, well, I had 70 cows, and I did pretty much all of it myself. So it was like 11 to 14 hours a day. I thought maybe I'd have a little bit more time off, and I do. So, yeah, I'm enjoying it.

INTERVIEWER: That's nice. Hi. Are you here for the group?

RESPONDENT 3: Yes.

INTERVIEWER: All right. Yeah, have a seat. So I was telling them a little bit before. We're just talking kind of about, the focus of the study is on antibiotic resistance on farms. And just kind of like how farm workers or that might influence it, and that kind of thing.

RESPONDENT 3: Okay.

INTERVIEWER: So I'm recording the conversation here. There won't be any identifying information or anything like that. We'll remove all of it. And you'll be given $25. Does that sound good?

RESPONDENT 3: Sounds good to me.

INTERVIEWER: All right.

RESPONDENT 3: I'll work with that.

INTERVIEWER: So the first, I was just in the first question anyway. So I'll ask you too, since you just sat down, about kind of what your goal on the farm is and how long you've been here, that kind of thing.

RESPONDENT 3: Yeah. I've been here my whole life . . . yeah. So goal on the farm, we're about making beautiful things around here. So we just, yeah, we enjoy the work we do. We enjoy the people we work with is really the big thing. So, yeah, that's basically the summary of . . .

INTERVIEWER: So what's your primary job?

RESPONDENT 3: Primary job?

INTERVIEWER: Yeah.

RESPONDENT 3: I kind of run around and do a lot of things. I work with treating cows, vaccinating dry cows. We just got done vaccinating the whole herd. I'll do some welding here and there too, so whatever needs to happen.

INTERVIEWER: Sounds good. So I guess next I'm just going to go into learning a little bit more about his farm in particular. So what is a typical shift like for each of you on a day-to-day basis?

RESPONDENT 1: What is the what?

INTERVIEWER: Like a typical shift. So like from the time you get here to the time you leave, what does that look like?

RESPONDENT 1: Oh, well, my typical shift, oh, it's about from 5:00 to 11:00, average, yeah, or noon. Yeah.

RESPONDENT 2: Yeah, and mine is about the same, so.

INTERVIEWER: Yeah. So I guess, what do you guys do if you have any questions about how to do your job, and something comes up throughout the day, where would you go?

RESPONDENT 1: Well, we do have a, what do you say, a veterinarian, I guess. Doctor Pearson(?) kind of over us. He comes like once a month or something.

RESPONDENT 3: Yep, yep. He's here every month.

RESPONDENT 1: Every once in a while, he checks in on what we're doing. So if we have a problem, we can give him a call too.

INTERVIEWER: Oh, okay. So you just have his number then, and . . .

RESPONDENT 1: Yeah.

INTERVIEWER: Okay. When do you typically talk with like your manager then?

RESPONDENT 1: Hmm?

INTERVIEWER: Like what kind of situations lead you to . . .

RESPONDENT 1: Oh, well, we don't call him too often because we have all our protocols kind of written down on what we're supposed to do. So it, I don't know, basically be a cow that's really stressed out and can't really figure out what's wrong, I guess. But don't seem to happen too often, yeah.

RESPONDENT 3: He'll take manure samples and . . .

RESPONDENT 1: Yeah.

RESPONDENT 3: Sometimes he'll, if she's got stomach bugs usually seem to be the common things, so we'll ask questions about that.

RESPONDENT 2: And otherwise, we're in contact with the owners. They're here all the time. So we're always speaking with them.

RESPONDENT 3: Andres usually has some opinions on what to do, and so forth.

INTERVIEWER: Yeah. So I guess, what is your relationship like with the managers around here?

RESPONDENT 2: Real well.

INTERVIEWER: Pretty good?

RESPONDENT 2: Yeah.

RESPONDENT 1: Yeah. Real well.

INTERVIEWER: Are there any situations in which like your managers would come talk to you?

RESPONDENT 1: Oh, yeah, we communicate just about every morning or every day, look at the cows together with them, yeah, try and figure out where to go from there.

RESPONDENT 3: Yeah, Sue is the herd manager. And she always is, every morning, she runs the list of the sick pen and . . .

RESPONDENT 1: Which, she kind of picks the cull cows and stuff. Points out some sick ones and all that stuff, so, yeah. Kind of a team effort. If one don't see it, somebody else sees it, or something, I don't know.

INTERVIEWER: No, that's good though. Always having extra eyes out and checking.

RESPONDENT 3: Yeah. We had cull cows organized yesterday. And it's like removing other cows into a milking pen, and it's like, wait a minute, a cow was mixed up between the two of them.

RESPONDENT 1: Yeah, I kind of, I'm getting older, so I kind of find myself going like, oh, yeah, I did, that, did that, did that. And then maybe once in a while, oh, did I do that, did I do that?

RESPONDENT 3: Well, between Greg and Sue and myself, we figure it out. We get it organized.

INTERVIEWER: So how do breaks works on this farm? Like when do you get your breaks, and when can you take them?

RESPONDENT 1: Our breaks, well, we take a break when we get the chores done.

INTERVIEWER: Yeah.

RESPONDENT 1: I don't know, yeah, we break at 11:00.

INTERVIEWER: Is it about the same time every day then?

RESPONDENT 2: Yeah.

INTERVIEWER: Yeah. How long is your break, typically?

RESPONDENT 1: I don't know. Well, we don't really take a break, you know what I mean? I used to take breaks when I worked until 5:00. But I only work six hours or seven, so I don't really take a break. I guess we're supposed to. I don't know.

RESPONDENT 3: Well, yeah, you guys wash the screens up in the manure room and stop for lunch at that point.

RESPONDENT 1: Yeah.

RESPONDENT 3: Or you'll do that, you'll go home.

RESPONDENT 2: Yep.

RESPONDENT 1: Oh, yeah. And then, yeah, when we're washing the parlor, we get a little break. And so look around at what's left to do and stuff. Yeah.

INTERVIEWER: So where would you typically eat lunch then?

RESPONDENT 1: I usually eat in my truck.

INTERVIEWER: In your truck?

RESPONDENT 3: Or there's a break room.

RESPONDENT 1: Yeah, there is a break room, yeah.

INTERVIEWER: And you said you leave sometimes?

RESPONDENT 2: Yeah, I'm so close to home that I'm only two miles away.

INTERVIEWER: Do you usually go home then for lunch?

RESPONDENT 2: Yeah.

INTERVIEWER: I gotcha. All right. And like as far as bathroom breaks or whatever, you just take those as needed, just short . . .

RESPONDENT 3: Yeah.

RESPONDENT 2: Yeah.

INTERVIEWER: So I think now I'm going to kind of get into some questions kind of about your daily work here and the clothes that you wear and some of the protection equipment. Does your farm have any rules on what you can wear on a daily shift?

RESPONDENT 3: Common sense, mostly.

RESPONDENT 2: Yes, right.

INTERVIEWER: Kind of up to you and what's most comfortable.

RESPONDENT 3: Yeah. There's no set rule like steel-toed boots or anything like that.

INTERVIEWER: Okay. Are there any times you would wear some sort of protective gear, either boots, shoe covers, gloves eye protection?

RESPONDENT 1: Well, we wear gloves . . .

RESPONDENT 2: Yeah, we always . . .

RESPONDENT 1: Right when we get here, basically, to milk and stuff. We wear the gloves.

INTERVIEWER: Is that the only kind of protection wear that you see around here?

RESPONDENT 1: Mm-hmm. About all I wear, yeah.

INTERVIEWER: Okay.

RESPONDENT 1: And then I've got my glasses, so.

RESPONDENT 3: Yeah, there's, when you guys are washing screens, we'll put gloves on for that or glasses on for that too. It gets a little splattery.

INTERVIEWER: Yeah, I could see that.

RESPONDENT 3: Yeah, for sure.

INTERVIEWER: So I guess, you talked about the gloves and stuff, were there situations where you have to change gloves between doing things, or is it just kind of, you throw them on when needed, and . . .

RESPONDENT 2: I only change them when they rip.

RESPONDENT 3: Yep.

INTERVIEWER: Okay. Gotcha. Do you wear any sort of different clothing or protective gear when you're dealing with calves that are sick or any other cows that are sick?

RESPONDENT 3: No, not usually. I've got a set of Udder Tech bibs. I'll put those on, but, if I'm out in the pens, but, no, it's just the normal clothes every day.

INTERVIEWER: Gotcha. And do you kind of like wear the same clothes home or . . .

RESPONDENT 3: Yeah, I do.

INTERVIEWER: Do you ever do laundry around here, or is it mainly just wear what you come in with and leave?

RESPONDENT 1: Yeah, you do.

RESPONDENT 3: I do laundry here.

RESPONDENT 2: Yeah.

RESPONDENT 3: Do you do laundry here?

RESPONDENT 2: I don't, but.

RESPONDENT 3: I know you started washing clothes.

RESPONDENT 1: No, I, no I don't.

RESPONDENT 3: Well, it's available.

RESPONDENT 1: Yeah.

INTERVIEWER: It's available if you need it.

RESPONDENT 1: Yeah.

RESPONDENT 3: But, yeah, it's kind of easier just to go home and wash it, generally speaking.

INTERVIEWER: Especially for you I'm sure because you live so close.

RESPONDENT 2: Yeah.

INTERVIEWER: So, I mean, I guess you talked about the gloves and stuff like that. Are there times where you think that you should where them where it's been difficult to throw them on or you never, like you just decide not to put them on?

RESPONDENT 3: There will be times when I'll take the gloves off. Like I use my fingers to check how warm the milk is. So I'll take the gloves off to check that, you know, when I'm feeding a calf. But normally, I just leave them on, so.

INTERVIEWER: Okay. And you're fine with wearing the gloves?

RESPONDENT 1: Mm-hmm.

RESPONDENT 2: Yeah. I've been wearing them all winter long, whether I'm here or not, keep my hands.

INTERVIEWER: Right.

RESPONDENT 3: Yeah. No, those gloves are nice.

RESPONDENT 2: Yeah.

RESPONDENT 3: Really nice to have some of those things, so.

INTERVIEWER: So what are your hand washing practices while at work?

RESPONDENT 2: I don't know, I guess they're, most of the time rinsing them off quite frequently.

RESPONDENT 3: Yeah, I'll rinse them off. I do a lot of work with calves. So every time I, you know, if I'm checking the milk, I'll always wash my hands first before then. Any time I'm getting ready to deal with a calf, I always, or deal with the milk, I try to wash. So that's pretty standard. But other times, when we're treating cows, they're in the parlor or out in the pen, it's kind of hard to run back in and wash and run back out.

INTERVIEWER: Yeah. Yeah.

RESPONDENT 2: And then we have the towels. We use them a lot too.

RESPONDENT 3: Yeah. The towels are kind of like confetti. They're everywhere.

RESPONDENT 2: Clean up with, yeah.

INTERVIEWER: . . . yeah. So I guess, what kinds of things are available to you to wash your hands? You know, soap, sanitizer, those kinds of things. What do you usually use?

RESPONDENT 2: Yeah, there's soap dispensers.

RESPONDENT 3: Does anyone know what the pink stuff is called?

RESPONDENT 1: No.

RESPONDENT 2: I know it's kind of a natural . . .

RESPONDENT 3: Yeah, it's the standard, farm, pink soap stuff.

RESPONDENT 1: Yeah, is it a dairy soap? It must be. For utensils?

RESPONDENT 3: I think so. Yeah, it's pretty powerful. But it's gentle enough on the hands. I've never had problems with it, so.

INTERVIEWER: And is that kind of just like a regular soap then that you still just wash off and?

RESPONDENT 1: Mm-hmm.

RESPONDENT 3: Yeah, it works the same. We use it for washing bottles, washing hands, washing buckets. So, yeah, it's available in the milk house and the calf room, so.

INTERVIEWER: Okay. So I guess, are there specific times where you're more likely to go wash your hands?

RESPONDENT 1: Well, you mean when you don't have gloves on or have gloves on?

INTERVIEWER: Or just in general. Anything that you do that you just, you know, afterwards, you feel like you need to go wash your hands?

RESPONDENT 2: Donut day.

RESPONDENT 3: Yeah.

RESPONDENT 1: When you get done checking a cow with a bad uterus, and you want to go eat a donut.

INTERVIEWER: That's fair.

RESPONDENT 1: Or a diarrhea or something. I guess, sometimes I just wipe them on a rag I guess.

INTERVIEWER: Yeah. Now, have your managers given you any instructions on hand washing when they want you to do it, anything like that?

RESPONDENT 1: No, how often to do it?

INTERVIEWER: Yeah, or just like when they'd want you to do it, any sort of instructions or requirements passed on from them?

RESPONDENT 1: No. I haven't really heard anything on that.

INTERVIEWER: No.

RESPONDENT 1: When we treat a cow that's got that disinfectant in it, doesn't it? I mean, I could probably learn to wash my hands a little bit better before I do that. But usually, I put them in the disinfectant water and then . . .

RESPONDENT 3: Oh, oh, yeah, with the bucket when you do an IV.

RESPONDENT 1: To IV the cow, I don't usually go to the faucet and wash my hands.

RESPONDENT 3: But you've got that bucket of Nolvasan. Just kind of run that before you fill the needle. That's pretty standard.

RESPONDENT 1: Should be all right, huh? I don't know.

INTERVIEWER: I guess, does hand washing impact your work at all? Do you notice, you know, the time taking up to do it or anything like that?

RESPONDENT 2: No.

RESPONDENT 3: No, it's, no. Life works a little bit better when you have clean hands. You deal with, like you said, you're dealing with stinky, messy, cows. It's kind of hard to go from taking notes to dealing with that. So anytime, as much as we can basically, I think is kind of the . . .

RESPONDENT 1: But, yeah, when we were down in the parlor, I guess, now you're wanting to know how many times we wash our hands. That is a good question because when I'm dipping cows and stuff, I probably spray my hands off 10, 20 times a day. Because they're full of tit dip and poop and stuff. I don't go to the sink and get soap but just water, clean them up with water.

RESPONDENT 2: And then the rags are there, the towels or whatever.

RESPONDENT 1: And then wipe off with a cow towel and go again.

INTERVIEWER: Okay. So how do you typically get ready to go to work for the day, what's your routine?

RESPONDENT 3: Well . . .

RESPONDENT 2: I eat a bowl of Cheerios. Well, you get here pretty early. Like you start milking at quarter to 6:00, but you've got kind of a system worked out before you get started.

RESPONDENT 1: What do I do? I don't know what I do. Put wood in the stove.

RESPONDENT 3: Oh, yeah.

RESPONDENT 1: I don't know, I don't do much of anything.

INTERVIEWER: So you guys just basically get fully ready at home and then come to the farm, ready to work?

RESPONDENT 1: Oh, yeah.

RESPONDENT 2: Pretty much.

RESPONDENT 3: Yeah, that's exactly it.

INTERVIEWER: Is there anything you do once you get to the farm to prepare for the day, or is it just come in right away?

RESPONDENT 1: I can't think of anything.

RESPONDENT 3: Just get the parlor ready.

RESPONDENT 1: Yeah, but I don't know if that’s considered work or not, or part of the work or what?

INTERVIEWER: Right. Getting that ready, versus, yeah, I guess I’m more talking about like your personal prep before you start.

RESPONDENT 1: Oh, personal prep. Yeah, I just make sure I’ve got enough clothes on, so I don’t get cold. And I’ve got my boots on, so your feet don’t get wet. That’s about it.

INTERVIEWER: Is there any like clothing stuff that you usually leave here, or is it all taken back with you?

RESPONDENT 1: I pretty much take mine.

INTERVIEWER: Like boots and everything? Okay. Do you ever like spray them off or anything before you go home?

RESPONDENT 3: Oh, yeah.

RESPONDENT 1: Mm-hmm.

RESPONDENT 2: Yep.

RESPONDENT 3: Yeah. But we don’t disinfect them. That’s never been a thing. But, yeah, we’ll always spray them off.

INTERVIEWER: Yeah, just like a hose or something, and . . .

RESPONDENT 3: There’s a hose in the milk house.

INTERVIEWER: Okay. Gotcha. So I’m going to move a little bit into talking very briefly about raw milk on the farm. How are raw milk products, I couldn't say that, how are they handled on the farm?

RESPONDENT 1: Raw milk. Well, they come from the cow and go into the bulk tank through the pipeline, I guess.

RESPONDENT 3: Yeah, we’ve had enough experiences with the cooler not being turned on that everybody knows to always check and make sure that the temp is reading right. So we’re always good about checking that. Let’s see.

RESPONDENT 1: Make sure the bulk tank gets turned on.

RESPONDENT 3: Exactly. Exactly.

RESPONDENT 1: Cool it down right away. That’s about the way she goes, I guess. Get it in the tank and cooled down.

RESPONDENT 3: We always keep the, no one is dipping, well, sometimes we’ll take like a milk sample to test for mycoplasma, you know, that’s like a weekly thing. But other than that, no one is dipping into the milk. It’s pretty clear. Nobody is dunking their fingers in there or something like that.

INTERVIEWER: Yeah. All right. So are those kind of like the overall rules would you say as far as how you handle the milk, then checking the temperature and all that stuff? Is there anything else that would be like a rule for handling raw milk?

RESPONDENT 3: I guess . . .

RESPONDENT 2: I mean, the udder prep and that kind of stuff. I mean, take good care in that.

RESPONDENT 3: Yeah, that's pretty clear. Pretty good about that. Sometimes we will, I guess the other thing, sometimes we milk a cow in the pen. And, you know, then we've got a separate little milking thing for doing that and milk into a stainless-steel bucket. And just it's never been articulated, necessarily, but it's always clear, you know, use a clean bucket. And when you introduce it back, don't just dump into the tank. We try to run it through the pipeline, so it goes through the sock and all that. So I guess that's the only other real obvious thing.

INTERVIEWER: And then what do you think about these rules?

RESPONDENT 1: Seem to be all right.

RESPONDENT 3: It's kind of common sense.

RESPONDENT 1: Seem to be good.

RESPONDENT 2: Yeah.

RESPONDENT 3: Common sense.

INTERVIEWER: Do you or anyone else that you know about on the farm ever consume raw milk products?

RESPONDENT 2: We used to years ago.

RESPONDENT 3: Yeah, you used to farm. You had your own herd. I did for a while. I haven't taken milk from the tank in a long time, so.

INTERVIEWER: Does anyone from this farm, do you know of, consume the raw milk?

RESPONDENT 3: Yeah, the managers do, Andres and Sue. They'll drink some milk. They'll take a bucket with them, not a bucket, but, you know, a jug.

INTERVIEWER: Okay. So that's all I had really on raw milk. I'm going to move a little bit into the antibiotic stuff I was talking about from before. So antibiotics are medicines that help doctors, that doctors use to help a person who has an infection with bacteria get better. And veterinarians also use these on animals with infections to help them as well if they get sick. What are your guys's personal experiences with using antibiotics? Have you ever taken any?

RESPONDENT 2: Yeah, I guess I have. Not a lot.

RESPONDENT 3: You had a snotty nose earlier this year.

RESPONDENT 2: Yeah, I didn't get any, and I should have.

RESPONDENT 3: Oh, you didn't get any?

RESPONDENT 2: No.

RESPONDENT 3: Oh, really?

RESPONDENT 2: No.

RESPONDENT 3: They let it slide.

RESPONDENT 2: But I had a finger infection. I had them then for a while. Oh, and I suppose strep throat, you get them then, don't you?

RESPONDENT 1: Can't remember if I've ever took one, tell you the truth.

RESPONDENT 3: Yeah, it's been a while since I've, if I've ever had antibiotics.

RESPONDENT 1: Antibiotics.

RESPONDENT 3: Maybe when I was a little kid or something, but that's about it.

INTERVIEWER: I gotcha. So in your experience, I guess, like what situations are antibiotics helpful, and are there any situations where they're not helpful, as far as what you guys know?

RESPONDENT 3: For humans or for cattle?

INTERVIEWER: Either one actually. Just any personal experience where they're either, you know, have been beneficial, or it just wasn't.

RESPONDENT 2: I don't know there's always a time and a place for them, so, I mean . . .

RESPONDENT 3: Yeah, we use them. We have protocols for using antibiotics. And we use them. I don't think we're excessive by any means. But mastitis and metritis and retained placentas and that kind of stuff, yeah, that's common. And it helps, it does. We've had a lot of animals where we've treated them with any one of those things, and they've come roaring back with plenty of milk. Yeah, we've, it's been good for us.

INTERVIEWER: And then what about for humans?

RESPONDENT 2: It's kind of the same thing. I mean, there's a place for it.

RESPONDENT 3: Yeah, yeah, it's kind of like, if you catch it, yeah, it's just like the cows. If you catch it early enough, it will actually do some good. Otherwise you, we all know that guy that kind of brushed off his swollen finger, infected finger, oh, no don't worry about it. Like Jim, yeah, he's got diabetes and all that, and he, have you met Jim Halbern(?)?

RESPONDENT 2: I think so, yeah.

RESPONDENT 3: He let his toe go too far.

RESPONDENT 1: Oh, really?

RESPONDENT 3: Yeah, they ended up cutting it off.

RESPONDENT 1: Oh, no. His finger?

RESPONDENT 3: His toe, actually.

RESPONDENT 1: Oh, God.

RESPONDENT 3: Yeah, he lost it, well, Jim . . . he lost his fingers too. So he's, yeah, I mean, he's been, taking him apart piece by piece. But anyway, so all I have to say is it's one of those things, if you use them right, and you use them early, it's not a miracle drug.

RESPONDENT 1: Yeah, I believe that antibiotics work. I mean, I've seen it in the grandkids. It seems like . . . you know, with this bad cold or something, and then they seem to get better, so I don't know.

RESPONDENT 2: Well, even like when my, you know, talking about my daughter got it, and she went and got antibiotics, and hers was over within a week.

RESPONDENT 3: She have strep throat or something?

RESPONDENT 2: No, she just, she had a bad cold like in December, and she went in and got some. Of course, she's a nurse. And she was over hers in a week. Mine lasted a month and half. So I should have went in.

RESPONDENT 3: So there's a time. There's a time and a place for them. My goodness.

RESPONDENT 2: Yeah. And I ended up with worse stuff.

RESPONDENT 3: Yeah, that was last November, wasn't it?

RESPONDENT 2: I had one in November. And then I got another one two days after Christmas. So I had two of them. The one two days after Christmas, the one that lasted a month and a half, and then that's when I ended up with those dizzy spells. And I had more doctor visits.

RESPONDENT 3: That's right. You couldn't hardly stand for a couple days.

RESPONDENT 2: Yeah.

RESPONDENT 3: So sometimes . . .

RESPONDENT 2: So I kind of wished I had went in.

INTERVIEWER: So if you needed antibiotics, where would you go to get them?

RESPONDENT 2: As far as on ourselves?

INTERVIEWER: Yeah, for you guys first.

RESPONDENT 2: You have to go to the doctor and get it prescribed.

RESPONDENT 3: Yeah.

INTERVIEWER: And you guys all said that you haven't had them recently, right?

RESPONDENT 2: Mm-hmm.

INTERVIEWER: Okay. So one more short thing to read here. So antibiotics sometimes stop working and are no longer able to kill or control the growth of bacteria in a person or an animal. Because of this, the specific antibiotic might not work anymore to treat the person's infection. This is called antibiotic resistance. So another way to say this is that antibiotic resistance happens when bacteria can resist the effect of the drug and continue to grow even when the drug is being taken. So it's just no longer helpful.

RESPONDENT 3: The drug evolves beyond . . .

INTERVIEWER: Like I said, it can affect both humans and animals. So what are your guys's thoughts kind of on antibiotic resistance? Is this something that you've kind of heard of before?

RESPONDENT 3: Yeah, and like you said, Dr. Pearson has been, done a lot of work with us developing protocols. And that's one of the reasons why we don't just dump antibiotics into an animal for days and days and days. It's five days, and if she's not better, we need to make a decision. So and that's part of the reason that we don't want to keep that kind of mess around here.

INTERVIEWER: Any other thoughts on resistance?

RESPONDENT 1: Well, I don't think there's any resistance around here, like Asher(?) said because, you know, a new cow comes, she'll get five days, and it wouldn't be like her taking this Polyflex and then another antibiotic and another and get resistance to it. She's only on it for a short time. And if she don't recover, well, then we just try and baby her in other ways, you know. So, yeah, I don't think there's any resistance to antibiotics. Do you?

RESPONDENT 3: No. I don't think so. And, yeah, we just, we try not to let it become a thing, so.

RESPONDENT 1: And I don't think I have any resistance because I don't know if I've ever took any.

RESPONDENT 3: Natural resistance.

RESPONDENT 1: So if I went to the doctor, any one of them should work, right? Isn't that what they do when you take one, and it don't work, they have another little different choice?

INTERVIEWER: Yeah. A lot of times, if one doesn't work out, they'll have other options. Yeah. There's like a whole . . .

RESPONDENT 1: But maybe one won't even work the first time. Is that such a thing? I mean, I don't really know.

INTERVIEWER: It depends on the infection, I would say. Yeah.

RESPONDENT 3: Case dependent.

RESPONDENT 2: I suppose it's even like a flu shot. They're guessing what's going to come. And if they get it right, it works. And if they don't, it doesn't.

INTERVIEWER: And have any of you or a loved one, someone that you knew, ever had an antibiotic-resistant infection where, you know, one of them just didn't work?

RESPONDENT 3: No, no, not myself.

RESPONDENT 2: Not that I know of.

RESPONDENT 1: I don't know of any.

INTERVIEWER: I mean, have you heard of like MRSA and like that kind of thing, where it's, yeah. So those are ones you see a lot in like hospitals. It's just like a resistant organism that no longer really responds to certain antibiotics. That's just like an example I was thinking of.

RESPONDENT 3: Yeah . . . the hospital, one of the hospitals in town has had a few cases of that. And I've heard stories, but I don't know anyone personally that's had to deal with it, so.

INTERVIEWER: Which is a good thing, right?

RESPONDENT 3: Yeah, definitely. It's kind of a scary thing.

INTERVIEWER: When you talk about it kind of, you know, being scary, what risks do antibiotic-resistant infections kind of pose to you or other members of the community?

RESPONDENT 3: Well, like Steve was saying, I don't think we have any bugs or anything that, any bacteria that's really antibiotic resistance, resistant. So the only risk is not much more than the general public. So I don't think it is. I mean, if you could get, if you have about as much a chance of picking up something like that at the farm as you do going to the hospital, it's kind of hard to justify changing your lifestyle.

INTERVIEWER: Any other thoughts from you guys?

RESPONDENT 1: Huh? Oh, on what, about the . . .

INTERVIEWER: Just like resistance, like the risk of resistance in the community or for you guys personally.

RESPONDENT 1: No. I'd say not a risk. Not much of a risk.

RESPONDENT 3: Definitely not something you think about a lot.

RESPONDENT 1: Nothing that's definitely worried me, I guess.

INTERVIEWER: Yeah, I gotcha. So I guess, what role, if any, do you think dairy farms have in antibiotic resistance?

RESPONDENT 2: Well, I mean, I guess all dairy farmers have a responsibility not to overdo it, so.

RESPONDENT 3: Yeah, it just, it makes sense.

RESPONDENT 2: Kind of like common sense.

RESPONDENT 3: You know, why would we dump new drugs down the drain if it's not helping?

RESPONDENT 2: And they're expensive too. So if you don't have to use it, you don't want to.

RESPONDENT 3: Right.

RESPONDENT 1: Mm-hmm.

INTERVIEWER: So you would say like the farm really isn't affected by resistance at all at this point?

RESPONDENT 2: No.

RESPONDENT 1: No.

RESPONDENT 3: No, most of the cows respond pretty well. Normally, if someone doesn't respond, it's because they're really struggling as a fresh cow. Like somebody that maybe they had a really, really hard calving. We had one, number 64, and she was just down and out. Or it's really bad mastitis, a repeat case, and they've got a bad teet end and other problems, so, yeah.

INTERVIEWER: I guess, how do you think that dairy farms that are like this one might be affected by antibiotic resistance?

RESPONDENT 1: Hmm.

RESPONDENT 3: Well, if they, it might be a fluke, or they might, you know, just be a fluke, happenstance where they get some sort of antibiotic-resistant bug. Or maybe they are kind of irresponsible with their drugs, or that might be a thing too. Keep treating a cow for two weeks at a time, it's not going to bode well for anyone.

RESPONDENT 2: Well, and then you don't want to go over the recommended dose either.

RESPONDENT 3: Yeah, if you're not using the drugs properly, that could make problems.

INTERVIEWER: So I guess you talked a little bit about the current rules on the farm about the antibiotic use, like the dosing and days. So I guess, who on the farm is allowed to use antibiotics on the animals?

RESPONDENT 3: These guys. Greg and Steve. And the managers, Andres and Sue. They will. But, yeah, so anytime, you know, there's a protocol.

RESPONDENT 2: Well, and then that's another thing you do good. You keep that in a separate area, contained.

RESPONDENT 3: Yeah, we have a separate medicine room.

RESPONDENT 2: And then a separate place where the cattle are treated, and you don't do it up in your main, you don't do any treating in the main milking center.

RESPONDENT 3: Yeah. Yeah, that's a good point.

RESPONDENT 2: You keep it, you know . . .

RESPONDENT 3: Everything is right here.

INTERVIEWER: So if any of the cows and stuff get sick, do you kind of separate them off from the rest of the group for the duration of the treatment?

RESPONDENT 3: Yeah, yeah, we have, there's a separate parlor down here. And there's a separate pen over there. And then anyone who has got antibiotics in their system, they get red bands, and they're put in that pen over there. So it's very, very clear. It's very rare that someone slips by, that we get a hot tank that's not normal.

INTERVIEWER: Gotcha. So with the overall rules for the use of antibiotics, how do you feel about the rules?

RESPONDENT 3: Good.

RESPONDENT 1: I feel the rules are good.

RESPONDENT 3: Common sense.

RESPONDENT 1: Well prescribed.

RESPONDENT 3: We've been doing it for so long, ten years, so that's, ten plus years.

RESPONDENT 1: I don't know what else to say about it, but, yeah.

INTERVIEWER: Well, that's all the questions I have for you. Is there anything else you guys want to talk about as far as either antibiotics or just stuff on the farm that you've thought of after like I asked? Any final thoughts I should say.

RESPONDENT 1: I don't know, not really, I guess. I'm thinking.

RESPONDENT 3: Yeah, common sense is kind of the rule of law. There is good communication between us and the managers, so it's, yeah.

INTERVIEWER: Good. All right.

Focus Group 9, Farm 6
Recording in Spanish

MODERATOR: . . . otra aquí. Ya. Bueno, entonces, de nuevo, les cuento que la idea es que no me compartan nombres, ni suyos ni de su compañero. La idea es que cada uno hable una vez, una persona a la vez, no todos juntos. Si pueden silenciar sus celulares o algo. Pero si, por ejemplo, quieren tomar una llamada, me dicen, y pueden tomarla sin ningún problema, o si quieren ir al baño también, si quiere tomar un break. Para empezar, me gustaría saber un poco sobre de ustedes, de cual es su rol o su función acá en la granja, que tipo de trabajo hacen, por ejemplo. Y si no, o sea, así podemos ir como cada uno que me cuenten un poco qué es lo que hace en la granja, cuánto tiempo lleva acá trabajando, y si tiene alguna experiencia en otra granja. ¿Quien le gustaría empezar? ¿Acá?
TRANSLATION: *. . . another one here. Okay. Well, so, again, I'll tell you that the idea is to not share names, not yours nor your peers'. The idea is that everyone speaks once, one person at a time, not everyone together. If you can silence your cellphones or something. But if, for example, you guys want to take a call, tell me, and you can take it with no problem, or if you want to go to the bathroom also, if you want to take a break.*
To begin, I would like to know a little more about you guys, what your role or function is here on the farm, what type of work you guys do, for example. And if, I mean, we can go like each one of you tell me a little what it is that you do on the farm, how long you've been working here, and if you have any experience on another farm. Who would like to start? Here?

MAN: Mi labor en el rancho es, pues, un poquito de casi de todo, asistir en vacas que están en la maternidad, un parto, vaccinación de vacas, tratamiento de vacas enfermas, observación, y monitoreo del grupo de vacas frescas. Un poco de cuidado de los pies, de los hooves de las vacas, un poco ya. Tratamos vacas que tienen mastitis aquí en el hospital. También ordeño, un poco de inseminación también. Y también laboral general, que se necesita, las camas de las vacas . . . cómo está todo eso. Uno empieza aquí, allá.
TRANSLATION: *My work on the ranch is, well, a little of almost everything, attending cows that are in maternity, a birth, cow vaccinations, treating sick cows, observation, and monitoring the group of fresh cows. A little bit of feet care, of the cows' hooves, a little now. We treat cows that have mastitis here in the hospital. I also milk, a little insemination as well. And also general labor, whatever is needed, the cows' beds . . . how all that is. You start here, there.*

MODERATOR: Okay. ¿Cuánto tiempo lleva trabajando acá?
TRANSLATION: *Okay. How long have you been working here?*

MAN: Pues, 18 años.
TRANSLATION: *Well, 18 years.*

MODERATOR: Dieciocho años. ¿Y antes, tenía experiencia en otros lados?
TRANSLATION: *Eighteen years. And before, did you have experience in other places?*

MAN: Absolutamente nada.
TRANSLATION: *Absolutely nothing.*

MODERATOR: Nada. Okay. Ya, que bien . . . la experiencia también sirve.
TRANSLATION: *Nothing. Okay. All right, good . . . experience also works.*

MAN: Lo que es ordeñando.
TRANSLATION: *Milking.*

MODERATOR: Mm-hmm, sí.
TRANSLATION: *Mm-hmm, yes.*

WOMAN: Nada más en el parto y ordeñar.
TRANSLATION: *Just in birth and milking.*

MODERATOR: Ordeña. Okay. ¿Cuánto tiempo lleva acá?
TRANSLATION: *You milk. Okay. How long have you been here?*

WOMAN: Tres meses.
TRANSLATION: *Three months.*

MODERATOR: ¿Y tiene experiencia en otra granja?
TRANSLATION: *And do you have experience in another farm?*

WOMAN: Sí, cuatro años en otro rancho.
TRANSLATION: *Yes, four years on another farm.*

MODERATOR: ¿Y también con vacas lecheras?
TRANSLATION: *And also with dairy cows?*

WOMAN: Sí, como lo mismo, ordeñar.
TRANSLATION: *Yes, like the same thing, milking.*

MODERATOR: Ordeñar. Okay. Perfecto.
TRANSLATION: *Milking. Okay. Perfect.*

WOMAN: Pues, mi función aquí es, llevo 14 años trabajando aquí. Estuve puchando vacas, ordeñando, y ahorita estoy en las becerreras. Tratamos las becerras de neumonía . . . de diarrea, y observarlas . . .
TRANSLATION: *Well, my job here is, I've been working here for 14 years. I was leading cows to milk, milking, and right now I am in the nurseries. We treat the calves for pneumonia . . . diarrhea, and observe them . . .*

MODERATOR: Mm-hmm, ¿y tiene experiencia en otra granja?
TRANSLATION: *Mm-hmm, and do you have experience on another farm?*

WOMEN: No.

MAN: Yo . . . como él. Las vacas frescas, curarlas en el hospital de mastitis. Y vacunando también, ordeñar también, sé cómo ordeñar. Donde nos pongan ahí hemos trabajado. Poco en las becerreras, no mucho, pero un poco. Es todo lo que hago yo aquí.
TRANSLATION: *I . . . like him. The fresh cows, cure them in the hospital of mastitis, and vaccinating as well, milking also, I know how to milk. We've worked where they put us. A little in the nurseries, not a lot, but a little. That's all that I do here.*

MODERATOR: ¿Y cuánto tiempo lleva acá?

TRANSLATION: *And how long have you been here?*

MAN: Aquí alrededor de siete años.
TRANSLATION: *Here around seven years.*

MODERATOR: ¿Y antes había trabajado?
TRANSLATION: *And had you worked before?*

MAN: Nueve años en otra granja.
TRANSLATION: *Nine years on another farm.*

MODERATOR: ¿Acá en Wisconsin?
TRANSLATION: *Here in Wisconsin?*

MAN: Sí.
TRANSLATION: *Yes.*

MAN: Yo tengo 13 años, soy ayudante del herdsman, trabajo en la noche, preparamos las vacas enfermas cuando . . . de fiebre de leche, mastitis de agua, no más las preparamos y ellos se encargan de . . . nosotros no más las dejamos preparadas porque . . . a llegar al turno del día. Trabajé cinco años en la becerrera. Estuve trabajando con el dueño del rancho, pero de allí me requirieron para venir acá a la granja grande. Parteo vacas, insemino vacas. Más que nada, es como más trabajar como ayudante de herdsman, pues, y labor también. Si necesita puchar, también pucho. El ordeño cuando se requiere, al ordeñar vacas, también, pero muy poquito.
TRANSLATION: *I've been here 13 years, I'm a herdsman's assistant, I work at night, we prep the sick cows when . . . milk fever, water mastitis, we just prep them and they're in charge of . . . we just leave them prepped because . . . the day shift arrives. I worked five years in the nursery. I was working with the owner of the farm, but from there, they required me to come here to the big farm. I assist the cows' births, inseminate cows. More than anything, it's like more working as a herdsman's assistant, well, and labor as well. If pushing is needed, I also push. Milking, when it's needed, milking cows too, but very little.*

MAN: Yo tengo 19 años trabajando aquí en este rancho y, pues, soy ayudante de todo aquí. Ayudo en lo que puedo. Mi trabajo es ser ayudante de todo, trabajo con el veterinario, con el mánager, con los herdsmen, con los ordeñadores, con los puchadores, con los becerreros, con cada quien, pero mi enfoque es de la, en el área de vacas y también soy del grupo de los inseminadores aquí. Hago trabajos generales . . . soy herdsman.
TRANSLATION: *I have been working here on this farm for 19 years and, well, I'm an assistant for everything here. I help in what I can. My work is to be an assistant for everything, I work in the veterinarian's office, with the manager, with the herdsmen, with the milkers, with the pushers, with the calf caretakers, with everyone, but my focus is in the area of cows. And I'm also part of the group of inseminators here. I do general jobs . . . I am a herdsman.*

MODERATOR: ¿Cuánto tiempo lleva acá?
TRANSLATION: *How long have you been here?*

MAN: Diecinueve años.
TRANSLATION: *Nineteen years.*

MODERATOR: ¿Y antes había trabajado en otro lado?
TRANSLATION: *And had you worked anywhere else?*

MAN: No.

MODERATOR: No. Okay.

MAN: No, aquí fue mi primer rancho y, pues, aquí estoy todavía.
TRANSLATION: *No, this was my first farm and, well, I'm still here.*

MODERATOR: ¿Usted también va a participar?
TRANSLATION: *Are you also going to participate?*

MAN: Sí.
TRANSLATION: *Yes.*

MODERATOR: ¿Sí? Le tengo que explicar, ¿sí? Sorry, pero tengo que, de que se trata el focus group, les vamos a hacer como una serie de preguntas sobre su trabajo acá en la granja. Esto también es confidencial. No me tiene que compartir su nombre ni firmar nada.
TRANSLATION: *Yes? I have to explain to you, okay? Sorry, but I have to, what the focus group is about, we are going to ask you like a series of questions about your work here on the farm. This is also confidential. You don't have to share your name with me nor sign anything.*

MAN: Sí.
TRANSLATION: *Yes.*

MODERATOR: La idea es que tampoco comparta los nombres de sus compañeros si es que va a referirse a ellos. No, es voluntario. Si se arrepiente de participar a mitad de camino, me puede decir y todo bien. No se le va a cobrar nada, sino que, por su tiempo, se les van a pagar $25. Si no quiere participar, tampoco va a afectar su trabajo ni nada por el estilo. Y, o sea, antes de que sigamos, ¿tiene alguna pregunta?
TRANSLATION: *The idea is to not share your peers' names either, if you are going to refer to them. No, it's voluntary. If you regret having participated halfway through, you can tell me, and it's all good. You won't be charged anything rather, for your time, you guys will be paid $25. If you don't want to participate, it won't affect your job or anything like that. And, I mean, before we continue, do you have any questions?*

MAN: No.

MODERATOR: No. ¿Quiere participar?
TRANSLATION: *No. Do you want to participate?*

MAN: Sí.
TRANSLATION: *Yes.*

MODERATOR: Sí. Okay. Ya, ahora podemos seguir. Entonces les estaba pidiendo que me contaran un poco que cuál es su rol acá en la granja, qué trabajo hace, cuánto tiempo trabaja.
TRANSLATION: *Yes. Okay. All right, now we can continue. So I was asking you all to tell me a little bit, what is your role here on the farm, what job you do, how long you've worked.*

MAN: Mi oficio, más que nada, es ordeñar.
TRANSLATION: *My job, more than anything, is to milk.*

MODERATOR: Ordeñar.
TRANSLATION: *Milking.*

MAN: Sí, ordeñar más que nada. Me han puesto a puchar aquí, puchar vacas, sé puchar vacas. Y también me han puesto a herdsmanear. No sé mucho de eso de herdsmanear así, partear y todo, no sé mucho porque mi carrera en rancho no ha sido muy larga, apenas voy empezando. Llevo como dos años trabajando en rancho y, pues, apenas voy empezando, y sabiendo la mayoría del tiempo, cuando llega uno, tiene que empezar ordeñando, así que, pues, no sé mucho de eso, pero, sí, de todo . . .
TRANSLATION: *Yes, milking more than anything. They have had me push here, push cows, I know how to push cows. And they've also had me help the herdsman. I don't know a lot about that, helping the herdsman like that, assisting the births and everything, I don't know much because my career on the farm hasn't been very long, I'm just starting. I have been working on the farm like two years and, well, I'm just starting, and knowing the majority of the time, when you arrive, you have to begin milking, so, well, I don't know a lot about that. But, yeah, everything . . .*

MODERATOR: ¿Y cuánto tiempo lleva acá?
TRANSLATION: *And how long have you been here?*

MAN: Aquí como un año ya.
TRANSLATION: *Here about a year now.*

MODERATOR: Un año. ¿Ya antes había trabajado en otra granja?
TRANSLATION: *A year. Had you worked on another farm already?*

MAN: Sí.
TRANSLATION: *Yes.*

MODERATOR: Okay. Bueno, y para seguir entonces, me gustaría saber cómo es un típico turno, el típico turno de cada uno de ustedes acá en la granja, ¿qué es lo que hacen durante el día? ¿Ya? No sé si alguien quiere empezar. Como que me describan un poco desde cuando llegan hasta cuando se van.
TRANSLATION: *Okay. Well, and to continue then, I would like to know what a typical shift is like, the typical shift of each one of you here on the farm, what is it that you do during the day? Got it? I don't know if someone wants to begin. Like describe to me a little from when you arrive until when you leave.*

MAN: Cada día es diferente, diferente rutina que hay.
TRANSLATION: *Every day is different, there's a different routine.*

MODERATOR: Pero, por ejemplo, ¿cuál es la más común para usted?
TRANSLATION: *But, for example, what is the most common for you?*

MAN: Estar sentado.
TRANSLATION: *Sitting around.*

WOMAN: Como nosotras, cuando llegamos en la mañana, tenemos que dar biberones a las becerritas, y luego las cubetas, y repartir todas las cubetas así. Y ya, después, tenemos que darles agua, tirarles maíz allá a las más grandes, y si hay que poner a secar las casas, si están mojadas, poner a secar. Y en la tarde, otra vez, darles agua a las grandes, a las que ya no toman leche, otra vez tirar maíz y tirar leche y biberones.

TRANSLATION: *Like we, when we arrive in the morning, we have to give bottles to the calves, and then the buckets, and distribute all the buckets like that. And then, after, we have to give them water, throw corn over there to the bigger ones, and if the houses have to be put out to dry, if they're wet, put them out to dry. And in the afternoon, again, give water to the big ones, the ones that don't drink milk anymore, throw corn again, and give out the milk and bottles.*

MAN: Lo que yo hago llegando es ver que es lo que me dejan en el turno de la noche, ir a revisar las vacas pues que están pariendo, ve lo que hay, y levantar las vacas secas a que, para irles limpiando . . .
TRANSLATION: *What I do when I arrive is see what they are leaving me from the night shift, go to look over the cows that are giving birth, see what there is, and get the dry cows up to go cleaning them . . .*

MODERATOR: Mm-hmm. ¿Alguien más quiere seguir contando su turno?
TRANSLATION: *Mm-hmm. Does anyone else want to continue telling about their shift?*

MAN: . . . estar un buen tiempo aquí hablando de la rutina, pero en un día normal que no es de, que no sea vaccinación, que no sea de veterinario, que sea un día, vamos a decir, que no hay tanta actividad de inyecciones o buscar vacas para revisar . . . buscan para secar en este caso, hay días que se secan. Pero en un día tranquilo, vamos a decir tranquilo entre comillas, llegamos, se comunica con el siguiente turno, del otro turno se comunican qué es lo que hay pendiente, qué situación hay por atender . . . buscar un animal para revisar o está enfermo, qué sé yo, por una situación ya lo notifican, y ya, de allí, pues, nos vamos para abajo.

Se revisan las vacas que están en el área de preparto, las vacas que están más mal dejada del parto, pues, de las que se secaron . . . se revisa todo eso. Se hacen las camas de esos animales . . . de ese grupo de vacas secas. Si hay alguna vaca en labor, pues, le das un seguimiento. Le atiendes. Pones su agua . . . le pones yodo al ombligo. Se vaccina, si es que es becerra, para la neumonía. Le das calostro lo antes posible. Se pone en la, un cuarto pequeño, en un calentón, que se seque . . . la vaca se ordeña, se hecha el calostro.

Se congela. Se etiqueta qué grado del calostro es. Y la vaca, pues, se le da su . . . vitamina, también su vaccinación para la neumonía . . . o sea, lo marca un numero en la espalda de la fecha en que tuvo su cría. Le pone su banda amarilla para notificar a cada . . . de que es vaca fresca, leche de calostro, y . . . eso es en un día, no más si es que hay algo ahí, también toca que trabaja . . . y de ahí, nos venimos para acá, para, en la mañana, pues, a atender en el hospital, como ya . . . se curan las que . . . mastitis, lo que se tiene que curar.

Se muestra, saca muestra de lo que hay que chequear si ya esté fuera de la, ya pasó un periodo del antibiótico . . . tenemos muestras y sale buena la muestra . . . antibiótico. Y ya . . . y de allí ya, durante el día, pues, lo que viene, pues, estamos viendo no más que hay de vacas pariendo, que hay de vacas para inseminar, si hay algunos de cuidar. Y ya, en lo que sigue, en lo que, entre semana, pues, ya empieza una rutina muy diferente. Los lunes es día de, de aquí se usa para la vaccinación . . . en la mañana . . . si hay una rutina que comenté al principio, ¿no?
TRANSLATION: *. . . to be here quite a while talking about the routine, but on a normal day, that's not vaccination, that's not a veterinarian day, that's a day, let's say, that there's not so much activity with shots or looking for cows to review them . . . they look to dry in this case, there are days that they dry. But on a calm day, let's say, quote, unquote, calm, we arrive, communicate with the next shift, those from the other shift communicate what there is left to do, what situation we have to attend to . . . look for an animal to review or that is sick, what do I know, for a situation, they notify us about it then, and then, from there, well, we head down.*

The cows that are in the pre-birth area are looked over, the cows that are worse off after their birth, well, the ones that dried . . . look over all that. Those animals' beds are made . . . that group of dry cows. If there is a cow in labor, well, you give it treatment. You attend to it. You put water . . .

you put iodine on the bellybutton. If it's a female calf, it's vaccinated against pneumonia. You give them colostrum as soon as possible. You put it in the, a small room, a heated room, to dry . . . the cow is milked, the colostrum is made.

It's frozen. It's labeled what grade of colostrum it is. And the cow, well, it's given its . . . vitamin, also its vaccination against pneumonia . . . I mean, it's marked with a number on its back of the date it had its baby. A yellow band is placed on it to notify every . . . that it's a fresh cow, colostrum milk, and . . . that's in a day, just if there's something else there, you also have to work . . . from there, we come here to, in the morning, well, to attend in the hospital, since . . . are treated . . . mastitis, whatever needs to be treated.

It's shown, a sample is taken of what needs to be checked, if it's now outside the, the antibiotic period already passed . . . we have samples and if the sample is okay . . . antibiotic. And then . . . and from there, during the day, well, what comes, well, we are just looking for cows that are giving birth, cows that need to be inseminated, if there are any, and then, what follows, during the week, well, already a very different routine starts. Mondays are, here, are used for vaccination . . . in the morning . . . if there is a routine that I commented on at the beginning, you know . . .

[Simultaneous discussion]

MAN: . . . trabajo sería dos personas.
TRANSLATION: *. . . work would be two people.*

MAN: . . . de dos porque ya, de allí, hay más trabajo el lunes. Se inyectan en la mañana a las vacas, en el ordeño de la mañana . . . se cura de lo que tenga que curar. Se atiende los partos que se tiene que atender.
TRANSLATION: *. . . two because then, from there, there is more work on Monday. The cows are given shots in the morning, morning milking . . . treat what needs treating, attend the births that need attending.*

MAN: . . . si no acaban . . .
TRANSLATION: *. . . if they don't finish . . .*

MAN: . . . en lo que acabe, de lo que se hace cada vez en la semana, casi es una rutina similar. Los miércoles como este día, hoy fue que vino el veterinario. Se pusieron a chequear todas las vacas el veterinario . . . las revisó, pues, ahí las revisamos en grupo. El día de hoy también se inyecta las vacas del, que se van a inseminar . . . se le da su inyección para que el turno de la noche empiece a . . . que se comunica eso . . .
TRANSLATION: *. . . in what's left, what's done each week, it's pretty much a similar routine. Wednesdays, like today, today was the veterinarian came. The veterinarian checks all the cows . . . he checked them over, well, there we looked them over as a group. Today, the cows are also given shots, the ones that are going to be inseminated . . . they are given their shot so that the night shift can begin to . . . to communicate that . . .*

MODERATOR: ¿Alguien más quiere compartir que tenga turno distinto? Mm-hmm.
TRANSLATION: *Does anybody else want to share that has a different shift? Mm-hmm.*

MAN: Un día como mañana, para mi, cada día es diferente, pero como un ejemplo, ya de mañana llego y chequeo las actividades en la computadora. Me enfoco en el programa de inseminación en la mañana y en el programa de la granja que se van a secar . . . de ordeña para llevarse al área de secas. Y después de eso, me enfoco en si alguien ocupa ayuda, estoy disponible. Si nadie me solicita, sigo con el programa que tengo. Si algo está roto en el rancho y está en mi alcance poderlo

reparar, yo lo reparo. Y sobretodo, si el encargado, el dueño esta ahí, le pregunto si tiene algún programa. Y si no, continuo con mi rutina diaria.
TRANSLATION: *A day like tomorrow, for me, every day is different, but as an example, in the morning, I arrive and check the activities on the computer. I focus on the insemination schedule in the morning and on the farm schedule that they are going to dry . . . milking to bring to the dry area. And after that, I focus on if anyone needs help, I'm available. If no one needs me, I continue with the schedule that I have. If something is broken on the farm and I am able to repair it, I repair it. And above all else, if the one in charge, the owner is there, I ask him if he has any schedule. And if not, I continue with my daily routine.*

MODERATOR: ¿Alguien más que quiere contar cómo es su rutina diaria?
TRANSLATION: *Does anyone else want to tell how their daily routine is?*

MAN: Pues, la verdad, lo que tienen los ranchos es casi lo mismo todo el tiempo. Llega uno, en mi caso, llega uno, ordeñas, te vas . . .
TRANSLATION: *Well, honestly, what the farms have is almost the same all the time. You arrive, in my case, you arrive, you milk, you leave . . .*

MODERATOR: ¿Todo el día ordeña?
TRANSLATION: *Do you milk all day?*

MAN: La mayoría del tiempo, sí. De hecho, yo me distraigo más cuando me ponen a puchar porque es un trabajo más, que siento que es mas distraído, ¿me entiende? Porque eso de ordeñar como que te enfada. Te, la verdad, te enfada ya con el tiempo. Todo el tiempo, todo el tiempo, ordeñando. Ya cuando te sacan a puchar, pues, así está . . . te distrae, ¿verdad? Sí.
TRANSLATION: *The majority of the time, yes. In fact, I enjoy it more when they ask me to push cows because it's a job that's more, I feel that it's more distracting, do you know what I mean? Because milking, it like annoys you. Honestly, it annoys you after a while. All the time, all the time, milking. Then, when they take you off to push, well, that's . . . it distracts you, right? Yeah.*

MAN: Como nosotros, lo del turno de la noche, los ayudantes de herdsman . . . una rutina diferente. Como a veces puchamos, a veces hacemos ayudante de herdsman, entonces cuando hacemos de ayudante, la prioridad primera es la actividad, chequéame en la computadora. Si ellos dejan algo pendiente, medicina, que ya no le da su tiempo, pues, o nos dicen necesitamos poner esta medicina. Eso es una prioridad mas importante, las vacas enfermas.

Y de ahí, las actividades, si ellos no entregan nada, vamos y recorremos el área de las vacas secas que van a parir, y verificamos si todo está bien. Y si no hay nada, pues, ayudamos a puchar . . . lo que él dijo, lo mismo, las vacas secas para ponerle nuevas, limpiarles . . . y de ahí, ya viene la rutina igual. Lo que haiga que hacer, como . . . necesita ayuda . . . o a veces el patrón viene y . . . que hay que hacer otros trabajos. Pero, sí, regularmente, es casi la rutina . . .
TRANSLATION: *Like us, the night shift, the herdsman's assistants . . . a different routine. Like sometimes we push cows, sometimes we are herdsman's assistants, so when we are being assistants, the first priority is the activity, check the computer. If they leave something pending, medicine, that they didn't have time to do, well, or they tell us we need to put that medicine. That's one of the most important priorities, the sick cows.*

And from there, the activities, if they don't turn in anything, we go and look over the area where the dry cows that are going to give birth, and we verify if everything is okay. And if there is nothing, well, we help to push . . . what he said, the same thing, the dry cows, to put new, to clean . . . and from there, then comes the same routine. Whatever there is to do, like . . . needs help . . . or

sometimes the boss comes and . . . that other jobs have to be done. But, yeah, regularly, that's pretty much the routine . . .

MODERATOR: ¿Alguien quiere agregar algo de su rutina? ¿No? ¿Y qué hacen, por ejemplo, si tienen una duda en su trabajo? ¿O sea, a quién recurren normalmente?
TRANSLATION: *Does anyone want to add some from their routine? No? And what do you do, for example, if you have a concern at your work? I mean, who do you go to normally?*

MAN: Como yo como ayudante de herdsman, primero veo qué es más sencillo y le hablo . . . encargado en el día. Ellos saben más que nosotros . . . el ayudante de herdsman en la noche. Les llamamos a ellos, y por lo regular siempre nos contestan. Pero si no nos contestan y si nuestra prioridad es ya que tenemos que hacer, tratar una vaca, ya hablamos al patrón.

Y hay cosas, como tenemos problemas con una vaca la que va a parir, el patrón nos ha dicho que, si nosotros no estamos a nuestro alcance, que le hable no más al veterinario. Y si, también nos ha dicho que, si haya, hubiera un accidente con el personal, que le hable no más, depende, tengo número de una enfermera . . . más grave, le podemos llamar al 911 . . .
TRANSLATION: *Like myself, as a herdsman's assistant, first I see what is most simple, and I talk to him . . . in charge during the day. They know more than us . . . the herdsman's assistant at night. We call them, and they usually always answer us. But if they don't answer us, and if our priority is that we have to do, to treat a cow, then we talk to the boss.*

And there are things, like we have problems with a cow, one that's going to give birth, the boss has told us, if we are not able to, that we just talk to the veterinarian. And if, he has also told us, if there is an accident with the staff, to just talk to, it depends, I have a nurse's number . . . more serious, we can call 911 . . .

MAN: Si yo tengo una duda, depende acerca de que sea la duda, ¿verdad? Si tengo una duda acerca de una vaca, si . . . para tratarse o no tratarse, si esté enferma, vengo y checo el sistema, y si sigo con mi duda, hablo con el dueño del rancho. Él toma decisiones cuando yo no puedo tomarlas. Él las toma, qué hacer. Él es mi jefe inmediato.
TRANSLATION: *If I have a concern, it depends on what the concern is, right? If I have a concern about a cow, if . . . to treat it or not treat it, if it's sick, I come and check the system, and if I still have the concern, I talk to the farm owner. He makes the decisions when I can't make them. He makes them, what to do, he is my immediate boss.*

MODERATOR: Mm-hmm. Perfecto. Okay. ¿Si tiene alguna duda?
TRANSLATION: *Mm-hmm. Perfect. Okay. If you have a concern?*

MAN: Pues, la verdad, cuando uno tiene una duda que no sabe, que no está a tu alcance, pues, aquí . . . pues, lo principal es preguntarse entre uno. Preguntarse entre uno, comunicarse, no más hacer las cosas, y buscar quien lo pueda hacer. Como digamos, el herdsman, él es el encargado, él, ya si él no puede, él puede buscar quien solucionar el problema. Sí.
TRANSLATION: *Well, honestly, when you have a concern that you don't know, that's not in your wheelhouse, well, here . . . well, the main thing is ask each other. Ask each other, communicate, just do things, and look for someone who can do them. Since, let's say, the herdsman, he is the one in charge, he, if he can't, he can look for someone to solve the problem. Yeah.*

MODERATOR: ¿Ustedes? ¿A quién le preguntan si tienen alguna duda?
TRANSLATION: *You guys? Who do you ask if you have a concern?*

WOMAN: Lo mismo que . . .
TRANSLATION: *The same as . . .*

WOMAN: En nuestro caso, las becerras, pues, preguntamos a ellos, a la, el señor, y él . . . o si necesitamos ayuda en algo, ellos nos ayudan.
TRANSLATION: *In our case, the calves, well, we ask them, the guy, and he . . . or if we need help on something, they help us.*

MODERATOR: ¿Ustedes, si tienen alguna duda?
TRANSLATION: *You guys, if you have a concern?*

MAN: Tomar la decisión . . .
TRANSLATION: *Make the decision . . .*

MAN: Si está en mi alcance tomar una decisión, la tomo basado en . . .
TRANSLATION: *If I am able to make a decision, I make it based on . . .*

[Simultaneous discussion]

MAN: . . . si no, pues, ya . . . con el mánager del rancho.
TRANSLATION: *. . . and if not, well, then . . . with the farm manager.*

MODERATOR: Perfecto. Y cuando hablas con el mánager, con el administrador, se quedan, ¿cómo es su relación con el mánager?
TRANSLATION: *Perfecto. And when you speak with the manager, with the administrator, are you, how is your relationship with the manager?*

MAN: Como yo, siempre casi estamos dialogando, yo siempre estoy dialogando con él. Siempre y cuando él tenga también tiempo . . . si tengo duda . . . esperamos, y si está con alguien, esperamos, espero que se desocupe, y ya le pregunto. Pero realmente, mi comunicación con el mánager es estable . . .
TRANSLATION: *Like I, we are always maintaining a dialogue, I am always maintaining a dialogue with him. Whenever he also has time . . . if I have a concern . . . we wait, and if he's with someone, we wait, I wait for him to have a free moment, and then I ask him. But really, my communication with the manager is stable . . .*

MODERATOR: Mm-hmm.

MAN: ¿Mi comunicación? Hablo con él casi siempre, estoy aquí, y él está en . . . mi comunicación con él es muy bien. Muy buena comunicación.
TRANSLATION: *My communication? I almost always talk with him, I'm here, and he is in . . . my communication with him is very good. Very good communication.*

MODERATOR: ¿También es frecuente?
TRANSLATION: *Is it also frequent?*

MAN: Casi para cosas de trabajo, no mucho, porque ya tenemos un programa que seguir, pero siempre nos estamos hablando, comunicando, y pasando el chisme, cualquier cosa, cualquier información y todo eso, noticias . . . es como una persona más en el grupo de trabajo. Una buena comunicación.
TRANSLATION: *In terms of work-related things, not really, because we already have a schedule to follow, but we are always talking, communicating, and shooting the breeze, anything, any information, and all that, news . . . he's like just another person in the work group. A good communication.*

MODERATOR: Muy bien. ¿Todos tienen la misma relación o la misma comunicación con el mánager?
TRANSLATION: *Very good. Do you all have the same relationship or the same communication with the manager?*

MAN: Pues, en si, nosotros, bueno, yo no tengo la comunicación así, que digamos, para hablar así mucho con él porque yo no tengo mucho tiempo en este rancho y casi no hemos hablado, diría, como dice aquí mi tío, de ponernos a chismear, ¿verdad? Pero para platicar, pues, más que nada, dialogar . . . pero, sí, se me hace que es una persona flexible, se adapta a lo que uno a veces necesita o puede o quiere. Como, digamos, si tu quieres un día de descanso, que no puedas venir, no más se lo pide con tiempo, y él te acomoda, él le acomoda el schedule y todo, y es una persona flexible.
TRANSLATION: *Well, really, we, well, I don't have the communication like, say, to talk like that a lot with him because I haven't been at this farm long, and we really haven't talked, I would say, like my uncle is saying here, to start gossiping, right? But to chat, well, more than anything, have a dialogue about . . . but, yeah, it seems to me like he is a flexible person, he adapts to what you sometimes need or can or want. Like let's say, if you want a day off, that you can't come in, just ask him for it ahead of time, and he accommodates you, he accommodates your schedule and everything, and he is a flexible person.*

MODERATOR: Okay.

MAN: Sí.
TRANSLATION: *Yeah.*

MODERATOR: ¿Y puedes describir en qué situaciones, por ejemplo, su manager viene hablar con usted?
TRANSLATION: *And can you describe in what situations, for example, your manager comes to talk with you?*

MAN: ¿Perdón?
TRANSLATION: *Pardon?*

MODERATOR: Como, ¿cuándo el mánager va a hablar con usted?
TRANSLATION: *Like when does the manager go to talk with you?*

MAN: En mi caso, cuando me equivoco un número, si apunto un número mal, pues, por seguro me va a preguntar cual es cual.
TRANSLATION: *In my case, when I get a number wrong, I write a number wrong, well, of course, he's going to ask me which is which.*

MAN: Qué es lo que pasó.
TRANSLATION: *What happened.*

MODERATOR: Pero, ¿o no es muy frecuente que él venga a hablar con ustedes así?
TRANSLATION: *But, or is it not very frequently that he comes to talk with you guys like that?*

MAN: No. Casi . . . se equivoque uno, va y pregunta, oye, qué pasó aquí, se equivocaron, qué es lo que hay . . . más te vaya a reclamar . . .

TRANSLATION: *No. Almost . . . you make a mistake, he goes and asks, hey, what happened here, you guys made a mistake, what is there . . . goes to you to complain . . .*

WOMAN: Como nosotros, en la noche . . .
TRANSLATION: *Like we, at night . . .*

MODERATOR: Okay. Y, ¿cómo funciona los, el tema de los descansos en la granja, así como en el día, si tienen algún, por ejemplo, un horario específico donde se pueden tomar un break para almorzar, o más que nada es como dependiendo de cuando tengan tiempo?
TRANSLATION: *Okay. And how do the . . . work . . . in terms of breaks on the farm, like during the day, if you have some, for example, a specific schedule where you can take a break to have lunch, or is it just depending on when you have time?*

MAN: Nosotros no tenemos tiempo exacto. Puede ser 7:00 a.m. Algunas veces, varias veces, puede ser 10:00, puede ser de la mañana, mediodía.
TRANSLATION: *We don't have an exact time. It can be at 7:00 a.m. Sometimes, various times, it can be 10:00, it can be in the morning, midday.*

MODERATOR: Mm-hmm.

MAN: . . . el promedio, vamos a decir que es en la mañana, antes de las 8:00. Pero no es nada seguro.
TRANSLATION: *. . . on average, we'll say that it's in the morning, before 8:00. But it's nothing certain.*

MODERATOR: ¿Y durante el día es variable?
TRANSLATION: *And it varies during the day?*

MAN: Ya durante el día, pues, si tenemos ganas de tomar agua y todo eso, pero, pues, hay necesidades que no podemos evitar.
TRANSLATION: *Then, during the day, well, if we need a drink of water and all that, but, well, there are necessities that we can't avoid.*

MODERATOR: Claro.
TRANSLATION: *Of course.*

MAN: Descansar, pues, si se pudiera, mediodía, después del mediodía.
TRANSLATION: *To rest, well, if able, midday, after midday.*

MAN: Nosotros lo de la noche, el turno de la noche casi por lo regular estamos acostumbrados a comer juntos, cenamos juntos. Nosotros estamos, así estamos acostumbrados. Lo que nos manda es el, la ordeña. Y como los ayudantes de herdsman . . . prioridad más importante es . . . pero lo demás, las otras personas siempre, cuando termina la ordeña, venimos a cenar. Pero el ayudante de herdsman, si tiene prioridades allá, ya no viene.

Pero en regular siempre, casi todas las noches, estamos casi siempre en la misma hora, y antes, el otro mánager decía que cuando la ordeña iba atrasada, como los ordenadores no pueden parar, ellos no paran . . . hasta que llega el turno de lavado. Entonces él siempre decía, si uno de los muchachos, necesitan ir a comer, el uno de atrás . . . que tendría que venir ayudar a la persona que quería venir a cenar. Pero siempre, lo regular, nosotros cenamos. Es así, estamos . . . juntos. Cenamos juntos.

TRANSLATION: *Us at night, the nightshift, almost regularly, we are accustomed to eating together, we have dinner together. We are, that's how we're accustomed. It depends on the milking schedule. And as herdsman's assistants . . . the most important priority is . . . but the rest, the other people always, when the milking ends, we come to eat. But the herdsman's assistant, if he has priorities over there, then he doesn't come.*

But regularly we always, almost every night, we're almost always at the same time, and before, the other manager was saying that when the milking was behind, like the milkers can't stop, they don't stop . . . until the washing shift arrives. So he always said, if one of the guys, they need to go to eat, the one behind . . . that he would have to come to help the person that wanted to come eat dinner. But always, regularly, we eat dinner. It's like that, we are . . . together. We eat dinner together.

MODERATOR: Entre ustedes se organizan entonces para tomar un break, otro turno lo cubre, ¿eso?
TRANSLATION: *So you organize amongst yourselves to take a break, another shift covers it, that?*

MAN: Nosotros no más tomamos break cuando ellos . . . para lo que es las 11:00 p.m., por el promedio. Y comenzamos a las 2:00.
TRANSLATION: *We just take a break when they . . . by 11:00 p.m., on average. And we start at 2:00.*

MODERATOR: ¿Sí? ¿Ustedes también tienen como el mismo esquema?
TRANSLATION: *Yeah? Are you guys also with the same outline?*

MAN: Es lo mismo. Bueno, de hecho, yo estoy en la noche con . . .
TRANSLATION: *It's the same. Well, in fact, I'm at night with . . .*

MAN: Casi la mayor parte de los . . . comemos dentro de ordeña y ordeña, verdad. Entonces nos tomamos un tiempo para comer ahí, y a veces no es posible, pero la mayor parte del tiempo, sí podemos hacer. Algunos, no, pero . . .
TRANSLATION: *Almost the majority of the . . . we eat in between milking, really. So we take some time to eat there, and sometimes it's not possible, but the majority of the time, we can do it. Some, no, but . . .*

MODERATOR: ¿Tratan de comer juntos?
TRANSLATION: *Do you try to eat together?*

WOMAN: Sí.
TRANSLATION: *Yes.*

MAN: Usted sabe que siempre somos comunicativos, los hispanos.
TRANSLATION: *You know that we're always communicative, us Hispanics.*

WOMAN: Sí.
TRANSLATION: *Yeah.*

WOMAN: Sí.
TRANSLATION: *Yeah.*

MODERATOR: Sí. Ustedes también, lo mismo, ¿sí?
TRANSLATION: *Yeah. You guys as well, the same thing, right?*

WOMAN: Sí.
TRANSLATION: *Yeah.*

WOMAN: Sí. Bueno, yo estoy en la noche con ellos.
TRANSLATION: *Yeah. Well, I'm at night with them.*

MODERATOR: Perfecto. ¿Y donde comen, por ejemplo? Si tienen, si van a comer, ¿dónde comen?
TRANSLATION: *Perfect. And where do you eat, for example? If you guys have, if you guys are going to eat, where do you guys eat?*

MAN: Aquí en el comedor.
TRANSLATION: *Here in the dining room.*

MODERATOR: ¿Acá en el comedor?
TRANSLATION: *Here in the dining room?*

MAN: Sí.
TRANSLATION: *Yes.*

MAN: Tenemos . . . comer aquí.
TRANSLATION: *We have . . . to eat here.*

WOMAN: Sí.
TRANSLATION: *Yes.*

MODERATOR: Okay. Sí. Que a veces, como uno . . . de repente. Okay. Y, por ejemplo, si quieren almorzar algunos de ustedes, no, por ejemplo, ¿no se va afuera de la granja o siempre están acá adentro? ¿Algunos se toman algún break de repente para salir?
TRANSLATION: *Okay. Yeah. Sometimes, like you . . . suddenly. Okay. And, for example, if some of you want to have lunch, you know, for example, do you go outside the farm or are you always here inside? Do some of you take a break to go out?*

MAN: Raramente, que haya alguno que no ha traído lunch o algo, puede ir aquí a la gasolinera a traer. Es rara, las veces que . . .
TRANSLATION: *Rarely, if there's someone who hasn't brought lunch or something, they can go here to the gas station to bring something. It's rare, the times that . . .*

WOMAN: Por lo regular, todos traemos lunch.
TRANSLATION: *Regularly, we all bring lunch.*

MODERATOR: . . . ¿sí? Okay. Bueno, ahora vamos a pasar por las preguntas en relación a qué equipo, como de protección personal, tienen que usar acá en su trabajo, ¿ya? Y, ¿qué reglas tiene la granja en términos de vestimenta? ¿Qué tienen que usar normalmente y qué está regulado aquí? No sé, por ejemplo, el overol, la protección de ojo, los guantes. ¿Qué es lo que, qué vestimenta es la que siempre tienen que usar acá para trabajar?
TRANSLATION: *. . . yeah? Okay. Well, now we are going to go to the questions in relation to what equipment, like for personal protection, do you have to use here in your work? All right? And what rules does the farm have in terms of dress? What do you normally have to use and what is regulated*

here? I don't know, for example, overalls, eye protection, gloves. What is it that, what dress do you always have to use here to work?

MAN: Pues, de hecho, el patrón le tiene todo el equipo. Que una gente no lo quiere usar . . . es diferente . . . pero nadie los usa. Nadie los quiere utilizar. Sí, y para los que andan puchando, ellos tienen su mascarilla para . . . pero poco . . .
TRANSLATION: *Well, in fact, the boss has all the equipment for you. That some people don't want to use it . . . it's different . . . but no one uses them. Nobody wants to use them. Yeah, and for those that are leading the cows to milk, they have to use their mask to . . . but a little . . .*

MAN: Se usan un poco . . .
TRANSLATION: *They are used a little.*

MAN: Poco lo usan.
TRANSLATION: *They use them very little.*

MAN: . . . como ahorita lo están usando, y está raro eso porque traen un par de los lentes . . .
TRANSLATION: *. . . like right now they are using it, and that's rare because they bring a pair of glasses . . .*

MAN: No, lo que pasa, perdón, lo que pasa es que ayer comenzó a hablar también . . .
TRANSLATION: *No, what happens, sorry, what's happening is that he also started to talk yesterday . . .*

MAN: Hablar lo del uso de toda las, todo lo que tenemos para la seguridad personal, pues, es, estaba . . . el uso común de eso, pero parece que sí funcionó . . .
TRANSLATION: *Talking about the use of all the, all that we have for personal safety, well, it's, it was . . . the common use of that, but it seems that it did work . . .*

MODERATOR: Pero qué cosas tienen que usar, que les dicen, ponle . . .
TRANSLATION: *But what things do you need to use, that they tell you, put . . .*

MAN: Guantes.
TRANSLATION: *Gloves.*

MAN: Guantes.
TRANSLATION: *Gloves.*

MAN: Es, no es obligatorio, como, bueno, en la ordeña, sí, obliga. Nosotros, en ocasiones, pues, usamos guantes. O tal vez, yo de repente ando sin guantes, pero, o sea, aquí . . . seguridad, de lentes de seguridad, todo eso, no lo uso.
TRANSLATION: *It's not obligatory like, well, in milking, yes, you're obligated. We, on occasion, well, we use gloves. Or perhaps, I sometimes go without gloves, but, I mean, here . . . safety, safety glasses, all that, I don’t use it.*

MODERATOR: ¿Las botas?
TRANSLATION: *Boots?*

MAN: Botas, nos provee el patrón. La ropa, pues, uno la tiene que traer. A los ordeñadores, en ocasiones, nos ha comprado overoles. Decían otras personas, había un patrón que ya murió, decían

que él compraba . . . pero, pues, en general, las cosas de seguridad para el rancho, sí los provee el mánager.
TRANSLATION: *Boots, the boss provides them for us. The clothes, well, you have to bring them. For the milkers, on occasion, he has bought us overalls. Other people said, there was a boss that already passed away, they said that he bought . . . but, well, in general, safety things for the farm, the manager does provide them.*

MODERATOR: Pero, ¿no están obligados a usarlas si no quieren?
TRANSLATION: *But you guys aren't obligated to use them if you don't want to?*

MAN: En los químicos, sí. Sí, estamos obligados. Ya si los usamos . . . la mascarilla, lentes, guantes. Como sulfato . . . pero se debe. Sí, lo usamos, bueno, nosotros lo usamos, cuando nosotros trabajamos con sulfatos, sí lo usamos.
TRANSLATION: *With the chemicals, yes. Yes, we are obligated. If we use the . . . the mask, glasses, gloves. Like sulphate . . . but you should. Yes, we use it, well, we use it, when we work with sulphates, we do use it.*

MAN: Todas las áreas de que, de uso de químicos . . .
TRANSLATION: *All the areas that, where chemicals are used . . .*

MAN: Tratamos . . .
TRANSLATION: *We try to . . .*

MAN: . . . tienen señalamientos en respeto al uso de todas las substancias.
TRANSLATION: *. . . they have signage with respect to the use of all the substances.*

MAN: Esa del área que está más enfocado en la protección.
TRANSLATION: *That's the area that's most focused on protection.*

MODERATOR: Claro.
TRANSLATION: *Of course.*

MAN: Sí.
TRANSLATION: *Yeah.*

MODERATOR: Que bien. ¿Ustedes también piensan que tienen el mismo reglamento?
TRANSLATION: *Good. Do you guys also think you have the same rules?*

MAN: . . . la verdad, pues, los ordeñadores, yo lo voy a decir porque, pues, básicamente, pues, yo ordeño, ¿verdad? Y atrás casi no nos han dado mucho, pero, sí, como dicen ellos, cuando uno, digamos, atrás puchando, tienes que, bueno, deberías usar la máscara y lentes. Pero la mayoría de las veces, no la usan, o, y, pues, para ordeñar, pues, sí, tienen las mangas y guantes.
TRANSLATION: *. . . honestly, well, the milkers, I'm going to say it because, well, basically, well, I milk, right? And back there, they really haven't given us much, but, yeah, like they say, when you, let's say, are behind pushing, you have to, well, you should use the mask and glasses. But the majority of the time, they don't use it or, and, well, to milk, well, yeah, they have the sleeves and gloves.*

WOMAN: Los lentes.

TRANSLATION: *Glasses.*

MAN: Y lentes, también.
TRANSLATION: *And glasses as well.*

MODERATOR: ¿Lentes para ordeñar, también?
TRANSLATION: *Glasses to milk as well?*

MAN: También, sí.
TRANSLATION: *As well, yes.*

MODERATOR: ¿Y poner las botas también?
TRANSLATION: *And to put on boots as well?*

MAN: Sí.
TRANSLATION: *Yes.*

WOMAN: También las botas.
TRANSLATION: *Also the boots.*

[Simultaneous discussion]

MODERATOR: Y el overol, también, lo tiene que usar, ¿no?
TRANSLATION: *And overalls, also, you have to use them, right?*

MAN: No. Como requerimiento, solamente tiene . . .
TRANSLATION: *No. As a requirement, you just have . . .*

MAN: Su mandil.
TRANSLATION: *Your apron.*

MAN: . . . usar guantes en la parla, y mascarilla y lentes en el área de química. Ese es el requerimiento básico que hay. Los ordeñadores tienen que usar guantes antes que toquen una chichi(?) o que toquen la leche. Y las personas que trabajan con químicos, se requiere que se pongan lentes y mascarilla. Es lo único requerimiento. So él tiene ahí más cosas de seguridad que se requiere, pero no las usamos. So, además, él compra lo que se le pide.
TRANSLATION: *. . . to use gloves in the parlor, and mask and gloves in the chemical area. That's the basic requirement there is. The milkers have to use gloves before they touch a teat or touch the milk. And the people that work with the chemicals, it's required that they put on glasses and mask. It's the only requirement. So he has more safety things there that are required, but we don't use them. So, also, he buys what you ask him for.*

MODERATOR: Okay. Y, por ejemplo, para los que trabajan con animales enfermos, tampoco . . .
TRANSLATION: *Okay. And, for example, for those that work with the sick animals . . .*

WOMAN: Sí, nosotros tenemos que usar guantes todo el tiempo porque andamos tocando las becerritas que traen diarrea. Si las tocamos así con la mano, podemos . . . y tenemos que usar también mascarilla a veces porque ponemos . . . o a veces . . . o los lentes también . . .
TRANSLATION: *Yeah, we have to use gloves all the time because we are touching the little calves that have diarrhea. If we touch them like that with our hands, we can . . . and we have to use, also, a mask sometimes because we put . . . or glasses as well . . .*

MODERATOR: Y, ¿qué piensan sobre los requerimientos de vestimenta? ¿Está bien, está mal? ¿Le gustaría que sea distinto? A su aparecer.
TRANSLATION: *And what do you think about the dress requirements? It's good, it's bad? Would you like it to be different? In your opinion.*

MAN: Sí, debería de comprarle a uno.
TRANSLATION: *Yeah, he should buy it for you.*

MAN: Hemos hablado acerca de eso, del uso de un uniforme, pero, pues, hemos acabado . . . cuando sugerimos eso, pues, ya se nos dice que cuanto cuesta todo esto, es por todo este servicio, un uniforme. Y, pues . . . tanto ha cobrado, ¿no?
TRANSLATION: *We have talked about that, about the use of a uniform, but, well, we have ended . . . when we suggested that, well, it's told to us how much all this cost, it's for this whole service, a uniform. And, well . . . charged so much, you know.*

WOMAN: Sí, nos cobra.
TRANSLATION: *Yeah, he charges us.*

MAN: Nos cobra una cierta cantidad por todo el mantenimiento de . . . y todo eso. Y, pues, ya como únicamente vemos que no nos conviene y acabamos poniendo nosotros nuestra misma ropa.
TRANSLATION: *He charges us a certain amount for all the maintenance of . . . and everything. And, well, so we just see that we don't need it, and we end up putting on our own clothes.*

[Simultaneous discussion]

MAN: Pero, sí, se ha manejado la posibilidad, años atrás . . .
TRANSLATION: *But, yeah, the possibility of it was considered, years back . . .*

MAN: Es bien difícil, como dice el señor, siempre acabamos en no control, ¿verdad? Porque las compañías que suplen la ropa a veces no tienen la ropa adecuada que nosotros ocupamos. Ellos suplen cierto tipo de ropa, entonces no todos ocupamos lo mismo porque hacemos diferentes trabajos. Entonces, por eso, no llegamos a ningún acuerdo. Porque pagar por algo que no me sirve . . .
TRANSLATION: *It's really difficult, like he says, we always end up without control, right? Because the companies that supply the clothing sometimes don't the adequate clothing that we use. They supply a certain type of clothing, so we don't all use the same thing because we do different jobs. So for that reason, we didn't arrive at an agreement, because paying for something that doesn't serve me . . .*

MAN: Las compañías que manejan todo eso de los uniformes, principalmente son uniformes industriales o de restaurantes, pero para lo que debe ser . . . la industria de lácteos . . . no, no nos proveemos . . . vamos, por ejemplo, la gente que trabaja fuera ocupa equipo chamarras de muy buena resistente para el frio. Botas igual. Nosotros, pues, un poco menos que ellos, pero, pues, también necesitamos una ropa más oficial. Ellos no proveen, proveen unas chamarras, pero no son chamarras muy . . .
TRANSLATION: *The companies that manage all that with the uniforms, they are mainly industrial or restaurant uniforms, but for what should be . . . the dairy industry . . . no, they don't provide us . . . we go, for example, the people that work outside need equipment like jackets with good resistance to the cold. Boots as well. Us, well, a little less than them, but, well, we also need more official clothing. They don't provide, they provide some jackets, but they aren't very . . . jackets . . .*

MAN: Y también yo pienso que, porque hacemos muy poquito, a lo mejor por él no provee más variedad o más, por hacemos poquito, pues. Y las empresas grandes, ya son más personal, entonces recolectan más.
TRANSLATION: *And also I think that, because we are very few, maybe he doesn't provide more variety or more, because we are few, you know. And the big businesses, they are more staff, so they need more.*

MODERATOR: ¿Y qué, cuáles son las practicas que tienen acá como . . . sobre lavado de manos en el trabajo? ¿Cuándo están como obligados a lavarse la mano, o si no?
TRANSLATION: *And what are the practices that you have here like . . . about handwashing at work? When are you guys like obligated to wash your hands, or if not?*

MAN: Cuando, la otra vez, teníamos un curso de una persona que vino, creo que, también de una universidad . . . ella nos dijo que era importante cuando venimos al baño, antes de, bueno, antes de hacer las necesidades . . . para comer, la gente que trabaja en el parlo es, la prioridad más importante es cambiarse los guantes si va a tomar agua o algo, quitar los guantes arriba y ponerse otros nuevos. Eso es obligatorio.
TRANSLATION: *When, the other time, we had a course from a person that came, I believe, also from a university . . . she told us that it was important, when we come to the bathroom, before, well, before doing what you need to do . . . to eat, the people that work in the parlor are, the most important priority is to change your gloves if you're going to drink water or something, take of your gloves above and put on new ones. That is obligatory.*

MAN: Obligatoriamente, no tenemos ninguna regla establecida. A veces andamos trabajando y comiendo . . . o sea, obligatorio, no tenemos ninguna regla que diga lávate la mano antes de hacer tal cosa, no lo tenemos.
TRANSLATION: *Obligatorily, we don’t have any established rule. Sometimes we are working and eating . . . I mean, obligatory, we don't have any rule that says, wash your hands before doing such and such thing, we don't have it.*

MAN: Yo trato de tener las manos más limpias posible cuando tengo contacto con alimento.
TRANSLATION: *I try to have my hands as clean as possible when I'm in contact with food.*

MODERATOR: Mm-hmm, claro.
TRANSLATION: *Mm-hmm, of course.*

MAN: Cuando trabajo con animales, siempre con mis guantes. Ya no trabajo con animales enfermos, pues, ocasionalmente o muchas veces . . . sin guantes. Pero cuando se trata de atender algún animal o algo, pues, eso, yo uso guantes.
TRANSLATION: *When I work with animals, always with my gloves. I don’t work with sick animals anymore, well, occasionally or many times . . . without gloves. But when it's a matter of attending some animal or something, well, that, I use gloves.*

[Simultaneous discussion]

MAN: Entonces a mi me pasó la experiencia, pero con un animal que tenía mastitis de agua, y me cayó sangre. Cayó sangre aquí . . . se secó, sentí una comezón . . . rojo, rojo . . . sangre. Se me hizo un, hicieron este grano . . . lo tenía por todos lados. De repente, fueron aquí, acá, aquí . . . cual era el problema. Yo pensé que . . . y, pues, eso era, es una seriedad.

Es muy serio todo eso . . . una enfermedad que nunca se imagina en el lugar que menos piensa uno. Y no sabe uno si va a salir vivo de eso. Lo que pasa es que . . . con esa enfermedad . . . la razón por la cual, deberíamos usar guantes todo el tiempo, y aún así, parece que no entendimos la experiencia, ¿verdad? Espero que, pues . . .
TRANSLATION: *So I had the experience, but with an animal that had water mastitis, and blood got on me. Blood fell here . . . it dried, I felt an itching . . . red, red . . . blood. I got these bumps . . . I had it everywhere. Suddenly, they went here, here, here . . . what the problem was. I thought that . . . and, well, that was, it's a serious thing.*
All of that is really serious . . . an illness that you'd never imagine where you least expect it. And you don't know if you're going to survive that. What happens is that . . . with that illness . . . the reason for which, we should use gloves all the time, and even so, it seems that we didn't learn from the experience, right? I hope that, well . . .

[Simultaneous discussion]

MODERATOR: Está bien, sí, la idea es que me compartan . . .
TRANSLATION: *It's okay, yeah, the idea is that you share with me . . .*

MAN: En el caso de Luis, Luis fue para en el hospital, que ya se murió de una bacteria que le quemó en el estómago . . .
TRANSLATION: *In Luis' case, Luis went to the hospital, he already died of a bacterium that burnt him in his stomach.*

MAN: Fue dos meses.
TRANSLATION: *It was two months.*

MAN: Dos meses que tenía un . . . de estomago . . . diarrea . . .
TRANSLATION: *Two months that he had a . . . stomach . . . diarrhea . . .*

WOMAN: Es que cuando uno agarra a veces los animales que están enfermos, aquí a veces los agarras y no traes guantes, y al rato te pasa la mano en la boca y . . .
TRANSLATION: *It's that sometimes, when you grab the animals that are sick, here sometimes you grab them, and you're not wearing gloves, and then you put your hand in your mouth, and . . .*

MAN: Estamos hablando de, no sé, cientos de miles de bacterias, tipos de bacteria. Y andamos diario . . . viviendo con ellas . . .
TRANSLATION: *We are talking about, I don't know, hundreds of thousands of bacteria, types of bacteria. And we are going daily . . . living with them . . .*

MODERATOR: Claro. ¿Tampoco tienen como una regla para cuando maneja el animal enfermo que les diga, no, tienen que lavarse las manos?
TRANSLATION: *Of course. Do you not have like a rule for when you are handling the sick animal, that tells you guys, no, you have to wash your hands, either?*

MAN: Es lo mismo, no hay una regla . . . nos sugiere el uso de guantes, pero no es una cosa obligatoria que esté sobre nosotros diciéndolo, ah, ya . . . sin guantes, tienes una . . .
TRANSLATION: *It's the same thing, there isn't a rule . . . suggests the use of gloves to us, but it's not an obligatory thing that's over us, saying, ah . . . without gloves, you have a . . .*

MAN: Warning.

MAN: Un warning o algún tipo de sanción. No se nos, nunca nos ha puesto así la administración . . . pues, yo pienso que vamos a cambiar nuestra actitud, pero ahorita casi es voluntario.
TRANSLATION: *A warning or some type of sanction. The administration has never put something like that on us . . . well, I think that we are going to change our attitude, but right now, it's pretty much voluntary.*

MODERATOR: Okay. ¿Sí, todos piensan igual?
TRANSLATION: *Okay. Yes, everyone thinks the same?*

WOMAN: Yo creo que, por el bien de uno, pues, tenemos que usar guantes . . . porque, si no, nos vamos a andar enfermando cada rato.
TRANSLATION: *I believe that, for your own good, well, we have to use gloves . . . because, if not, we are going to be getting sick all the time.*

MODERATOR: Y ¿qué usan, por ejemplo, cuando se lavan las manos, qué usan para lavarse las manos, tienen algún jabón especial? ¿Qué les proveen, algo, desinfectante?
TRANSLATION: *And what do you use, for example, when you wash your hands, what do you use to wash your hands, do you guys have some special soap? What do they provide you, something, disinfectant?*

MAN: Desinfectante.
TRANSLATION: *Disinfectant.*

MODERATOR: ¿Sí?
TRANSLATION: *Yeah?*

MAN: Desinfectante.
TRANSLATION: *Disinfectant.*

MODERATOR: ¿Hay desinfectante disponible?
TRANSLATION: *Is there disinfectant available?*

MAN: Sí.
TRANSLATION: *Yes.*

MAN: Sí, hay, aquí hay todo.
TRANSLATION: *Yes, there is, here there's everything.*

MAN: . . . agua caliente, todos tienen agua caliente.
TRANSLATION: *. . . hot water, everyone has hot water.*

MAN: En todos los lugares donde se ve un sink o hay manera de lavarse, que hay agua, hay un desinfectante al alcance.
TRANSLATION: *Everywhere you see a sink or there's a way to wash yourself, where there's water, there is a disinfectant within reach.*

MAN: Y agua fría y agua caliente, están las dos.
TRANSLATION: *And cold water and hot water, both are there.*

MODERATOR: Perfecto. Y, bueno, ¿cómo se prepara para salir del trabajo en el día, o sea, si se cambian de ropa? O, cómo es cuando ya estás, terminan su trabajo, ¿cómo se van para la casa?

TRANSLATION: *Perfect. And, well, how do you prepare yourself to leave work for the day, I mean, if you change clothes? Or how is it when you are, you guys finish your work, how do you go home?*

MAN: . . . llega uno con su ropa aquí de la casa, y aquí tienen otra ropa para cambiarse. Aquí se queda la ropa sucia, aquí se lava, y se queda.
TRANSLATION: *. . . you arrive with your clothes from home, and here you have other clothes to change into. The dirty clothes stay here, they are washed here, and they stay.*

MODERATOR: ¿Tienen donde lavar y todo eso?
TRANSLATION: *Do you guys have a place to wash and all that?*

WOMAN: Sí.
TRANSLATION: *Yes.*

MODERATOR: Okay. ¿Todos hacen lo mismo?
TRANSLATION: *Okay. You all do the same thing?*

WOMAN: Sí.
TRANSLATION: *Yes.*

MODERATOR: O, ¿de repente se van con lo puesto?
TRANSLATION: *Or do you guys ever leave with what you have on?*

MAN: No, del rancho, no.
TRANSLATION: *No, from the farm, no.*

MAN: Bueno, por mi, yo me cambio también, me lavo, y no sé a cada quien . . .
TRANSLATION: *Well, for me, I change also, I wash myself, and I don't know how each person . . .*

MAN: Yo casi no me cambio. Porque yo uso un overol encima de mi ropa, entonces me quito mi overol sucio, me llevo, no me cambio, pues, pantalón. Tengo un lugar especial en la casa donde manejo mi ropa de trabajo. No me cambio todos los días del pantalón, ni de playera, no, y la ropa interior . . .
TRANSLATION: *I almost never change. Because I use overalls over my clothes, so I take off my dirty overalls, I'm wearing, I don't change, well, pants. I have a special place at the house where I handle my work clothes. I don't change my pants nor my shirt every day, you know, and underwear . . .*

MODERATOR: ¿Pero deja el overol acá?
TRANSLATION: *But you leave the overalls here?*

MAN: No sale del trabajo, sí. Por sí, overol y camisa, dejo lavando y me llevo mi pantalón, como este, trabajé ayer con esto . . . no hay problema. Siempre me quito mi overol y la ropa sucia . . .
TRANSLATION: *They don't leave work, yeah. The overalls and shirt, I leave in the wash, and I wear my pants, like this, I worked yesterday with this . . . there's no problem. I always take off my overall and the dirty clothes . . .*

MODERATOR: ¿También?
TRANSLATION: *As well?*

MAN: Pues, la mayoría del tiempo, pues, tienen, bueno, yo me he cambiado todo el tiempo, y, pues, dejo aquí lo sucio ya . . .
TRANSLATION: *Well, the majority of time, well, they have, well, I have changed all the time, and, well, I leave the dirty stuff here . . .*

WOMAN: Todos, creo. Bueno . . .
TRANSLATION: *Everyone, I believe. Well . . .*

MODERATOR: ¿Igual que las botas, también, las dejan acá, todo eso?
TRANSLATION: *Same with the boots also, you guys leave them here, all that?*

MAN: Sí.
TRANSLATION: *Yeah.*

MODERATOR: Okay. Y . . . ah, no . . . es sobre la leche cruda que se maneja acá la granja. ¿Cómo maneja la leche cruda y si hacen algún producto, algo, si se toma leche, o no sé? ¿Cuál es como el manejo usual?
TRANSLATION: *Okay. And . . . ah, no . . . it's about the raw milk that is managed here on the farm. How is raw milk managed and if there is a product being made here, anything, or if you drink the milk, or I don't know? What is like the usual management?*

MAN: La leche cruda . . .
TRANSLATION: *The raw milk . . .*

MAN: No . . .

MAN: No . . .

MAN: . . . no tiene que estar expuesta por periodos largos. Si es que es, lo único que está expuesto aquí es la leche que colectan del grupo de vacas frescas . . . ordeñan dos, tres vacas en un . . . y acabando su grupo de vacas frescas, la leche, la vienen y la depositan en el tanque de la pasteurizadora para darle de comer a las terneras. Ese . . . todo esto. Entonces, pues, es una manera de . . . de la leche que colectamos del hospital.

Toda esa leche cae ahí y se . . . toda la leche calostro que se colecta ahí, según primera . . . va a parar ahí. Y no dura mucho expuesta. Esa es la única leche, y se puede ver aquí en esta área. La otra área donde se pone, tenemos leche cruda, raw milk, pues, en el área de parto. Ahí colectamos para darles de comer a las becerritas, a los becerros. La colectamos, la pasterizamos, tenemos una pasteurizadora allá, y la congela donde la congeladora . . . de calidad. Y también dura muy poco tiempo expuesta esa lecha . . .
TRANSLATION: *. . . it's not supposed to be exposed in the open for long periods. If it is, the only thing that is exposed in the open here is the milk that is collected from the group of fresh cows . . . they milk two, three cows in . . . and after finishing with the group of fresh cows, the milk, they bring it and deposit it into a pasteurizing tank in order to feed the calves. That . . . all that. So, well, it's a way of . . . of the milk that is collected from the hospital.*

All that milk falls in there, and it . . . all the colostrum is collected there, based on the first . . . ends up there. And it doesn't last for long if left exposed. That's the only milk, and it can be seen here in this area. The other area where it's put, where we have raw milk, raw milk, is, well, in the birthing area. There we collect it in order to give it to the calves. We collect it, we pasteurize it, we have a pasteurizer over there, and we freeze it in the freezer . . . of quality. That too lasts for very little time if that milk is exposed out in the open . . .

MAN: Aquí el rancho no hace ningún producto de leche. Aquí toda . . . se procesa en la pasteurizadora para los becerros. Y la demás leche cruda va, sin que nadie la toque, al tanque, y del tanque al tráiler. Nadie hace ningún producto.
TRANSLATION: *Here, the farm doesn't make any milk products. Everything here . . . is processed in the pasteurizer for the calves. The rest of the raw milk goes, without anyone touching it, into the tank, and from the tank to the trailer. No one makes any products.*

MAN: No tenemos autorización para la leche. Está prohibido.
TRANSLATION: *We don't have authorization for the milk. That is prohibited.*

MODERATOR: Mm-hmm, y ¿qué piensan sobre esa regla? O sea, ¿está bien, está mal?
TRANSLATION: *Mm-hmm. What do you guys think about that rule? Like is that good or is that bad?*

MAN: Para mi, está bien.
TRANSLATION: *For me, it's good.*

MAN: Está bien esa regla, también.
TRANSLATION: *For me, that rule is good too.*

MAN: Yo creo, yo era, soy de las personas que me gusta usar, si se puede, usar leche cruda para . . . de allá, de la tierra de uno, que solo trabajas con leche de ese tipo. Entonces llevas en la casa, se, no le pasteurizas. Pues . . . nosotros tenemos unos platillos que se necesita . . .
TRANSLATION: *I think, I was, I'm one of those people that I like to use, if you can, use raw milk to . . . from there, from one's country, where you only work with milk of that type. And so you bring it home, and you don't pasteurize it. So . . . we have some dishes that it's needed . . .*

MODERATOR: O sea, ¿que hay veces que igual tienen cruda en la casa?
TRANSLATION: *So like are there times that you have raw milk at home too?*

MAN: Yo, yo de aquí, no, yo de aquí no, no, no, pues . . .
TRANSLATION: *I, from here, no, from here, no, no, no. Like . . .*

MAN: O sea, acá está prohibido llevar leche de aquí. No está, nadie puede agarrar leche así no más.
TRANSLATION: *Like here, it's prohibited bringing milk from here. It's not, no one can grab milk just like that.*

MODERATOR: Claro, pero alguna vez ustedes o algún compañero toma leche cruda, saben si . . .
TRANSLATION: *Of course, but have you or your coworkers ever drank raw milk, do you know if . . .*

MAN: No.

MAN: No, de esto, no.
TRANSLATION: *From here, no.*

WOMAN: No.

MAN: Tomar leche . . .
TRANSLATION: *Drinking milk . . .*

MODERATOR: Okay. Estas, sí, que son las últimas preguntas. Bueno, me gustaría hacerles algunas preguntas sobre el uso de antibiótico. Como ustedes saben, los antibióticos son medicamentes que se usan para ayudar a una persona o un animal con alguna infección bacteriana. ¿Y cuál es, primer . . . cuál es su experiencia tomando antibiótico? Su experiencia. Alguna vez han tomado antibiótico cuando, sí, aquí, no.
TRANSLATION: *Okay. These are the last questions, for sure. Well, I would like to ask a couple of questions about the use of antibiotics. As you guys know, antibiotics are medications that are used to help out a person or an animal with some sort of bacterial infection. And what is, first . . . what is your experience in taking an antibiotic? Your experience. Have you ever taken antibiotics when, yes, here, no.*

MAN: Penicilina.
TRANSLATION: *Penicillin.*

MODERATOR: ¿Penicilina?
TRANSLATION: *Penicillin?*

MAN: Tenía infección.
TRANSLATION: *I had an infection.*

MAN: Pero has tomado de, no de los animales, ¿no?
TRANSLATION: *But you have taken, not the animal ones, right?*

MODERATOR: No, no, no. Es como cuando ustedes han tenido que tomar antibiótico.
TRANSLATION: *No, no, no. It's like when you guys have had to take antibiotics.*

MAN: Como mi dentista, cuando tenía infección bocal, me lo recetó . . . no mucho, pero, sí, un poquito. Me lo recetó cuatro días. Entonces, pero . . .
TRANSLATION: *Like my dentist, when I had an oral infection, I was prescribed . . . not a lot, but, yeah, a little. I was prescribed it for four days. So but . . .*

[Simultaneous discussion]

MAN: . . . así solo, ¿no? Me tenían que recetar, como mi dentista . . . hace el año pasado, me inflamó por la ansiedad y . . . cuatro días no más.
TRANSLATION: *. . . just that, right? They had to prescribe it to me, since my dentist . . . last year, they got swollen due to my anxiety and . . . just for four days.*

MAN: Pues, a veces la verdad, a mi me dolió este lado. No tiene mucho, tendrá como unas dos semanas. Yo pensé que era el apéndice o algo así por el estilo. Pero antes mi camarada, Freddy, que trabajo con otro en la noche, me habló, me dijo, oiga, ¿me puedes ir a trabajar por mi? Yo no puedo, me duele, estoy en el doctor y se me duele aquí. Y ya, yo me acordé . . . que yo vine por . . . para el hospital y todo. Y ya, más adelante, yo me acorde que se ha ido al hospital por lo mismo, también.

Ya llegó al hospital y dice, y le pregunté que, ¿qué le habían dicho, pues? Dice, mira, yo fui al hospital y me dijeron que no más era un gas que yo tenía. Y la verdad empiezo a creer que, sí, es cierto, a veces uno no más, llegas al hospital, te estás muriendo, llegas al hospital, te estas muriendo, de allí, llegas a tu casa y estás bien otra vez. El dolor de aquí me dolía bastante la verdad. Sentía como algo así, como inflaba así en este lado. Ya . . . se me quitó. Y lo mismo le dijeron a él que no más era un gas que tenía o una tripa torcida no más.

TRANSLATION: *Well, in reality, sometimes I've had some pain on this side. It hasn't been for a long time, might be almost two weeks. I thought it was the appendix or something of that type. Before however, my friend, Freddy, who worked with another person during the night, he asked me, hey, could you go work in place of me? I can't go, I feel pain, I'm with the doctor and it hurts right here. So yeah, I remembered that . . . that I came for . . . to the hospital and everything. So, yeah, after that, I remember that he had gone to the hospital for the same thing.*

He got to the hospital, and he said, and I asked him what they had told him. He told me, look, I went to the hospital, and they told me that it was just some gas that I had. So in reality, you start to believe that, yeah, it's true, sometimes you just, you go to the hospital, you're dying, you get to the hospital, you're still dying, from there, you arrive back home, and you're fine again. To tell you the truth, the pain that I felt here hurt me a lot. I felt as if something like inflamed on this side. Yeah . . . it went away. They told him the same thing, that it was just some gas that he had or perhaps just a twisted intestine.

MAN: Mi experiencia con antibióticos, una vez tenía una infección en el estómago. Y fui al hospital y me dieron un tipo de tratamiento que es un derivado de penicilina. So el doctor me dijo, te vas a mejorar pronto. Pasaron tres días, a los tres días, no mejoraba y fui al doctor, le digo, oiga, doctor, yo estoy así de que, si un antibiótico no le funciona a uno . . . le dije . . . me puede dar otro tipo de antibiótico.

Ya me tomó. Cómo quieres que . . . no sé, le dije mire, porque yo trabajo con animales. Y si no me ha trabajado un antibiótico, entonces yo le pongo otro. Entonces yo le sugiero a usted que si usted me podía cambiar de un derivado penicilina a un derivado de sulfa(?). Me dijo, oh, entonces, si puedo hacerlo, lo voy a hacer. Entonces me dio un derivado de sulfa. Me funcionó bien.
TRANSLATION: *My experience with antibiotics was that one time I had an infection in my stomach. I went to the hospital, and they gave me a treatment that was derived from penicillin. So the doctor told me that you're going to get better soon. Three days passed, and after three days, I wasn't getting better, and I went back to the doctor. I told him, hey, doctor, I'm still like this, and if an antibiotic doesn't work for you . . . I told him . . . he could give me another type of antibiotic.*

He didn't understand it. How do you want me to . . . I don't know. I told him, look, because I work with animals. If an antibiotic doesn't work on them, I give them a different one. So I'm suggesting if you could change me from a derivative of penicillin to a derivative of sulfa(?). He told me, oh, if I can do it, I will. So he gave me one that was derived from sulfa. It worked well on me.

MODERATOR: ¿Alguien más ha tomado antibiótico? Todo . . .
TRANSLATION: *Has anyone else taken antibiotics? Everyone . . .*

WOMAN: Sí.
TRANSLATION: *Yes.*

WOMAN: Sí.
TRANSLATION: *Yes.*

MODERATOR: ¿Frecuentemente o no mucho?
TRANSLATION: *Frequently or not much?*

[Simultaneous discussion]

WOMAN: No mucho.
TRANSLATION: *Not much.*

MAN: No mucho.

TRANSLATION: *Not much.*

MAN: Muy raro.
TRANSLATION: *Very rare.*

MODERATOR: Y, por ejemplo, y si cuando tienen que tomar, ¿cómo lo hacen para conseguirlo? O sea, ¿dónde lo consiguen?
TRANSLATION: *For example, when you have to take it, how do you guys get it? Like where do you guys get it from?*

MAN: Como yo, por la receta de la doctora, fui a la Wal-Mart, me mandó a la Wal-Mart. Allá tenía mi, tenia todo ya etiquetado. No más llegué y di mi nombre y ya, ahí me dieron.
TRANSLATION: *Like I get it prescribed from the doctor. I go to Walmart, they send me to Walmart. Over there, they have it for me, already labeled. I just showed up and said my name, and that's it, they gave it to me there.*

WOMAN: Igual.
TRANSLATION: *Same.*

MODERATOR: ¿También, con prescripción del doctor?
TRANSLATION: *With a prescription from the doctor as well?*

WOMAN: Sí . . .
TRANSLATION: *Yes . . .*

MODERATOR: Y, bueno, como mencionaron ustedes algunas veces los antibióticos dejan de funcionar. Y no, ya no . . . bacteria, en una persona o en un animal. Esto es lo que se le llama resistencia antibiótica. Bueno, ¿qué piensan ustedes sobre la resistencia antibiótico? Alguna vez, bueno, usted ya me comentó que le había pasado algo por el estilo, ¿alguna de ustedes o algún familiar ha tenido alguna infección resistente a antibiótico?
TRANSLATION: *As you guys have mentioned, antibiotics stop working sometimes. And they don't . . . bacteria, in a person or in an animal. This is what's called antibiotic resistance. Well, what do you guys think about antibiotic resistance? Has there been a time, well, you've already mentioned to me that something of that nature has happened to you, have any of you or a family member had an infection be resistant to an antibiotic?*

MAN: Yo, sí, he conocido.
TRANSLATION: *Yeah, I know of someone.*

MODERATOR: ¿Sí? A usted o . . .
TRANSLATION: *Yeah? To you or . . .*

MAN: No, no. Alguien que conozco, que conocí . . .
TRANSLATION: *No, no. Someone I know, that I knew . . .*

MODERATOR: Y . . .
TRANSLATION: *And . . .*

MAN: Pues, hubo un seguimiento . . . porque no estaba funcionando, y se le cambió, y . . .
TRANSLATION: *Well, there was monitoring . . . because it wasn't working, and they changed it, and . . .*

MODERATOR: Y mejoró, ya.
TRANSLATION: *They got better, yeah.*

MAN: Yo tuve, uno de mis niños tuvo una infección. Bueno, no fue infección. Él nació con un riñón más chico que otro, entonces el conducto que va entre el riñón a la . . . la bolsa de orina era demasiado grande. Entonces los doctores, para prevenir la, algún tipo de infección, le pusieron en un antibiótico que era una dosis muy baja. Entonces el antibiótico lo que hizo es que le adquirió los anticuerpos naturales del niño, entonces empezó a entrar el flujo de lo que había en el . . . al riñón.

Entonces se provocó una infección porque el antibiótico que le pusieron, le lajeó el sistema. Entonces le siguieron, le subieron un poquito la dosis. Cuando lo trataron, no tenia infección, pero el doctor dijo, estos casos siempre se infectan. Entonces le subieron un poquito la dosis y lo tuvieron como un año con esa dosis. Y llego el momento que él tuvo fiebre, lo llevamos al hospital, y aún tenía infección. Entonces le dije al doctor, está bajo tratamiento. Luego, mi hijo, lo mandamos a un especialista, a Madison.

El especialista dijo, oh, es que lo que pasa, este antibiótico ya se hizo amigo con esta bacteria. Entonces ahorita están compaginado los dos. No podemos matarlo con estos. Hagamos un estudio y damos un antibiótico diferente para atacar esto. Porque ya el antibiótico y la bacteria que tanto tiempo viviendo juntos, se habían hecho, entonces atacaron con otro antibiótico y desapareció todo. Ultimo, después creció todo normal y ya, desapareció. Pero con la resistencia antibiótica, bien difícil. Ya lo miraba por todo, dos veces por mes lo miraba al especialista. Sí, una clínica en Milwaukee.
TRANSLATION: *I had, one of my children had an infection. Well, it wasn't an infection. He was born with a kidney that was smaller than the other one, so the duct that goes between the kidney and the . . . the urine bag was too big. And so the doctors, in order to prevent some type of infection, they gave him an antibiotic that was a very small dose. What the antibiotic did was acquire the natural antibodies of the child, and it began to get into the fluids that were in the . . . into the kidney.*

So an infection occurred because the antibiotic that they gave him, slowed down his system. So they continued, they raised his dosage a little bit more. When they treated him, he didn't have an infection, but the doctor told me, in these cases, there is always an infection. So they raised his dosage a little bit, and he was with that dosage for about a year. A time came when he had a fever, we took him to the hospital and . . . he still had an infection. So I told the doctor that he was on treatment. Later we took my son to a specialist in Madison.

The specialist told us, oh, so what's happening is that the antibiotic became friends with this bacterium. What's happening now is that they are both combined. We can't kill it with this. Let's do a study and give him a different antibiotic to attack it. Because since the antibiotic and the bacterium were living together for such a long time, they had become, and so they attacked with another antibiotic, and everything disappeared. Finally, it grew normally after, and then it disappeared. But with the antibiotic resistance, very difficult. I checked him for everything, two times a month, the specialist checked him. Yeah, in a clinic in Milwaukee.

MODERATOR: Y, bueno, ¿qué riesgo piensan que representa para ustedes o otras personas que una infección se haga resistente a antibiótico?
TRANSLATION: *Well, what type of risk do you guys think it represents for you or other people that an infection become resistant to an antibiotic?*

MAN: ¿Qué es lo que representa?
TRANSLATION: *What does it represent?*

MODERATOR: O sea que, un riesgo. ¿Si ustedes piensan o no que un riesgo exista, este tipo de resistencia, para ustedes o para las comunidades?

TRANSLATION: *Like a risk. If you guys think, or not, if there is a risk that exists, this type of resistance, for you or for the community?*

MAN: Pudiera ser potencialmente, sí, algo . . .
TRANSLATION: *It could be potentially, yes, something . . .*

MAN: Si hay . . .
TRANSLATION: *If there is . . .*

MAN: . . . peligroso para uno en el supuesto caso que uno contrajera ciertas enfermedades.
TRANSLATION: *. . . dangerous for you in the case that you contract certain illnesses.*

MAN: Porque se hacen inmune, ¿verdad?
TRANSLATION: *Because they become immune, right?*

MAN: Y que puede ser inmune al antibiótico que debería atacar a esa enfermedad. Entonces . . .
TRANSLATION: *And that it can be immune to the antibiotic that supposedly should attack that sickness. So . . .*

MAN: Yo pienso que es el riesgo mas grande que existe que un, que, pues que alguien tenga resistencia al antibiótico porque se hace un mutante. Entonces una persona que va al doctor y tiene la, ese tipo de problema, resistencia al antibiótico, es igual que si no fuera. Porque su bacteria no está muriendo. Pues, entonces . . . para que se den cuenta que tiene resistencia. La persona se grava demasiado, depende qué tipo de problema sea. Para mi el riesgo más grande que existe, más grande, en la medicina.
TRANSLATION: *I think that it's the greatest risk that exists, that someone has a resistance to an antibiotic because it becomes a mutant. So a person that goes to the doctor and has that type of problem, antibiotic resistance, it's the same as if it they didn't go. Because their bacteria isn't dying. So . . . so they can find out that they are resistant, the person becomes extremely worse, depending on what problem it is. That, for me, is the greatest risk that exists, the greatest in medicine.*

MODERATOR: ¿Alguien más quiere comentar algo? ¿Qué papel piensan ustedes que la granja lechera juega en el tema de las resistencias antibióticos?
TRANSLATION: *Does anyone else want to comment something? What role do you think dairy farms play within the topic of antibiotic resistance?*

MAN: El 30%.
TRANSLATION: *Thirty percent.*

MODERATOR: ¿Por qué así? ¿Cómo así?
TRANSLATION: *Why that? How so?*

MAN: Porque . . . de carne, solamente la de pollo, la de cerdo . . . todo eso . . .
TRANSLATION: *Because . . . of meat, just chicken, pork . . . all that . . .*

MODERATOR: ¿Pero ustedes piensan que tiene alguna relación la granja lechera con el hecho que existe resistencia al antibiótico?
TRANSLATION: *But do you guys think that there is some relation between dairy farms and the fact that resistance to antibiotics exists?*

MAN: Yo pienso que sí.

TRANSLATION: *I think so.*

MODERATOR: ¿Sí?
TRANSLATION: *Yeah?*

MAN: Más bien, estoy seguro.
TRANSLATION: *Actually, I'm certain.*

MAN: Cuando no se trata bien el problema de las enfermedades. A veces se usa medicina incorrecta. Si yo como, si yo estoy tratando un animal, y mas específicamente una becerrita, trabajaba con el dueño y decía, las becerritas son como las babies o como un niño, y este es el futuro de la granja. Depende de la cantidad de medicinas que le metan a una becerra, va a hacer problema en la granja en producción de leche, en dinero, y enfermedades. Una becerrita que tenga, antes cuando yo trabajaba con él, tuvimos mucho problema por que estaba mal organizado.

Yo comencé con él, no sé, antes, ellos tienen mas tiempo, pero cuando yo recién entré aquí, fue en el 2006, ellos comenzaban a, creo que apenas estaban comenzando a hacer de manejo de becerritas, la crianza. Entonces tuvieron mucho problema, neumonía, diarrea. Entonces comenzaban a meter . . . algo de medicina. Entonces llegó el veterinario, y dijo, ¿saben qué? Esto, esto, y esto. Y comenzaron a dar las practicas de manejo de medicina. Entonces ya tenía que tratar . . . porque si le metía más medicina . . .
TRANSLATION: *When the illness problem isn't treated well. Sometimes incorrect medicine is used. If I'm treating an animal, and more specifically a calf, I worked with the owner, and he said, calves are like babies or like a child, and this is the future of the farm. Depending on the quantity of medicines that they use on a calf, it's going to cause a problem in milk production, with money, and illnesses. A calf that has . . . before when I worked with him, we had a lot of problems because it was disorganized.*

I started out with him, I don't know, before. They have more time here, but when I just started working here, it was in 2006, they started to, I think they were just starting to do calf management, in the upbringing of the calves. They had a lot of problems, pneumonia, diarrhea. They started injecting . . . some type of medicine. The veterinarian came and said, you know what, this, this, and this. They then started giving medicine management practices. I had to start to . . . because if I kept injecting more medicine . . .

MAN: Él no puede estar ahí.
TRANSLATION: *He can't be there.*

[Simultaneous discussion]

MAN: . . . podía hacer, y hubieron casos que esos becerros murieron . . . ese es mi punto . . .
TRANSLATION: *. . . could do, and there were cases were those calves died . . . that's my point . . .*

MAN: Yo, contestando la pregunta, yo sí creo que tienes que ver el manejo y el conducto del rancho respeto a la comunidad que pudiera agarrar una bacteria.
TRANSLATION: *Answering the question, I do think that you have to look at the management and the conduct of the farm in respect to the community that could contract a bacterium.*

MODERATOR: ¿Y quién en la granja está autorizado, por ejemplo, para usar antibióticos en los animales?
TRANSLATION: And who in the farm is authorized, for example, to use antibiotics on the animals?

MAN: ¿Las personas autorizadas?
TRANSLATION: *The people who are authorized?*

MODERATOR: Mm-hmm.
TRANSLATION: *Mm-hmm.*

MAN: Pues . . .
TRANSLATION: *Well . . .*

[Simultaneous discussion]

MAN: . . . son un grupo de, seremos cuatro, somos como cinco . . .
TRANSLATION: *. . . a group of, we will be four, we're like five . . .*

MAN: Cuatro, cinco . . .
TRANSLATION: *Four, five . . .*

MAN: . . . seis, siete.
TRANSLATION: *. . . six, seven.*

MAN: Aquí presentes estamos uno, dos, tres, cuatro, cinco, seis. Falta otra persona. Son siete personas.
TRANSLATION: *Those present here, we are one, two, three, four, five, six. One person is missing. They're seven people.*

MODERATOR: Ya. ¿Hay solamente cierta cantidad de personas autorizadas?
TRANSLATION: *Okay. Is there only a certain amount of people who are authorized?*

MAN: Sí.
TRANSLATION: *Yes.*

MAN: De ahí, nadie mas puede dar medicina.
TRANSLATION: *Apart from that, no one else can give medicine.*

MODERATOR: ¿Y qué piensan sobre esa regla?
TRANSLATION: *What do you guys think about that rule?*

MAN: Que está bien. Para mi, está bien.
TRANSLATION: *That it's fine. For me, it's okay.*

MAN: Sí . . .
TRANSLATION: *Yes . . .*

MODERATOR: ¿Sí?
TRANSLATION: *Yeah?*

MAN: . . . estar controlado.
TRANSLATION: *. . . to be controlled.*

MAN: Eso es lo que . . . el control . . .
TRANSLATION: *That is what . . . the control . . .*

MODERATOR: Uh-huh.
TRANSLATION: *Uh-huh.*

MAN: . . . debe saber lo que está . . . que si le ponen medicamento . . . se va a la carne a venderse.
TRANSLATION: *. . . should know what is . . . if they put medication . . . it goes in the meat to be sold.*

MAN: Aquí la persona encargada del rancho, él . . . ha tratado de minimizar lo menos que se pueda las personas que tienen acceso con los antibióticos, por cuestiones de seguridad de la leche, que no sea contaminada por antibióticos. Y de aparte, implementamos control . . . unos reportes que nos hacen . . . el manejo de esos antibióticos, por quién se usó y qué se usó.
TRANSLATION: *The person that is in charge of the farm, he . . . has tried to minimize the amount of people that are able to have access to the antibiotics, for issues of safety of the milk, in order for it to not be contaminated by antibiotics. And aside from that, we implement control . . . some reports that they make us . . . the handling of those antibiotics, who used it and what was used.*

MAN: Y por qué se usó.
TRANSLATION: *And why it was used.*

MODERATOR: Y por qué.
TRANSLATION: *And why.*

MAN: Y a qué vaca se lo puso . . .
TRANSLATION: *And what cow it was used on . . .*

MAN: Y el día que se usó, y todos esos datos están continuamente alimentando la computadora.
TRANSLATION: *And the day that it was used, and all that information is continuously added into the computer.*

MAN: Cuándo fue la primera vez que se le puso, y cuando fue su último día.
TRANSLATION: *When was the first time it was used, and when was its last day.*

MODERATOR: Claro. Sí. ¿Alguien mas quiere compartir algo en respeto a esto?
TRANSLATION: *Of course. Yeah. Anyone else want to share something in respect to this?*

MAN: Le decía, verdad, que casi estoy seguro de que los ranchos están en, o sea, son responsables porque el señor de aquí, a los granjeros, siempre nos manda a nosotros a . . . así, y nos manda a las plantas. Todavía tenemos un poquito de conocimiento cuantos animales llegan a la planta contaminados, que en la planta los encuentra contaminados. Entonces miramos que la mayor parte de animales que van . . . de antibióticos, son de las granjas productoras de leche. Sí.

En el caso de nosotros, como decía el señor, muy poco, o sea, no tenemos ese problema últimamente. Pero vemos que las otras granjas lecheras, sí. Una de las cosas que se mira aquí que cuando vamos a una junta, por decir este muchacho, van cuatro de aquí a una junta que hay por ahí en . . . muy pocas personas de los ranchos alrededores van. Muy pocos. Eso indica que los empleados tienen muy mala preparación . . .
TRANSLATION: *I was telling you that I'm pretty sure that the farms are, like, they're responsible because the guy here, to the farmers, he always sends us to . . . like that, and he sends us to the plants. We still have a little bit of knowledge as to how many contaminated animals arrive at the plant, of those that are found in the plant contaminated. We're seeing that the majority of the animals that are going . . . antibiotics are those from dairy farms. Yeah.*

In our case, like the man said, very little, I mean, we don't have that problem lately. But we see that other dairy farms do. One of the things you see here is when we go to a meeting, this guy, four of them go from here to the meeting that there is over there in . . . very few people from the surrounding farms go. Very few. That means that the employees are very unprepared . . .

MAN: Sí . . . son muy importantes. Mucha gente nueva aprende y la gente que ya, como ellos saben, muchos, pues, aprenden un poquito más y la gente que apenas estamos aprendiendo, a veces en la junta, aprendemos de ellos. Pues, son muchas experiencias . . . demasiada experiencia que, a veces . . . otra gente, como la gente que vamos comenzando en medicina. Hicimos eso, a mi me pasó. Para la otra ya no me va a pasar. Y ya tiene un poquito de enfoque en lo que te dice las personas, lo que recolectas y llevas a la granja, a tu área . . .
TRANSLATION: *Yeah . . . are very important. A lot of new people learn and the people who already know, a lot of them, well, they learn a little bit more. The people, like us who are just learning, sometimes at the meetings, we learn from them. They are a lot of experiences . . . a lot of experience that, at times . . . other people, people like us who are just beginning with medicine. We did that, that has happened to me. Next time, it won't happen to me again. There is already a little bit more of attention on what people tell you, what you take in and take back to the farm, within your area . . .*

MODERATOR: Bueno, esa eran solamente las preguntas que teníamos. Muchas gracias por participar. Ha sido muy útil la información que nos entregaron. No sé si tienen alguna pregunta, una consulta, ¿algo mas? ¿Sí?
TRANSLATION: *Well, those were the only questions that we had. Thank you for your participation. The information that you have given us is very helpful. I don't know if you have a question, an inquiry, anything else? Yeah?*

MAN: Yo, sí. ¿Cada qué tiempo hacen la evaluación de las drogas?
TRANSLATION: *I do. How often are the drugs evaluated?*

MODERATOR: ¿Con cada cuánto tiempo?
TRANSLATION: *How often?*

MAN: ¿Cada cuántos años hacen la evaluación de las drogas?
TRANSLATION: *Every how many years are the drugs evaluated?*

MODERATOR: Pero qué, quién . . .
TRANSLATION: *But what, who . . .*

MAN: Oh, el FDA.
TRANSLATION: *Oh, the FDA.*

MODERATOR: Oh, ah, I don't know. No sé. Do you know, like, how often do they, like the FDA or something, test the drugs, like antibiotics . . . if they have some like protocol or something?

WOMAN: For testing the milk or testing like . . .

MODERATOR: No, no, like the, like antibiotics and how they work.

WOMAN: Oh, yeah. I'm not sure.

MAN: Le pregunto . . .
TRANSLATION: *I'm asking . . .*

MAN: . . . perdón que le interrumpa a todos, no será algo que tenga, es una pregunta más bien con el que produce los antibióticos de laboratorio que se encarga, ¿no será algo de los encargados de todo esto?
TRANSLATION: *. . . sorry for interrupting everyone, wouldn't it be something that has, it's a question more for who produced the antibiotics from the laboratory who is in charge, wouldn't it be something for the people in charge of all of this?*

MODERATOR: Bueno, yo . . . testear cómo trabaja el antibiótico, cómo funciona . . .
TRANSLATION: *Well, I . . . testing how the antibiotic works, how it functions . . .*

MAN: Claro, claro que eso es autorizado por el departamento de comida . . .
TRANSLATION: *Of course, of course it's authorized by the food department . . .*

MAN: Le preguntaba porque, un ejemplo, hoy salió una medicine y sale con ciertos labels, dentro de, a poco tiempo, se escucha decir, oh, esta medicina tiene tales restricciones. Un ejemplo, el Banamine.
TRANSLATION: *I'm asking you because, for example, a medication came out today and it comes with certain of labels, for which, shortly after, you hear that, oh, this type of medication has such restrictions. For example, Banamine.*

MAN: Oh, sí, es cierto.
TRANSLATION: *Oh yeah, that's true.*

MAN: Quince años atrás, nosotros, yo todavía guardo los libros de los productores y de los doctores, ¿verdad? Diez años atrás, el Banamine, o quince años atrás, no tenía . . .
TRANSLATION: *Fifteen years ago, we . . . I still have the books of the producers and the doctors, right? Ten years ago, Banamine, or fifteen years ago, it didn't have . . .*

[Simultaneous discussion]

MAN: Van variando.
TRANSLATION: *They vary.*

MAN: El Naxcel . . .
TRANSLATION: *Naxcel . . .*

MAN: Hablamos del furosemide . . .
TRANSLATION: *Let's talk about the furosemide . . .*

MAN: El Naxcel no tenía . . . so ahora las tenemos, pues. Por eso, yo me pregunto, ¿cada qué tiempo hacen la evaluación?
TRANSLATION: *Naxcel didn't have . . . so now we have it. Because of that, I'm wondering, how often do they do the evaluation?*

MODERATOR: La verdad, no sé. O sea . . .
TRANSLATION: *To tell you the truth, I don't know. Like . . .*

MAN: Hasta que se muere mucha gente, cualquier . . .
TRANSLATION: *Until a lot of people die, any . . .*

[Simultaneous discussion]

MODERATOR: . . . tampoco estaba segura.
TRANSLATION: *. . . I wasn't sure either.*

MAN: Que sí, porque . . .
TRANSLATION: *Yes, because . . .*

MAN: Porque falta . . . medicamentos.
TRANSLATION: *Because . . . missing . . . medications.*

MAN: La verdad, sí, sí, me asombré de eso, eso que me dijo él el otro día.
TRANSLATION: *In reality, yes, yes. That amazed me, that he told me the other day.*

MAN: Mi pregunta es, si el . . . por 20 años . . .
TRANSLATION: *My question is, if the . . . for 20 years . . .*

MODERATOR: Es que cuando les autorizan, cuando, para vender, para, o sea, muchos tienen que . . .
TRANSLATION: *When they authorize them, when, to sell, for, I mean, a lot of them have to . . .*

[Simultaneous discussion]

MAN: Sí, ¿pero . . . a toda esa gente la dejamos contaminada?
TRANSLATION: *Yes. But . . . we leave all those people contaminated?*

MODERATOR: No . . .

Focus Group 10, Farm 7
Recording in Spanish

MODERATOR: Bueno entonces la idea es que se les voy a hacer una serie de preguntas. Y para contestar, es que vayan respondiendo una persona a la vez, ya él que quiera obviamente. Y si pueden silenciar sus celulares, sería ideal. Y, por ejemplo, si quiere ir al baño o tomar una llamada, obviamente que pueda hacerlo, ya. Bueno, y para empezar primero, me gustaría saber un poco sobre ustedes y su role en la granja. O sea, cuál es su rutina diaria, lo que hace, hace cuanto tiempo trabaja acá, y si tiene alguna experiencia en otra granja en Wisconsin o en cualquier otro lado. Así que . . .
TRANSLATION: *Well, so the idea is that I'm going to ask you all a series of questions. And to answer, you can just respond one person at a time, whoever wants to, obviously. And if you can silence your phones, that would be ideal. And, for example, if you want to go to the bathroom or answer a phone call, obviously, you can do that. Okay, and to start, I'd like to know a little about you all and your role on the farm. So what your daily routine is, what you do, how long you've worked here, if you have any experience on another farm in Wisconsin or wherever. Like that . . .*

MAN: . . . para allá?
TRANSLATION: *. . . over there?*

MODERATOR: Yeah.

MAN: Yo tengo tres años con la compañía. Soy mánager del parlor. ¿Qué más?
TRANSLATION: *I've been here three years with this company. I'm the parlor manager. What else?*

MODERATOR: Que si tiene alguna experiencia previa.
TRANSLATION: *If you have any previous experience.*

MAN: Sí, pues, yo trabajo con el ganado diario y pues sé un poco de medicina.
TRANSLATION: *Yes, well, I work with livestock on a daily basis, and, well, I know a little bit about medicine.*

MODERATOR: Otra experiencia en otra granja, por ejemplo, o esta es la primera . . .
TRANSLATION: Another experience on another farm, for example, or is this the first . . .

MAN: Son dos granjas de la misma compañía.
TRANSLATION: *They are two farms with the same company.*

MODERATOR: Ah, perfecto. Okay.
TRANSLATION: *Oh, perfect. Okay.*

MAN: Mm-hmm.

MAN: Yo trabajo en el área de maternidad. Soy el líder allí. Y tengo 11 años trabajando en este rancho. Y sí, yo he tenido experiencia previa en otros lados.
TRANSLATION: *I work in the maternity area. I'm the leader there. I've worked on this farm for 11 years. And, yeah, I've had previous experience in other places.*

MODERATOR: Y ¿siempre en granja lechera?
TRANSLATION: *And always on a dairy farm?*

MAN: Sí.
TRANSLATION: *Yes.*

MODERATOR: Okay. ¿Usted?
TRANSLATION: *Okay. And you?*

MAN: Yo, ¿qué le puedo decir? Yo también yo tengo diez años. Hago . . . un poco de todo, arreglar maquina, ordeñar, escrepiar . . .
TRANSLATION: *Me, well, what can I say? I've also been here for ten years. I do . . . a little bit of everything, fix machinery, milk, scrape . . .*

MODERATOR: Okay. Y tiene . . .
TRANSLATION: *Okay. And you have . . .*

MAN: Despezuñar que le llama la pata.
TRANSLATION: *I trim hooves, what we call hooves.*

MODERATOR: Uh-huh.

MAN: Sí, hago un poquito de todo. Pero es el único . . . ese de aquí.
TRANSLATION: *Yes, I do a little bit of everything. But that's the only . . . this one here.*

MODERATOR: Gracias.
TRANSLATION: *Thank you.*

MAN: Yo tengo dos años, tres años trabajando en esta granja. Trabajo en el área de maternidad. Trabajé de ordeño también un tiempo. No tengo experiencia previa. Esta, mi experiencia la he conseguido aquí. Y me dedico regularmente a sacar pastura y a tratar becerros, ayudarles a las vacas a parir, y levantar los becerritos que han parido, darles de comer, y atender la vaca.
TRANSLATION: *I've been working on this farm for two, three years. I work in the maternity area. I've worked as a milker too for a little while. I don't have any previous experience. The experience I have, I've gained here. And I also regularly feed and treat calves, help cows give birth, get the new calves up to feed them, and take care of the cow.*

MAN: Yo tengo dos años trabajando también. También hago un poquito de todo. Ordeño, escripeo, pucho. Y sí he trabajado anteriormente en otras granjas, y también lo mismo.
TRANSLATION: *I've been working here for two years too. I also do a little bit of everything. I milk, I scrape, I push cows. And I have worked on other farms before doing the same things.*

MAN: Dos años ya, pasadito de dos años. También igual ordeño, escripeo, corraleo, pues, también un poquito de todo, limpieza de cualquier cosa que se ofrezca aquí. No, no tengo experiencia en otra área.
TRANSLATION: *I've been here two years, a little more than two years. I also milk, scrape, corral, well, also a little bit of everything, cleaning whatever needs to be cleaned here. No, I don't have any other experience.*

MAN: Yo tengo 12 años trabajando aquí. E igual también hago un poquito de todo. También escrepie, ordeñe y hago despezuñar. Y sí, he trabajado en otro rancho, pero también es la misma compañía.
TRANSLATION: *I've been working here for 12 years. And same here, I do a little bit of everything. I*

also scrape, I milk, and I do hoof trimming. And, yes, I've worked on another farm, but also with this same company.

MODERATOR: Okay. Bueno. Y vamos a hablar, vamos a hacer algunas preguntas sobre de cual es como más o menos, específicamente, ¿cómo son los turnos acá en la granja? ¿Qué es lo que hacen durante el día, por ejemplo? ¿Cuál sería un día común acá dentro de sus labores?
TRANSLATION: *Okay. Good. And now we're going to talk, we're going to ask some questions about what is more or less, specifically, how are your shifts here on the farm? What do you do in a day, for example? What would a normal day be like here doing your jobs?*

MAN: Depende del área. So hay muchas áreas. Cada área tiene diferente rutina. Como maternidad, pues, es venir a ver, a relevar a las personas que trabajaron porque aquí son, todo el tiempo hay personas trabajando entonces día y noche. Y los ordeñadores, pues relevan a sus compañeros. Los que andan allá atrás limpiando y trayendo vacas, pues, igual, relevan. No más se releva y se hace lo mismo durante el día.

Maternidad, pues, igual, depende de la necesidad que tenga. Si hay cosas que hacer, se hacen al momento o lo importante son las vacas y los becerros, atenderlas, y lo demás, pues, puede esperar. Pero lo importante es atender al ganado. Y igualmente, en el hospital, donde están las vacas enfermas, allí, pues a las 6:00 empieza los turnos del día. Y a esa hora, entramos todos y cada uno a sus áreas a atender cada cosa que hace.
TRANSLATION: *It depends on the area. So there are a lot of areas. Each area has a different routine. Like in maternity, well, you come in to see, to relieve the people that are working because here, it's, there are always people working, so it's day and night. And the milkers, well, they relieve their coworkers. The ones that are in the back cleaning, and bringing in cows, well, they are relieved as well. Everyone just gets relieved, and then you go and do the same thing during the day.*

Maternity, well, same, depending on what the needs are. If there are things to do, they are done right away. The important thing is taking care of the cows and the calves, and everything else, well, it can wait. But the important thing is taking care of the livestock. And same, in the hospital, where the sick cows are, the shift starts at 6:00 in the morning. And at that time, we all come in and go to our areas to do the things that we have to do.

MODERATOR: Y por ejemplo, ¿cómo sería su turno específicamente?
TRANSLATION: *And, for example, how is your shift specifically?*

MAN: Mi turno sería venir a checar el personal, que todo esté completo. Si me hablan porque hay alguien enfermo, pues, trato de cubrirlo. Ya una vez que esté todo listo, la ordeña bien con personal, entonces ya veo hacia una maquina mala o hay algo que hacer allí afuera para atender a las vacas porque muchas veces tenemos vacas que necesitan tratamiento inmediato. Entonces tengo que ir a ayudar al hospital para ver qué es lo que hay.
TRANSLATION: *My shift would be coming in to check on staffing, that everything is complete. If someone calls me because they are sick, well, I try to cover them. And then once everything is ready, the parlor is fully staffed, then I direct my attention to a machine that needs work, or if there is something that needs to be done outside with the cows because a lot of times, we have cows that need immediate attention.*

MODERATOR: Perfecto. ¿Usted?
TRANSLATION: *Perfect. And you?*

MAN: Bueno, pues, mi rutina diaria es, pues, todos los días es diferente una cosa allí. Pero mi turno, pues, un día siempre es a las 6:00 de la mañana. Y pues, llegar a, como diario es algo diferente, pues, llegar a saber que todo esté en orden allí que no haiga problemas allí, vacas caídas,

vacas que tuvieron problemas. Y pues, todos los días es algo diferente en la semana a los movimientos, pues, que hacemos allí en el área de maternidad, pero por lo principal, llegar a mirarte, pues, que todo esté en orden y que no hay muchos problemas por allí. Sí.
TRANSLATION: *Okay, well, my daily routine is, well, every day is something different. But my shift, well, a day always starts at 6:00 in the morning. And, well, I come in, since it's different every day, well, find out if everything is in order there, and there aren't any problems, cows that are down, cows with problems. And, well, every day is different during the week with what goes on there in the maternity area. But for the most part, come in and make sure that everything is in order and that there aren't a bunch of problems there. Yeah.*

MODERATOR: Mm-hmm. ¿Usted?
TRANSLATION: *Mm-hmm. And you?*

MAN: Lo mismo. La misma rutina es siempre dependiendo del área que te toque. Si te toca separar vacas, a separar vacas. A despezuñar, despezuñar. Empieza uno a las 6:00 de la mañana. Termina a las 3:00, 4:00 de la tarde. Sería la rutina de, a veces dos días a las semanas, a veces son tres. A veces es una semana completa. Y sí, ayudarle lo que se pueda.
TRANSLATION: *Same. The routine always depends on the area you're assigned to. If you're separating cows, then separate cows. If you're trimming hooves, hoof trimming. You start at 6:00 in the morning. You finish at around 3:00 or 4:00 in the afternoon. That would be the routine for, sometimes it's two days a week. Sometimes it's three. Sometimes it's a whole week. And, yeah, just helping where you can.*

MODERATOR: Mm-hmm. ¿Y usted?
TRANSLATION: *Mm-hmm. And you?*

MAN: Mi puesto empieza a las 5:00 de la mañana. Yo me dedico de quitar la pastura vieja de las vacas para que la pastura le pueda poner nueva. Empezamos a las 5:00 de la mañana con tres corrales. De allí al, toma media hora para alimentar a cada corral. So ley doy tres corrales. Espero una hora. Luego le limpio los tres corrales. Y así va alimentando y luego quitando pastura, y así.
TRANSLATION: *My job starts at 5:00 in the morning. My job is to get the old food out so that the new food can be put down. We start at 5:00 in the morning with three corrals. From there, it takes a half an hour with each corral. So I feed three corrals. I wait for one hour. Then I clean up those three corrals. And just like that, we are putting down and picking up feed.*

MODERATOR: Mm-hmm.

MAN: Pues, mi turno depende de los que me toque hacer. Si me toca a ordeñar, entro a las 6:00 de la mañana. Y como le dijo anteriormente él, llego y relevo a los que están del turno de la noche, y ya seguir la rutina y empezar a trabajar con las vacas.
TRANSLATION: *Well, my shift depends on what I'm doing. If I'm milking, I come in at 6:00 in the morning. And like he said before, I come in and relieve the people that are working the night shift. And then I continue the routine and start working with the cows.*

MODERATOR: Y allí, si le toca ordeñar, ¿está todo el día ordeñando?
TRANSLATION: *And if you have to milk, are you milking all day?*

MAN: Sí. Todo el día trabajando.
TRANSLATION: *Yes. Working all day.*

MAN: Pues, es . . .
TRANSLATION: *Well, it's . . .*

MODERATOR: Lo mismo.
TRANSLATION: *The same.*

MAN: Prácticamente lo mismo.
TRANSLATION: *Practically the same.*

MODERATOR: ¿Y usted?
TRANSLATION: *And you?*

MAN: Pues, yo entro igual a las 6:00. Y también me toca puro despezuñar todos los días. Y los fines de semana, ayudar al encargado curar a los . . . vaca, y ya es todo lo que hago.
TRANSLATION: *Well, I come in at 6:00 too. I just do hoof trimming every day. And on the weekends, I help the manager take care of the . . . cows, and that's all that I do.*

MODERATOR: Y por ejemplo, ¿qué hacen o a quién recurren si tiene alguna duda en su labor diario?
TRANSLATION: *And, for example, what do you do, or who do you go to if you have a concern during your workday?*

MAN: Con él.
TRANSLATION: *With him.*

MODERATOR: ¿Siempre?
TRANSLATION: *Always?*

MAN: Sí.
TRANSLATION: *Yes.*

MODERATOR: Okay. ¿Por ejemplo, y usted, a quien recurre si necesita ayuda?
TRANSLATION: *Okay. And, for example, and who do you go to if you need help?*

MAN: A Google. No, pues, yo trato de, si no tengo a veces mis patrones dejan a mi aquí encargado. Como los fines de semana, ellos me dicen, no voy a estar. Pero siempre me dejan un número donde hablar si yo tengo problemas. Si yo tengo problemas con las vacas, yo tengo a una clínica veterinaria que yo puedo hablar, y ellos me asisten. Trato de no, de hecho, no lo uso casi. Pero cuando es necesario, pues, ya sé. Y regularmente, pues, él está disponible, mi patrón que es . . . y pues, a veces por teléfono solucionamos. Él me dice que hacer e igual yo a ellos, cuando hay problema y yo no estoy aquí, por teléfono tratamos de solucionar el problema.
TRANSLATION: *To Google. No, well, I try to, sometimes my bosses leave me in charge here. Like on the weekends, they say to me, I'm not going to be here. But they always leave me with a number to call if I have problems. If I have problems with the cows, I have a veterinary clinic that I can call, and they help me. And normally, well, he is available, my boss . . . and well, sometimes over the phone, we solve things. He tells me what to do, and I do the same with them. If there is a problem, and I'm not here, we try to solve it over the phone.*

MODERATOR: Okay. O sea, ¿Cómo sería su comunicación, frecuente o cada?
TRANSLATION: *Okay. So how is the communication, frequent or every?*

MAN: Frecuente.
TRANSLATION: *Frequent.*

MAN: Todos los días.
TRANSLATION: *Every day.*

MAN: Sí.
TRANSLATION: *Yeah.*

MODERATOR: ¿Sí? Okay. Y ¿pueden describir alguna situación en que su mánager vendría hablar con usted?
TRANSLATION: *Yeah? Okay. And can you describe a situation in which your manager would come talk to you?*

MAN: ¿Cómo?
TRANSLATION: *What?*

MODERATOR: Al revés, sino, por ejemplo, si ustedes tienen alguna duda, ustedes saben a quien pueden recurrir. Pero ¿cuándo su mánager viene hablar con ustedes?
TRANSLATION: *The reverse, instead, for example, if you have a question, you know who you can go to. But when does your manager come and talk to you?*

MAN: Cuando uno la riega. Cuando uno hace un trabajo mal que él te vea, pues, sí, puede llamarte la atención y dice lo estás haciendo mal porque no . . . lo está haciendo mal, hasta allí es lo más.
TRANSLATION: *When you screw up. When you do a bad job, and he sees you, well, he could get your attention and say, you're doing it wrong because . . . you're doing it wrong. That's it.*

MAN: Yo pienso que también cuando hay como cambio de rutina. So si hay algo extra o un cambio del plan del día, entonces, pues.
TRANSLATION: *I think that also when there is a change in routine. So if there is something extra or a change of plans for the day, then.*

MODERATOR: Se acerca hablar con usted.
TRANSLATION: *He would come talk to you.*

MAN: Sí. Tiene uno que comunicarse porque si no, pues, no funciona.
TRANSLATION: *Yeah. You have to communicate because if you don't, it doesn't work.*

MODERATOR: Y ¿Cómo funciona los descansos acá en la granja? Los breaks por ejemplo, ¿dónde comen, mientras trabajan o si tienen algún lugar y eso? Si son establecidos o se lo toman dependiendo de la cantidad de trabajo.
TRANSLATION: And how do the breaks work here on the farm? The breaks, for example, where do you eat, while you work, or do you have a place for that? If the breaks area established, or if they're just taken depending on the amount of work.

MAN: No pues, por lo regular, son establecidos. Siempre nos, ya abajo está la cocina y allí de las, los otros en el área de maternidad de las 10:00 en adelante, empezamos a tomar break.
TRANSLATION: No, well, normally, they are established. We always, down there is the kitchen. And the people in the maternity area start from 10:00 and on, we start to take breaks.

MODERATOR: Okay. ¿Y se van turnando para hacer un break?
TRANSLATION: *Okay. And you take turns for breaks?*

MAN: Pues, agarramos un break casi por lo regular cuando hacemos un poco de tiempo. Tratamos de que, a esa hora, nos de tiempo para venir al break. Sí, a veces nos turnamos, pero cuando no, pues, hacemos un poco de tiempo para venir a tomar break.
TRANSLATION: *Well, we take a break almost always when we make a little bit of time. We try to, at that time, we try to come in for a break. Yeah, sometimes we take turns. But when we don't, well, we make some time to come in and take a break.*

MODERATOR: ¿Y ustedes también?
TRANSLATION: *And you guys too?*

MAN: Sí, también.
TRANSLATION: *Yes, also.*

MAN: La ordeña, ¿Cómo es la ordeña?
TRANSLATION: *Milking, how is it for milking?*

MAN: Hay un checador que solamente se dedica a dar break.
TRANSLATION: *There is a checker whose job it is to give breaks.*

MODERATOR: Ah, okay.

MAN: Sí, le da break a una persona y ya viene y come media hora. Y ya de allí va saliendo y se va relevando a la otra persona para que nos de break a todos.
TRANSLATION: *Yeah, they give a break to one person, and then they come and eat for half an hour. And then from there, they come back and relieve the other person so that we all get a break.*

MODERATOR: Okay.

MAN: Ya para la 1:00 de la tarde, ya todos comieron.
TRANSLATION: *By like 1:00 in the afternoon, we've all eaten.*

MODERATOR: Y ¿Comen siempre, tienen algún lugar donde comen?
TRANSLATION: *And do you always eat, do you have a place to eat?*

MAN: Sí.
TRANSLATION: *Yes.*

[Simultaneous Discussion]

MAN: A la cocina.
TRANSLATION: *In the kitchen.*

MAN: Sí.
TRANSLATION: *Yes.*

MAN: Allí está un break room.
TRANSLATION: *There is a break room.*

MODERATOR: Okay. Perfecto. ¿Y por ejemplo, qué pasa con los descansos para ir al baño?
TRANSLATION: *Okay. Perfect. And, for example, what happens with the breaks to go to the bathroom?*

MAN: Hay siempre una persona que te cubre.
TRANSLATION: *There is always someone there to cover you.*

MODERATOR: Ah, ya.
TRANSLATION: *Oh, okay.*

MAN: Sí. Siempre hay alguien que puedes decir, oye, necesito yo de ir cinco minutos al baño. Esta persona viene, toma tu puesto, y tú vas.
TRANSLATION: *Yeah. There is always someone that you can say, hey, I need to go for five minutes to the bathroom. That person comes, takes your spot, and you go.*

MODERATOR: Perfecto.
TRANSLATION: *Perfect.*

MAN: Pero pues, como para maternidad, no es necesario porque nosotros en maternidad solo trabajan tres personas en el día y una en la noche. Y nosotros, si no hay una vaca pariendo o no hay nada allí, pues tienes chanza de irse a la cocina o pasar al baño.
TRANSLATION: *Well, for maternity, it's not necessary because there are three of us during the day and one at night. And we, if there isn't a cow giving birth or anything going on, well, you have a chance to go to the kitchen or go to the bathroom.*

MODERATOR: Okay.

MAN: A cualquier persona, si los ordeñadores ocupan asistencia, pues, todos sabemos ordeñar. Entonces empezamos ordeñando. Si alguna persona tiene necesidad de algo, pues . . . cubrir un momento.
TRANSLATION: *For whatever person, if the milkers need help milking, well, we all know how to milk. So we start milking. If someone needs something, well . . . cover for a moment.*

MODERATOR: Okay. ¿Alguna vez en ese descanso se van de la granja, o siempre se lo toman aquí?
TRANSLATION: *Okay. Do you ever leave the farm on your break, or you always take it here?*

[Simultaneous Discussion]

MAN: No, nunca.
TRANSLATION: *No, never.*

MAN: Aquí.
TRANSLATION: *Here.*

MAN: Todo el tiempo aquí.
TRANSLATION: *We're always here.*

MODERATOR: Y bueno, ahora vamos a pasar a las preguntas sobre el equipo de protección que ocupan para trabajar. ¿Qué reglas tiene la granja sobre su vestimenta de trabajo, o si es que hay regla? O sea, ¿qué cosas tiene que ocupar, sí o no?

TRANSLATION: *And, okay, now we are going to move onto some questions about the protective equipment that you use to work. What rules does the farm have about work clothing, if there are any rules? I mean, what things do you have to use?*

MAN: ¿Sobre vestimenta?
TRANSLATION: *About clothing?*

MODERATOR: Claro.
TRANSLATION: *Of course.*

MAN: No creo que hay.
TRANSLATION: *I don't think there are.*

MAN: Como . . .
TRANSLATION: *Like . . .*

MAN: Pues, vestimenta es todo lo que necesita.
TRANSLATION: *Well, clothing is everything you need.*

MODERATOR: Exacto.
TRANSLATION: *Exactly.*

MAN: Dependiendo del área.
TRANSLATION: *Depending on the area.*

MAN: No tiene, como nosotros que usamos los tractores, no tenemos que usar la ropa muy . . .
TRANSLATION: *We don't, like those of us that use the tractors, we can't use clothing that's very . . .*

MAN: Oscura.
TRANSLATION: *Dark.*

MAN: Suelta porque nos podemos tener accidente.
TRANSLATION: *Loose because we could have an accident.*

MODERATOR: Okay.

MAN: Y tenemos que traer las sudaderas visibles para que no tengamos algún accidente con los tractores.
TRANSLATION: *And we have to wear the visible sweatshirts so that we don't have an accident with the tractors.*

MAN: Con cinturón de seguridad.
TRANSLATION: *Wear a seatbelt.*

[Loud noise in background begins]

MAN: Usar los cinturones de seguridad cuando uno opera los tractores.
TRANSLATION: *Use the seatbelts when you operate the tractors.*

MAN: Y la ropa . . .
TRANSLATION: *And the clothes . . .*

MODERATOR: . . .

MAN: Siempre cada persona que usan equipo de motor, deben de traer la sudadera . . . para poder verse y las luces prendidas todo el tiempo.
TRANSLATION: *Every person that uses any kind of motorized equipment has to wear the sweatshirt . . . so that they can be seen, and they have to have the lights on at all times.*

[Loud noise in background ends]

MAN: Todo el momento.
TRANSLATION: *All the time.*

MAN: Y pues, no sé, los ordeñadores tienen . . .
TRANSLATION: *And, well, I don't know, the milkers have to . . .*

MODERATOR: Sí, ¿tienen algún tipo de vestimenta que tiene que ocupar para el trabajo?
TRANSLATION: *Yeah, do you have any kind of clothing that you need to wear for work?*

MAN: Pues, nos proporcionan un mandil, que es para cubrirnos de la suciedad y mangas que son bolsas y, pues, la ropa de uno.
TRANSLATION: *Well, they give us an apron, which protects us from filth, and sleeves, which are like bags and, well, your own clothes.*

MODERATOR: Y las botas o . . .
TRANSLATION: *And the boots . . .*

MAN: Botas también.
TRANSLATION: *Boots too.*

MAN: Botas también.
TRANSLATION: *Boots too.*

MODERATOR: Botas también. ¿Protección de ojos?
TRANSLATION: *Boots too. Eye protection?*

MAN: Sí, también.
TRANSLATION: *Yes, that as well.*

MODERATOR: También. ¿Alguien más que le gustaría contar si tienen alguna . . .
TRANSLATION: *That too. Would anyone else like to talk about if you have any . . .*

MAN: Pues allí donde yo estoy, pues, igual también, usamos lentes para la protección de los ojos. Usamos unas, a lo que hago pues uso una maya y un protector porque como uso una máquina para no cortarme y todo eso porque sí está moviendo. Tratamos de protegernos bien para no causarnos accidentes.
TRANSLATION: *Well, where I'm at, well, same thing, we use safety glasses for eye protection. We use some, screens and a protector so we don't cut ourselves because we use machinery, and it's moving. We try to protect ourselves well so we don't have accidents.*

MAN: o sea, que sí nos proporcionan, pues, todo el equipo que a veces uno no lo usa.
TRANSLATION: *I mean, so, yeah, they provide us with, well, all the gear that you might need.*

MAN: Es opcional de quien utiliza y quien no. Pero ellos sí te proveen con lentes, te proveen con guantes, bolsas y mandiles. Y ya decides tú si los usas o no.
TRANSLATION: *It's optional if you use it or not. But they do provide you with safety glasses. They give you gloves, bags, and aprons. And then you decide if you're going to use them or not.*

MAN: En ciertas áreas.
TRANSLATION: *In certain areas.*

MAN: En ciertas áreas. Sí, en ciertas áreas.
TRANSLATION: *In certain areas. Yeah, in certain areas.*

MAN: Porque el trabajo que hace él y él que es de usar, es una graneadora y tiene cuchillas. Entonces para cortarle la pezuña de la vaca, usan protección aquí. Esa deben de usarla todo el tiempo para, porque usan cuchillo también, muchos se han cortado aquí por no usar protección o porque la protección llega hasta aquí. Entonces muchas veces, pues, la vaca pues, se mueve, y muchas se han cortado aquí. Y una maya, deben de usar esa protección que va de aquí a acá que protege el brazo y una maya de acero que proteja toda la mano para que no quede cortado con los cuchillos.
TRANSLATION: *Because like for his job, he uses a grinder, and it has knives. So when they're trimming hooves, they use protection. They need to use that all the time, because they use knives too. Many people have been cut here before from not using protection or because the protection only comes up to here. So a lot of time, well, the cow can move, and many people have been cut here. And a screen, you should use that protection that goes from here to here to protect your arms, and a steel screen that protects your hands so that you don't get cup with the knives.*

MODERATOR: Y ¿los que trabajan con animales enfermos, tienen alguna vestimenta que tienen que usar?
TRANSLATION: *And what about those that work with sick animals, do they have any kind of clothing that they have to wear?*

MAN: No, no tenemos nada. Solamente, pues, la precaución para cuando usamos antibiótico de usar a las jeringas correctamente, tener la vaca segura cuando vayamos a suministrárselo porque, pues, se puede mover, y uno se puede lastimar. Sí.
TRANSLATION: *No, we don't have anything. Just, well, to be careful when we are using antibiotics and using the syringes correctly, making sure the cow is secure when we are going to administer it because, well, they can move, and you can get hurt. Yeah.*

MODERATOR: Okay. Y ¿qué piensan sobre el requerimiento de las reglas de vestimenta? ¿Esta bien, está mal, o a veces incómoda, no sé?
TRANSLATION: *Okay. And what do you think about the rules and requirements about clothing? Are they good, bad, or sometimes uncomfortable? I don't know.*

MAN: Yo siento que está muy bien porque para nosotros que manejamos alrededor de todo el rancho, nos ocupamos el vernos el ser fosforescente más en tiempo de neblina que los corrales se convierten completamente oscuro, y gracias a lo fosforescente que nos vemos, donde andas, eso y las luces.
TRANSLATION: *I feel like they are very good because for those of us that drive around the outside*

of the farm, we need to be seen with the fluorescents mostly when it's foggy and the corrals become totally dark. And thanks to the fluorescents we can be seen where we are, that and the lights.

MODERATOR: Claro.
TRANSLATION: *Of course.*

MAN: La compañía nos proporciona el overol a las personas que trabajamos como en ciertas áreas. A los ordeñadores, no, porque no manejan mucho ganado más bien con las manos. Pero todos los demás, tratamos de que, de todo traigamos overol, ya sea de invierno o sea de verano. Y pues, allí traemos todo lo que necesitamos, radio y todo.
TRANSLATION: *The company provides us with overalls to people who work in certain areas. Not to the milkers because they don't handle the livestock very much, except for with their hands. But everyone else, we try to wear the overalls whether it's winter, or it's summer. And, well, we carry everything we need in there, the radio and all that.*

MODERATOR: Y los guantes, por ejemplo.
TRANSLATION: *And gloves, for example.*

MAN: Todos. Guantes son provenidos y en cada área. Todos utilizamos guantes.
TRANSLATION: *Everyone. Gloves are provided in every area. We all use gloves.*

MODERATOR: Okay. ¿Todos están de acuerdo con el uso de ese tipo de vestimenta?
TRANSLATION: *Okay. All of you agree with the use of that kind of clothing?*

MAN: Con el equipo . . . sí. Sí, porque lo tenemos, como le digo, si no lo tuviéramos y lo necesitáramos, sí hubiera quejas de otra parte, pero . . .
TRANSLATION: *With the gear . . . yes. Yes, because we have it, how can I say, if we didn't have it, and we needed it, there would be complaints about that, but . . .*

MODERATOR: Claro. Y bueno, ¿hay ocasiones en las que es difícil usar el equipo de protección que ocupan?
TRANSLATION: *Of course. And, okay, are there times where you find it difficult to use the protective gear that you need?*

MAN: Es . . .
TRANSLATION: *It's . . .*

MAN: Sí.
TRANSLATION: *Yes.*

MAN: Puede ser uno incómodo, por ejemplo, yo nunca uso lentes. Para mí, es incómodo ponérmelos y usarlos. Pero, pues, cuando es necesario utilizarlos, los utilizo. Pero no los uso al diario porque, pues, para mí son incómodo.
TRANSLATION: *You can get uncomfortable, for example, I never use safety glasses. For me, it's uncomfortable to wear them and use them. But, well, when it's necessary to use them, I use them.*

MAN: Yo pienso que para los que usamos anteojos, lentes, es incómodo usar al cobre boca porque se . . .
TRANSLATION: *I think that for those of us that use safety glasses, it's uncomfortable to use a face mask because it . . .*

MAN: O use uno . . .
TRANSLATION: *Or use a . . .*

MAN: . . . no vemos, entonces . . .
TRANSLATION: *. . . we can't see, so . . .*

MAN: O use el cobre boca o usan los lentes.
TRANSLATION: *You either use the face mask, or you use the safety glasses.*

MAN: Sí. Sería lo único, allí incómodo sería en el frio que usamos demasiada ropa.
TRANSLATION: *Yes. That would be the only thing. It can be uncomfortable in the cold when we have to use so much clothing.*

MODERATOR: Ah, claro.
TRANSLATION: *Oh, of course.*

MAN: Sí, nada más. Pero en si, pues, el equipo no es incómodo ni pesado ni nada.
TRANSLATION: *Yeah, just that. But for the most part, the gear is not uncomfortable or heavy or anything.*

MODERATOR: ¿Alguien más quiere . . . y cuáles, por ejemplo, son las prácticas de lavado de manos en el trabajo? ¿Hay como una regla que dice, tienen que lavarse las manos aquí, aquí, no sé, o es más libre?
TRANSLATION: *Does anybody else want to . . . and what, for example, are the hand washing practices at work? Is there like a rule that says, you have to wash your hands here, here, I don't know, or is it more liberal?*

MAN: Donde yo estoy, pues, yo entro desde las, pues, sí, que entro a las 6:00, y a las 6:30 pues estoy comenzando a trabajar. Y uso guantes, y ya no me las quito hasta que yo acabe, hasta que ya me vengo yo a comer. Entones, ya, me quito los guantes y ya ves que hago mi lavado de mano y todo eso. Pero no hay veces de que quites los guantes y vete a lavar las manos cada rato, no, pues, no. Eso es nada más.
TRANSLATION: *Where I'm at, well, I come in at, well, yeah, I come in at 6:00. And at 6:30, well, I'm starting to work. I use gloves, and then I don't take them off until I finish, until I come in to eat. So then I take off my gloves, and then I wash my hands and all that. But there aren't times when you take your gloves off, and you go wash your hands every so often, no. That's it.*

MODERATOR: Sí, ¿Y ustedes?
TRANSLATION: *Yeah. And you guys?*

MAN: Sí, es lo mismo. También desde que entramos, no los quitamos hasta cuando vamos a tomar break.
TRANSLATION: *Yeah, it's the same. From the time we start, we don't take them off until we are going to take a break.*

MODERATOR: ¿No es que tengan así, cada cierto tiempo?
TRANSLATION: *You don't have like, every so often?*

MAN: No. De lavar las manos, no, simplemente nada más de cambiar guantes. Cada posición cuando te toca una, cada posición dura como 40 minutos. Al cambiártela, vas a cambiar los guantes nada más.

TRANSLATION: *No. For hand washing, no, just for changing gloves. Each position that you go to, every position lasts about 40 minutes. When you switch, you go change your gloves, that's it.*

MAN: En mi área, igual, son los guantes. Como nosotros nos dedicamos en agarrar vacas que han recién parido, tenemos que tener mucho cuidado en no llevar bacteria hacia ella. Y siempre es cambiar guantes, o si vas a tocarla con tu mano, que esté limpie que no trae suciedad o paja, cualquier tipo.
TRANSLATION: *In my area, it's the same, just the gloves. Since our job is to grab cows that have just recently given birth, we have to be really careful not to pass bacteria to her. And we always have to change our gloves. Or if you're going to touch her with your hand, that it's clean, and it's doesn't have filth or hay on it, like that.*

MODERATOR: ¿Ustedes igual, es como más o menos lo mismo?
TRANSLATION: *Are you guys the same, is it more or less the same?*

MAN: Sí, de hecho, tenemos que, más que lavarnos las manos, tenemos que usar guantes todo el tiempo de látex para nuestra protección también porque hay mucha infección, mucha bacteria. Todo lo que tocamos es bacteria, teléfono, radio, lo que sea tiene bacteria. Entonces tenemos obligado usar guantes. Y la bacteria . . . no más en las manos así, o sea, es obligatorio usar guantes.
TRANSLATION: *Yes, in fact, we have to, more than washing out hands, we have to use latex gloves all the time for our protection as well because there is a lot of infection and bacteria. Everything we touch has bacteria on it, the phone, radio, everything has bacteria. So we have to wear gloves. And the bacteria . . . just on your hands, so it is mandatory to wear gloves.*

MODERATOR: Y ¿qué usan, por ejemplo, ya cuando tienen que lavarse las manos? ¿Qué usan para lavarse las manos? ¿Tienen algún producto especial, desinfectante?
TRANSLATION: *And what do you use, for example, when you have to wash your hands? What do you use to wash your hands? Is there some special product, disinfectant?*

MAN: El trabajo nos provee jabón para manos que viene siendo como desinfectante.
TRANSLATION: *They provide us with soap for our hands which is like a disinfectant.*

MAN: En el área de maternidad y hospital, tenemos varios productos. Tenemos el jabón, tenemos yodo, tenemos chlorine, entonces todo eso usamos, inclusivo tenemos alcohol también para jeringas y para, puede usar es sus manos también. Entonces ya tenemos, depende del área usamos todo eso. Yo uso mucho el chlorine y el yodo por lo que manejo las vacas en todo.
TRANSLATION: *In the maternity and hospital areas, we have various products. We have soap, we have iodine, we have chlorine, so we use all of that. We even have alcohol too for the syringes, and you can use that on your hands too. So we have, depending on the area, we use all of that. And I use the chlorine a lot and the iodine because I handle the cows.*

MODERATOR: Okay. ¿Alguien más? Sí. Y cómo afecta, bueno, ¿cómo afecta el lavado de manos en su trabajo? Bueno, la verdad yo creo que está pregunta yo creo que esta de abajo, que ya me dijeron un poco. Por ejemplo, ¿hay un momento en que es más difícil lavarse las manos que en otro durante su rutina?
TRANSLATION: *Okay. Anyone else? Yeah. And how does it affect, well, how does hand washing affect your job? I mean, I think we already went over this question, and you already told me a little bit. For example, is there a moment when it's more difficult to wash your hands during your routine?*

MAN: Cuando tenemos mucho trabajo yo pienso. Sí.
TRANSLATION: *When we have a lot of work to do, I think. Yeah.*

MAN: Pues, casi no necesita lavarse las manos todo el tiempo como todo el tiempo trae guantes.
TRANSLATION: *Well, you really don't need to wash your hands all the time because you're wearing gloves all the time.*

MAN: Nada más te cambias guantes.
TRANSLATION: *You just change your gloves.*

MAN: Te cambias de guantes, y cuando ya sientes que estás sucio o vas a hacer algo con la pura mano, pues, ya.
TRANSLATION: *You change your gloves. And when you feel like you're dirty, or you're going to do something with your bare hand, then, yeah.*

MAN: Regularmente, sus manos no se ensucian en si. Siempre traes un guante protegiéndola.
TRANSLATION: *Normally, your hands don't get dirty. You always have gloves on protecting them.*

MODERATOR: Okay. ¿Y cómo se preparan para salir del trabajo para el día, por ejemplo, tienen algún lugar donde se cambian eso?
TRANSLATION: *Okay. And how do you prepare to leave work at the end of the day, for example, do you have a place where you change and all that?*

MAN: Sí. Tenemos un área de cambia donde están nuestros lockers. Cada trabajador le asignan un locker allí tiene sus cosas personales. Cuando llegas, te cambias. Aquí, mantenemos nuestra ropa de trabajo. Aquí se lava, y aquí se seca.
TRANSLATION: *Yes. We have a changing area where our lockers are. Each worker is assigned a locker where they keep their personal things. When you come in, you change. We keep the clothing we use for work here. It's washed here, and dried here.*

MODERATOR: ¿Allí tienen lavandería y todo?
TRANSLATION: *You have a laundry area and everything?*

MAN: Sí, lavandería y secadora. So uno llega allí tienes en tu área de locker, allí va a estar un montón de ropa . . . un montón. De allí, escoges lo que es tuyo, te vistes, ropa tu ropa limpia la pones en tu locker, y te puedes ir.
TRANSLATION: *Yes, washer and dryer. So you come here. You have your locker area, and there's going to be a big pile of clothing . . . a big pile. From there, you pick out what is yours. You get dressed. You put your clean clothes in your locker, and you can go.*

MODERATOR: ¿Ustedes tienen la misma rutina?
TRANSLATION: *You guys have the same routine too?*

[Simultaneous discussion]

MAN: Sí.
TRANSLATION: *Yes.*

MAN: Sí.
TRANSLATION: *Yes.*

MODERATOR: O sea, que siempre maneja la ropa sucia acá.
TRANSLATION: *So the dirty clothing is always handled here?*

[Simultaneous discussion]

MAN: Ah, sí.
TRANSLATION: *Oh, yes.*

MAN: Sí.
TRANSLATION: *Yes.*

MAN: Aquí se queda.
TRANSLATION: *It stays here.*

MODERATOR: Perfecto. Bueno, ahora vamos a pasar a preguntas respeto a la leche cruda. ¿Cómo se maneja la leche cruda acá y los productos lácteos si es que se producen aquí en la granja, si tienen contacto?
TRANSLATION: *Perfect. Well, now we're going to move on to some questions about raw milk. How is raw milk handled here and the lactose products, if they are produced here on the farm, if you have contact with them?*

MAN: Pues, la leche es toda para la compañía. So nosotros no manejamos leche cruda aquí. La leche, directamente, conectamos el tanque, y la compañía viene, la recoge, y se la lleve. So nosotros no manejamos la leche así.
TRANSLATION: *Well, all the milk is for the company. So we don't handle the raw milk here. The milk goes directly to the tank, and the company comes. They get it out, and they take it. So we don't handle the milk or anything.*

MAN: Cuando siempre que la leche solamente la vemos en la manguera y . . .
TRANSLATION: *We only just see the milk in the tube and . . .*

MAN: De allí, es pumpeada al tanque, del tanque, desaparece.
TRANSLATION: *From there, it's pumped to the tank. From the tank, it disappears.*

MAN: Si queremos leche, pues, la compramos en Walmart.
TRANSLATION: *If we want milk, well, we buy it at Walmart.*

MAN: Sí. No manejamos. Los productos nos los proporciona la compañía que se llama Cheese. . . Company. Ese, todos los productos de esa compañía nos los pueden proporcionar por medio de, nos dan una lista y todos podemos comprar queso o los productos que se hacen de esta leche.
TRANSLATION: *Yeah. We don't handle it. The company provides us with products, it's called Cheese . . . Company. They can provide us with all of the products from the company by way of, they give us a list, and we can all buy cheese or the other products they make with the milk.*

MODERATOR: Claro. Pero en ningún caso como . . .
TRANSLATION: *Of course. But there's never a time when . . .*

MAN: Que nosotros, no, no, no podemos. Tenemos prohibido usar.
TRANSLATION: *That we, no, no, we can't. It is prohibited to use it.*

MAN: Reglas.
TRANSLATION: *Rules.*

MODERATOR: Okay.

MAN: Sí.
TRANSLATION: *Yeah.*

MODERATOR: ¿Qué piensan sobre esas reglas?
TRANSLATION: *What do you think about those rules?*

MAN: Se ve bien. Evita uno contagiarse algo que no quieres. Puede tener algún químico o algo.
TRANSLATION: *They seem fine. It avoids contracting something you don't want. There could be some chemicals or something.*

MODERATOR: ¿Alguna vez ustedes o algunos de su compañeros han consumido la leche cruda? O sea, puede ser aquí, puede ser en otro lado.
TRANSLATION: *Has there ever been a time where you or any of your coworkers has consumed raw milk? I mean, it could be here. It could be somewhere else.*

[Simultaneous discussion]

MAN: Oh, en otro lado, sí.
TRANSLATION: *Oh, somewhere else, yeah.*

MAN: Yo sí.
TRANSLATION: *I have.*

MAN: En otro lado, sí.
TRANSLATION: *Somewhere else, yes.*

MAN: En nuestro país, bueno, yo soy de México, y en nuestro país, pues, no hay problema.
TRANSLATION: *In our country, well, I'm from Mexico. And in our country, well, there's no problem with that.*

MAN: No hay problema.
TRANSLATION: *There's no problem.*

MAN: Tomamos la leche directamente de, la ordeñamos en un vaso y la toman. Pero aquí no podemos porque va por políticas de la compañía. Aunque nosotros sepamos que es una vaca buena, limpia, no podemos por política de la compañía. Entonces es si nos llegamos a enfermar o otra, aunque no sea de la leche, pero, pues, no es bueno.
TRANSLATION: *We drink the milk directly from, we milk it into a cup and drink it. But here, we can't because it goes against the company policies. Even though we know that it's a good, clean cow, we can't because of the company policies. So it's so that if we were to get sick, even if it's not from the milk, well, but, it's not good.*

MODERATOR: Okay.

MAN: Yeah.

MODERATOR: ¿Y qué piensan sobre la leche cruda, o sea, de tomar leche cruda?
TRANSLATION: *And what do you think about raw milk, I mean, drinking raw milk?*

MAN: Pues, es el medicamento y tanto que le meten a la vaca dices tú, no, pues, ya no es leche . . .
TRANSLATION: *Well, it's the medicine and all that that they put into the cow. You say, no, well, it's not really milk anymore . . .*

MAN: Leche normal.
TRANSLATION: *Normal milk.*

MAN: Yeah, no. Y sin embargo en nuestro rancho, pues, sí, porque no se le aplica tanta medicina a la vaca, y la ordeña, pura pastura, aquí no. Aquí tanto la comida con todo lo que lleva es medicina. Si dan ganas a veces, pero recordar de todas las cosas te quita la gana de tomar leche cruda. Sí.
TRANSLATION: *Yeah, no. However, on our farm, well, yeah, because we don't administer so much medicine to the cow, and then milk it, just pure pasture, not here. Here even the food with all that it has in it has medicine. Sometimes you do want to, but you remember all those things, and it takes away the desire to drink raw milk. Yeah.*

MODERATOR: ¿Alguien más?
TRANSLATION: *Anyone else?*

MAN: Hay más . . .
TRANSLATION: *There is more . . .*

MODERATOR: Bueno. Y ahora me gustaría hacerle la última ronda de preguntas que es sobre el uso de antibiótico y antimicrobiano. Como alguno de ustedes saben, los antibióticos son medicamentos que se usan para ayudar a una persona o a un animal con alguna infección bacteriana. Y los veterinarios, bueno, ustedes también acá ocupan antibiótico. Y quería saber un poco cual es su experiencia tomando antibiótico. O sea, ¿en qué situaciones ustedes piensan que los antibióticos son útiles o no son útiles?
TRANSLATION: *Okay. And now I would like to ask you the last round of questions, which is about the use of antibiotics and antimicrobials. Like some of you may know, antibiotics are medicines that are used to help a person or an animal with a bacterial infection. And the veterinarians, well, you guys also use antibiotics here. And I want to know a little about what your experience is with taking antibiotics. So in what situations do you think antibiotics are useful or not useful?*

MAN: Sobre uno o . . .
TRANSLATION: *About ourselves, or . . .*

MODERATOR: Sí, sobre ustedes.
TRANSLATION: *Yes, about yourselves.*

MAN: Pues, cuando andas malo del estómago, cuando traes dolor de cabeza, ya tomas alguna pastilla para el dolor, o sea, un antibiótico.
TRANSLATION: *Well, when you have an upset stomach, when you have a headache, you take a pill for the pain, I mean, an antibiotic.*

MAN: Yo pienso que el antibiótico, pues, sí, cuando lo necesita que tiene una herida, te la tomas. Tomas algo, te inyecta algo, pero, pues, sí, sí sirven de algo porque a veces . . .
TRANSLATION: *I think that an antibiotics, well, yeah, you need it if you have wound, you take it. You take something, they inject something, but, well, yeah, it is useful for something because sometimes . . .*

MODERATOR: ¿Alguien más?
TRANSLATION: *Anyone else?*

MAN: Igual, cuando tienes, que tú regularmente, pues, uno sea todo diagnostican, verdad. Si a veces por no ir al doctor por . . . ya cuando te sientes muy mal, te vas al doctor. Pero uno se auto médica también. So uno siempre buscando, pues, si tengo una fiebre, pues algo para la fiebre. Y a veces no contamos con que la fiebre deriva de algo. A veces tenemos infección, no sé, en el oído o infección nasal, algo. Y nosotros usamos, ahora sí que los comunes que es la pastilla de ibuprofen y cosas para la fiebre es lo único que usamos es el ibuprofen.
TRANSLATION: *Also, when you have, you normally, well, you're diagnosed, right. Sometimes if you don't go to the doctor for . . . then when you're feeling sick, you go to the doctor. But you self-medicate too. So you're always looking for like, well, if I have a fever, well, something for the fever. And sometimes we don't know where the fever is coming from. Sometimes we have an infection, I don't know, in the ear or in the nose, something like that. And we use, the common pills are ibuprofen and things for fever. The only thing we use is ibuprofen.*

MODERATOR: Sí, por ejemplo, porque el ibuprofeno es un antinflamatorio. El antibiótico es cuando tiene una bacteria, por ejemplo, la penicilina y todas esas.
TRANSLATION: *Yeah, for example, because ibuprofen is an anti-inflammatory. Antibiotic is for when you have a bacteria, for example, penicillin and all of those.*

MAN: Mm-hmm.

MAN: La penicilina es prescripta, no.
TRANSLATION: *Penicillin is a prescription, right?*

MODERATOR: Claro.
TRANSLATION: *Of course.*

MAN: Sí.
TRANSLATION: *Yeah.*

MAN: So claramente.
TRANSLATION: *Of course.*

MAN: Entonces . . .
TRANSLATION: *So . . .*

MAN: Exactamente, no te la puedes . . .
TRANSLATION: *Exactly, you can't . . .*

MAN: Solo podríamos usar bajo una receta.
TRANSLATION: *We can only use it with a prescription.*

MAN: Solamente. Solamente bajo una receta.
TRANSLATION: *That's it. Just with a prescription.*

MODERATOR: Y ¿cómo se llama? ¿y qué haría para obtener antibióticos si los necesitan?
TRANSLATION: *And what's it called? And what would you do to get antibiotics if you needed them?*

MAN: Entonces sí . . .
TRANSLATION: *So, yeah . . .*

MAN: Dependiendo como esté uno tan grave para que necesitar unos antibióticos, pero pues, no hay manera. Uno tiene que ir al doctor, y ellos te recetan los antibióticos porque, pues, si son muy fuertes y uno no puede conseguir lo que son, pues traen drogas que están fuertes, entonces para eso, sí, hay que ir al doctor.
TRANSLATION: *It depends on how sick you are in order to need antibiotics, but, well, there isn't a way. You have to go to the doctor, and they prescribe you the antibiotics because, well, they are very strong. And you can't find them, well, they contain drugs that are very strong. So for that, yeah, you have to go to the doctor.*

MAN: Sí porque hablamos pues de unas pastillitas que . . . es lo que nos podemos obtener porque ya antibiótico fuerte, ya no por falta de una receta.
TRANSLATION: *Yeah, because we're talking about some pills that . . . that is what we can obtain because you can't get a strong antibiotic without a prescription.*

MAN: Al menos . . . de que te enfermas, pero . . .
TRANSLATION: *Unless . . . you get sick, but . . .*

MAN: Sí, muchas veces, pues, no sabemos en realidad que tenemos. Y pues solo tomamos para a ver si le atinamos y el ibuprofen o Tylenol y eso, pues, es lo común. Hasta los doctores muchas veces no dan antibióticos. Lo único que dan y dicen, es una bacteria. Toma un Tylenol para la fiebre, y es lo único. Pero igualmente, ellos, los doctores en si no te recetan antibiótico a la primera.
TRANSLATION: *Yeah, a lot of times, well, we don't know what we have. And, well, we just take something to see if that helps and ibuprofen or Tylenol, stuff like that, well, that's what is most common. Doctors a lot of times don't even give you antibiotics. The only thing they say is that it's bacteria. Take some Tylenol for the fever, that's it. But the doctors won't give you antibiotics right away.*

MAN: De allí vamos aprendiendo pues que si va al doctor, regresa con una cajita de Tylenol y va más de vuelta dices tú, pues, ¿para qué voy? Si me van a dar eso, mejor me cajita allí en Walmart y ya, evita pierde tu día. Sí.
TRANSLATION: *From there, we're learning that, well, if you go to the doctor, you come back with a box of Tylenol, and then you've just run around saying, why did I go? If they're going to give me this, I might as well just go to Walmart, and that's it. That avoids wasting your time.*

MODERATOR: Y bueno, a veces, los antibióticos dejan de funcionar y no pueden matar ni controlar el crecimiento de bacteria que es la que se está tratando. Y eso es lo que se le llama la resistencia antibiótico, que pueda pasar en una persona o un animal. ¿Qué piensan sobre la resistencia antibiótico? ¿Algunas veces ustedes han tenido alguna infección o estado enfermo donde un antibiótico no le ha servido o ha sido como resistente?
TRANSLATION: *And, well, sometimes the antibiotics stop working, and they can't kill or control the growth of the bacteria that you're trying to treat. And that is what is called antibiotic resistance, which can happen in a person or in an animal. What do you think about antibiotic resistance? Have you ever had an infection or been sick where an antibiotic wasn't working, or the infection has been resistant?*

MAN: Hasta ahorita, no, yo no.
TRANSLATION: *Up to now, no, not me.*

MAN: Yo ahorita la gripa que traigo.
TRANSLATION: *The cold that I have now.*

MAN: Una semana, pues.
TRANSLATION: *A week.*

MAN: Sí, pero son pasajeras, pues. Pero de lo que usted está hablando es que te permanece en el cuerpo, que no lo pueda, o sea, que ya sea inmune a cualquier antibiótico.
TRANSLATION: *Yeah, but they pass through. But what you're talking about is that it stays in your body, that you can't, I mean, that it becomes immune to whatever antibiotic.*

MODERATOR: Mm-hmm. ¿A ninguna le ha pasado o que tenía algún familiar?
TRANSLATION: *Mm-hmm. That's never happened to any of you or a family member?*

MAN: No.

MODERATOR: ¿No, tampoco? ¿Y qué piensan sobre los riesgos, qué riesgos representa para ustedes u otras personas en su comunidad que exista resistencia antibiótico?
TRANSLATION: *Not you either? And what do you think about the risks, what risks does the existence of antibiotic resistance present to you or other people in your community?*

MAN: Es muy peligroso, ¿no? Si imagínate que si ya se inmune a una enfermedad muy grave, y luego, spread . . .
TRANSLATION: *It's very dangerous, right? I mean, image if an illness became immune, and then, spread . . .*

MODERATOR: Se transmite.
TRANSLATION: *It transmits.*

MAN: Se transmite por todas las demás personas que tú conoces, y así es como las enfermedades graves empiezan.
TRANSLATION: *It is transmitted to all the other people that you know, and that's how serious illnesses start.*

MAN: Pero, pues, gracias a dios no, ni la comunidad donde vivimos sabemos que haiga algo o aquí en el trabajo, los trabajadores, los compañeros de trabajo, no.
TRANSLATION: *But, well, thank God, not, not in the community we live in. We're not aware of anything like that here or at work, the workers, the coworkers, no.*

MODERATOR: ¿Y a los animales, por ejemplo, ¿qué piensan significa algún riesgo que haya resistencia antibiótica?
TRANSLATION: *And with the animals, for example, what are the risks if there is antibiotic resistance?*

MAN: Yo pienso que sí, porque igualmente, puede ser contagioso. Entonces si hay, pues, tenemos el, nosotros podemos, pues, deshacernos de ese animal. Podemos venderlo, podemos sacrificarlo dependiendo de que tan grave sea. Pero, pues . . . diferente.
TRANSLATION: *I think that, yeah, because it can be contagious. So if there is, well, we have, we can, well, get rid of that animal. We can sell it. We can euthanize it depending on how bad it is. But, well . . . different.*

MODERATOR: Claro.
TRANSLATION: *Of course.*

MAN: Sí. Pero, pues, para un racho, sí, es muy malo también. Hay enfermedades muy peligrosas y que puedan acabar con ranchos completos.
TRANSLATION: *Yeah. But, well, for a farm, yeah, it's very bad too. There are illnesses that are very dangerous and can wipe out entire farms.*

MODERATOR: Mm-hmm. Sí. ¿Y qué papel piensan ustedes que la granja lechera como que representa en la resistencia antibiótico, si tienen algún tipo de responsabilidad dentro de que exista resistencia antibiótica? Puede ser no tal vez, sí, o, no, lo que piensan.
TRANSLATION: *Mm-hmm. Yeah. And what role do you think dairy farms play in antibiotic resistance, if they have any kind of responsibility for antibiotic resistance existing? It could be yes or no, whatever you think.*

MAN: Pues, no entendemos a la pregunta, pero.
TRANSLATION: *Well, we don't understand the question.*

MODERATOR: Lo que, por ejemplo, si ustedes piensan que la granja lechera, no solo esta, sino en general, tiene alguna función dentro de que exista, o alguna tipa de responsabilidad que exista resistencia antibiótica.
TRANSLATION: *What, for example, if you guys think that dairy farms, not just this one, but in general, is responsible at all for the fact that antibiotic resistance exists.*

MAN: Pues, de hecho, sí. Pero lamentablemente, como detectaríamos nosotros que la enfermedad proviene de allí. O si el doctor lo sabe, pues, tampoco te lo va a decir que lo agarraste de allí. Entonces hay cosas muy, están para pensarse pues de como actuar, como te daría cuenta tú que la enfermedad que tiene la desarrollaste en esa área.
TRANSLATION: *Well, in fact, yes. But unfortunately, how would we tell what illness comes from there. Or if the doctor knows, but he's not going to be able to tell if you got it from there. So these are things to think about regarding how we act. How would you find out if the illness that you got developed in this area?*

MODERATOR: ¿Y por ejemplo, cuales son las políticas sobre el uso de los antibióticos acá en la granja, quienes pueden usarlos, o?
TRANSLATION: *And, for example, what are the policies about the use of antibiotics here on the farm, who can use them?*

MAN: Solamente los herdsmen, las personas que trabajan curando las vacas. Si maternidad u otra persona ve que una vaca está enferma, pues, nos la manda y nosotros decidimos que darle. Tenemos un protocolo que llevamos con el veterinario, entonces en caso de que tengamos dudas, nosotros preguntamos para que suministrarle o para . . .
TRANSLATION: *Only the herdsmen, the people that work with sick cows. If someone in maternity or another person sees a sick cow, well, they send them to us, and we decide what to give them. We have a protocol with the veterinarian. And so if we have any doubts, we can ask what to give or to . . .*

MODERATOR: O sea, ¿solamente hay personas autorizadas acá para aplicar antibiótico?
TRANSLATION: *So, only authorized people here can administer antibiotics?*

MAN: Sí. Sí.
TRANSLATION: *Yes. Yes.*

MODERATOR: ¿Y qué piensan sobre estas reglas, está bien, está mal?
TRANSLATION: *And what do you think about those rules, are they good, bad?*

[Simultaneous discussion]

MAN: Está bien.
TRANSLATION: *They are good.*

MAN: Está bien.
TRANSLATION: *They are good.*

MAN: Está bien.
TRANSLATION: They are good.

MAN: Está bien. Sí, porque a veces no falta quien ande jugando con algo más delicado.
TRANSLATION: *They are good. Yeah, because you don't need anyone playing around with something so delicate.*

MAN: Sí.
TRANSLATION: *Yeah.*

MAN: Algunos antibióticos también infectan la leche. Las hacen que tenga droga, y es muy importante que la persona que la administró sepa que esa vaca va a un lugar dónde no vaya a infectar a toda la manada e infectar 56,000 galones de leche que es algo muy malo.
TRANSLATION: *Some antibiotics infect the milk too. They make it so that it contains drugs. And it is very important that the person who is administering it knows that that cow needs to go to a place where it is not going to infect the rest of the herd and infect 56,000 gallons of milk, which is something that is very bad.*

MAN: Muy delicado, pues, para manejar eso.
TRANSLATION: *Managing that is a very delicate thing.*

MAN: Mm-hmm. Por eso mismo tenemos restricciones porque podemos afectar mucho. Si nosotros mandamos un tanque contaminado con antibiótico, es un problema muy grande. Entonces, no, lo tenemos muy claro eso de que no se suministra antibiótico que la tenemos controlado. Entonces nada más personas autorizadas.
TRANSLATION: *Mm-hmm. For that reason, we have restrictions because it could affect a lot. If we send milk to the tank that is contaminated with antibiotics, it's a huge problem. So, no, it is very clear to us that we don't give antibiotics, and that we keep them controlled. So only authorized people can do it.*

MODERATOR: Perfecto. Estos son todas las preguntas que tengo. No sé si tienen alguna duda o si le gustaría compartir algo más.
TRANSLATION: *Perfect. Those are all the questions I have. I don't know if you have any questions or if you would like to share something more.*

MAN: Por mi, no queda mucho.
TRANSLATION: *On my part, there's not much more.*

MODERATOR: Sí, como le había dicho, es solamente relacionado con su trabajo y la rutina que hacen y todo eso. Así que muchas gracias por su participación. Fue muy útil.
TRANSLATION: *Yes, like I said, it's just related to your job and your routine and all that. So thank you very much for your participation. It was very useful.*

Focus Group 11, Farm 8
Recording in Spanish

MODERATOR: Bueno, primero que nada, un poquito de información sobre ustedes. Entonces, participe un poquito más de persona a persona, y luego, el resto de la discusión va a ser más de todo el grupo, ¿entienden? Entonces, primero que nada, ¿cuántos años tienen y cómo describiría tu raza, si hispánico, latino, u otro grupo. Y si pueden ir por uno simplemente diciendo esa información rápido. No tiene que decir el nombre. Puede si quiera, pero . . .
TRANSLATION: *Well, first off, a little information about you guys. So participate a little more person to person, and then the rest of the discussion is going to be more of the whole group, does that makes sense? So, first off, how old are you and how would you describe your race, if Hispanic, Latino, or another group? And if you can go one by one simply saying that information quickly. You don't have to say your name. You can if you want, but . . .*

MAN: Yo soy primero. Tengo 42 años y soy hispano.
TRANSLATION: *I'm first. I am 42 years old, and I am Hispanic.*

MAN: Cuarenta años, hispano.
TRANSLATION: *Forty years old, Hispanic.*

MAN: Veintiséis años, hispano.
TRANSLATION: *Twenty-six years old, Hispanic.*

MAN: Treinta años, hispano.
TRANSLATION: *Thirty years old, Hispanic.*

MAN: Veintisiete años, hispano.
TRANSLATION: *Twenty-six years old, Hispanic.*

MAN: Veintinueve años, hispano.
TRANSLATION: *Twenty-nine years old, Hispanic.*

WOMAN: Treinta y tres, hispana.
TRANSLATION: *Thirty-three, Hispanic.*

MAN: Estás mentirosa.
TRANSLATION: *Liar.*

MAN: Veintiocho, hispano.
TRANSLATION: *Twenty-eight, Hispanic.*

MODERATOR: Está bien. Perfecto. Yo tengo 27. No sé . . . ¿cuánto tiempo han estado trabajando aquí y qué hacen más o menos en un día típico?
TRANSLATION: *Okay. Perfect. I am 27. I don't know . . . how long have you been working here, and what do you do more or less in a typical day?*

MAN: Muchas cosas.
TRANSLATION: *Many things.*

WOMAN: No, tú empiezas.
TRANSLATION: *No, you start.*

MAN: O sea, en el orden.
TRANSLATION: *I mean, in order.*

MAN: No, me voy a tardar.
TRANSLATION: *No, I'm going to take a while.*

MODERATOR: No tiene que ser tan exhaustivo. O sea, no tiene que ir cosa por cosa, pero lo que es en general.
TRANSLATION: *It doesn't have to be so exhaustive. I mean, you don't have to go thing by thing, but what it is in general.*

MAN: ¿Todo lo que hago?
TRANSLATION: *Everything I do?*

MODERATOR: No tiene que ser tan, tan detallado, pero, en términos globales, ¿qué hace aquí en la granja? ¿Qué es, más que nada, trabaja con las vacas en lechera, no sé si, esto o lo otro.
TRANSLATION: *It doesn't have to be so, so detailed, but, in global terms, what you do here on the farm? What is, mostly, working with the dairy cows, I don't know if, this or that?*

MAN: Yo trabajo con medicina por las vacas enfermas, maternidad, inseminación, checar de preñadas 32 días hasta 290 días. O sea, todo lo que es, por te dije que es mucho.
TRANSLATION: *I work with medicine for sick cows, maternity, insemination, checking pregnant cows 32 days until 290 days. I mean, everything that is, that's why I told you it's a lot.*

[Simultaneous discussion]

WOMAN: Y ahora, ¿cuánto llevas trabajando?
TRANSLATION: *And now, how long have you been working?*

MAN: Catorce años, casi 15.
TRANSLATION: *Fourteen years, almost 15.*

MAN: Dos años trabajando acá. Ordeñamos y ya vamos a puchar comida, que es acercar la comida a las vacas, maternidad y, principalmente.
TRANSLATION: *Two years working here. We milk, and now we're pushing food, which is moving the food closer to the cows, maternity and, mainly.*

MAN: Dos años trabajando aquí, ordeñar, puchar vacas, comida y maternidad, y de vez en cuando, separamos vacas.
TRANSLATION: *Two years working here, milking, pushing cows, food, and maternity, and once in a while, we separate cows.*

MAN: Cuando mixtean(?) vacas.
TRANSLATION: *When the cows get mixed up.*

MAN: Las que mezclan.
TRANSLATION: *Those that get mixed up.*

MAN: Yo llevo seis meses. Las actividades que hago, ordeño, nada más.

TRANSLATION: *I've been here six months. The activities I do, I milk, nothing else.*

[Simultaneous discussion]

MODERATOR: ¿Falta uno?
TRANSLATION: *Anyone left?*

MAN: A veces . . .
TRANSLATION: *Sometimes . . .*

MAN: Le pondrá música.
TRANSLATION: *He'll put on music.*

WOMAN: El DJ.
TRANSLATION: *The DJ.*

MODERATOR: El DJ, está bien.
TRANSLATION: *The DJ, okay.*

MAN: Estoy por cumplir cuatro meses y, por el momento, solo ordeño.
TRANSLATION: *I've almost been here four months and, for now, I just milk.*

MAN: Está pollito todavía.
TRANSLATION: *He's still new.*

MAN: Estoy en la junta.
TRANSLATION: *I'm in the meeting(?).*

MAN: Llevo año y medio, ordeño, pucho vacas y pucho comida.
TRANSLATION: *I've been here a year and a half, I milk, push cows, and push food.*

WOMAN: Ocho años, y cuido a los becerros.
TRANSLATION: *Eight years, and I take care of the calves.*

MAN: Un año, cuatro meses, alimentación y cuidado de becerros.
TRANSLATION: *One year, four months, nutrition and calf care.*

[Simultaneous discussion]

MAN: No estoy el más viejo . . .
TRANSLATION: *I'm not the oldest one . . .*

MODERATOR: Y como oíste, les iba a preguntar también juntos, pero si ha trabajado en otro tipo de granja haciendo esto, o sea, ¿cuánto tiempo ya están haciendo esto tipo de actividades? ¿Todo el tiempo que uno ha estado en esta granja, o también han trabajado en otras distintas?
TRANSLATION: *And as you heard, I was going to ask you some questions together, but if you have worked on another type of farm doing this, or, I mean, how long have you been doing these types of activities? Have you been at this farm the whole time, or have you also worked in different ones?*

MAN: Yo solo en esta granja, y llevo como siete años haciéndolo.

TRANSLATION: *I've just been at this farm, and I've been doing it for like seven years.*

MAN: Aquí en Estados Unidos, tres meses en una granja de Minnesota.
TRANSLATION: *Here in the United States, three months on a farm in Minnesota.*

MAN: Yo he trabajado en dos otros ranchos.
TRANSLATION: *I've worked on two other farms.*

MODERATOR: ¿Cuánto tiempo, más o menos?
TRANSLATION: *How long, more or less?*

MAN: En uno, seis meses, y en el otro, cuatro años.
TRANSLATION: *On one, six months, and on the other, four years.*

MAN: Yo solo, primera granja que es acá . . .
TRANSLATION: *I just, first farm has been here . . .*

MAN: Igual, primer granja.
TRANSLATION: *Same, first farm.*

WOMAN: Otro rancho, cuatro años.
TRANSLATION: *Another farm, four years.*

MAN: Primer granja . . .
TRANSLATION: *First farm . . .*

MAN: Y ya.
TRANSLATION: *And that's it.*

MAN: . . . y ya.
TRANSLATION: *. . . and that's it.*

[Simultaneous discussion]

MODERATOR: Okay. ¿Alguna vez que están trabajando, se sienten apresurados en para terminar el trabajo rápido, para terminar a tiempo? ¿Es algo que sucede? ¿Sí?
TRANSLATION: *Okay. Have you ever felt pressured to finish your work quickly in order to finish on time? Is that something that happens? Yes?*

MAN: Hay diferentes áreas. A lo mejor, ellos, sí, sienten un poco más presionados. Lo digo porque ellos son los que ordeñan y, pues, que ordeña, tiene que estar . . .
TRANSLATION: *There are different areas. Maybe, they, yes, feel a little more rushed. I'm saying that because they are the ones that milk and, well, those who milk, they have to be . . .*

MODERATOR: ¿Tiene que hacerlo rápido?
TRANSLATION: *You have to do it quickly?*

MAN: Y puchando, trabajando afuera, contigo, trabajo con vacas . . . afuera.
TRANSLATION: *And pushing, working outside, with you, I work with cows . . . outside.*

MODERATOR: Muy bien. O sea, que . . . el ordeño es la actividad que tiene, la gente se siente más presionados para hacerlo con velocidad?
TRANSLATION: *Very good. I mean . . . milking is the activity that has, the people feel more pressured to do it quickly?*

MAN: Uh-huh.

MODERATOR: Está bien. Bueno, la última pregunta, ¿cuál es la parte favorita del trabajo de ustedes?
TRANSLATION: *Okay. Well, the last question, what is your favorite part of your job?*

MAN: Lonche.
TRANSLATION: *Lunch.*

MAN: Lonche.
TRANSLATION: *Lunch.*

MAN: Lonche, dice.
TRANSLATION: *Lunch, he says.*

MAN: Para ti, sí.
TRANSLATION: *For you, yeah.*

MAN: Pues, a mi, todo.
TRANSLATION: *Well, for me, everything.*

MODERATOR: ¿Todo?
TRANSLATION: *Everything?*

MAN: Si no, no lo hiciera el trabajo.
TRANSLATION: *If not, I wouldn't do the job.*

MODERATOR: Claro. Okay. Uh-huh. Bueno, ahorita el resto de las preguntas son cómo está la granja organizada y cómo es operada. En general, reciben muchas direcciones de sobre cómo hacer el trabajo? ¿Qué tipo de direcciones normalmente se haga aquí típicamente? O sea, ¿esta granja es una granja donde hay mucha, están constantemente dando direcciones, dando la presión, o tienen más como independencia de cómo trabajan?
TRANSLATION: *Of course. Okay. Uh-huh. Well, now the rest of the questions are how the farm is organized and how it's operated. In general, do you receive many directions about how to do the job? What type of directions are typically given here? I mean, is this farm a farm where there is a lot of, they are constantly giving directions, pressuring, or do you guys have more like independence with how you work?*

MAN: No dan, dan, digamos que nos dan los protocolos de trabajo, y de allí, están revisando constantemente que no hay desviaciones de los protocolos.
TRANSLATION: *They don't give, they give, let's say they give us work protocols, and from there, they are reviewing constantly that there aren't deviations from the protocols.*

MODERATOR: Está bien. ¿Y cuánta gente consideraríamos que son gerente or jefes en la granja?
TRANSLATION: *Okay. And how many people would we consider as managers or bosses on the farm?*

MAN: Uno.
TRANSLATION: *One.*

[Simultaneous discussion]

MODERATOR: Oh. Okay. ¿Y hay buena comunicación con el jefe?
TRANSLATION: *Oh. Okay. And is there good communication with the boss?*

MAN: Sí, habla español.
TRANSLATION: *Yes, he speaks Spanish.*

MAN: Sí.
TRANSLATION: *Yeah.*

MODERATOR: Habla español. Así está bien.
TRANSLATION: *He speaks Spanish. That's good.*

MAN: Eso ayuda.
TRANSLATION: *That helps.*

MODERATOR: Perfecto. Okay. Bueno, entonces, estas preguntas más o menos relacionan si tienen a alguien que ustedes le pueden hacer preguntas y qué tan cómodo se sienten haciéndole preguntas al jefe. Si allí, ¿cómo . . . en ese sentido?
TRANSLATION: *Perfect. Okay. Well, then, these questions more or less relate to if you guys have someone that you can ask questions and how comfortable you feel asking the boss questions. If there, how . . . in that sense?*

MAN: Sí . . .
TRANSLATION: *Yeah . . .*

MAN: Sí, apertura de respuestas.
TRANSLATION: *Yes, open with answers.*

MODERATOR: Perfecto. ¿Y gente sí pueden hablar en español con él?
TRANSLATION: *Perfect. And people can speak Spanish with him?*

MAN: Sí.
TRANSLATION: *Yes.*

MAN: Sí.
TRANSLATION: *Yes.*

MODERATOR: Okay. Y esta pregunta que es un poco más individual, si están cómodos hablando en inglés con, hay gente en la granja que no hable español?
TRANSLATION: *Okay. And this question is a bit more individual, if you feel comfortable speaking in English with, are there people on the farm that don't speak Spanish?*

MAN: Sí.
TRANSLATION: *Yes.*

MAN: Sí.
TRANSLATION: *Yes.*

MODERATOR: ¿Y ustedes tienen que comunicarse con ellos diariamente?
TRANSLATION: *And do you guys have to communicate with them daily?*

MAN: Pues, no es necesario, pero . . .
TRANSLATION: *Well, it's not necessary, but . . .*

MODERATOR: Si toca hacerlo.
TRANSLATION: *If you need to.*

[Simultaneous discussion]

MAN: Sí . . .
TRANSLATION: Yeah . . .

MAN: Depende del trabajo que hagamos unos.
TRANSLATION: *It depends on the work that some of us do.*

MAN: Algunas se interactúan más con otras.
TRANSLATION: *Some interact more with others.*

MODERATOR: ¿Con la gente que no habla español? ¿Como, por ejemplo, qué área?
TRANSLATION: *With the people that don't speak Spanish? Like, for example, what area?*

WOMAN: Nosotros, con los becerros. Cuando tenemos que limpiar algo que es para la comida, pero . . .
TRANSLATION: *Us, with the calves. When we have to clean something that is for the food, but . . .*

MAN: Pero ella es nuestra interprete.
TRANSLATION: *But she is our interpreter.*

MAN: Interprete, dice. Y sí ¿verdad?
TRANSLATION: *Interpreter, he says. And, yeah, right?*

MAN: Es nuestra interprete.
TRANSLATION: *She is our interpreter.*

MAN: O sea, si nos anda engañado y más dice la mitad de lo que están diciendo a ella.
TRANSLATION: *I mean, if she's tricking us and just telling us half of what they're telling her.*

MAN: Se amortigua.
TRANSLATION: *She's a buffer(?).*

MODERATOR: Okay. Y con la gente de la granja que hablan español, ¿sí sienten cómodos hablando inglés con ellos, con la gente que tienen que hablar inglés?
TRANSLATION: *Okay. And with the people on the farm that speak Spanish, do you feel comfortable speaking English with them, with the people that have to speak English?*

[Simultaneous discussion]

MAN: Es que depende también, digamos, la posición de uno como empleado. Digamos aquí es mi compañero, Julio, él, o sea, tiene más compromisos de hablar inglés. ¿Por qué? Porque hace otras actividades. Y nosotros que somos de ordeña, o sea, no tenemos que hablar con las personas que hablen inglés. ¿Por qué? Porque yo, el que es nuestro jefe, ya habla español.
TRANSLATION: *That also depends, let's say, your position as an employee. Let's say here is my partner, Julio, he, I mean, has more obligations to speak English. Why? Because he does other activities. And those of us that milk, I mean, we don't have to talk with the people that speak English. Why? Because I, the one that's our boss, he already speaks Spanish.*

MODERATOR: Claro, depende como del supervisor de . . .
TRANSLATION: *Of course, depends like on the supervisor of . . .*

MAN: Uh-huh, depende del supervisor y el tipo de actividades que se hacen.
TRANSLATION: *Uh-huh, it depends on the supervisor and the type of activities that are done.*

MODERATOR: Ya, entonces, hay algunos supervisores que sí pueden hablar español, otros que no hablen español, entonces eso depende completamente.
TRANSLATION: *Yeah, so there are some supervisors that can speak Spanish, others that don't speak Spanish, so that really depends.*

MAN: Mm-hmm, sí.
TRANSLATION: *Mm-hmm, yes.*

MODERATOR: Ya veo. No sé, ¿algún otro tipo de obstáculo para hablar con o comunicarse con su jefe o gerente o otros compañeros de trabajo? ¿No?
TRANSLATION: *I see. I don't know, any other type of obstacle talking with or communicating with your boss or manager or other colleagues? No?*

MAN: No.

MAN: No.

MAN: No.

MODERATOR: Okay. Bueno, estas preguntas son un poquito repetitivas, pero, si trabaja con alguien que no habla la misma lengua que ti, y ¿cómo te comunicas con estos compañeros y . . . se sienten que pueden hablar con ellos? Creo más o menos ya respondimos a esta pregunta.
TRANSLATION: *Okay. Well, these questions are a little repetitive, but, if you work with someone who doesn't speak the same language as you, and, how do you communicate with these colleagues and . . . you feel that you can speak with them? I think, more or less, we already answered this question.*

MAN: Sí.
TRANSLATION: *Yes.*

MODERATOR: Depende, más que nada, entonces, del área especifica y del supervisor.
TRANSLATION: *It depends, more than anything, then, on the specific area and on the supervisor.*

MAN: Uh-huh.

MAN: Mm-hmm.

MODERATOR: A ver, otra pregunta. ¿Dónde, de quién recibe más información sobre lo que está sucediendo en la granja?
TRANSLATION: *Let's see, another question. Where, who do you receive more information from about what is happening on the farm?*

MAN: El mánager, no?
TRANSLATION: *The manager, right?*

MODERATOR: ¿El mánager?
TRANSLATION: *The manager?*

MAN: Sí.
TRANSLATION: *Yes.*

MODERATOR: El mánager es el que . . .
TRANSLATION: *The manager is the one that . . .*

MAN: Sí.
TRANSLATION: *Yes.*

MAN: Sí.
TRANSLATION: *Yes.*

[Simultaneous discussion, banter]

MODERATOR: Okay. Estas preguntas son sobre la salud de ustedes y la salud de las vacas en la granja. Primero, nos gustaría que hagan unas preguntas . . . preguntas sobre antibióticos. Solo para aclarar, los antibióticos son medicinas que utilizan los médicos para ayudar a una persona que tiene una infección con bacterias para mejorarse. Los veterinarios usan estas medicinas también para ayudar los animales con infecciones para mejorarse. Son preguntas para los que están familiarizados con los antibióticos. ¿Están familiarizados con los antibióticos? ¿Qué piensan de los antibióticos?
TRANSLATION: *Okay. These questions are about your health and the health of the cows on the farm. First, we would like to ask some questions . . . questions about antibiotics. Just to clarify, antibiotics are medicines that doctors use to help someone with a bacterial infection get better. Veterinarians use these medicines also to help animals with infections get better. These are questions for those that are familiar with antibiotics. Are you guys familiar with antibiotics? What do you guys think about antibiotics?*

MAN: Tú eres la que ocupas más ahorita.
TRANSLATION: *You're the one that deals with that more right now.*

WOMAN: Uh-huh, a mi no me gusta usar tanto antibiótico.
TRANSLATION: *Uh-huh, I don't like to use so much antibiotics.*

MODERATOR: ¿Para ti, personal, o?

TRANSLATION: *For you, personally, or?*

WOMAN: Personal. Para los animales, mm, tampoco.
TRANSLATION: *Personally. For the animals, mm, either.*

MODERATOR: Okay. ¿Y por qué no?
TRANSLATION: *Okay. And why not?*

WOMAN: Porque estás matando la buena bacteria del animal, al menos de que esté grave o está poniendo mal, no me gusta usarlo.
TRANSLATION: *Because you're killing the good bacteria of the animal, unless it's serious or it's getting sick, I don't like to use it.*

MODERATOR: Claro, pero, o sea, ¿por qué no te gusta usarlo en ti, también porque sientes que matan a la buena bacteria?
TRANSLATION: *Of course, but, I mean, why do you not like to use it for yourself, also because you feel it kills the good bacteria?*

WOMAN: No, simplemente no me gusta.
TRANSLATION: *No, I just don't like it.*

MAN: Y otra para, también no crear resistencia, ¿no?
TRANSLATION: *And another for, also to not create resistance, right?*

WOMAN: Mm-hmm.

MAN: Mm-hmm, en esta granja, hay tendencia no usar antibióticos en general, contra mastitis, por ejemplo.
TRANSLATION: *Mm-hmm, on this farm, there is a tendency not to use antibiotics in general, against mastitis, for example.*

MODERATOR: Okay. ¿Y eso es diferente en otras granjas?
TRANSLATION: *Okay. And is that different than other farms?*

MAN: Sí.
TRANSLATION: *Yeah.*

MODERATOR: ¿En otras granjas, sí lo utilizan más?
TRANSLATION: *On other farms, they do use it more?*

MAN: Sí, de primero.
TRANSLATION: *Yes, first thing.*

MAN: Yo lo ocupé un tiempo nada más en vacas con mastitis.
TRANSLATION: *I used it for a time just in cows with mastitis.*

MODERATOR: Sí, ¿en esta granja?
TRANSLATION: *Yeah, on this farm?*

MAN: En esta granja, pero ya después, tiene como cinco o seis años que ya le dije, no, sabes qué, pues, no. Y lo quitaron.
TRANSLATION: *On this farm, but then it's been like five or six years already that I told him, no, you know what, no. And they took it away.*

MODERATOR: Oh. Okay. ¿Recién recibieron antibióticos ustedes, alguna vez el médico le ha explicado cómo tomarlos? Si le ha dado instrucciones, la dosis . . .
TRANSLATION: *Oh. Okay. Did you recently receive antibiotics, has the doctor ever explained to you guys how to take them? If you've been given instructions, the dose . . .*

WOMAN: Sí, mm-hmm.
TRANSLATION: *Yes, mm-hmm.*

MAN: Sí.
TRANSLATION: *Yes.*

MODERATOR: Claro. ¿Qué tan fácil pueden obtener los antibióticos si piensan que los necesitan?
TRANSLATION: *Of course. How easily can you obtain antibiotics if you think you need them?*

MAN: Acá, casi no.
TRANSLATION: *Here, not much.*

MAN: No.

MAN: Necesita una receta, con receta médica.
TRANSLATION: *You need a prescription, with a medical prescription.*

MAN: Una receta médica. Y sí, te va a costar.
TRANSLATION: *A medical prescription. And, yeah, it's going to cost you.*

MAN: Un buen dinero.
TRANSLATION: *A good chunk of money.*

[Simultaneous discussion]

MODERATOR: ¿Por lo costoso?
TRANSLATION: *Because it's expensive?*

MAN: Por lo costoso.
TRANSLATION: *Because it's expensive.*

MODERATOR: ¿Del antibiótico o obtener la receta?
TRANSLATION: *The antibiotic or getting the prescription?*

WOMAN: De las dos cosas.
TRANSLATION: *Both things.*

MODERATOR: De las dos cosas.
TRANSLATION: *Both things.*

MAN: Por la receta, te vas a la clínica free, y te la dan, sea antibiótico que sea, pero la medicina también está cara.
TRANSLATION: *For the prescription, you go to the free clinic, and they give you, whatever antibiotic it is, but the medicine is also expensive.*

WOMAN: De aquí, quieres una cita . . .
TRANSLATION: *From here, if you want an appointment . . .*

MAN: Ya estaba, ya te moriste.
TRANSLATION: *You already died.*

MAN: Sí.
TRANSLATION: *Yeah.*

MAN: Se muere uno, positivo esa infección ya sí es más grande.
TRANSLATION: *You die, positive that infection is bigger.*

MODERATOR: O sea, del momento que uno se enferma hasta que lo obtiene . . .
TRANSLATION: *I mean, from the moment you become sick until you get . . .*

MAN: Es lo malo de aquí también, si estás en enfermo y hablas, o necesitas una cita. Oye, señora, me estoy muriendo, ¿cómo quiere que haga una cita, quiere que me aguante dentro de cuántos días? ¿Qué es lo que tiene? Tantas preguntas, pues, ya tu cuerpo ya está . . .
TRANSLATION: *That's the bad thing here also, if you are sick and you call, oh, you need an appointment. Listen, ma'am, I'm dying, how do you want me to make an appointment, you want me to deal with it for how many days? What's wrong with you? So many questions, you know, your body is already . . .*

MODERATOR: Está bien. Entonces, no es tan fácil obtener algunos antibióticos por, o sea, por pasar por el proceso . . .
TRANSLATION: *Okay. So it's not so easy to get some antibiotics through, I mean, going through the process.*

MAN: Mm-hmm. Sí, tienes que pasar por un proceso como de dos semanas.
TRANSLATION: *Mm-hmm. You have to go through like a two-week process.*

MODERATOR: ¿Dos semanas, más o menos?
TRANSLATION: *Two weeks, more or less?*

MAN: Cinco días o seis, no, que te dan la cita.
TRANSLATION: *Five days or six, right, they'll give you an appointment.*

MODERATOR: Okay. Próxima pregunta, ¿de dónde obtienen antibióticos usualmente?
TRANSLATION: *Okay. Next question, where do you usually get antibiotics?*

MAN: Para el rancho, del veterinario.
TRANSLATION: *For the farm, from the veterinarian.*

MODERATOR: Del veterinario.
TRANSLATION: *From the veterinarian.*

MAN: Veterinario, sí.
TRANSLATION: *Veterinarian, yeah.*

WOMAN: Es el que autoriza cuánto antibiótico . . .
TRANSLATION: *He's the one that authorizes how much antibiotic . . .*

MAN: El que autoriza la compra, mm-hmm.
TRANSLATION: *He authorizes the purchase, mm-hmm.*

MODERATOR: Ya, ¿y para uso personal?
TRANSLATION: *Okay, and for personal use?*

WOMAN: Doctor.

MAN: Doctor.

MODERATOR: Doctor, la farmacia.
TRANSLATION: *Doctor, the pharmacy.*

MAN: Tienda mexicana, más barato.
TRANSLATION: *Mexican store, cheaper.*

MODERATOR: Sí, los importan, ¿no?
TRANSLATION: *Yeah, they import them, right?*

MAN: Sí.
TRANSLATION: *Yes.*

MAN: Ya no diga más.
TRANSLATION: *Don't say anything more.*

MODERATOR: No, igual esto es como, va a ser completamente confidencial. ¿Algunos han tenido alguna infección, han tomado antibióticos y los antibióticos no les hicieron sentir mejor? En el pasado.
TRANSLATION: *No, just the same, this is like, it's going to be completely confidential. Have any of you had an infection, have you taken antibiotics and the antibiotics did not make you feel better? In the past.*

[Simultaneous discussion]

MAN: Que he sentido mejor, sí.
TRANSLATION: *That I've felt better, yes.*

MODERATOR: No, si ha pasado el contrario, que sienten más enfermo, que tomar el antibiótico y sientes que no mejoraste con el antibiótico.
TRANSLATION: *No, if the contrary has happened, that you've felt sicker, that taking the antibiotic and you feel that you didn't get better with the antibiotic.*

MAN: Uh-huh.

MODERATOR: ¿También ha pasado?

TRANSLATION: *That has also happened?*

MAN: Sí.
TRANSLATION: *Yes.*

[Simultaneous discussion]

MODERATOR: Okay. Algunas veces los antibióticos paran de trabajar y no pueden matar o controlar el crecimiento de la bacteria en una persona o animal. De allí, esto, el antibiótico puede que ya no funciona para tratar la infección de esa persona o ese animal. Este nombre se llama, esto tiene nombre, resistencia antibiótica. Otra manera de decir esto, es que cuando la bacteria empieza a resistir el efecto de la medicina y continúa creciendo, incluso cuando la gente se toma la medicina correctamente. La resistencia antibiótica puede afectar a los humanos y a los animales también. ¿Están familiarizados con la resistencia antibiótica? Alguien aquí mencionó el problema de la resistencia, que no le gusta tomar antibióticos por este motivo, ¿no?
TRANSLATION: *Okay. Sometimes antibiotics stop working and they can't kill or control the growth of the bacteria in a person or animal. From there, this, it might be that the antibiotic doesn't work anymore to treat the infection of that person or that animal. This name is called, this has a name, antibiotic resistance. Another way of saying this is when the bacteria starts to resist the effect of the medicine and it continues growing, even when people take the medicine correctly. Antibiotic resistance can affect humans and animals as well. Are you guys familiar with antibiotic resistance? Someone here mentioned the problem of resistance, that they don't like to take antibiotics for that reason, right?*

MAN: Bueno, la pregunta, ¿cuál es?
TRANSLATION: *Well, the question, what is it?*

MODERATOR: A los animales, pero en las personas también . . .
TRANSLATION: *In animals, but in people as well . . .*

MAN: Mm-hmm, mm-hmm.

MODERATOR: Necesitamos estar más familiarizados con la resistencia antibiótica, ¿no? Sí. Entonces la pregunta, en tu vida cotidiana, una vez has escuchado o discutió del antibiótico, resistencia antibiótica? Yo creo que hasta ya hemos hablado, lo discutimos.
TRANSLATION: *We need to be more familiar with antibiotic resistance, right? Yeah. So the question, in your daily life, have you ever heard or discussed antibiotic resistance? I believe that, by now, we've talked, we discussed it.*

MAN: Sí.
TRANSLATION: *Yes.*

MODERATOR: ¿Alguna vez tuvo un ser querido que ha tenido una infección de una bacteria resistente a los antibióticos?
TRANSLATION: *Did you ever have a loved one that has had an infection from a bacterium that was resistant to antibiotics?*

MAN: No.

MAN: No.

MAN: Hmm-mm.

MAN: Bueno, diagnosticada, no. O sea, nunca te han dicho, tienes una bacteria resistente a este antibiótico. O sea, es como una conclusión que hace cada uno.
TRANSLATION: *Well, diagnosed, no. I mean, they've never told you, you have a bacterium resistant to this antibiotic. I mean, it's like a conclusion that you come to.*

MODERATOR: Ya, porque de tomar el antibiótico . . .
TRANSLATION: *Okay, because taking the antibiotic . . .*

MAN: Y no te hace nada, no te hace efecto. Y te moriste.
TRANSLATION: *And it doesn't do anything to you, it doesn't take effect. And you died.*

MODERATOR: Okay. Piensan que ustedes o algún familiar está en riesgo de contraer una infección resistente a los antibióticos?
TRANSLATION: *Okay. Do you guys think you or some family member is at risk of contracting an infection that's resistant to antibiotics?*

WOMAN: No.

MAN: No.

MODERATOR: No. Okay. ¿Piensan que lecherías como esta son, están en riesgo de ser afectadas por esa resistencia antibiótica?
TRANSLATION: *No. Okay. Do you think that dairy farms like this are at risk of being affected by that antibiotic resistance?*

MAN: No, como dicen mis compañeros, que no lo utilizan.
TRANSLATION: *No, as my colleagues say, they don't use it.*

WOMAN: Sí y no.
TRANSLATION: *Yes and no.*

MAN: Muy poco, ¿no?
TRANSLATION: *Very little, right?*

WOMAN: Porque sí puede pasar.
TRANSLATION: *Because it can happen.*

MODERATOR: ¿Sí puede pasar?
TRANSLATION: *It can happen?*

WOMAN: Sí, puede pasar.
TRANSLATION: *Yeah, it can happen.*

MODERATOR: ¿Pero, a la vez, ustedes dicen que esta granja ha tratado de usar lo menos antibiótico?
TRANSLATION: *But, at the same time, you guys say that this farm has tried to use less antibiotic?*

WOMAN: Sí, pero, sí, puede pasar.
TRANSLATION: *Yes, but, yeah, it can happen.*

MAN: Pero en unos 20 años, no ahorita, ¿sí?
TRANSLATION: *But in some 20 years, not right now, right?*

WOMAN: No, porque si está creciendo una bacteria, tenemos que cambiar el medicamento . . .
TRANSLATION: *No, because, if a bacterium is growing, we have to change the medicine . . .*

MAN: Oh, sí, tienes que cambiar el medicamento.
TRANSLATION: *Oh, yeah, you have to change the medicine.*

WOMAN: . . . o combinarlo con algo más para que mate . . .
TRANSLATION: *. . . or combine it with something else in order to kill . . .*

MAN: La bacteria.
TRANSLATION: *The bacterium.*

MODERATOR: ¿Piensan que una lechería como esta tiene algún papel en crear resistencia a los antibióticos? ¿Por qué piensan que sí o que no?
TRANSLATION: *Do you guys think that a dairy farm like this one has some role in creating resistance to antibiotics? Why do you think yes or no?*

MAN: ¿En general, las lechería, o esta?
TRANSLATION: *In general, dairy farms, or this one?*

MODERATOR: En general, las lecherías.
TRANSLATION: *In general, dairy farms.*

MAN: Sí, por su uso indiscriminado, ¿no?
TRANSLATION: *Yes, because of indiscriminate use, right?*

MODERATOR: Ya, ¿y piensan que si se ponen reglas estrictas y protocolos sobre los antibióticos en una granja, se beneficia a los animales y a la comunidad?
TRANSLATION: *Okay, and do you guys think that, if strict rules and protocols about antibiotics on a farm are put in place, the animals and community benefit?*

WOMAN: Mm-hmm, sí.
TRANSLATION: *Mm-hmm, yes.*

MAN: Sí.
TRANSLATION: *Yes.*

MODERATOR: Okay. Bueno . . . hemos discutido un poco, pero la pregunta que viene es, ¿cuáles son las reglas actuales en la granja sobre el uso de antibióticos? ¿Podrían explicarme cómo son el uso de los antibióticos actualmente en la granja y cómo ustedes se enteran si llega algún cambio en las reglas o con el protocolo del uso de antibióticos en esta granja?
TRANSLATION: *Okay. Well . . . we've discussed a bit, but the next question is, what are the current rules on the farm about the use of antibiotics? Could you explain to me how the use of antibiotics currently on the farm and how you guys find out if there is some change in the rules or with the protocol for use of antibiotics on this farm?*

WOMAN: Tenemos un protocolo de, como si una becerra se enferma o tiene temperatura o está caída, que no se levanta, pero hay protocolo de que tienes que hacer tantos puntos antes de curar este animal. Esto es provenido, no lo da el veterinario. Y si, en ese puntaje, no se cura el animal hasta que se ponga más mal inclusive.
TRANSLATION: *We have a protocol, like if a calf is sick or has a temperature or falls, it can't get up, but there is a protocol that you have to do so many points before curing this animal. This comes from, the veterinarian doesn't give it. And if, with that scoring system, the animal doesn't get better or even becomes worse.*

MODERATOR: Mm-hmm. ¿Entonces allí sí es cuando se usa el antibiótico, y si no, no antes?
TRANSLATION: *Mm-hmm. Then that is when you do use the antibiotic, and if not, not before.*

WOMAN: Mm-hmm.

MODERATOR: Y si llegar un cambio en el protocolo . . . alguien decide alterar esos puntos, ¿cómo ustedes se enterarían?
TRANSLATION: *And if there is a change in the protocol . . . someone decides to alter those points, how do you guys find out?*

WOMAN: Eso solamente lo hace el veterinario. El veterinario nos dice según el área donde ese problema.
TRANSLATION: *Only the veterinarian does that. The veterinarian tells us according to the area where the problem is.*

MODERATOR: O sea, ¿el veterinario se comunica directamente con ustedes?
TRANSLATION: *I mean, does the veterinarian communicate with you guys directly?*

WOMAN: Sí, mm-hmm.
TRANSLATION: *Yes, mm-hmm.*

MODERATOR: ¿No está, de repente, escrito?
TRANSLATION: *It's not written ever?*

WOMAN: No.

MODERATOR: Okay.

MAN: Pero si los rotan, ¿verdad? O sea, van como rotando los antibióticos, ¿o no?
TRANSLATION: *But they rotate them, right? I mean, they are always rotating the antibiotics, right?*

WOMAN: No, sí, tenemos un protocolo si uno no te funciona, va a usar el siguiente.
TRANSLATION: *No, yes, we have a protocol if one isn't working for you, he'll use the next one.*

MAN: ¿Pero cuántos días usas uno y cuántos días vas a usar el otro? . . .
TRANSLATION: *But how many days do you use one and how many days do you use the other? . . .*

WOMAN: . . . hasta tres días, y si no miras que el becerro esté mejorando, cambia, no tienes que esperar a terminar seis días.
TRANSLATION: *. . . up to three days, and if you don't see that the calf is getting better, you change, you don't have to wait to finish six days.*

MODERATOR: Y . . . comunicado del veterinario ustedes, no está de repente como anotado en alguna . . .
TRANSLATION: *And . . . communication from the veterinarian, it's not suddenly noted in some . . .*

WOMAN: Está en el protocolo, todo está en un protocolo. Sí, entonces el veterinario puede venir aquí y decirnos qué tenemos que hacer o cómo debemos cambiarlo, pero al menos de que se haga final que así va a ser, solamente está escrito a mano. Hasta que se hace un nuevo protocolo a final de seis meses.
TRANSLATION: *It's in the protocol, everything is in the protocol. Yes, then the veterinarian can come here and tell us what we have to do or how we should change it, but unless it's definitive that's how it's going to be, it's just written by hand. Until they make a new protocol after six months.*

MODERATOR: Okay. Perfecto. A ver, ¿y quién en la granja tiene permiso para usar los antibióticos en los animales?
TRANSLATION: *Okay. Perfect. Let's see, and who on the farm has permission to use the antibiotics on the animals?*

WOMAN: Sí, veterinario.
TRANSLATION: *Yeah, veterinarian.*

MODERATOR: El veterinario.
TRANSLATION: *The veterinarian.*

WOMAN: Bueno, él lo autoriza.
TRANSLATION: *Well, he authorizes it.*

MODERATOR: Él autoriza.
TRANSLATION: *He authorizes.*

WOMAN: Mm-hmm.

MODERATOR: ¿Y los que lo administran son?
TRANSLATION: *And those who administer it?*

WOMAN: Nosotros. Bueno, él y nosotros.
TRANSLATION: *Us. Well, him and us.*

MODERATOR: Oh. Okay. Bueno, las próximas preguntas son sobre el trabajo aquí en la granja, la ropa y el tipo de protección que se utilice cuando hacen estos trabajos. ¿Piensan que en la granja dan algún tipo de ropa de protección para que administran? ¿Piensan que la granja, hay unas cosas que la granja provee que ayudan a protegerse en el trabajo?
TRANSLATION: *Oh. Okay. Well, the next questions are about work here on the farm, the clothes and the type of protection that is used when you do these jobs. Do you think the farm gives some type of protective clothing that they give out? Do you think that the farm, are there some things that the farm provides that help you to protect yourself at work?*

WOMAN: Sí.
TRANSLATION: *Yes.*

MAN: Sí.

TRANSLATION: *Yes.*

MAN: Sí.
TRANSLATION: *Yes.*

MAN: Sí.
TRANSLATION: *Yes.*

WOMAN: Y tenemos muchos colores.
TRANSLATION: *And we have lots of colors.*

MODERATOR: ¿Muchos colores?
TRANSLATION: *Lots of colors?*

WOMAN: Negro, azul, de rosa . . .
TRANSLATION: *Black, blue, pink . . .*

MAN: Rosita.
TRANSLATION: *Pink.*

WOMAN: Próximamente, morado.
TRANSLATION: *Next, purple.*

MODERATOR: Está bien, ¿y qué tipo de cosas son?
TRANSLATION: *Okay, and what types of things are they?*

WOMAN: Son . . .
TRANSLATION: *They're . . .*

MODERATOR: ¿Cómo?
TRANSLATION: *What?*

WOMAN: Son bibs.
TRANSLATION: *They are bibs.*

MODERATOR: Okay.

MAN: Guantes.
TRANSLATION: *Gloves.*

WOMAN: O guantes.
TRANSLATION: *Or gloves.*

MAN: Overoles.
TRANSLATION: *Overalls.*

MAN: Lentes.
TRANSLATION: *Glasses.*

MAN: Los lentes.
TRANSLATION: *The glasses.*

MAN: Guantes.
TRANSLATION: *Gloves.*

MAN: Guantes.
TRANSLATION: *Gloves.*

MAN: Botas.
TRANSLATION: *Boots.*

WOMAN: Unas mangas.
TRANSLATION: *Some sleeves.*

MAN: Las mangas.
TRANSLATION: *The sleeves.*

MAN: Sí, te provee todo para que te sientas confortable cuando tú andas trabajando con los animales.
TRANSLATION: *Yeah, they provide everything for you so that you feel comfortable when you're working with the animals.*

MODERATOR: Okay. Bueno, ¿cuáles son algunas cosas que hacen para mantener la salud tuya y de las vacas?
TRANSLATION: *Okay. Well, what are some things that they do to maintain your and cows' health?*

WOMAN: Usar guantes.
TRANSLATION: *Using gloves.*

MAN: Usar guantes.
TRANSLATION: *Using gloves.*

MODERATOR: O sea . . . protección. Okay. ¿Cuáles son algunas, bueno, creo que ya lo hemos tocado un poco, pero cuáles son algunas de las cosas que están obligados a utilizar en la granja para proteger tu salud? Si tiene allí algún uniforme que ustedes pueden utilizar y si así sí la granja se lo provee, cosas como overoles, guantes, protección de ojos, mascara facial, mascara de filtro, cubierta de zapatos?
TRANSLATION: *I mean . . . protection. Okay. What are some, well, I believe that we already touched on this a little, but what are some of the things that you are obligated to use on the farm to protect your health? If you have some uniform that you can use and if the farm provides it to you, things like overalls, gloves, eye protection, face mask, filter mask, shoe covers?*

MAN: Sí, todo.
TRANSLATION: *Yes, everything.*

MAN: No se me tocó . . .
TRANSLATION: *I haven't needed . . .*

MAN: Oh, te tocó.
TRANSLATION: *Oh, you needed to.*

MODERATOR: Okay. Entonces, dicen que sí proveen, por ejemplo, guantes.

TRANSLATION: *Okay. So you're saying that, yes, they provide, for example, gloves.*

WOMAN: Sí.
TRANSLATION: *Yes.*

MAN: Sí.
TRANSLATION: *Yes.*

MAN: Sí.
TRANSLATION: *Yes.*

MAN: Sí.
TRANSLATION: *Yes.*

MODERATOR: ¿Cuándo utilizan las guantes cuando están en la granja?
TRANSLATION: *When do you use the gloves when you are on the farm?*

MAN: Todo el día casi. Todo el tiempo.
TRANSLATION: *Almost all day. All the time.*

MAN: Menos para lonchar . . .
TRANSLATION: *Except to have lunch . . .*

MAN: Vacas . . . para cuando van a poner, ponen los guantes.
TRANSLATION: *Cows . . . for when you're going to put, you put on the gloves.*

MODERATOR: ¿Qué tipo de guantes son, son de cuero?
TRANSLATION: *What type of gloves are they, are they leather?*

MAN: Látex.
TRANSLATION: *Latex.*

MAN: El látex.
TRANSLATION: *Latex.*

MODERATOR: Oh. Okay. ¿Con qué frecuencia cambian o limpian los guantes?
TRANSLATION: *Oh. Okay. How often do you change or clean the gloves?*

MAN: Cada 15 minutos, cada 10 minutos.
TRANSLATION: *Every 15 minutes, every 10 minutes.*

MAN: Son desechables.
TRANSLATION: *They're disposable.*

MAN: Desechables. Entonces quito . . .
TRANSLATION: *Disposables. So I take off . . .*

MAN: A discreción de cada quien, digamos.
TRANSLATION: *At your own discretion, let's say.*

MODERATOR: Okay. Y lo mismo con los overoles, ¿cuándo utilizan los overoles en la granja, todo el tiempo?
TRANSLATION: *Okay. And the same with the overalls, when do you guys the overalls on the farm, all the time?*

MAN: Todo el tiempo.
TRANSLATION: *All the time.*

WOMAN: Todo el día.
TRANSLATION: *All day.*

MAN: Todo el tiempo, todo el día.
TRANSLATION: *All the time, all day.*

MODERATOR: Okay. ¿Y con qué frecuencia lavan los overoles?
TRANSLATION: *Okay. And how often do you wash the overalls?*

MAN: Todos los días.
TRANSLATION: *Every day.*

MAN: Cada media hora.
TRANSLATION: *Every half hour.*

MODERATOR: Oh. Okay. ¿Se lavan las manos durante el día de trabajo?
TRANSLATION: *Oh. Okay. Do you wash your hands during the workday?*

MAN: Sí.
TRANSLATION: *Yes.*

MAN: Sí.
TRANSLATION: *Yes.*

MODERATOR: Okay. Bueno, más o menos, ¿cuántas veces se lavan las manos? ¿Y qué utilizan para lavar las manos, agua, jabón, gel antiséptico?
TRANSLATION: *Okay. Well, more or less, how many times do you wash your hands? And what do you use to wash your hands, water, soap, hand sanitizer?*

MAN: Los dos.
TRANSLATION: *Both.*

MAN: Las dos cosas.
TRANSLATION: *Both things.*

MAN: Los dos.
TRANSLATION: *Both.*

MAN: Todo.
TRANSLATION: *Everything.*

MODERATOR: ¿Hay lugares accesibles fácilmente para lavar las manos cuando sienten que necesitan hacerlo?

TRANSLATION: *Are there easily accessible places for you to wash your hands when you feel you need to do so?*

WOMAN: Sí.
TRANSLATION: *Yes.*

MAN: Sí.
TRANSLATION: *Yes.*

MAN: Sí.
TRANSLATION: *Yes.*

MAN: Sí.
TRANSLATION: *Yes.*

MAN: Hasta para los ojos, si te cae caca, luego los lavas los ojos.
TRANSLATION: *Even for your eyes, if shit falls on you, then you wash your eyes out.*

MODERATOR: ¿Lavarse las manos alguna vez interfiere con su capacidad para completar los deberes asignados?
TRANSLATION: *Has washing your hands ever interfered with your ability to complete your assigned tasks?*

WOMAN: No.

MAN: No.

MAN: No.

MAN: Te lavas las manos y te pones los guantes.
TRANSLATION: *You wash your hands and you put on gloves.*

MODERATOR: ¿El empleado tiene algunas reglas sobre el lavado de manos o sobre el uso de equipo protector? Si es así, ¿cuáles son las reglas? O sea, si dan ciertas instrucciones como cada cuanto tiempo hay que lavarse las manos, cómo hacerlo, cómo utilizar el equipo protector para que . . . hacerlo. ¿Hay como reglas sobre toda esa actividad?
TRANSLATION: *Does the employee have some rules about washing hands or about the use of protective equipment? If so, what are the rules? I mean, if they give certain instructions like how often you have to wash your hands, how to do it, how to use protective equipment so that . . . do it. Are there rules about all of that activity?*

MAN: Sí.
TRANSLATION: *Yes.*

WOMAN: Sí, sí.
TRANSLATION: *Yes, yes.*

MAN: Sí, sí hay.
TRANSLATION: *Yes, there is.*

MODERATOR: Okay. ¿Cómo le informan el empleado sobre esas reglas?
TRANSLATION: *Okay. How do they inform the employee about those rules?*

WOMAN: Con cada junta.
TRANSLATION: *With every meeting.*

MAN: Hay miles reuniones.
TRANSLATION: *There are thousands of meetings.*

MAN: Es cuando hablas también del procedimiento.
TRANSLATION: *When you talk about procedure as well.*

MODERATOR: ¿Cuál es su opinión sobre el uso de estas medidas y esto protector?
TRANSLATION: *What is your opinion about the use of these measures and this protection?*

MAN: Que ayuda.
TRANSLATION: *It helps.*

MAN: Es para el bien de uno, si los ocupas.
TRANSLATION: *It's for your own good, if you use it.*

MAN: Por tu salud.
TRANSLATION: *For your health.*

MAN: Por tu salud, dice.
TRANSLATION: *For your health, he says.*

MODERATOR: Okay. Al momento de salir de la granja, ¿cómo te preparas para salir del trabajo para el día, hay un vestuario que utiliza o es el mismo que utiliza en el trabajo?
TRANSLATION: *Okay. When you leave the farm, how do you prepare yourself to leave work for the day, is there clothing that you use or is it the same that you use at work?*

MAN: Es diferente.
TRANSLATION: *It's different.*

MODERATOR: Diferente.
TRANSLATION: *Different.*

WOMAN: Depende cuanta prisa lleve.
TRANSLATION: *Depends on how in a hurry you are.*

MODERATOR: Si se puede que un día que está muy apresurado, sale con la ropa del trabajo a la casa?
TRANSLATION: *So it's possible that on a day that you are in a hurry, you leave to go home with your work clothes on?*

WOMAN: Mm-hmm.

MODERATOR: Está bien. Ahorita, ¿llevas la ropa y los zapatos de trabajo a la casa?
TRANSLATION: *Okay. Now, do you wear work clothes and shoes home?*

MAN: No, no, es que se quedan.
TRANSLATION: *No, no, they stay.*

MAN: Normalmente no, pero unas veces, sí, lleva, ¿no?
TRANSLATION: *Normally no, but sometimes, yes, you wear it, right?*

MAN: Usted, yo no. Yo no.
TRANSLATION: *You, not me. Not me.*

MAN: Yo tampoco.
TRANSLATION: *Me either.*

MAN: Nunca las he llevado para allá.
TRANSLATION: *I've never worn them there.*

MAN: Yo no.
TRANSLATION: *Not me.*

MAN: Mire, nada.
TRANSLATION: *Look, nothing.*

MODERATOR: Algunos me dijeron que trabajan con becerros y vacas enfermas, ¿verdad?
TRANSLATION: *Some of you told me that you work with calves and sick cows, right?*

WOMAN: Sí.
TRANSLATION: *Yes.*

MAN: Sí.
TRANSLATION: *Yes.*

MODERATOR: Okay. ¿Lleva ropa especial o ropa diferente con estos animales?
TRANSLATION: *Okay. Do you wear special clothing or different clothing with those animals?*

WOMAN: Sí.
TRANSLATION: *Yes.*

MAN: Siempre los overoles.
TRANSLATION: *Always the overalls.*

WOMAN: Overoles, mm-hmm.
TRANSLATION: *Overalls, mm-hmm.*

MODERATOR: ¿Qué tan efectivo piensa que el uso de la ropa protectora es para mantenerte seguro cuando trabaja con animales enfermos?
TRANSLATION: *How effective do you think the use of protective clothing is to stay safe when you are working with sick animals?*

MAN: Hasta ahorita, no se ha enfermado uno.
TRANSLATION: *No one has gotten sick yet.*

MODERATOR: Okay. ¿Y no siente que cuando trabaja con vacas o becerros enfermos, siente riesgo de contraer una enfermedad de ellos?
TRANSLATION: *Okay. And you don't feel that when you work with sick cows or calves, do you feel a risk of contracting a disease from them?*

MAN: No.

WOMAN: No.

MAN: Hmm-mm.

WOMAN: Porque te estás cambiando de los guantes y el overol.
TRANSLATION: *Because you're changing gloves and overalls.*

MAN: Uh-huh.

MODERATOR: ¿Por el uso de la protección?
TRANSLATION: *Because of the use of protection?*

WOMAN: Mm-hmm.

MAN: Por la protección.
TRANSLATION: *Because of the protection.*

MODERATOR: Ya. La última pregunta, dialoguen, y estamos casi terminando, ¿cómo te sientes sobre el uso de los guantes, overoles, o protección de los . . .
TRANSLATION: *Okay. The last question, talk about it, and we're almost done, how do you feel about the use of gloves, overalls, or protection for . . .*

MAN: . . . todo el día, no creo que . . .
TRANSLATION: *. . . all day, I don't think that . . .*

MAN: ¿Cómo?
TRANSLATION: *What?*

MODERATOR: ¿Cómo te sientes sobre el uso de guantes, overoles, o protección de ojo? ¿Se siente que . . .
TRANSLATION: *How do you feel about the use of gloves, overalls, or eye protection? Do you feel that . . .*

MAN: Algo estándar. Andas más . . .
TRANSLATION: *It's something standard. You are . . .*

MAN: Seguro.
TRANSLATION: *Safe.*

MAN: . . . seguro trabajando, cómodo. Sí.
TRANSLATION: *. . . safer working, comfortable. Yeah.*

WOMAN: No, sí, cómodo también porque, de allí, ensuciar tu ropa.

TRANSLATION: *No, yeah, comfortable as well because, from there, you get your clothes dirty.*

MAN: Oh, sí, y luego luego te cambias otra vez. Eso sí. Si andas . . . mal, que ya estás bien sucio, allí hay más ropa y allí te cambias, y otra vez . . .
TRANSLATION: *Oh, yeah, and then you change again. That's true. If you go . . . bad, you're already really dirty, there is more clothing there and you change there, and again . . .*

MAN: De alguien más.
TRANSLATION: *Someone else's.*

MAN: Los que ordeñan, somos que se ensucian más. Somos que nos cambiamos, bueno, no como, yo me cambio unas dos, tres veces.
TRANSLATION: *Those that milk, we're the ones that get the dirtiest. We are the ones that change, well, like I change two, three times.*

MODERATOR: ¿Dónde comen sus comidas cuando están en el trabajo? ¿Hay una sala de descanso, un lugar especifico para comer las comidas?
TRANSLATION: *Where do you eat your meals when you are at work? Is there a break room, a specific place to eat meals?*

MAN: En la cocina.
TRANSLATION: *In the kitchen.*

MAN: Sí, hay un comedor.
TRANSLATION: *Yeah, there's a dining room.*

MAN: Comedor. Bueno, comedor, oficina.
TRANSLATION: *Dining room. Well, dining room, office.*

MAN: Cuarto de reunión.
TRANSLATION: *Meeting room.*

MODERATOR: Okay. ¿No comen, no toca a veces comer en el lugar de trabajo?
TRANSLATION: *Okay. You don't eat, you don't sometimes have to eat where you're working?*

MAN: Sí.
TRANSLATION: *Yes.*

MAN: Sí.
TRANSLATION: *Yes.*

MAN: Ehh, y todo el día.
TRANSLATION: *Ehh, and all day.*

MAN: Sí. La verdad que sí . . .
TRANSLATION: *Yes. Honestly, yes . . .*

MAN: Te traes un snack en la bolsa, y te vas a caminar y vas comiendo.
TRANSLATION: *You bring a snack in your bag, and you go walking and you're eating.*

MAN: Puchando vacas con un taco.
TRANSLATION: *Pushing cows with a taco.*

MAN: De taco en la mano.
TRANSLATION: *Taco in hand.*

MAN: Como está pegado a la vaca.
TRANSLATION: *Like you're stuck to the cow.*

MAN: Pizza, ordeñando.
TRANSLATION: *Pizza, milking.*

MAN: Pizza.

MAN: Es asco.
TRANSLATION: *That's gross.*

MODERATOR: ¿Cómo se maneja la leche cruda y los productos de la lecha cruda en esta granja?
TRANSLATION: *How do you handle the raw milk and the raw milk products on this farm?*

WOMAN: Nadie tiene acceso de, bueno, sí hay acceso, pero nadie de la . . .
TRANSLATION: *No one has access to, well, yeah, there's access, but no one from the . . .*

MAN: Puedes. No debes.
TRANSLATION: *You can. You shouldn't.*

MODERATOR: ¿Y consumen leche cruda o los productos de la leche cruda?
TRANSLATION: *And do you consume raw milk or raw milk products?*

MAN: No.

MAN: No.

MAN: No.

MODERATOR: Okay. ¿Hay reglas en la granja sobre el consumo de leche cruda?
TRANSLATION: *Okay. Are there rules on the farm about the consumption of raw milk?*

MAN: Sí.
TRANSLATION: *Yes.*

MAN: Si te la tomas, te pagas.
TRANSLATION: *If you drink it, you pay for it.*

MAN: Siempre la tomas, te enfermas, dice. Te enfermas, también.
TRANSLATION: *Whenever you drink it, you get sick, he says. You get sick also.*

MAN: Y la paga, también.
TRANSLATION: *And you pay for it also.*

MODERATOR: Okay. Y la última pregunta, ¿piensan que hay cualquier riesgo, hay un tipo de riesgo de beber leche cruda o consumir alimentos hecho con leche cruda?
TRANSLATION: *Okay. And the last question, do you think that there is a risk, is there some type of risk drinking raw milk or consuming foods made from raw milk?*

MAN: Sí.
TRANSLATION: *Yes.*

MAN: Sí.
TRANSLATION: *Yes.*

MAN: Sí.
TRANSLATION: *Yes.*

MAN: Se agarra diarrea luego luego. Al momento, sí.
TRANSLATION: *You get diarrhea then. Right away, yeah.*

MAN: Sí.
TRANSLATION: *Yeah.*

WOMAN: Yo no sé.
TRANSLATION: *I don't know.*

MAN: Lo digo, no.
TRANSLATION: *I say so, you know.*

MAN: Sí, porque las bacterias están disponibles, ¿no? No está pasteurizada.
TRANSLATION: *Yes, because the bacteria are available, right? It's not pasteurized.*

MAN: Al menos que ya está fría o pasteurizada, sí, como a la mejor menos daño.
TRANSLATION: *Unless it's already cold or pasteurized, yeah, like maybe less harm.*

[Simultaneous discussion]

WOMAN: . . . que tan limpio crees que está.
TRANSLATION: *. . . how clean you think it is.*

MAN: Eso también.
TRANSLATION: *That too.*

WOMAN: O sea, la mayoría tiene bacteria . . .
TRANSLATION: *I mean, the majority have bacteria . . .*

MAN: Donde quiere, hay bacterias aquí.
TRANSLATION: *Wherever you go, there are bacteria here.*

MODERATOR: Bueno, perfecto. Gracias por su tiempo, y ya estamos listo con las preguntas. Muchísimas gracias por participar. Ahorita . . . que firmen y retiren su efectivo.
TRANSLATION: *Well, perfect. Thank you for your time, and we are now good with the questions. Thank you very much for participating. Now . . . sign and take your cash.*

MAN: ¿Usted es doctor?
TRANSLATION: *Are you a doctor?*

MODERATOR: Sí.
TRANSLATION: *Yes.*

MAN: ¿Veterinario?
TRANSLATION: *Veterinarian?*

MAN: ¿Le puedo hacer una pregunta?
TRANSLATION: *Can I ask you a question?*

MODERATOR: Sí, cómo no.
TRANSLATION: *Yes, of course.*

MAN: O sea, hay una, allí lleva una tendencia de transmisión de enfermedades de animales a humanos, o sea, hay más riesgo obviamente los que trabajan con, en las granjas, ¿no?
TRANSLATION: *I mean, is there a, there's a tendency of transmission of diseases of animals to humans, I mean, there's more risk, obviously, those that work with, on the farms, right?*

MODERATOR: Sí, claro. Por eso, el uso de todo esto, del equipo de protección y tratar de reducir al mínimo el uso de los antibióticos para no crear resistencia antibiótica en el animal. Así crea la resistencia de la bacteria, resistente de un animal, y, pues, se la transmite a la persona, y eso un problema de tratar porque no hay manera de hacer. Entonces, justamente este estudio son preguntas de este estilo para identificar donde es más fácil la transmisión de la vaca, del animal, a la persona y tratar de eliminar esos puntos, ¿me entiende?
TRANSLATION: *Yes, of course. For that reason, the use of all this, of the protective equipment and trying to reduce to a minimum the use of antibiotics in order to not create antibiotic resistance in the animal. That's how the bacterium's resistance is created, resistant in an animal, and, well, it's transmitted to a person, and that's a problem to treat because there is no way to do it. So, precisely this study has questions of that style to identify where transmission from the cow, the animal, to the person is easiest and try to eliminate those points, does that make sense?*

MAN: Yo hasta pensaba que, digamos, que las bacterias eran especificas . . . al especie que no brincaba muchas veces . . .
TRANSLATION: *I was even thinking that, let's say, that the bacteria were specific . . . to the specie that doesn't jump a lot . . .*

MODERATOR: Hay unas que sí, otras que no. Entonces unos que nada más que agarran las vacas y no transmiten al humano, y hay unos que sí son como de vacas a humanos, otros animales, perros, tú sabes. Entonces esas son las más, claro, son las más delicadas, las que estamos tratando acá. Porque las que atacan los animales, bueno, muy triste por el animal, pero por lo menos no atacan a la persona, ¿no? Entonces, bueno, en . . . gracias de este estudio . . . identificar esa bacteria.
TRANSLATION: *There are ones that do, others that don't. So there are some that just get the cows and they don't transmit to humans, and there are some that are like from cows to humans, other animals, dogs, you know. So those are the most, of course, they're the tricky ones, the ones that we're talking about here. Because the ones that attack animals, well, very sad for the animal, but at least they're not attacking the person, right? So, well, in . . . thanks to this study . . . to identify that bacteria.*

MAN: Ya, gracias.
TRANSLATION: *Okay, thank you.*

MODERATOR: Como no.
TRANSLATION: *Of course.*

MAN: ¿Cuál sería la bacteria más común en una vaca que te puede transmitir?
TRANSLATION: *What's the most common bacterium in a cow that can transmit to you?*

MODERATOR: ¿La más común? Oye, eso depende yo creo que también de la granja especifica, pero yo creo que varias. La E. Coli, por ejemplo, es uno muy común. Más que nada, que producen diarrea, creo que son las más comunes.
TRANSLATION: *The most common? Listen, that depends I think also on the specific farm, but I think various ones. E. Coli, for example, is a very common one. More than anything, the ones that produce diarrhea, I think they're the most common ones.*

MAN: Por eso, usen guantes.
TRANSLATION: *For that reason, use gloves.*

MODERATOR: Usen guantes, sí.
TRANSLATION: *Use gloves, yes.*

WOMAN: Y lavarse las manos.
TRANSLATION: *And wash your hands.*

MAN: Lavarse las manos directamente. Mm-hmm, ¿ya ven? Porque, hasta ahorita, aquí nadie se ha enfermado, ¿verdad?
TRANSLATION: *Wash your hands directly. Mm-hmm, you see? Because, as of right now, no one has gotten sick, right?*

MAN: Hmm-mm.

MAN: No sé, pronto de diarrea un par de días sí ha sido gente.
TRANSLATION: *I don't know, diarrhea for a few days, yeah, there have been people.*

MAN: Pero porque se llenan de comida, no por la culpa de las vacas. ¿Cómo es culpa de la vaca?
TRANSLATION: *But because they fill up with food, not because of the cows. How is that the cow's fault?*

MAN: Puede ser eso, ¿no?
TRANSLATION: *It could be that, right?*

MAN: Sí.
TRANSLATION: *Yes.*

MAN: O no se lava bien las manos.
TRANSLATION: *Or they don't wash their hands well.*

MAN: Como que la toallita no te protege mucho.

TRANSLATION: *Like the towel doesn't protect you much.*

MAN: Hay que poner una máscara.
TRANSLATION: *You have to put on a mask.*

MAN: . . .

MAN: Oh, porque sale bien protegido antes, ¿verdad? Este hombre, sí, pone una máscara aquí, se pone otra en la cabeza, se ve, está bien cubierto el hombre.
TRANSLATION: *Oh, because you go out well protected before, right? This guy, yeah, he puts a mask here, puts another on his head, he looks, the guy is well covered.*

MAN: Parece un jugador de hockey.
TRANSLATION: *He looks like a hockey player.*

MAN: Así ni más ni menos.
TRANSLATION: *Nothing more, nothing less.*

[Simultaneous discussion, banter]

MAN: Pues, esperemos que les haiga servido la información.
TRANSLATION: *Well, we hope this information has been useful to you guys.*

MODERATOR: Sí, vale, muchas gracias de nuevo por su tiempo. Sé que son varias preguntas y son así como medio repetitivas, pero.
TRANSLATION: *Yes, okay, thank you very much again for your time. I know that there are various questions that are like kind of repetitive, but.*

MAN: Sí, hay preguntas repetitivas.
TRANSLATION: *Yeah, there are repetitive questions.*

[Simultaneous discussion]
